# Early hominins from Morocco basal to the *Homo sapiens* lineage

**DOI:** 10.1038/s41586-025-09914-y

**Published:** 2026-01-07

**Authors:** Jean-Jacques Hublin, David Lefèvre, Serena Perini, Giovanni Muttoni, Matthew M. Skinner, Shara E. Bailey, Sarah Freidline, Philipp Gunz, Mathieu Rué, Mohssine El Graoui, Denis Geraads, Camille Daujeard, Thomas W. Davies, Kornelius Kupczik, Mykolas D. Imbrasas, Alejandra Ortiz, Christophe Falguères, Qingfeng Shao, Jean-Jacques Bahain, Alain Queffelec, Asier Gómez-Olivencia, Stefano Benazzi, Adeline Le Cabec, Rita Sorrentino, Inga Bergmann, Fatima-Zohra Sbihi-Alaoui, Rosalia Gallotti, Jean-Paul Raynal, Abderrahim Mohib

**Affiliations:** 1 Chaire de Paléoanthropologie, CIRB, Collège de France, Université PSL, CNRS, Paris, France; 2 Max Planck Institute for Evolutionary Anthropology, Leipzig, Germany; 3 LabEx Archimède and ASM-UMR 5140, Université de Montpellier Paul Valéry, CNRS, Campus Saint Charles, Montpellier, France; 4 Dipartimento di Scienze della Terra “A. Desio”, Università degli Studi di Milano, Milan, Italy; 5 Centre for the Study of Human Origins, Dept. Anthropology, New York University, New York, NY USA; 6 Department of Anthropology, University of Central Florida, Orlando, FL USA; 7 Department of Human Origins, Max Planck Institute for Evolutionary Anthropology, Leipzig, Germany; 8 SARL Paléotime, Villard-de-Lans, France; 9 Université de Montpellier Paul Valéry, CNRS, ASM UMR 5140, Campus Saint Charles, Montpellier, France; 10 Institut National des Sciences de l’Archéologie et du Patrimoine, Rabat, Morocco; 11 CR2P-UMR 7207, CNRS, MNHN, Sorbonne Université, CP 38, Paris, France; 12 HNHP-UMR 7194, CNRS, MNHN, UPVD, Paris, France; 13 Department of Evolutionary Biology, University of Vienna, Vienna, Austria; 14 Departamento de Antropología, Facultad de Ciencias Sociales, Universidad de Chile, Santiago de Chile, Chile; 15 HNHP-UMR 7194, CNRS, MNHN, UPVD, Alliance Sorbonne Université, Institut de Paléontologie Humaine, Paris, France; 16 College of Geography Science, Nanjing Normal University, Nanjing, China; 17 Université de Bordeaux, CNRS, Ministère de la Culture, PACEA, UMR 519, Pessac, France; 18 Departamento de Geología, Facultad de Ciencia y Tecnología, Universidad del País Vasco/Euskal Herriko Unibertsitatea (UPV/EHU), Leioa, Spain; 19 Dipartimento di Beni Culturali, Università di Bologna, Ravenna, Italy; 20 Department of Biological, Geological and Environmental Sciences, Università di Bologna, Bologna, Italy; 21 Direction Provinciale de la Culture, Quartier Administratif, Kenitra, Morocco; 22 Present Address: Université de Bordeaux, CNRS, Ministère de la Culture, PACEA, UMR 519, Pessac, France

**Keywords:** Anthropology, Geomagnetism

## Abstract

Palaeogenetic evidence suggests that the last common ancestor of present-day humans, Neanderthals and Denisovans lived around 765–550 thousand years ago (ka)^1^. However, both the geographical distribution and the morphology of these ancestral humans remain uncertain. The *Homo antecessor* fossils from the TD6 layer of Gran Dolina at Atapuerca, Spain, dated between 950 ka and 770 ka (ref. ^2^), have been proposed as potential candidates for this ancestral population^3^. However, all securely dated *Homo sapiens* fossils before 90 ka were found either in Africa or at the gateway to Asia, strongly suggesting an African rather than a Eurasian origin of our species. Here we describe new hominin fossils from the Grotte à Hominidés at Thomas Quarry I (ThI-GH) in Casablanca, Morocco, dated to around 773 ka. These fossils are similar in age to *H. antecessor*, yet are morphologically distinct, displaying a combination of primitive traits and of derived features reminiscent of later *H. sapiens* and Eurasian archaic hominins. The ThI-GH hominins provide insights into African populations predating the earliest *H. sapiens* individuals discovered at Jebel Irhoud in Morocco^4^ and provide strong evidence for an African lineage ancestral to our species. These fossils offer clues about the last common ancestor shared with Neanderthals and Denisovans.

## Abstract (web summary)

New hominin fossils from the Grotte à Hominidés at Thomas Quarry I (ThI-GH) in Casablanca, Morocco, dated to around 773 thousand years ago are similar in age to *Homo antecessor*, yet are morphologically distinct.

## Main

Our understanding of the evolutionary history of both Neanderthals (*Homo neanderthalensis*) and *H. sapiens* is firmly grounded in morphological, genetic and archaeological analyses of extensive fossil hypodigms and numerous prehistoric sites across Europe and Africa. However, identifying the last common ancestor of these two species remains challenging. At times, *Homo heidelbergensis* was proposed as this ancestor^5^. Yet, anatomical and chronological evidence suggests that fossils assigned to *H. heidelbergensis* may not represent a coherent species^6^. Most of the Eurasian specimens assigned to this species probably belong to the common ancestral form of the Neanderthals and their Asian sister group, the Denisovans, or belong to them, but are not ancestral to *H. sapiens*^6^. Some have considered a Eurasian origin of *H. sapiens*^7^, but the morphological evidence for this is limited. By contrast, recent fossil evidence has pushed back the presence of *H. sapiens* in Africa to over 300 ka (ref. ^4^), highlighting the need to understand hominin diversity in Africa during the late Early Pleistocene (EP) and the first half of the Middle Pleistocene (MP). MP African fossils—such as those from Kabwe (Zambia), Bodo (Ethiopia) and Saldanha (South Africa)—are generally considered close African relatives of *H. heidelbergensis* (or *Homo rhodesiensis*). Among MP African specimens, those from Ndutu (Tanzania) and Salé (Morocco) have been more closely associated with the ancestry of *H. sapiens*^8^.

**Fig. 1:**
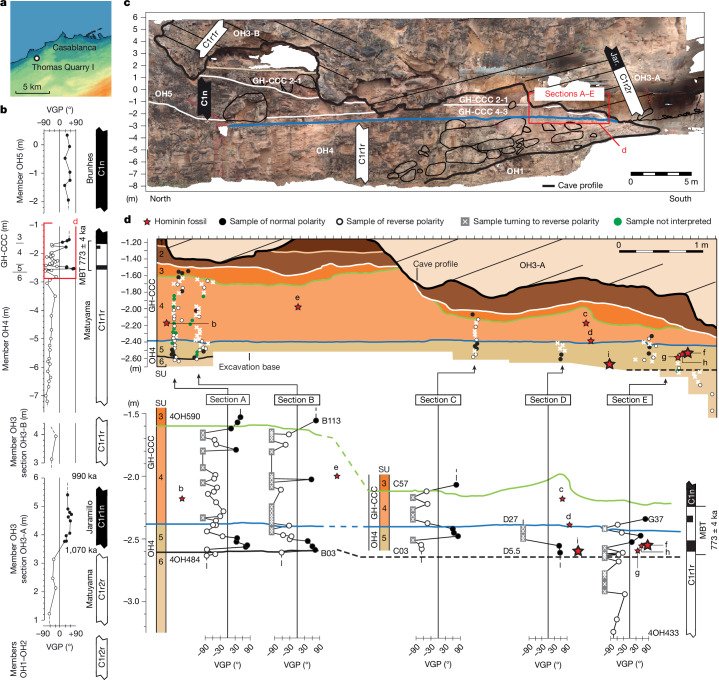
ThI-GH site. **a**, Location map of ThI, modified according to ref. ^13^.** b**, Magnetostratigraphy of members OH3A, OH3B, OH4, GH-CCC and OH5 of ref. ^13^ and this study. The black bars represent normal polarity, and the white bars represent reverse polarity. Further details are provided in Supplementary Fig. 2. Magnetochron ages are from ref. ^22^.** c**, Photograph of the outcrop stratigraphy with indication of magnetic polarity from this study and a previous study^13^ and lithologic members. Here we focused on sections A–E, of which only section A is reported here.** d**, Magnetostratigraphy of sections A–E comprised stratigraphic units OH4 SU6–5 and GH-CCC SU4–3. Context and details for lithostratigraphic units are provided in Extended Data Fig. 2. The red stars with labels represent hominin remains (the larger stars indicate mandibles) (Extended Data Table 1): ThI-GH-UA28-7 (femur, a); ThI-GH-OA23-24 (tooth, b); ThI-GH-SA26-88 (tooth, c); ThI-GH-SA26-90 (tooth, d); ThI-GH-PA24-107 (tooth, e); ThI-GH-10717 (mandible) and ThI-GH-10717/1-5 (vertebrae, f); ThI-GH-10725 and ThI-GH-10725/1 (vertebrae, g); ThI-GH-10726 (vertebra, h); and ThI-GH-10978 (mandible, i). Note that ThI-GH-UA28-7 (a) is located outside the section on the right. Close to the bottom wall of the cavity, its insertion into the stratigraphy is imprecise (SU4/5).

**Extended Data Fig. 1:**
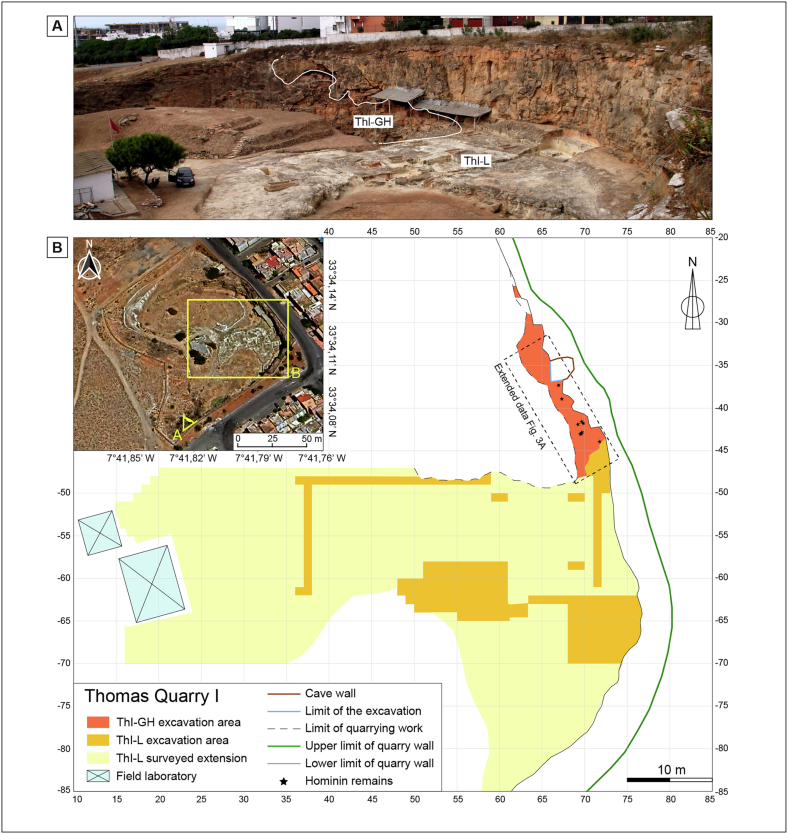
Location of the Hominin Cave in the Thomas Quarry I site. A) General view of the quarry from the southwest (M. Rué). ThI-GH: Grotte à Hominidés. ThI-L: Unit L,early Acheulean site. See details in Extended Data Fig. 2a. B) Excavations plan in the local coordinate system (R. Gallotti, J.-P. Raynal, A. Mohib, M. Rué). Top left: Google Earth view (WGS84 coordinate system).

**Extended Data Fig. 2:**
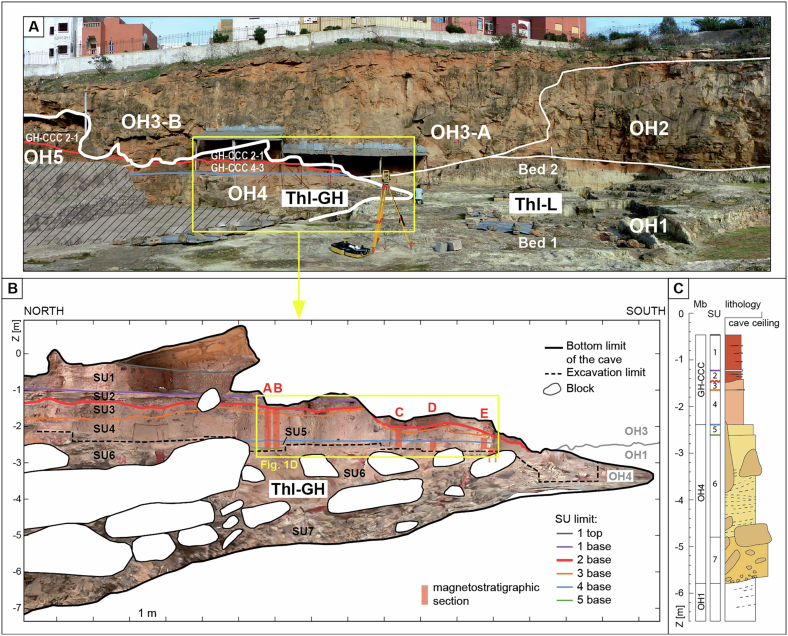
Stratigraphical context of the Grotte à Hominidés (ThI-GH). A) Thomas Quarry I (ThI). The Oulad Hamida Formation: OH1 to OH5 Members and GH - Continental Cave Complex (CCC) (D. Lefèvre, M. Rué). ThI-GH: Grotte à Hominidés. ThI-L: Unit L, early Acheulan site. OH1: coarse calcirudite and calcarenites (Bed 1) overlain by bioclastic sands and limestones (Bed 2); OH2: coarse cross-bedded and finer planar-bedded intertidal biocalcarenites, then massive coarse bioclastic aeolianites; OH3A: massive coarse or coquinoid biocalcarenites, OH3B: clino-stratified aeolianites; OH4: planar-bedded intertidal calcarenites; OH5: cross-bedded coarse aeolian sands; GH-CCC: continental cave deposits. B) Sedimentary infilling of ThI-GH (D. Lefèvre, M. Rué, photogrammetric survey by S. Sanz). C. Synthetic lithostratigraphic log of ThI-GH (M. Rué, D. Lefèvre). Mb: Member, SU: Stratigraphic unit. ThI-GH infilling starts with OH4 marine deposits: calcirudites (SU7) preserved in a notch and plurimetric collapsed blocks of calcarenites and calcirudite with blunt-surfaced onlapped by fine, grey, planar-bedded intertidal biocalcarenites (SU6), and then well-sorted bioclastic and quartzose sands derived from reworked loose littoral deposits (SU5). Without any apparent disconformity, the sequence transitions into continental well-sorted bioclastic and quartzose reddish sands (GH-CCC-SU4 and 3). The upper continental deposits lie in discontinuity on SU3. SU2 consists of multilayer dripstone interbedded with loose red sands, and SU1 comprises massively bedded, rubified sands. At the entrance of the cavity (see A), cross-bedded grey aeolianite sandstones (OH5) are interposed between SU3 and the upper continental deposits SU2-SU1.

Thomas Quarry I (ThI), located in the southwest part of the city of Casablanca, Morocco (Fig. 1a), represents a key archaeological locality in northwest Africa. ThI is excavated in the Oulad Hamida Formation (OHF)^9,10^ and comprises two primary sites (Extended Data Figs. 1 and 2a).

**Fig. 2:**
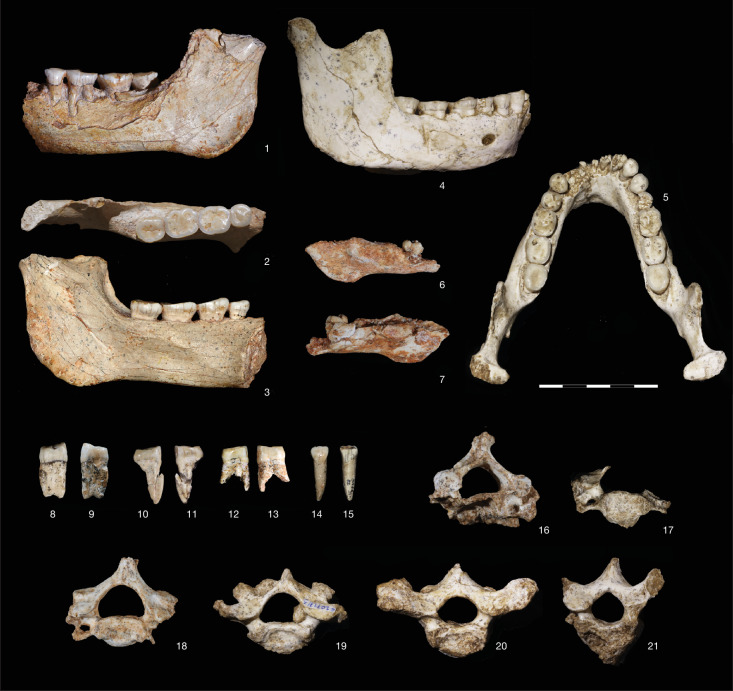
Hominin specimens from ThI-GH. Mandible ThI-GH-1: (1) lateral view; (2) occlusal view; (3) lingual view. Mandible ThI-GH-10717: (4) right lateral view; (5) occlusal view. Mandible ThI-GH-10978: (6) lateral view; (7) lingual view. UP4 ThI-GH-OA23-24: (8) distal view; (9) mesial view. UP3 ThI-GH-PA24-107: (10) distal view; (11) mesial view. UP3 ThI-GH-SA26-90: (12) mesial view; (13) distal view. UI1 ThI-GH-SA26-88: (14) buccal view; (15) lingual view. (16) Fused C2 and C3 vertebrae ThI-GH-10725 and ThI-GH-10725/1, caudal view. (17) C4 vertebra ThI-GH-10717/5, cranial view. (18) C6 vertebra ThI-GH-10717/1, cranial view. (19) C7 vertebra ThI-GH-10717/3, cranial view. (20) T1 vertebra ThI-GH10717/2, cranial view. (21) T2 vertebra ThI-GH-10717/4, cranial view. Scale, 5 cm.

In the oldest member of the OHF, the ThI-L site has yielded one of the most extensive early Acheulean lithic assemblages in Africa, dating back to around 1.3 million years ago^11–13^. The second site is a cave opened in the northeastern wall of the quarry named in 1994 Grotte à Hominidés (hereafter, ThI-GH) by the research team. In 1969, Philippe Beriro, an amateur collector, found a partial hominin mandible (ThI-GH-1) (Fig. 2) on a slope below the northwestern part of this cave, along with other mammal fossils and lithics. This material probably originated from the filling of the ThI-GH cave, which had been partially disturbed by quarrying activities^14,15^. ThI-GH-1 was initially described as *Atlanthropus mauritanicus*^16^. Subsequent systematic investigations at ThI-GH, carried out between 1994 and 2015, yielded an Acheulean industry, a diverse faunal assemblage and several additional hominin fossils in an undisputable stratigraphic context thanks to modern controlled excavations^17–19^.

**Extended Data Fig. 3:**
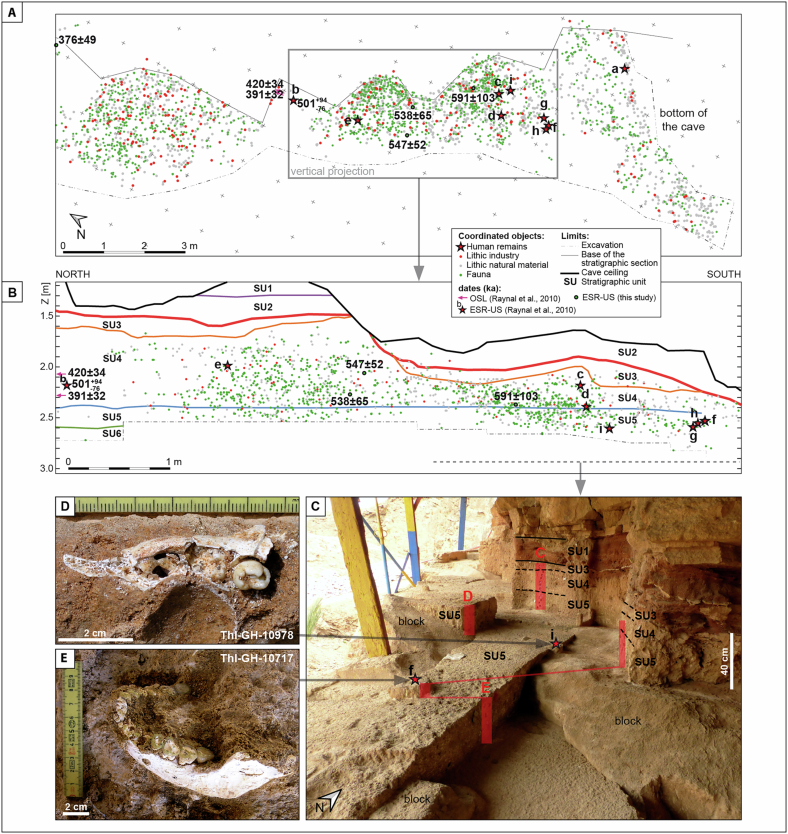
Excavations at ThI-GH. A) Horizontal and B) vertical distribution of all coordinated remains including hominin fossils and OSL/ESR-U series dating (M. Rué, R. Gallotti, A. Mohib, J.-P. Raynal). Same location as Fig. 1d. The red stars with labels represent hominin remains: a: ThI-GH-UA28-7 (femur); b: ThI-GH-OA23-24 (tooth); c: ThI-GH-SA26-88 (tooth); d: ThI-GH-SA26-90 (tooth); e: ThI-GH-PA24-107 (tooth); f: ThI-GH-10717 (mandible), ThI-GH-10717/1-5 (vertebrae); g: ThI-GH-10725 and ThI-GH-10725/1 (vertebrae); h: ThI-GH-10726 (vertebra); i: ThI-GH-10978 (mandible). The stratigraphic insertion of femur ThI-GH-UA28-7 (a) located near the bottom wall of the cavity is uncertain (SU4/5). C) Photograph of the excavation (D. Lefèvre, M. Rué). SU: stratigraphic unit (see Extended Data Fig. 2). Section C, D and E: magnetostratigraphic sections (see Fig. 1d). Red stars: hominin remains: f (ThI-GH-10717) and i (ThI-GH-10978). D) and E) Photographs of hominin fossils ThI-GH-10978 and ThI-GH-10717 (Photographs J.-P. Raynal).

ThI-GH is a cave that was carved during a marine high-stand into the older marine-aeolian OH1 and OH3 deposits of the OHF. It was filled by marine (OH4 stratigraphic unit 6, SU6) then supratidal (SU5) deposits and, without any discontinuity, by continental deposits (GH-CCC SU4 and SU3). Then, aeolian deposits (OH5) separated the latter from upper continental deposits (SU2 and SU1)^10,18^ (Fig. 1c, Extended Data Fig. 2, Supplementary Note 1 and Supplementary Fig. 3). A rich palaeontological assemblage has been recovered from OH4 SU5 and GH-CCC SU4, with hominin remains and lithic artifacts^17–20^ (Extended Data Fig. 3a,b and Supplementary Notes 2 and 3). The abundance of carnivores, numerous coprolites and carnivore-modified bone remains lacking evidence of cut or chop marks, combined with the scarcity of lithic artifacts, point to the presence of a carnivore den^21^ (Supplementary Note 2). The most representative hominin specimens have been found in SU5, including an adult mandible (ThI-GH-10717), eight associated vertebrae (ThI-GH-10717/1 to 5, ThI-GH-10725, ThI-GH-10726 and ThI-GH-10725/1) and a fragmentary mandible (ThI-GH-10978) of a child who died aged at most 1.5 years (Fig. 2 and Supplementary Note 7). A portion of a hominin femoral shaft (ThI-GH-UA28-7) scavenged by a large carnivore, probably a hyena^21^, was found at the back of the cavity in a layer belonging to SU4 or SU5. Although the precise stratigraphic origin of the ThI-GH-1 hemimandible remains uncertain, sedimentological analysis of the embedding sediment suggests that it also probably derives from either SU4 or SU5 (ref. ^18^).

## Dating

We proposed a chronostratigraphic and depositional model for the OHF within a sequence stratigraphy framework shaped by Pleistocene sea-level fluctuations and moderate regional uplift^9,10,13^. Sea-level transgressive phases mark calcarenite onlap and the carving of cliff and erosional notch at the base of previously lithified aeolian dunes, whereas regressive phases involve seaward progradation and the buildup of new dunes. Early cementation in semiarid, bioclastic-rich coastal settings allows rapid lithification^9^, enabling successive transgressive erosion and cliff formation (Supplementary Note 1 and Supplementary Fig. 2). A previous study^13^ placed the Matuyama–Brunhes transition (MBT, 773 ka)^22^ close to the base of SU4 and recognized the Jaramillo subchron (1,070–990 ka) in member OH3. This interpretation excludes hiatus long enough to imply older subchrons like the Olduvai in place of the Jaramillo, which would also contradict the Acheulean lithics found at ThI-L. However, the preliminary sampling within the GH-CCC SU4 and OH4 SU5 deposits containing the human remains (five samples in section A) did not allow precise placement of the MBT in relation to these remains. We refined this model by adding 119 new magnetostratigraphic samples (Methods and Supplementary Note 5) from OH3, OH4 and GH-CCC to the 62 from ref. ^13^, improving the resolution of the Jaramillo and the MBT^22^ (Fig. 1b,c).

Characteristic remanent magnetization (ChRM) component directions of samples from two different sections yielded virtual geomagnetic pole (VGP) latitudes indicating that the Jaramillo subchron lies within member OH3. Most of the samples from SU6 to SU3 (Fig. 1c,d (sections A–E)) provided VGP latitudes of reverse magnetic polarity or ChRM directions showing a tendency towards reverse polarity (Supplementary Note 5). This post-Jaramillo interval of dominant reverse polarity is punctuated by a thin normal polarity excursion in SU5. Above, a reverse-to-normal polarity transition occurs in GH-CCC close to the SU4–SU3 contact, with stable normal (Brunhes) polarity extending into GH-CCC-SU3 (Fig. 1d) and continuing into younger OH5 deposits (Fig. 1c).

**Extended Data Fig. 4:**
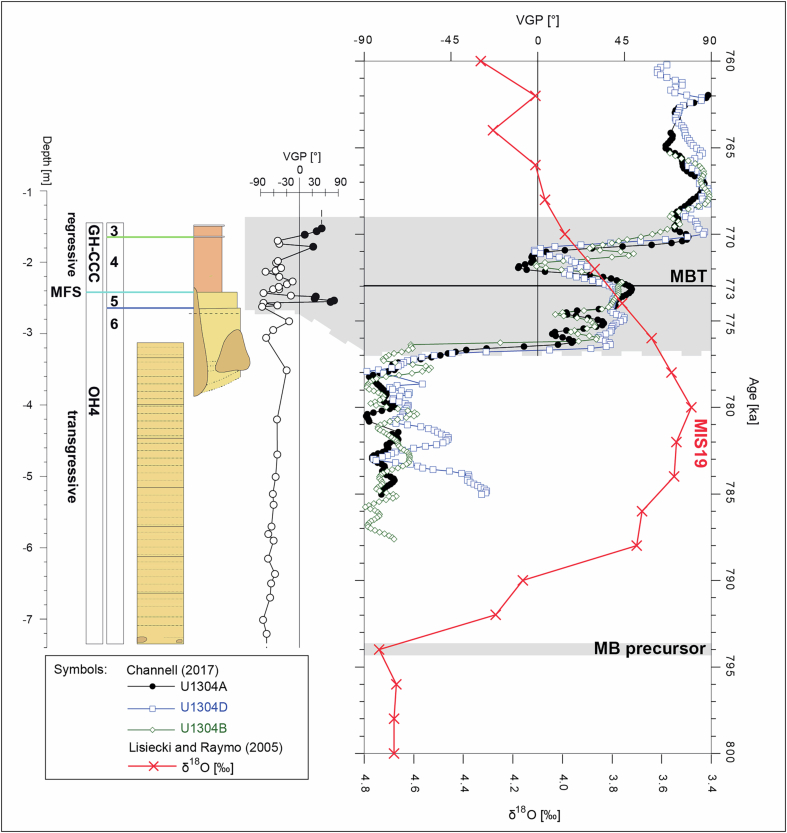
MBT record and MIS 19. Comparison of magnetostratigraphic (VGP) data across the MBT in Members OH4 and GH-CCC of Thomas Quarry I and in North Atlantic IODP deep-sea cores^24^, correlated to the δ^18^O [‰] stack of 8 centred on Marine Isotope Stage (MIS) 19. Considering that the MBT (773 ± 4 ka) occurs during MIS 19c to MIS 18e^24^ and that the sea-level transgression of MIS20/MIS19 began at ~795 ka^26^, we interpreted the intertidal biocalcarenites of OH4-SU6 and the littoral sands of OH4-SU5 as part of this transgressive phase culminating whit a maximum flooding surface corresponding to the top of OH4-SU5. The following continental deposits of GH-CCC-SU4-3 are interpreted as the ensuing regressive phase associated to the MIS 19 high-stand beginning approximately at 780 ka. The transition between marine and continental sedimentation is gradual and substantially in stratigraphic continuity, implying a continuous and high-resolution recording of the MBT that is characterized by VGP swings between stable reverse (Matuyama) and stable normal (Brunhes) polarity. According to this interpretation, we attribute the thin normal magnetic polarity interval recorded in OH4-SU5 to the VGP variability of the MBT and exclude an attribution to the MB precursor dated to 794 ka^23^ as this geomagnetic event occurred during MIS 20 and may have been recorded at or below the base of the sampled section.

These results reveal a detailed recording of the MBT occurring throughout SU6 to SU3. In records of high sediment accumulation rate (>15 cm per thousand years), the MBT is characterized by brief VGP excursions occurring between stable reverse (Matuyama) and stable normal (Brunhes) polarity^23,24^, with a mid-point at 773 ka and a transition duration of around 8 or 10.8 thousand years^23,24^. Our sampling probably captured one such excursion in OH4-SU5 (Fig. 1b,d and Extended Data Fig. 4). The intertidal biocalcarenites of SU6 and the littoral sands of SU5 are interpreted as representing the marine isotope stage (MIS) 20–MIS19 transgression of sea-level (starting at around 795 ka)^25^ and the subsequent maximum flooding surface, respectively. The continental deposits of SU4–SU3 are interpreted as part of the ensuing regressive system tract associated with the MIS19 highstand (around 780 ka). This is consistent with a sedimentation rate of around 20 cm per thousand years, largely sufficient to capture the MBT variability. As in the Gran Dolina TD6 layer (Sierra de Atapuerca)^2^, our analysis indicates hominin ages younger than 990 ka (top of Jaramillo) and close to the MBT at a nominal age of 773 ± 4 ka (ref. ^23^) (Fig. 1d and Extended Data Fig. 4).

Biochronological data closely agree with the magnetostratigraphic ones (Supplementary Note 2). The fauna includes 37 species of mammals; it shares many species with that of Tighennif in Algeria, at least 1 million years old^26^. It documents the last known occurrences of the hare *Trischizolagus* and of the rhino *Ceratotherium mauritanicum*; *Theropithecus oswaldi* and *Kolpochoerus* are also indicative of an early age. Comparisons with other African sites are in good agreement with an age close to the EP–MP boundary^20,27^. Resemblances with East and South African faunas attest to easy latitudinal exchanges, demonstrating that the Sahara was not a permanent barrier in EP times owing to the recurrent expansion of savanna landscapes across North Africa in response to short-lived, astronomically driven periods of enhanced monsoon rainfall^28,29^.

Optically stimulated luminescence (OSL) dating, performed in unit SU4 on cemented sands provided age estimates of 420 ± 34 ka and 391 ± 32 ka (refs. ^17,19^), of the same order as the ages obtained from OH2 to OH5 (ref. ^30^). OSL ages appear to be inconsistent with the evidence that these formations belong to at least three glacioeustatic cycles^9,10^ and, for this reason, can be disputed. Combined electron-spin resonance (ESR) and U-series dating methods applied directly to an isolated hominin tooth from SU4 resulted in an estimated age of 501 ka +94 ka/−76 ka (refs. ^17–19,31^). The same method yielded ages ranging from 591 ± 103 ka to 538 ± 52 ka on three well-preserved herbivore teeth from SU4 (Extended Data Fig. 3a,b and Supplementary Note 4). However, the ThI-GH ESR samples have high uranium content in the dental tissues and particularly in enamel. In this case, the internal dose rate is probably too high to efficiently generate ESR signals in hydroxyapatite, leading to varying degrees of equivalent dose underestimation. Thus, combined ESR and U-series results obtained at ThI-GH are considered to be minimum ages (Supplementary Note 4).

## Hominin fossils

While the femoral shaft of ThI-GH-UA28-7 has already been analysed in detail^21^, most of the hominin fossils of the ThI-GH have not been described, including two partial mandibles, a large number of teeth and several vertebrae, which provide invaluable phylogenetic information.

### Mandibular morphology

**Extended Data Fig. 5:**
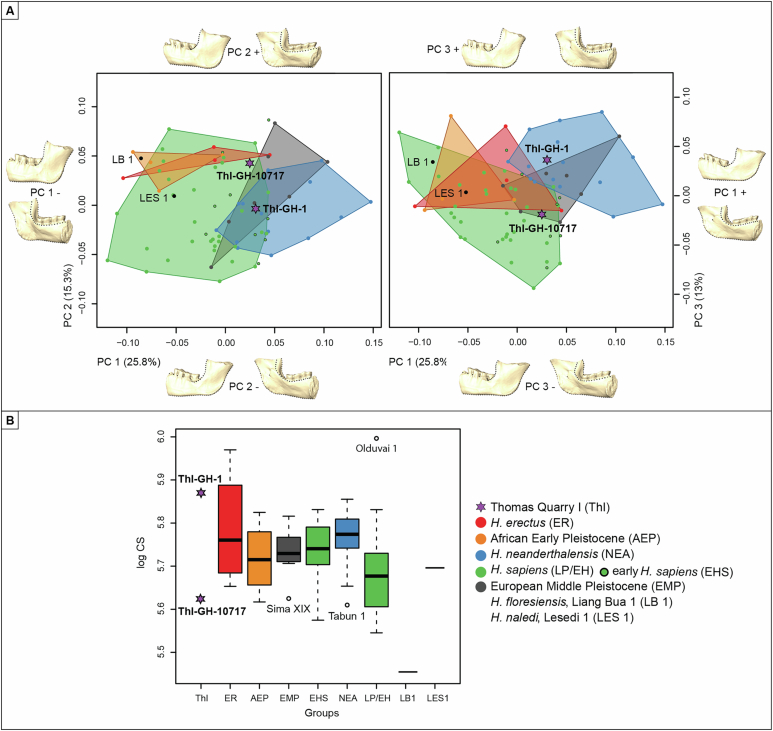
Mandibular shape and size. A) Principal Component Analysis Plots of the ThI-GH mandibular shape. Shape changes along PC 1 relate to the height and breadth of the corpus, PC 2 (left) the breadth of the ramus and the gonial profile, and PC 3 (right) the breadth of the ramus and shape of the anterior ramus. Both ThI-GH mandibles have a corpus height and breadth intermediate between *H. sapiens* and Neanderthals; whereas ThI-GH-10717 has a wider ramus and more everted gonial profile ploting with *H. sapiens* variation and near to the *H. erectus* hull and ThI-GH-1 has a taller and narrower ramus ploting within Neanderthal variation. (B) Box plot of mandibular size, measured as log centroid size (CS); ER n = 4, AEP n = 4, EMP n = 7, EHS n = 9, NEA n = 15, LP/EH n = 32. ThI-GH-10717 is considerably smaller than Th1-GH-1, and most fossils in the sample. Horizontal lines represent the median of each group. Boxes show the interquartile range (IQR, 25th to 75th percentile). Whiskers extend to 1.5 times IQR. Outliers are represented by circles.

ThI-GH-10717 is a gracile and nearly complete adult mandible preserving a full (although worn) dentition (Fig. 2, Extended Data Fig. 5 and Supplementary Note 6). Its corpus is long, low and narrow, with a slight pre-angular notch. In the lateral view, its symphysis is receding—an orientation similar to *Homo erectus* sensu lato. There is a small mentum osseum, conforming to category 2 (ref. ^32^). Its superior portion forms a faint incurvatio mandibulae. This morphology is also present in *Homo* sp. ATE9-1 from Sima del Elefante, as well as several early *Homo* individuals^33^ (such as Olduvai, Dmanisi, Malawi, Koobi Fora and Sangiran 9). Like ATE9-1, ThI-GH-10717 displays an archaic marked submental incisura. The anterior marginal tubercle is weak and located below the fourth mandibular premolar (P_4_), in a similar position to that observed on the EP mandibles from Tighennif, Algeria. The internal morphology of the symphysis is relatively smooth in its topography. It lacks both a superior and inferior transverse torus and expresses a shallow genioglossal fossa. The planum alveolare is nearly vertically oriented and has slight alveolar prominence. These features are similar in *H. antecessor* and considered derived relative to *H. erectus*^33^. The mental foramen is located below P_4_, intermediate between the archaic position at P_3–4_, found in *Homo habilis*, *Homo ergaster*, *H. erectus* and *H. antecessor* and the mandibles from Tighennif, and the derived position below the first mandibular molar (M_1_), which is found in some MP hominins and Neanderthals^34^. The lateral prominence of the corpus is weak, with the maximum expression at the level of M_2_. The M_3_ is partially covered by the ramus in lateral view. In contrast to the common condition in EP and MP hominins^35^, the masseteric fossa is shallow as in Neanderthals and *H. sapiens*^36^. The flat pterygoid fossa and the symmetrical mandibular notch are reminiscent of *H. sapiens*^37^ and diverge from the Neanderthal pattern^36–38^.

ThI-GH-1 is a more robust, but less complete, left adult hemimandible missing both the coronoid process and the mandibular condyle and preserving P_4_–M_3_ in situ. Like ThI-GH-10717, the corpus is low but with a more pronounced pre-angular notch that is also found in some European MP hominins^33,39^. It differs from ThI-GH-10717 in having a more pronounced and posterior lateral prominence at the level of M_2_–M_3_, an M_3_ that is not covered by the ramus in lateral view and an intermediate (between parallel and oblique) trajectory of the mylohyoid line in relation to the alveolar margin. These three features align it with some European MP hominins and Neanderthals^34,37,40,41^. It also has a deeper relief of the masseteric fossa than ThI-GH-10717, a frequent condition in EP and MP hominins^35^. However, like ThI-GH-10717, Tighennif and TD6 hominins, and unlike the archaic condition found in *H. erectus*^42^, the internal corpus shows moderate hollowing of the subalveolar fossa.

In three-dimensional (3D) landmark-based geometric morphometric analysis, the size of ThI-GH-10717 is modest, with a centroid size at the low end of the *H. erectus* sensu lato range. In shape analyses, it plots within the *H. erectus* sensu lato range of variation, along with African EP and MP hominins. Its shape differs from both Neanderthals and *H. sapiens* by having a broad ramus, narrow mandibular breadth, long and low corpus, and receding symphysis. Compared with all three groups, it has a smaller coronoid process, a more-expanded gonial profile and a lower anterior corpus. ThI-GH-1 is considerably larger than ThI-GH-10717, and falls within the *H. erectus* range. It also plots closer than ThI-GH-10717 to the European MP and Neanderthal range of variation in shape space.

### Dental morphology

**Extended Data Fig. 6:**
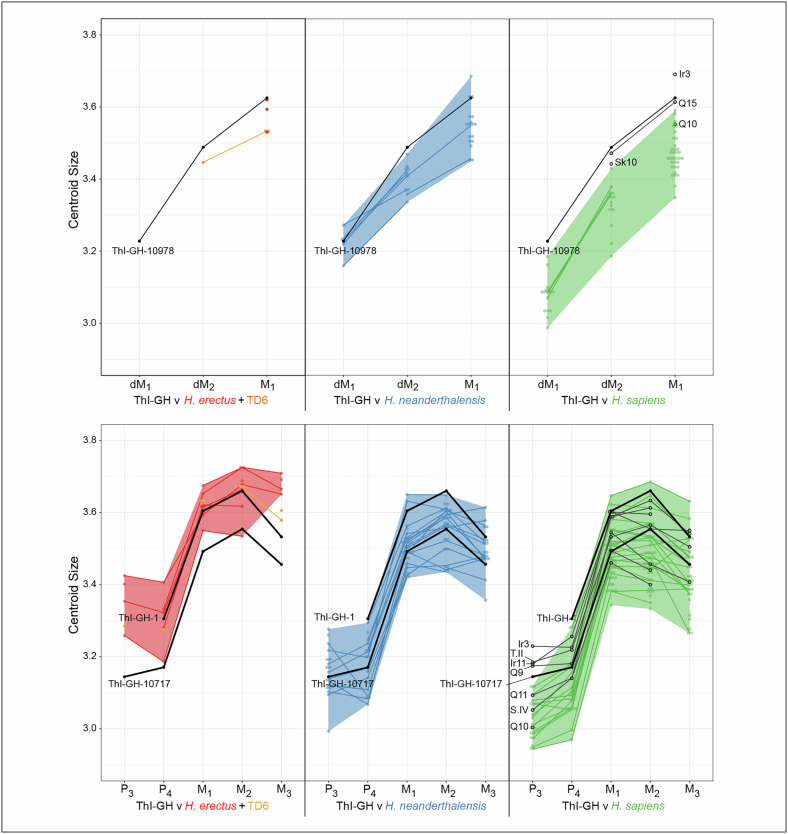
Tooth size comparisons. Plots of tooth size measured as centroid size comparing the three ThI-GH specimens (black solid lines) to *H. erectus/H. antecessor* (left), *H. neanderthalensis* (middle), and *H. sapiens*(right). The top row compares the first deciduous molar, second deciduous molar, and first permanent molar, and the bottom row the third mandibular premolar to the third mandibular molar. Shaded areas represent the range for *H. erectus* (red), *H. neanderthalensis* (blue) and *H. sapiens* (green). Fossil *H. sapiens* are marked separately and individually identfied by accession number. Continuous lines connect the measurements corresponding to different teeth of the same individual. The teeth of the ThI-GH-10978 immature mandible are larger than those of each comparative sample, whereas the permanent postcanine dentition of adult individuals displays smaller third premolars and third molars than *H. erectus*, and shows a similar pattern of relative tooth size to *H. neanderthalensis* and *H. sapiens*.

The fossils of ThI-GH include a sizable series of well-preserved permanent and deciduous teeth (comprehensive descriptions are provided in Supplementary Note 8 and Supplementary Tables 20–22). The postcanine teeth of ThI-GH-1 are consistently larger than the corresponding teeth of ThI-GH-10717. In both individuals, the molar size pattern is M1 < M2 > M3 (Extended Data Fig. 6), with a strong reduction in M3 contrasting with the conditions usually observed in *H. erectus*. Molar size patterns are variable, especially in *H. sapiens*^43^; however, this pattern is more common in *H. antecessor*, *H. sapiens* and Neanderthals. The crown outlines are similar to those of other EP hominins but, for the deciduous molars, they are closer to those of early *H. sapiens* than to those of *H. antecessor* (Supplementary Note 9).

**Fig. 3:**
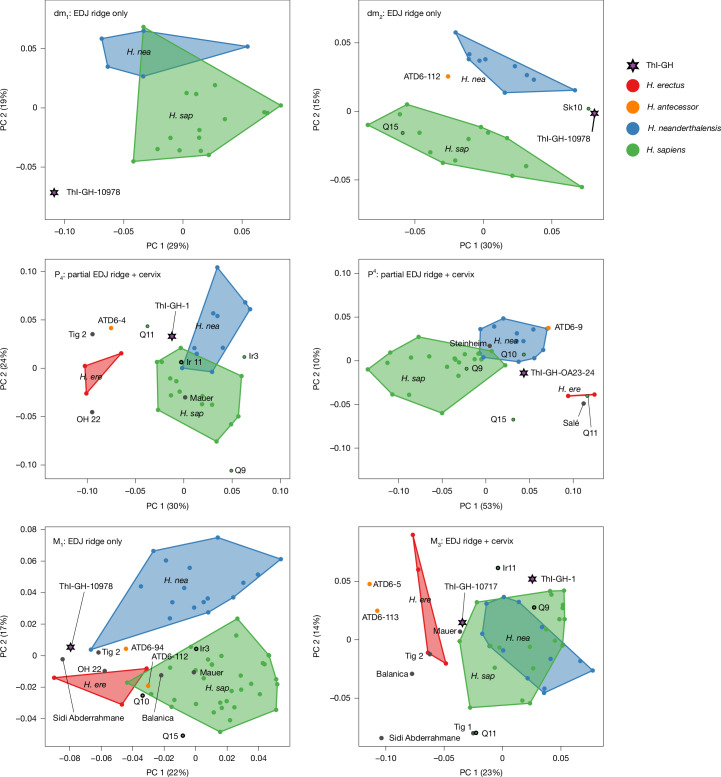
EDJ morphology of teeth. Principal component (PC) analyses of EDJ shape variation for the first (top left) and second (top right) deciduous molars, the mandibular fourth premolar (middle left), the maxillary fourth premolar (middle right), the first mandibular molar (bottom left) and the third mandibular molar (bottom right). Shape is captured using 3D landmarks on the primary cusps and marginal ridge of the EDJ and, in the case of the the fourth premolars and third mandibular molar, also the cervix (partial EDJ ridge means that only the unworn portion of the marginal ridge was analysed). For each tooth position, the ThI-GH teeth fall outside and adjacent to our samples of Neanderthals and *H. sapiens*. Fossil *H. sapiens* (*H. sap*), MP hominins and *H. antecessor* specimens are identified individually by accession number. *H. erectus* is identified by *H. ere *and *H. neanderthalensi*s by *H. nea*.

**Extended Data Fig. 7:**
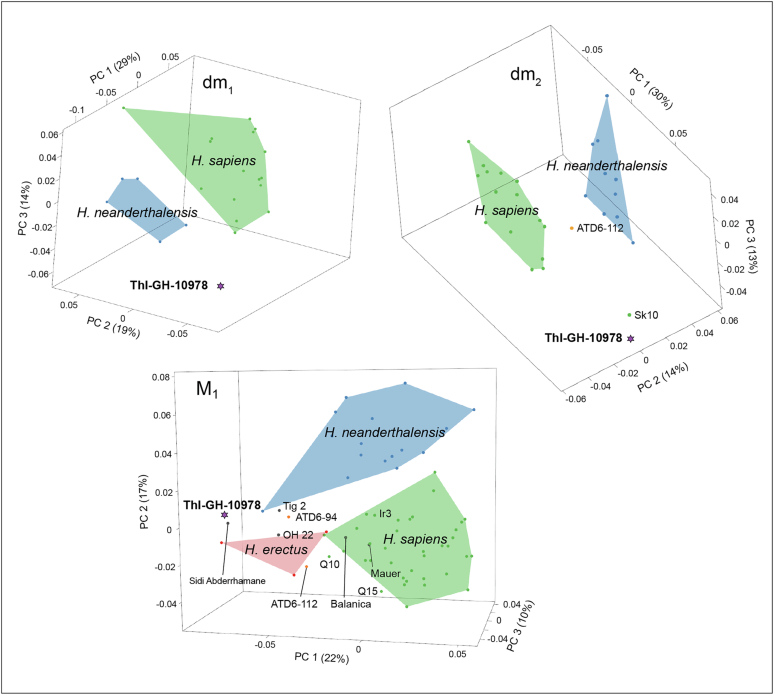
Molar morphology of ThI-GH-10978. Principal component plots of enamel-dentine junction (EDJ) shape of the first deciduous molar (top left), second deciduous molar (top right), and first permanent molar (bottom) from the ThI-GH-10978 mandible. The EDJ shape of all molars is different, and approximately equidistant from *H. neanderthalensis* and modern *H. sapiens*. Note that the *H. antecessor* second deciduous and first permanent molar are more derived in shape and sit closer to these two taxa.

**Extended Data Fig. 8:**
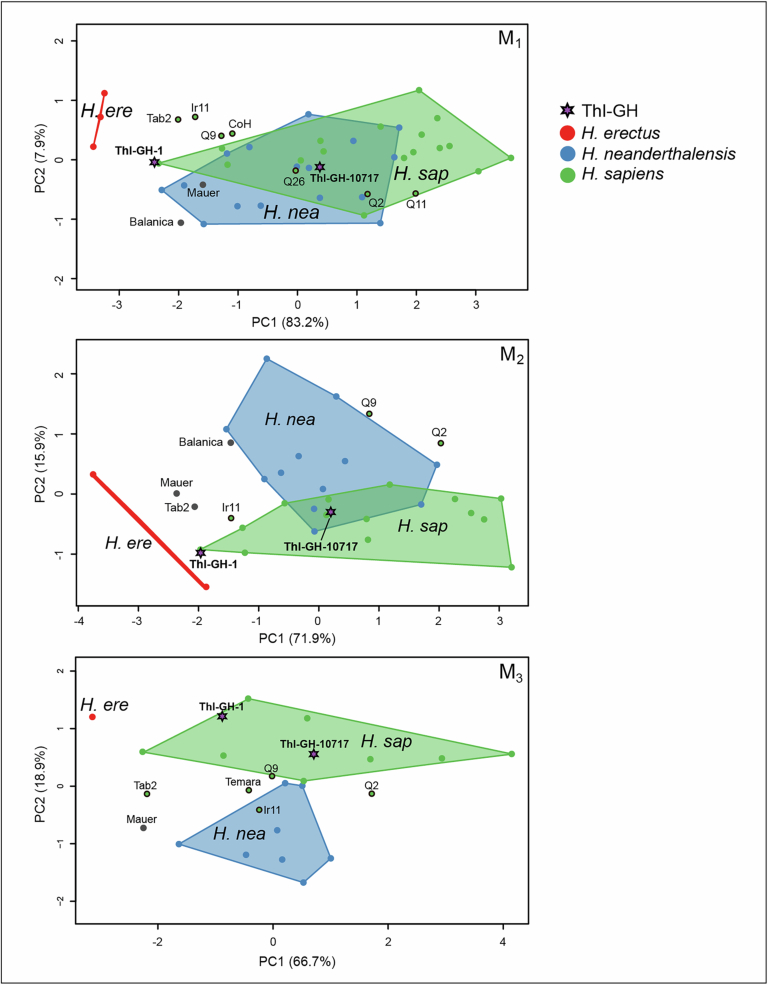
Mandibular molar root size and shape. Principal component analyses of four molar root variables: root length, square root of cervix plane area, cube root of root cervix volume, and cube root of root branch volume. There is decreasing overlap between *H. sapiens* and *H. neanderthalensis* from the first to the third mandibular molar. ThI-GH-10717 falls within the variation of both taxa for the first and second molar, but with *H. sapiens* for the third molar. Thl-GH-1 falls on the edge of the *H. sapiens* distribution for each molar position and towards the *H. erectus* samples.

The shape of the enamel–dentine junction (EDJ) can be studied on four permanent and two deciduous post-canine tooth positions using 3D landmark-based geometric morphometrics (Fig. 3 and Extended Data Fig. 7). In the deciduous dentition, the EDJ ridge shape of the ThI-GH-10978 first lower deciduous molar (dm_1_) falls outside *H. sapiens* and Neanderthals. This is also the case for the ThI-GH-10978 dm_2_, which also falls close to the fossil *H. sapiens* specimen Skhul 10. The *H. antecessor* TD6-112 dm_2_ is more similar in EDJ ridge shape to *H. sapiens* and Neanderthals, falling broadly between these two species. The P_4_ EDJ ridge and cervix shape of ThI-GH-1 place it close to the *H. sapiens* and Neanderthal samples, while the same features of ATD6-4 *H. antecessor* place it closer to *H. erectus*. The P^4^ EDJ ridge and cervix shape of ThI-GH-OA23-24 place it close to *H. sapiens* and Neanderthals, while ATD6-9 is close to Neanderthals. The EDJ ridge shape of the ThI-GH-10978 M_1_ (the cervix is not preserved) falls close to Sidi Abderrahmane, *H. erectus*, and relatively close to OH 22 and *H. antecessor* specimens ATD6-94 and ATD6-112. Like the dm_2_, *H. antecessor* M_1_ specimens ATD6-94 and ATD6-112 are more similar in shape to Neanderthals and *H. sapiens*. Finally, the EDJ ridge and cervix shape of the ThI-GH-10717 M_3_ fall just outside the *H. sapiens* sample and adjacent to the *H. erectus* sample, while the ThI-GH-1 M_3_ falls close to both *H. sapiens* and Neanderthal samples. In the molar roots, there is a decreasing overlap between *H. sapiens* and Neanderthals from M_1_ to M_3_, with *H. erectus* clustering on its own (Extended Data Fig. 8). The ThI-GH-1 M_1_ roots fall on the margin of recent *H. sapiens* and close to early *H. sapiens* from North Africa. The M_1_ roots of ThI-GH-10717 are more derived in their small size, towards recent *H. sapiens*. ThI-GH-1 M_2_ and M_3_ roots fall close to early *H. sapiens* specimens and near *H. erectus*, but ThI-GH-10717 M_2_ and M_3_ roots are well within the recent *H. sapiens* cluster.

The anterior dentition of ThI-GH-10717 is heavily worn (right canine) or broken (left canine and all incisors), but the roots are preserved although fragmented. The right canine is gracile (crown and root), similar to modern humans, and much smaller than in other EP and MP hominins such as Tighennif, Irhoud and Neanderthal individuals (Supplementary Note 10). The incisor ThI-GH-SA26-88 has a relatively small crown that is within the early and recent *H. sapiens* variations. By contrast, its root length falls in the upper end of the *H. sapiens* variation, in the lower range of Neanderthals, and is smaller than that of *H. erectus* (KNM-WT 15000).

From a non-metrical dental trait perspective (Supplementary Table 20), the mandibular molars from ThI-GH are comparable to other EP and MP teeth from North Africa (for example, Sidi Abderrahmane and Tighennif) and are similar to the TD6 *H. antecessor* molars in their variable expression of trigonid crests, cusp 7 and lack of cusp 6. However, in both deciduous and permanent postcanine teeth, the cusps of the TD6 specimens are more closely spaced than they are in the North African specimens. In this way, the TD6 *H. antecessor* specimens appear more derived towards Neanderthals. Moreover, ThI-GH-1 M_2_ and M_3_ (as well as Tighennif 1) differ from the TD6 material in the way they taper distally, which is an archaic feature seen in African *H. erectus*. Furthermore, neither the crown of the Tighennif nor the ThI-GH teeth show any lingual relief, especially shovel-shaped morphology for incisors.

### Vertebrae

**Extended Data Fig. 9:**
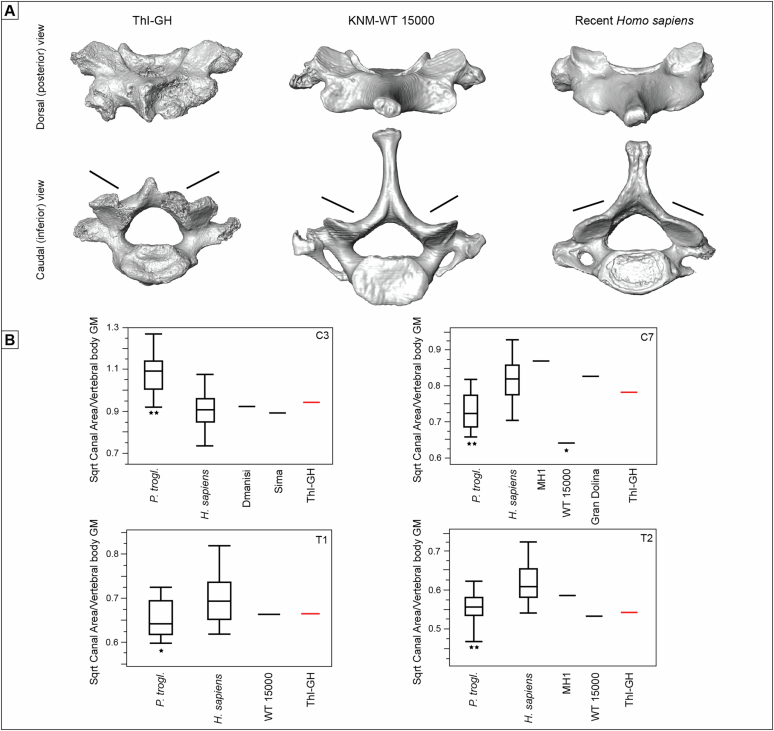
Vertebral morphology. A) Articular pillar morphology and lower facet orientation. Comparison in dorsal and caudal views of the seventh cervical vertebra from ThI-GH-10717/3 compared to KNM-WT 15000 and to a recent *Homo sapiens* (figures not to scale). The arrow indicates the presence of a marked notch in the articular pillars between the upper and the lower articular facets. The black lines indicate the approximate orientation of the lower articular facets, which are slightly convex and display a ventral-lateral orientation in ThI-GH and KNM-WT 15000, while they are flatter with a ventral to ventral-medial orientation in recent *Homo sapiens*. B) Box plots of vertebral canal area relative to vertebral body geometric mean (following and modified from^134^). All the Casablanca specimens fall close to the *H. sapiens* mean, except for the T2, where both KNM-WT 15000 and ThI-GH specimens show very low value. In the boxplots, the whiskers represent the 1.5 times the interquartile range, the box represents the interquartile range, and the centre represents the median. The *Pan troglodytes* sample was compared to the *Homo sapiens* sample using a two-tailed Student’s T-tests. The different fossil specimens (Dmanisi, Sima de los Huesos, ThI-GH, KNM WT-15000 and Gran Dolina-TD6 and MH1) were compared to the *Homo sapiens* sample using a z-score analysis. * = significantly different from *Homo sapiens* (*p* < 0.05); ** = significantly different from *Homo sapiens* (*p* < 0.01). Comparative sample sizes: *Pan troglodytes* (C3: n = 18; C7: n = 13; T1: n = 10; T2: n = 19); *Homo sapiens* (C3: n = 62; C7: n = 37; T1: n = 25; T2: n = 52)^134^.

Directly underneath ThI-GH-10717, a series of eight vertebrae (six cervical and two thoracic) was discovered (Fig. 2 and Extended Data Fig. 9). Their small size and very close spatial proximity to the mandible suggest that they belonged to the same small-bodied adult. Although the fossil record allows limited comparisons, morphologically, the most complete vertebrae (C7, T1 and T2) are more similar to *H. erectus* than to recent *Homo* species. In particular, C7 displays a more lateral (ventral–lateral) orientation of the lower articular facets relative to the condition observed in *H. sapiens* and Neanderthals. The immature *H. antecessor* C7 (ATD6-75) is more *H. sapiens*-like in the orientation of the lower articular facets^44^. Moreover, the orientation of the transverse processes in the ThI-GH both T1 and T2 vertebrae is slightly more dorsal than in recent *H. sapiens*, while it is notably more dorsal in KNM-WT 15000 (Supplementary Table 23). The vertebral canal section area in the ThI-GH specimens is similar to the Dmanisi C3 and to the *H. antecessor* C7 vertebrae, and similar (T1) or larger than those of KNM-WT 15000 (C7, T2). These areas are below the mean but not significantly different from a recent *H. sapiens* sample (Supplementary Table 24). When standardized relative to the geometric mean of vertebral body linear dimensions, all of the values fall within the *H. sapiens* variation, except for the T2, where both KNM-WT 15000 and ThI-GH specimens show very low values (Extended Data Fig. 9).

## Discussion and conclusions

In North Africa, the ThI-GH hominins are the only specimens unearthed within an indisputable stratigraphic context and securely dated to the MBT at a nominal age of 773 ± 4 ka. These hominins cannot be directly compared with later specimens, such as the Kabwe or Bodo skulls, which have been tentatively assigned to *H. heidelbergensis*. Not only do these specimens differ substantially in age, but they also lack preservation of comparable anatomical parts. Our analysis suggests that the ThI-GH hominins probably belong to an evolved form of *H. erectus* sensu lato in North Africa, much as *H. antecessor* does in Europe. However, the ThI-GH hominins offer an interesting contrast to both the Spanish fossils and the considerably older fossils from Tighennif (Algeria), which are likely to date to at least 1,000 ka (refs. ^45–48^). The fossil mandibles from Tighennif appear more primitive, larger and more robust than both the European *H. antecessor* and the northwest African ThI-GH fossils. The Spanish and Moroccan fossils share several features in their teeth and mandibles. Both groups display a combination of archaic and derived features reminiscent of later hominins (Supplementary Table 25). These similarities revive the question of possible exchanges across the Strait of Gibraltar during the EP. Nevertheless, the ThI-GH hominins are different from the TD6 hominins. The pattern of these differences suggests that regional differentiation between Europe and North Africa was already present by the late EP. Apparent Neanderthal-like features on the larger ThI-GH-1 mandible could reflect primitive retentions, allometric effects or convergent evolution but, when more phylogenetically informative dental characters are considered, the Spanish specimens appear more derived towards the Neanderthal morphology that later emerged in western Eurasia (see also refs. ^49,50^).

The origin of *H. sapiens*, and the precise timing of the divergence of its ancestral populations from the Neanderthal–Denisovan clade, remain subjects of debate. Anatomical evidence has at times been used to argue for a split predating 800 ka (ref. ^51^) and even for an alternative Asian ancestry of our species^52^. In this context, the Maghreb fossils are key to understanding the diversification of MP hominins. The morphology of the ThI-GH hominins places them close to the split between the African and Eurasian lineages. Our findings not only align with the phylogenetic structure inferred from palaeogenetic data but also highlight the Maghreb as a pivotal region for understanding the emergence of our species, reinforcing the case for an African rather than a Eurasian ancestry of *H. sapiens*.

## Methods

### Excavation methods

ThI-GH SU4 and SU5 have been systematically excavated since 1994. A 0.5/1-m-deep sequence of an area of 48 m^2^ was excavated (Fig. 3a and Extended Data Fig. 1b). Excavation was performed according to the stratigraphic sediment deposition, and stratigraphic units were subsequently numbered from 1 to 7 from top to bottom. We established an arbitrary excavation 1 m × 1 m grid, and spatial data (*x*, *y*, *z*) of all finds (worked and unworked lithics, as well as faunal and human remains) were recorded (Extended Data Table 1). From 1994 to 2005, single finds were assigned unique IDs consisting of the quarry acronym (ThI), the site acronym (GH), the name of the square and a progressive number (for example, ThI-GH-SA26-88). From 2006 onwards, spatial data measurement was carried out with the total station. The code for each find consists of the quarry acronym, the site acronym and a number from 10000 (for example, ThI-GH-10634). We documented layers, special features and profiles in 3D models using total station measurements, digital photographs and drawings. The 3D models were referenced with control points recorded with the total station to align them to the excavation grid. Sediments have been collected for every m^2^, dissociated with diluted formic acid and wet-screened to recover lithic and faunal small fragments.

### Stratigraphy of the OHF

The chronostratigraphic framework of the OHF exposed at ThI is based on the direct observations of sedimentary formations, stratigraphic boundaries and facies. The successions and associations of facies have been used to characterize the depositional environments, their evolution and to infer sea level changes. According to the sequence stratigraphy concept, an allostratigraphic unit is defined by a sedimentary sequence characterized by a succession of deposits attributed to intertidal, supratidal and aeolian/continental environments, bounded at its base and top by unconformities. This sequence is essentially deposited during phases of marine transgression and sea-level high stands. According to the international stratigraphic guide, the allostratigraphic units were formalized as members of the OHF. Microfacies analysis was carried out on large thin sections prepared from blocks of oriented sediments vacuum-impregnated with polyester resin. These analyses provided specific information about diagenetic processes occurring during and after deposition.

### Sedimentology of ThI-GH infilling

Stratigraphic units SU5 to SU3 were studied using a geoarchaeological approach, integrating field observations (sedimentary structure, colour, discontinuities and so on), micromorphology and analyses (particle size distribution, magnetic susceptibility measurements and energy-dispersive X-ray fluorescence (ED-XRF) analyses). Micromorphology was based on the observation of large thin sections taken continuously in stratigraphic order. Particle size analyses were performed on bulk samples after decarbonatation. Volume magnetic susceptibility was measured along the section using a Bartington MS2K sensor, with a vertical resolution of 2 cm. Air-dried and crushed bulk samples (<2 mm) were analysed by ED-XRF using a calibrated portable spectrometer (SPECTRO X-SORT) (Supplementary Note 1).

### Magnetostratigraphy and rock magnetic properties

Magnetostratigraphic data were obtained from a population of 119 oriented core-samples retrieved from members OH3, OH4 and GHCCC in 2022 and 2023 and integrated with 62 samples previously analysed in 2018–2019^13^ from the same members plus member OH5. The sampling of ThI-GH infilling (from SU6 to SU3) was conducted along 5 sections, A and B (68 samples), C (14 samples), D (6 samples) and composite section E (19 samples) (Fig. 1d and Extended Data Fig. 2b), yielding a total of 107 oriented core-samples taken to better anchor the hominin bearing site ThI-GH to the MBT. Furthermore, 13 samples were retrieved from member OH3 to refine the record of the Jaramillo subchron previously observed^13^.

Magnetostratigraphic samples were thermally demagnetized from room temperature up to a maximum of 690 °C with a TD48 ASC furnace. Alternating-field (AF) demagnetization up to 200 mT performed on two test samples with an LDA5 AF demagnetizer resulted inadequate to resolve the magnetic remanence of the samples. After each thermal demagnetization step, the initial magnetic susceptibility was measured using a Bartington susceptibility bridge. The natural remanent magnetization was measured on a 2G DC-SQUID cryogenic magnetometer located in a magnetically shielded room. Standard least-squares analysis was used to calculate ChRM component directions from vector end-point demagnetization diagrams, from which VGP latitudes were derived (positive VGP values for normal polarity, negative values for reverse polarity). Great circles were used to assess qualitatively the ChRM orientation in absence of stable end points. The magnetic mineralogy was investigated using hysteresis experiments from −1.5 T to +1.5 T, low-resolution first-order reversal curves (FORCs, *n* = 76) interpreted with FORCinel^53^, stepwise acquisition of an isothermal remanent magnetization (IRM) up to 1.5 T, AF decay of a 1 T IRM in AF peak fields from 50 mT to 1.5 T and thermomagnetic decay of a 1 T magnetization performed in Ar atmosphere from room temperature to 680 °C. These experiments were performed using a MicroSense EZ7 Vibrating Sample Magnetometer with heating ability. Additional samples were also subjected to thermal demagnetization of a three-component IRM using orthogonal fields of 1.5 T, 0.4 T and 0.12 T imparted with an ASC pulse magnetizer. Details are provided in the main text and Supplementary Note 5.

### Geometric morphometric analysis of ThI-GH-1 and ThI-GH-10717 mandibles

The fossil sample (Supplementary Table 17) comprises Early, Middle and Late Pleistocene hominins from Africa, Europe and Asia, including specimens attributed to *H. erectus*, *Homo floresiensis*, *Homo naledi*, *H. neanderthalensis*, *H. sapiens* and Denisovan (Xiahe mandible). As the taxonomy of the European MP hominins is contested, we have refrained from assigning specimens from this period to a taxon but refer to them as European MP hominins. Moreover, there are several EP fossils from Africa with ambiguous taxonomic attribution (that is, from Baringo Kapthurin, Kenya and Tighennif, Algeria) that we refer to as African EP hominins. We used the term early *H. sapiens* to refer to the oldest members of our species from around 300 to 100 ka found at sites in Africa and the Near East (such as Jebel Irhoud, Klasies River Mouth, Border Cave, Skhul and Qafzeh). All of the specimens are adults based on dental eruption and spheno-occipital fusion, except for KNM-WT 15000.

Micro-computed tomography (micro-CT) scans of ThI-GH-1 and ThI-GH-10717 were made with Diondo d3 at the Department of Anthropology, Max Planck Institute for Evolutionary Anthropology, Leipzig, Germany, with a scan resolution of 30 µm. 3D surface models were reconstructed from these CT scans using Avizo v.7.1 (Thermo Fisher Scientific). 3D surface models of the comparative sample were created from either CT scans using Avizo v.7.1 or photogrammetry. For the latter, between 40 and 90 2D photographs were taken using the Nikon D600 (4,512 × 3,008 pixels) and processed with Agisoft PhotoScan Professional v.1.2.0 (Agisoft)^53^. Error tests evaluating differences in imaging techniques are within the acceptable range of error in osteometry^54^. For most fossils, surface models were generated from the original specimen; however, when surface models from the original specimen were not available research quality casts were used^54–58^.

Minor virtual reconstruction was needed for most specimens in the comparative sample and was performed in either Geomagic Studio 2014 v.3.0 (3D Systems) or Avizo v.7.1. The type of reconstruction varied considerably depending on the specimen, but generally included the filling of cracks or holes, removal of sediments, smoothing of abraded areas and refitting of fragments. For some fossils in which one side was missing or deformed, bilateral symmetry was exploited by mirror-imaging. Specific details regarding the reconstruction techniques and error tests have been published previously^54–59^.

Geometric morphometric methods were used to analyse the shape and size of the ThI-GH fossils in a comparative context. Separate landmark datasets (Supplementary Figs. 32 and 33) were created according to the preserved anatomical elements of the ThI-GH mandibles: (1) a mandibular dataset, consisting of 301 (semi)landmarks, based on the preserved morphology of ThI-GH-10717; (2) a left mandibular dataset consisting of 87 (semi)landmarks, based on the preserved morphology of ThI-GH-1; and (3) and an anterior corpus dataset, consisting of 153 (semi)landmarks, which allowed for an expanded comparative sample. Three-dimensional coordinates of anatomical landmarks and curve semilandmarks were digitized on the surface models using Landmark Editor (v.3.0.0.6)^60^. Landmark and semilandmark data were processed and analysed previously^61^ using the packages Morpho (v.2.9)^62^ and geomorph (v.4.0.2)^63,64^. For each dataset, missing bilateral landmarks and semilandmarks were estimated by mirroring the preserved side. Missing landmarks and semilandmarks lacking a bilateral counterpart were estimated by deforming the sample average onto the deficient configuration using thin-plate spline interpolation^56–58,65^. Curve and surface semilandmarks were slid by minimizing the bending energy of a thin-plate spline deformation between each specimen and the sample mean shape^66,67^. After sliding, all landmarks and semilandmarks datasets were symmetrized and converted to shape variables using a generalized Procrustes analysis^68^.

For each dataset, the Procrustes coordinates were analysed using principal component analyses (PCA) in shape space, and nearest neighbours were calculated according to interindividual Procrustes distances. The ThI-GH fossils were projected into this PCA space. Shape changes were visualized along PC 1 and PC 2 by warping the sample mean shape along the positive and negative ends of PC 1 and PC 2, ±2 s.d. from the sample mean. To evaluate the size of the ThI-GH mandibles, the natural logarithm of centroid size was calculated for each specimen and compared across groups.

Mandibular metric data are shown in Supplementary Note 6 and Supplementary Tables 7 and 8. Linear measurements were taken by I.B. on 3D surface models generated from micro-CT scans in Avizo and were complemented by measurements of the original specimens taken by E. Trinkaus and by comparative data taken from the literature^4,14,34,69–116^. The *H. antecessor* data include ATD6-96, ATD6-5, ATD6-113; the *H. habilis* data include KNM-ER 1501, KNM-ER 1502, KNM-ER 1805, OH 7, OH 13, OH 37; the *H. ergaster* data include KNM-ER 730, KNM-ER 992A, OH 22, OH 23, and OH 51; the *H. erectus* data include Zhoukoudian Lower Cave (G1.6, G1/G2, H/1), Lantian, Sangiran (1b, 5, 9); the African EP sample includes KNM-BK 67, KNM-BK 8518, Sidi Abderrahmane 2, Tighennif (1, 2, 3); the European MP sample includes Arago (II, XIII), Mauer, Montmaurin 1, Sima de los Huesos (XIX, XXI, XXVIII), AT-1, AT-75, AT-300, AT-605, AT-607; the Neanderthal sample includes Amud 1, Arcy II, Banyoles, Chagyrskaya 6, El Sidrón (1, 2, 3), Guattari (2, 3), Hortus 4, Kebara 2, Krapina (57, 58, 59), Suard S 36, Bourgeois Delaunay 1, La Ferrassie 1, La Quina H5, La Naulette 1, Le Regourdou 1, Saint-Césaire 1, Shanidar (1, 2, 4), Sima de las Palomas (1, 6, 23, 59), Spy (1, 3), Subalyuk 1, Tabun C1, Vindija (206, 226, 231, 250, 11.39, 11.40, 11.45), Weimar-Ehringsdorf F1009 and Zafarraya; the early *H. sapiens* sample includes Contrebandiers 1, Dar es-Soltan II-H5, Dire Dawa, El Harhoura 1, Jebel Irhoud 11, Klasies River (KRM 13400, KRM 14695, KRM 16424, KRM 21776, KRM 41815), Qafzeh (9, 25), Skhul (IV, V) and Tabun C2. The Upper Palaeolithic and Epipalaeolithic sample includes individuals from Abri Pataud 1, Arene Candide (2, 18), Asselar, Barma del Caviglione, Chancelade, Cro Magnon (1, 3), Dar es-Soltan (II-H2, II-H3), Dolni Věstonice (3, 13, 14, 15, 16), El Mirón, Grotte des Enfants 4, Hayonim (8, 17, 19, 20, 25, 27, 29 and 29a), Isturitz (106 and 115), Le Roc (1, 2), Minat 1, Moh Khiew, Muierii 1, Nahal Oren (6, 8, 14, 18), Nazlet Khater 2, Oase 1, Oberkassel (1, 2), Ohalo II (1, 2), Pavlov 1, Předmostí (3, 21), Sunghir (1, 6), Villabruna 1 and Zhoukoudian Upper Cave (101, 104, 108).

### 3D EDJ shape analysis

The shape of the EDJ was examined for multiple tooth positions represented in the ThI-GH sample and compared to a sample of fossil hominins as well as early and recent modern humans. Details of the comparative sample are listed in Supplementary Table 22. TIFF stacks were filtered using only a mean of least variance filter (kernel size one), or a 3D median filter (kernel size of three) followed by a mean of least variance filter (kernel size of three) using MIA open source software^117^. Enamel and dentine tissues of each tooth were then segmented using the watershed module in Avizo 6.3 (Thermo Fisher Scientific). After segmentation, the EDJ was reconstructed as a triangle-based surface model. We then used Avizo 6.3 to digitize 3D landmarks and curve-semilandmarks on these EDJ surfaces. Anatomical landmarks were placed on the tip of the dentine horn of the protocone/protoconid and metacone/metaconid (premolars), as well as the entoconid and hypoconid (molars). A sequence of landmarks was also placed along the marginal ridge connecting the dentine horns beginning at the top of the protocone/protoconid moving in the lingual direction. In R^61^, a smooth curve was fit through this set using a cubic spline function, before dividing the curve into sections using the dentine horn landmarks (four sections for molars, two for premolars). A fixed number of equidistant semilandmarks were then placed along each section of the curve (landmark numbers reflect the relative length of these sections; in premolars the sections have 20 and 25 landmarks, respectively; and, in molars, they have 18, 15, 22 and 12 landmarks). Likewise, we digitized and resampled a curve along the cemento–enamel junction (cervix) as a closed curve starting either on the mid-face of the base of the protocone/protoconid (premolars) or on the mesiobuccal corner below the protoconid (molars). Homologous landmarks were then derived in R using the packages Morpho^62^ and princurve^118^ using a freely available R-based software routine^119^. Anatomical landmarks were fixed while the resampled points along the curves were treated as semilandmarks and allowed to slide along their curves so as to reduce the bending energy of the thin-plate spline interpolation function calculated between each specimen and the Procrustes average for the sample^66,67^. Sliding was performed twice, with landmarks projected back onto the curve after each step, before landmarks were considered geometrically homologous. Slid landmarks were then converted into shape coordinates using generalized least squares Procrustes superimposition, which removes scale, location and orientation information from the coordinates^68,120^. PCA was used to summarize shape variation in the comparative sample and assess morphological affinities of the ThI-GH teeth.

### Tooth size analysis

Tooth size was analysed for two sets of mandibular teeth: dm_1_/dm_2_/M_1_ and P_3_/P_4_/M_1_/M_2_/M_3_. The size of each tooth was represented by its centroid size, calculated using the cervix or EDJ landmark set used in the EDJ GM analysis (see above). Centroid size was calculated as the square root of the sum of squared distances of each landmark to the centroid of all landmarks. Associated teeth are represented in plots by lines between points of adjacent teeth. The sample used for the tooth size analysis is listed in Supplementary Table 22.

### Tooth wear

Wear categories were scored according to Molnar^121^.

### Tooth descriptions and measurements

The outer enamel surface (OES) and EDJ of the ThI-GH specimens were scored using a combination of visual inspection of the originals, photographs (OES) and virtual 3D models (OES and EDJ).

Descriptions of the pulp cavity configurations of the ThI-GH specimens were based on virtual 3D models. Measurements of the buccolingual and mesiodistal lengths of the ThI-GH specimens were taken from virtual 3D models. No corrections were made for interproximal wear and teeth that were too damaged to measure were omitted. With a few exceptions (for example, Atapuerca material) morphological assessment and crown measurements of the comparative material were taken from the original specimens using Mitutoyo digital callipers.

### Crown and root morphology

Scores for most non-metric traits were obtained using a combination of written descriptions and (where applicable) reference plaques of the Arizona State University Dental Anthropology System (ASUDAS^122^). Traits that are not part of the ASUDAS, or those that have been reassessed since its publication were scored using different methods. We scored shovelling for incisors and canine; labial convexity for incisors; Tomes’ root for lower third premolar, anterior fovea, deflecting wrinkle, fissure pattern, cusp 6; and protostylid and enamel extension for lower molars as described previously^122^. We scored lower molar cusp 7 variation according to ref. ^123^. Trigonid cresting patterns and variation on the lower molars were assessed as described previously^124^. Premolar asymmetry and transverse crest on the lower fourth premolar were assessed based on ref. ^125^. We referred to ref. ^126^ to make assessments of maxillary premolar accessory ridges or MxPAR on the upper premolars.

### Crown outline

The 3D digital models of the teeth were aligned with the cervical plane parallel to the *x*–*y* plane of the Cartesian coordinate system and rotated around the *z* axis with the lingual side parallel to the *x* axis. For the ThI-GH specimens, the right dm_1_ and dm_2_ were first mirrored in Geomagic Design X (3D Systems Software) to be compared with the left-side comparative sample. The crown outlines of the digital models were then extracted as a .igs file and imported into Rhinoceros v.5 (Robert McNeel & Associates). The centroid of the crown outlines was calculated based on the area of the outlines. A total of 24 equiangularly spaced radial vectors (with the first radius directed buccally and parallel to the *y* axis of the Cartesian coordinate system) from the centroid determined 24 pseudolandmarks of the crown outlines. The crown outlines were centred by their centroid and scaled to unit centroid size, transforming them into Procrustes shape coordinates, which were then used for computing PCA^127–129^.

ThI-GH specimens were projected in the shape-space PCA computed on Neanderthals and *H. sapiens* from the published comparative sample of dm_1_ and dm_2_^128,130,131^, updated with unpublished specimens (Supplementary Table 22). The differences in crown outlines among Neanderthals, *H. sapiens* (combined recent and Upper Palaeolithic) and early *H. sapiens* were investigated using permutation tests on the first three PCs (*n* = 10,000). A leave-one-out cross-validation quadratic discriminant analysis was performed, as the assumption of normality of variance was violated, on the minimum number of PCs accounting for 70% to 90% of the total variation^131,132^, to discriminate Neanderthal and *H. sapiens* groups and to estimate the taxonomic attribution of ThI-GH specimens to one of these groups with posterior probabilities (*P*_post_). Statistical analyses were performed using R (v.4.2.3)^61^.

### Anterior tooth root morphology

From the micro-CT scans, the tooth tissues of the anterior teeth were segmented (that is, enamel, dentine, pulp cavity). Linear, surface and volume variables were measured on the anterior tooth roots after the protocol described previously^133^. Comparative samples involve Neanderthals, early and recent *H. sapiens*, as well as early and lower Pleistocene hominins, some from Northern Africa. Details are provided in Supplementary Note 10.

### Age at death for the juvenile

Based on 2D virtual sections generated from the micro-CT scans, the calcification stage of the ThI-GH-10978 deciduous and forming permanent teeth was scored. These scores were compared with other juvenile fossil hominins (details are provided in Supplementary Note 7).

### Reporting summary

Further information on research design is available in the Nature Portfolio Reporting Summary linked to this article.

## Online content

Any methods, additional references, Nature Portfolio reporting summaries, source data, extended data, supplementary information, acknowledgements, peer review information; details of author contributions and competing interests; and statements of data and code availability are available at 10.1038/s41586-025-09914-y.

## Supplementary information

Supplementary Information Supplementary Notes 1–10, Supplementary Figs. 1–32, Supplementary Methods, Supplementary Tables S1–S25 and Supplementary References.

Reporting Summary

## Figures and Tables

**Extended Data Table 1:**
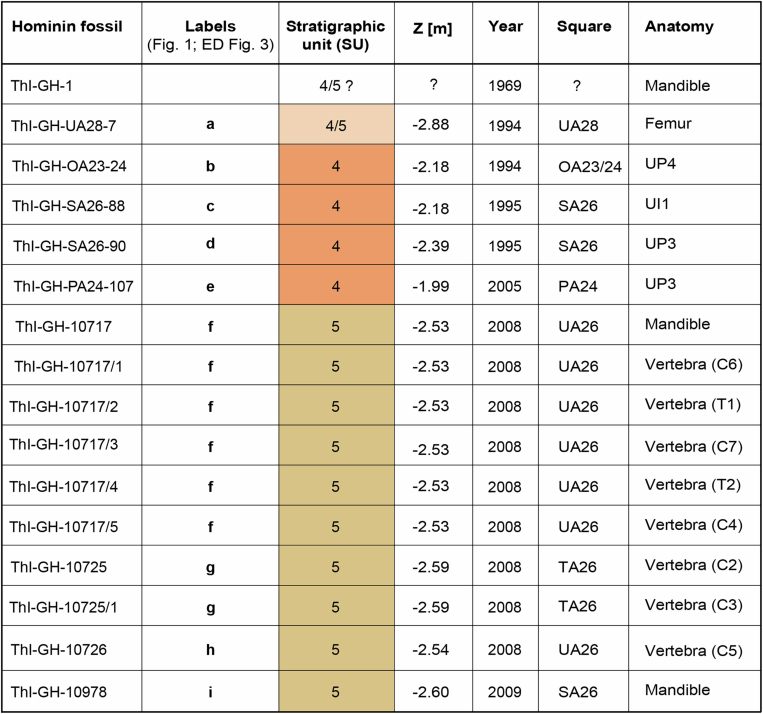
List of hominin fossils

Labels refer to Fig. 1 and Extended Data Fig. 3.

## Data Availability

All data supporting the findings of this study are provided in the Article and its Supplementary Information. Additional raw data (3D scans) are available from the corresponding authors on reasonable request.
