# Supplementary material for: Early hominins from Morocco basal to the Homo sapiens lineage
Source: Nature. 2026 Jan 7;649(8098):902–8. doi: 10.1038/s41586-025-09914-y (PMC12823423; doi:10.1038/s41586-025-09914-y)
Supplement: Supplementary file 1 — Supplementary Notes 1–10, Supplementary Figs. 1–32, Supplementary Methods, Supplementary Tables S1–S25 and Supplementary References. [file 41586_2025_9914_MOESM1_ESM.pdf]

nature portfolio
https://doi.org/10.1038/s41586-025-09914-y

Supplementary information

Early hominins from Morocco basal to the *Homo sapiens* lineage

In the format provided by the authors and unedited

Supplementary Information

Supplementary Notes:

Supplementary Note 1 - Geological and chronological setting……………………..………..……....………p. 2

Supplementary Note 2 - Fauna……………………………………………...……………….……;….p 11

Supplementary Note 3 - Lithic assemblage……………...…………………...……………..…………p. 18

Supplementary Note 4 - ESR Dating……………………………………...…………….…………p. 20

Supplementary Note 5 - Magnetostratigraphy……………………………..………….….......………p. 23

Supplementary Note 6 - Adult mandibular morphology………………...……..………..;….…………….p. 33

Supplementary Note 7 - Developmental stage of ThI-GH-10978…….……..……………………….....p. 42

Supplementary Note 8 - Dental morphology……………………...…………..………………………...p. 46

Supplementary Note 9 - ThI-GH-10978 Crown outline morphometry……...…….……………………p. 53

Supplementary Note 10 - Anterior dentition morphometrics…………………...………………………p. 56

Supplementary Method 1

Landmarks and curves semilandmarks used in the mandibles geometric morphometric analyses………. p. 62

Supplementary Tables:

Supplementary Table 16: Magnetic component data of the studied sample…………………………….…..p. 66

Supplementary Table 17: Comparative sample used in the Mandibular Study……….……….....…..…p. 72

Supplementary Table 18: Procrustes distances of ThI-GH-10717 Mandibular Shape…………..................p. 75

Supplementary Table 19: Procrustes distances of ThI-GH-10717 and Th1-GH-1 left mandible shape….…p. 77

Supplementary Table 20: Non-metric Dental Traits……………………………………………...…..p. 79

Supplementary Table 21: Dental Metrics…………………………………………………..……..……p. 81

Supplementary Table 22: Tooth Comparative Samples……………………………………...……p. 84

Supplementary Table 23: Vertebral Transverse process orientation……………………………..…….p. 100

Supplementary Table 24: Vertebral foramen cross-sectional area……………………………...……p. 101

Supplementary Table 25: Comparative overview of key anatomical features in the ThI-GH hominins ......p. 102

Supplementary References……………………...……………………………….;..……p. 104

Supplementary Note 1 - Geological and chronological setting

D. Lefèvre, J.-P. Raynal, M. Rué, G. Muttoni, S. Perini, M. El Graoui and A. Queffelec

The Casablanca hinterland shows a vast system of longitudinal dune ridges parallel to the modern coast, composed of marine and aeolian calcarenites. It records an exceptional succession of palaeoshorelines since the end of the Miocene thanks to tectonic uplift and sea-level variations.

The Pleistocene Casablanca sequence

In the Southwest of Casablanca, four Formations associated with four raised platforms and including several Members have been defined from the Early to the Late Pleistocene: the Oulad Hamida Formation (late Early Pleistocene), the Anfa and the Kef Haroun Formations (Middle Pleistocene), and the Dar Bou Azza Formation (Late Pleistocene) (**Fig. 1A, B**)[1-5].

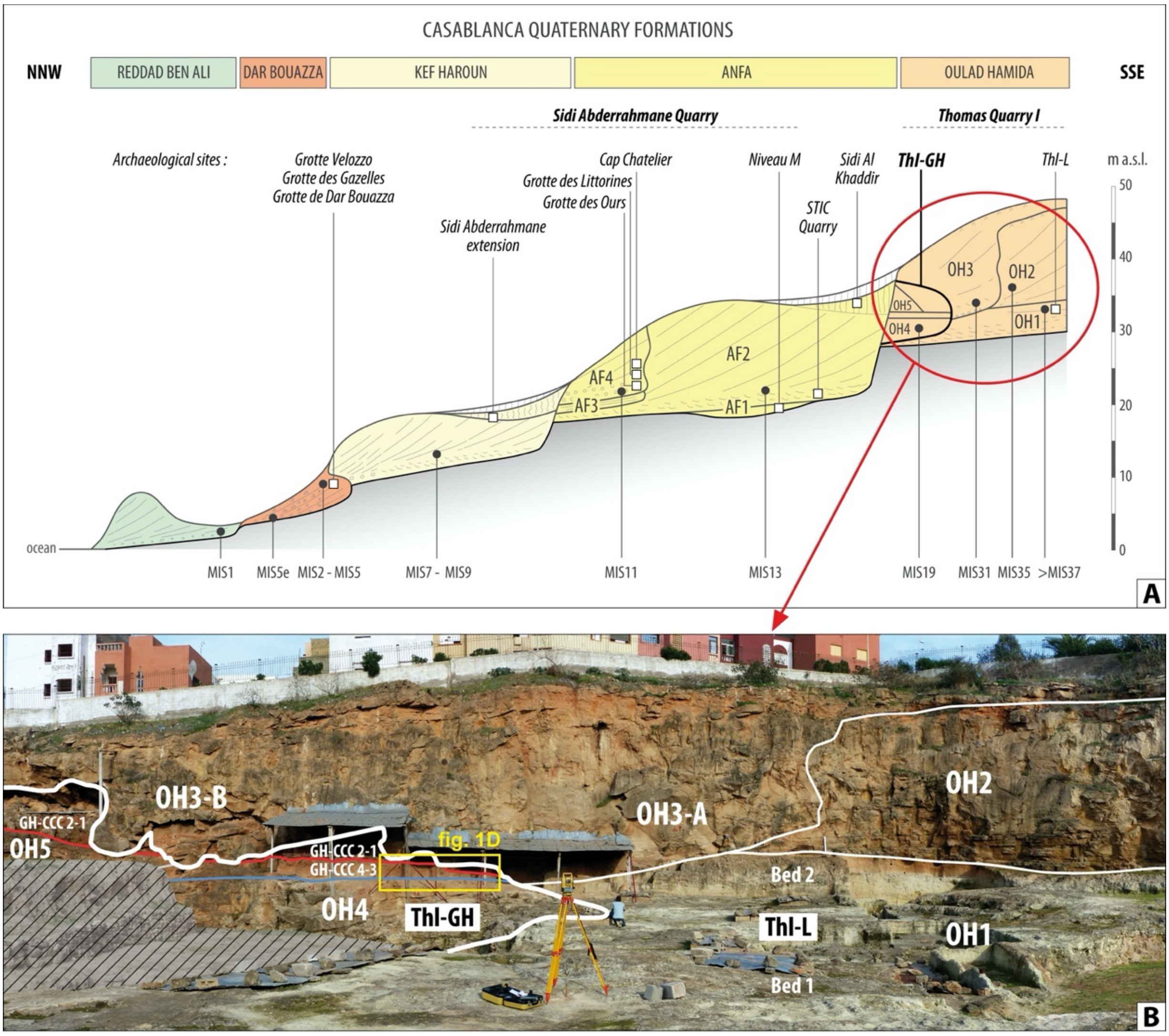

Figure 1. Panel (A) reports the general stratigrafic architecture of the late Early to Late Pleistocene Casablanca sequence. Panel (B) details the Oulad Hamida Formation (OHF) across Members OH1-OH4, GH-CCC and OH5 outcropping at Thomas Quarry I (D. Lefèvre, J.-P. Raynal, M. Rué). ThI-GH is the Grotte à Hominidés, while ThI-L is Thomas Quarry I Unit L.

The sedimentary formations of Thomas Quarry I (ThI) were succinctly considered as an extension of the Sidi Abderrahmane formations even if their altitude was higher[6]. The stratigraphic revision carried out in relation to the excavation of the *Grotte à Hominidés* (ThI-GH) and Thomas Quarry Unit L (ThI-L) archaeological sites showed that these deposits could not be continuous with those of the Sidi Abderrahmane Quarries and belonged to an earlier morphostratigraphic unit that we have called the Oulad Hamida Formation (OHF).

OHF sits on a major unconformity formed at an altitude of ~28 m above sea level (asl) at the expense of the Cretaceous and Palaeozoic substratum, ~10 m above the platform of the Anfa Formation (AF) in the Sidi Abderrahmane Quarries.

OHF includes Members – OH1 to OH4 from bottom to top (**Fig. 1B and ED Fig. 2A**). Each Member corresponds to a sedimentary sequence composed of associations and successions of facies characteristic of intertidal, supratidal, aeolian dune environments and continental slope deposits or soils and is bounded at its bottom and top by unconformities. Lithification of beach and aeolian sands is a rapid process. The regressive diagenetic sequence is characterized by an early phase of cementation in marine vadose environment and then by a phase of dissolution-precipitation of carbonates developed under continental vadose environment[7].These sedimentary sequences record sea-level fluctuations[3,5,7]. Marine transgressive phases mark calcarenite onlap and the carving of cliff and erosional notch at the base of previously lithified aeolian dunes while ensuing regressive phases involve seaward progradation and the buildup of new dunes **(Fig. 2)**. Thus, OH1 to OH4 deposits are correlative to sea-level high-stands (odd marine isotope stages –MIS–of the global Pleistocene eustatic record) and are preserved at positive elevations due to the moderate regional uplift of the area [1,3,5,7-9]. This sequence stratigraphic interpretation of the depositional cycles provides a robust framework to reconstruct the overall stratigraphic architecture of the Oulad Hamida Formation.

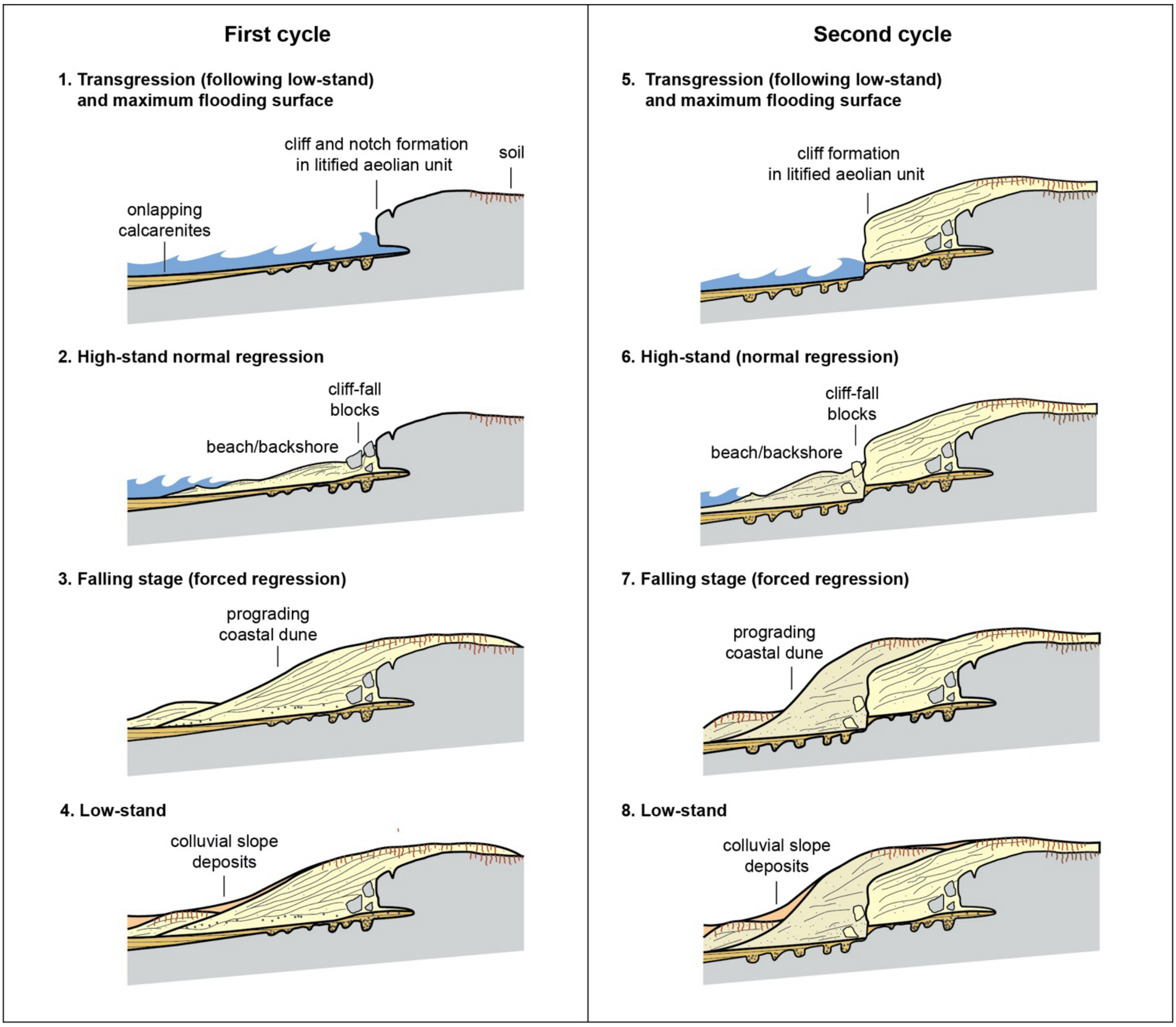

Figure 2. Morpho-stratigraphic evolution of coastal depositional setting across two relative sea-level cycles interpreted under the concepts of sequence stratigraphy (D. Lefèvre, G. Muttoni and S. Perini).

We provide below a description of Members OH1–OH4.

OH1 Member

Bed 1 is composed of coarse calcirudite at the base and stratified coarse-grained biocalcarenite.

Bed 2 – Unit L. The lower part of Bed 2 (Subunits L1 to L4) is a 2-3 m succession of yellow lenticular limestone beds with cross-bedding structure, composed of microsequences of mudstones and intraclastic sands. They are separated by emergent surfaces. This points to a continental fluviolacustrine hydrosystem with shifting channels and a temporary water table[5,10]. Bioclastic sands more or less bioturbated, partially decarbonated and cemented form the upper part (Subunit L5) of Bed 2. They represent aeolian deposits which accumulated in damp vegetated depressions with evidence of pedogenesis during warm episodes of subaerial exposure.

Subunit L1 at the bottom and L5 at the top contain early Acheulean lithic artifacts[11]. The mammalian assemblage comprises the genus *Hippopotamus*, bovids (gazelle, a medium-size alcelaphine, and a bovine), an *Equus* sp., an Elephantidae, and the suid *Kolpochoerus* sp.. Among the microfauna, there are reptiles, amphibians, and a few rodents, *Praomys*, *Paraethomys*, *Meriones* and *Gerbillus,* and the hare *Trischizolagus*[12-14].

The Bed 2 limestone deposits was dated to c. 0.99 ± 0.21 million years ago (Ma) using OSL method[15]. Bed 2 record a reverse magnetic polarity of the pre-Jaramillo Matuyama Chron and is at least older than MIS 35 in agreement with the faunal data. Saharian dust inputs (Si, Ti, Zr) link L1 to MIS 43 or 39, and subunit L5 to MIS 41 or early MIS 37[10].

OH2 Member. Overlying an erosional surface above OH1 deposits, there is a succession from bottom to top of 1) coarse biocalcarenites with a curved cross-bedding, 2) finer inclined planar-bedding biocalcarenites formed within intertidal depositional environments, 3) massive coarse bioclastic aeolianites about ten or so meters thick affected at their top by fersiallitic pedogenesis. OH2 presents a reverse magnetic polarity and its bottom part has recorded a brief normal palaeomagnetic event, possibly related to the Cobb Mountain Subchron (1.221–1.186 or 1.215–1.178 Ma, MIS 37/36 or 35)[10]. Thus, the OH2 intertidal deposits record a sea-level highstand which can be correlated with MIS 35.

OH3 Member is associated with a shoreline marked by a basal erosion platform and a cliff carved into OH1 and OH2 deposits, whose base is at an altitude of 37 m NGM. The OH3 deposits are composed from bottom to top of 1) blocs and pebbles from OH1 and OH2 calcarenite and limestone, 2) coarse and/or coquinoid biocalcarenites with inclined planar bedding, 3) aeolian calcarenites. OH3 presents at the bottom and the top a reverse magnetic polarity and belongs to the Matuyama Chron. Its middle part records a normal magnetic polarity indicating the Jaramillo Subchron (990-1070 ka)[10]. Therefore, OH3 intertidal deposits record a sea-level highstand which can be undoubtedly correlated with MIS 31.

OH4 Member is associated with a palaeoshoreline marked with cliff and deep cavities cut into OH1 and OH3 deposits. OH4 deposits are composed of a calcirudite with a coarse coquinoid matrix and plurimetric collapsed blocks of calcarenites and calcirudite coming from earlier formations, whose surfaces are smoothed, associated with a well-defined notch at an altitude of about 34 m asl; this chaos of blocks is onlapped by a fine, planar-bedding calcarenite which is well developed laterally. They record a sea-level highstand which may have correlated with the MIS 19 sea level highstand[10] (and this study).

Regarding the OSL dating, it is noteworthy that all the ages obtained across the entire OHF fall within a relatively narrow range of 360–440 ka[15]: 370 ± 58 ka for the youngest member (OH5, see below), 440 ± 38 ka for OH4, 444 ± 105 ka for OH3, and 359 ± 76 ka for the oldest member, OH2. This clustering of OSL ages appears inconsistent with stratigraphic and sedimentological evidence indicating that Members OH2 to OH5 were deposited during at least three distinct Pleistocene marine (glacioeustatic) cycles. As such, the OSL age range cannot be considered reliable for constraining the chronology of these formations.

Geological and chronological setting of the ThI-GH infilling

ThI-GH is a large and deep cavity associated with a cliffed shoreline carved by a marine highstand into late Early Pleistocene OH1 and OH3 deposits[3,7,10] (**Fig. 3 and ED Fig. 2B**).

OH4 marine deposits constitute the lower set of the infilling[3,7,9]. They are composed of 1) calcirudite with a coarse coquinoid matrix (Stratigraphic Unit – SU7), preserved in a well-defined notch at an altitude of about 34 m asl., and collapsed plurimetric blocks of calcarenites and calcirudite with blunt-surfaced coming from earlier formations, and 2) onlapped by a fine, planar-bedding calcarenite which is well developed laterally (OH4 SU6). Upwards, these sands become more massive (OH4 SU5) and are composed of well-sorted bioclastic and quartzose sands secondary cemented by sparitic calcite, derived mainly from reworked loose littoral deposits. Above follows, without any apparent

disconformity, the upper set of the infilling composed of continental deposits (GH-CCC - Continental Cave Complex). It can be subdivided in two parts, lower units SU4-SU3 and upper units SU2-SU1 separated by OH5 Member.

The limit between OH4 SU5 and GH-CCC SU4 is gradual to neat. SU4 and SU3 deposits are composed of bioclastic and quartzose reddish sands. The well-graded sorting as well as the mineralogical and petrographical constitution of the sediment indicate that it originates mainly from reworked loose littoral deposits. Clays associated could be due to in situ weathering of the sediments or/and were brought into the cavity with the rest of the sediment, together with rare soil aggregates coming from the ground surface by mud-flows. They are probably edified in several episodes and fed by the dismantlement of dunes and soils by low energy processes, likely runoff and wind transport.
Thus, the sedimentation progressively evolved without discontinuity from intertidal (SU6), supratidal (SU5) to continental (SU4) depositional environment. The intertidal biocalcarenites of SU6 and the reworked loose littoral sands of SU5 are interpreted as representing the MIS20/MIS19 transgression of sea-level and the subsequent maximum flooding surface, respectively. The continental deposits of SU4-3 are interpreted as part of the ensuing regressive system tract associated with the MIS19 highstand (**ED Fig. 4**).

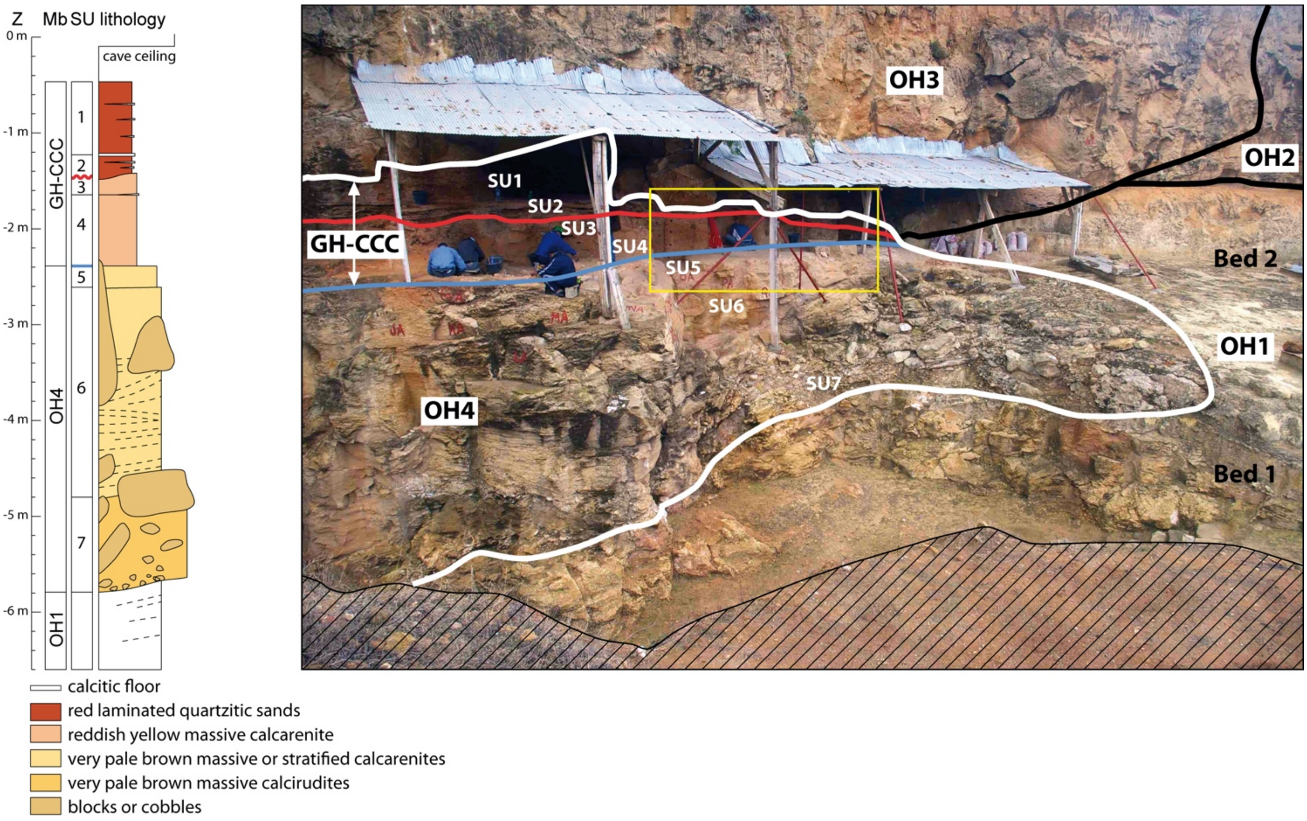

Figure 3. Lithostratigraphy of the ThI-GH site (D. Lefèvre, J.-P. Raynal, M. Rué). The yellow rectangle locates **Fig. 1D**.

SU5 and SU4 contain lithic artifacts, macro- and micro-fauna, and hominin fossils[16-18] (**ED Fig. 3A and 3B** and **Sup. Note 2 and Note 3**). Human fossils are distributed into SU5 and SU4 (**ED Fig. 3A to 3E**). The most representative specimens were found in *SU5*: an adult mandible (ThI-GH-10717), eight associated vertebrae (ThI-GH-10717/1 to 5, ThI-GH-10725, ThI-GH-10725/1, and ThI-GH-10726), and a child mandible (ThI-GH-10978). Although the precise location of the mandible

published in 1969 remains uncertain, analysis of its enclosing sediment suggests that it is probably stratigraphically contemporaneous with the SU5 and SU4 hominin remains.

OSL analyses on continental SU4 deposits yielded ages from 391±32 ka to 420±34[16,17], of the same ranging as the ages obtained from OH2 to OH5. Thus, OSL ages appear to be inconsistent with the evidence that these formations belong to at least three glacio-eustatic cycles, and for this reason can be disputed. A hominin tooth provided a laser abrasion ICP-MS age of 501 +89/-66 ka[16,17, 19]. New combined ESR/U-series dating was applied on three well preserved herbivorous teeth. The obtained ESR-US model ages are comprised between 538 ± 65 and 591 ± 103ka (**Sup. Note 4**). These scattered dates seem to indicate a Middle Pleistocene (473-694 ka) age for SU4. However, it should be noticed that the samples have high uranium content in the dental tissues and particularly in enamel. In this case, the internal dose rate probably is too strong for efficiently creating ESR signals in hydroxyapatite, which in turn results in an underestimation of De values to different extent. Hence, ESR-US results obtained at ThI-GH are considered as minimum ages **(Sup. Note 4)**.
On the other hand, biostratigraphic data suggested a late Early to early Middle Pleistocene age for SU5 and SU4[12-14,20] (**Sup. Note 2)**.

A clear discontinuity separates the lower continental deposits SU3 from the upper continental deposits SU2 and SU1. SU2 is composed of a multilayer stalagmitic floor interbedded with loose red sands, which grades laterally into a speleothem. This speleothem is dated to 92.3 ± 7.3 ka (inner part of a stalagmite) and 85.7 ± 5.2 ka (external part of a stalagmite) using U/Th method[17]. SU1 are massive bedded, rubified sands rich in microfauna and fragments of gastropod shells, originating from reworked superficial red soils (SU1).

OH5 Member. At the entrance of the cavity, the wide cross-bedding grey sands of an aeolianite are intercalated between the continental units SU3 and the upper units SU1-2. OH5 sands were dated at 440 ± 38 ka and 370 ± 58 ka[15].

SU5 and SU4 sedimentary processes

Field observations
Stratigraphic units SU3 and SU4, locally separated by a calcitic floor constitute the lower set of the continental deposits of the cave infilling (GH-CCC) (**ED Fig.2B and 2C, Fig. S4a**). At macroscopic scales, SU3 and SU4 are formed by a reddish yellow (5YR6/6) massive calcarenite containing few and sparse coarse fraction (>2 mm). Some flat millimeter beds, oriented in the direction of stratification or slightly inclined, appear at the top of SU4, probably formed by runoff or aeolian transport. These beds are sometimes enriched with red sands. Under these two units, SU5 is formed by a very pale brown (10YR8/3) massive calcarenite also containing a sparse and weak coarse fraction.
The boundary between SU4 and SU5 is clear and regular. It presents locally centimetric to decimetric irregularities of unknown origin (faunaturbation?). The global apparent dip is less than 2° towards the bottom of the cavity. No speleothem, concentration of lithic material or fauna remains are observed along the limit. The surface of the calcarenite blocks does not show any wear difference at the transition through this limit. This configuration, associated with the analytical results (see infra), suggests the absence of a sedimentary gap between SU4 et SU5.

Material and methods
Stratigraphic units were studied using a crossed geoarchaeological approach on both the lithic and faunal remains (spatial analysis, search for refittings, particle size distribution of the lithic industry) and the sediment (micromorphology, particle size distribution, magnetic susceptibility measurements,

and ED-XRF analyses, mainly on the OA24 square stratigraphic section). Micromorphology is based on the observation of large thin sections taken continuously. Particle size analyses were performed on bulk samples, after decarbonatation[22]. Volume magnetic susceptibility was measured along the section using a Bartington MS2K sensor with a vertical resolution of 2 cm[21]. Air-dried and crushed bulk samples (<2 mm) were analysed by ED-XRF using a calibrated portable spectrometer SPECTRO XSORT[23].

Analytical results

According to micromorphological observations, SU4 and SU5 are mainly composed by wellsorted bioclastic and quartzose sands secondary cemented by sparitic calcite (**Fig. 4b-g**). Compared to SU5, SU4 is characterized by a red-brown silty-clay sediment between sandy grains, red soil aggregates, coprolites and fauna remains (**Fig. 4g-h and 5**). The micromorphological interpretations suggest that the sedimentary material mainly comes from the reworking ofl ittoral deposits (possibly intertidal). After a weak weathering episode that occurred either before reworking or inside the karst, a lithification phase in freshwater phreatic conditions took place and a dissolution episode caused the formation ofl arge voids. The rare soil aggregates originate from the grounds urface and were probably entrained into the cavity at the same time as the rest of the sediment[16,17].

No diagnostic sedimentary figures are visible in the thin sections ofS U4 and SU5 ont he OA24 section. Only a slight flat orientation of elongated sand grains is perceptible, which may be caused by runoff episodes or aeolian inputs (**Fig. 5**). The limit between SU4 and SU5 is marked by the appearance of the red-brown silty-clay sediment (**Fig. 4e**). No indicators of non-deposition or erosion episode are visible.

Due to the diagenetic evolution, the initial texture of the sediment is difficult to appreciate. The particle size analyses reveal a plurimodal silty sand with no significant change towards the top (**Fig. 5**). The sandy mode (around 200-300 µm) derives from the calcarenitic bedrock. The lower modes have more complex origin (aeolian and continental inputs, *in situ* alteration, etc.). Coarse elements (gravels and cobbles) are rare and may result from anthropogenic (manuports) or natural inputs by reworking of previous coarse marine deposits.

The magnetic susceptibility signal indicates a light and progressive increase in magnetic particles towards the top of the SU4, probably caused by the enrichment in soil particles (red-brown siltyclay or red clay aggregates as detected by the thin sections (**Fig. 5**). Geochemical results show a global increase upwards in the rate of SiO2, Fe2O3, K2O and the Mn/Fe and Rb/Sr ratios, in parallel with a decrease in the content of CaO and the Ca/Ti ratio (**Fig. 5**). No major changes appear between the top of SU5 and the base of SU4. These results are consistent with a progressive continentalization of the environment during the deposition of SU4, maybe influenced by local modifications of the sedimentation, like a topographic modification of the cave entrance.

Conclusion

The sedimentary dynamic that contributed to the accretion of SU5 and SU4 is mainly generated by low energy processes, likely by runoff and wind transport. In SU5, the surface states of the lithic industry and the fauna remains are incompatible with a direct marine action. The sedimentation progressively evolved from a supratidal (SU5) to continental (SU4) environment, with no significant gap between these units that contain the human remains.

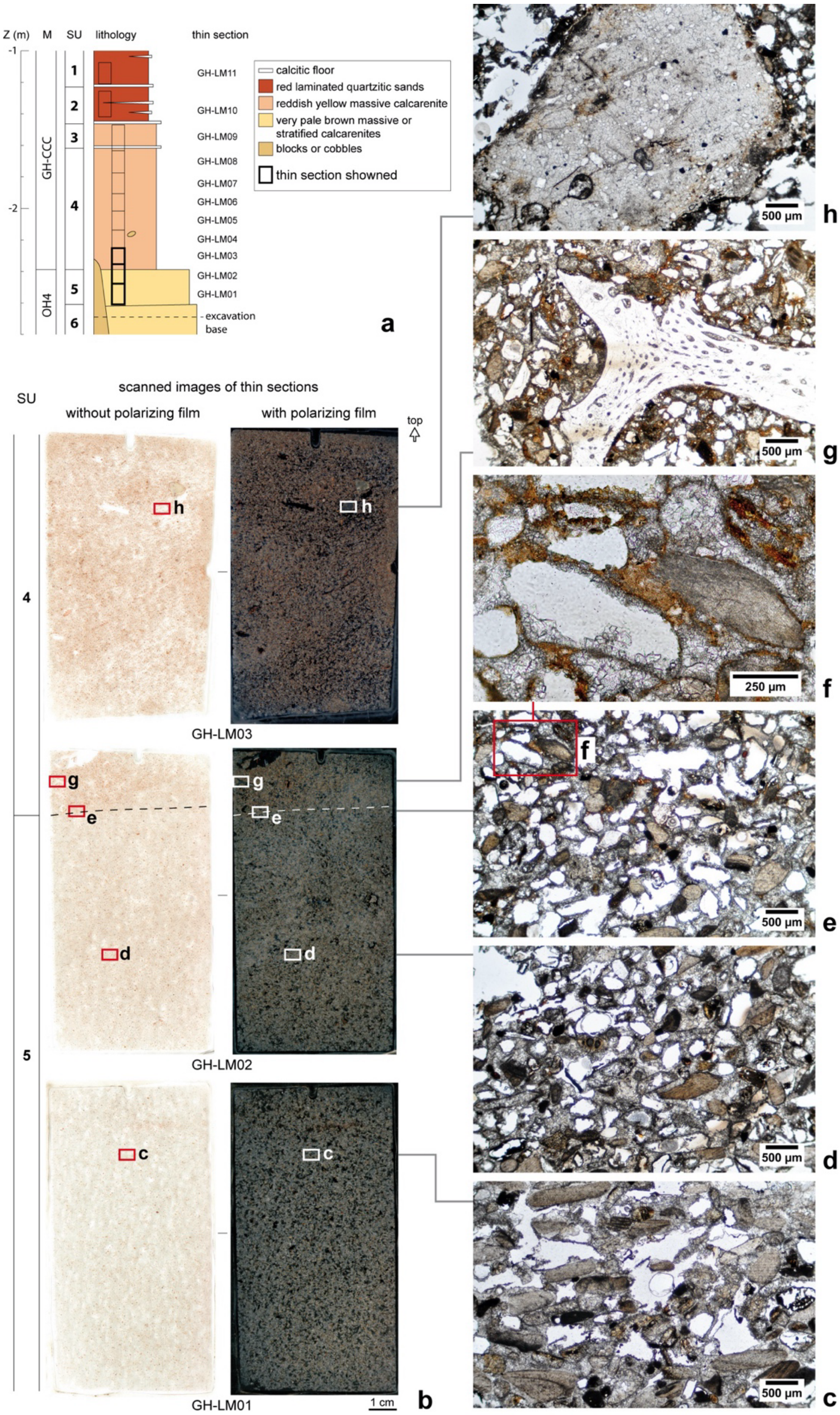

Figure 4. SU5 and SU4 microfacies (M. Rué). a: Location of the thin sections on the OA24 stratigraphic section; b: Scanned images of the thin sections; c and d: SU5 facies presenting locally a flat arrangement of the elongated sandy particles; e: Transition between SU4 and SU5 facies underlined by the appearance of a red-brown silty-clay sediment; f: Detail of the previous view; g and h: Fragment of microfauna (g) and coprolite (h) punctuating SU4 (absent in SU5).

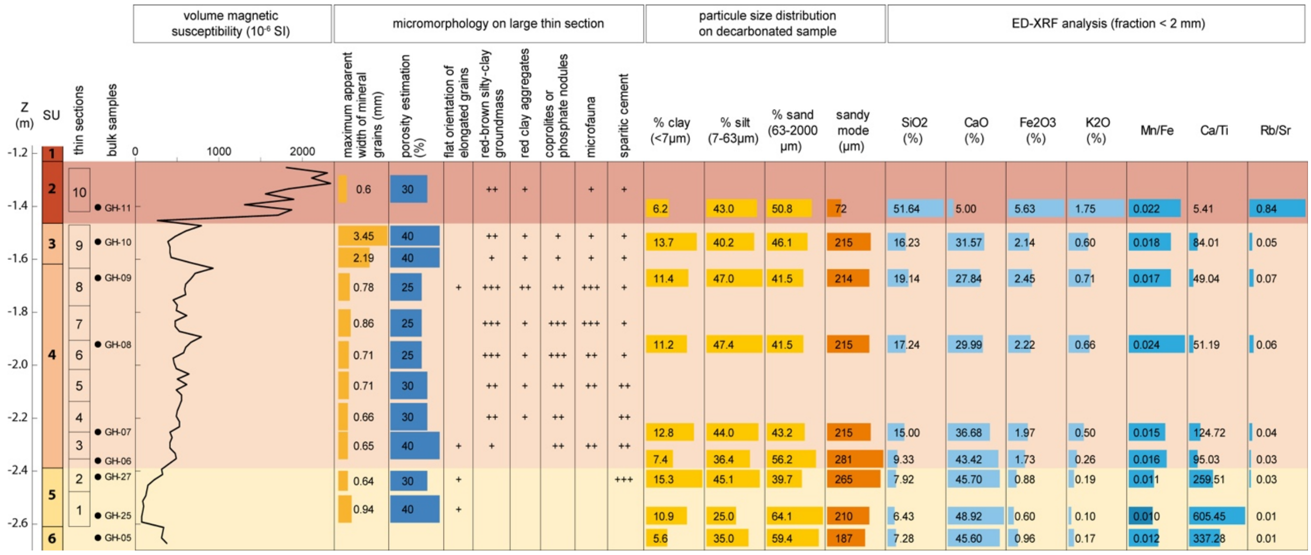

Figure S5. Vertical evolution on the OA24 stratigraphic section of magnetic susceptibility, microfacies, particle size distribution, and chemical elements (M. Rué).

Supplementary Note 2 - Fauna

D. Geraads and C. Daujeard

Following its first systematic description[24], a general account of the fauna from ThI-GH SU4 and SU5 and *Grotte des Rhinocéros* in Oulad Hamida Quarry 1 (OHQ1-GDR) has recently been provided[14]. In sedimentary units SU4 and SU5, bovids are dominant among ungulates, especially gazelles (*Gazella* cf. *atlantica*) and Alcelaphini, but bovines and reduncines are also present, along with the white rhinoceros (*Ceratotherium*), zebra (*Equus mauritanicus*), and two suids. *Theropithecus* is the only non-human Primate. Carnivores are abundant and diverse, including seal, bear, ratel, hyenas, leopard, and wild cat, but the jackal *Lupulella mohibi* largely dominates. Rodents are extremely common but not diverse, as in other North African sites, with which they share the main genera *Paraethomys*, *Mus*, *Gerbillus* s.l., and *Meriones*.

A short account of the fauna and a taxonomic list (**Tab. 1**) are provided below, together with comparisons with the nearby site of OHQ1-GDR, and with Tighennif in Algeria, which could date to at least 1 Ma[14,20-26].

Taphonomic context and depositional processes

The taphonomic analysis of Th1-GH concerns large mammals from SU4 and SU5[14,16,18]. All age groups of gazelles and alcelaphines have been documented, with juveniles dominating in the case of gazelles. Other herbivores, like bovines, rhinocerotids, equids, and suids mostly include juveniles or old individuals. We found some juvenile remains among jackals, but most specimens are from mature individuals, like other carnivores. Among small bovids, elements of the skull and upper limbs are well represented, contrasting with a paucity of elements of the trunk and small joint bones. In large bovids, axial and limb elements are more equally present. Other large mammals are mainly represented by elements of the autopod and isolated teeth.

Most of the bone surfaces have been preserved from post-depositional alterations, such as weathering, root etchings, or dissolution. The fossils are therefore in a good state of preservation, despite being highly fragmented. Nearly 40% of the long bones have green bone fractures. A large number of bones shows traces of carnivore teeth (19%) or have been semi-digested (6%). However, the presence of well-preserved articular parts shows that the large carnivores had only a moderate impact on the bone assemblage. The numerous coprolites and the dimensions of cortical pits confirm that bone-eaters of different sizes were using the cave, with jackal-sized tooth-marks being dominant. We have also observed carnivore tooth marks on both ends of a human femur. These marks clearly fit large carnivores, with a high probability that it is a hyena[27,28]. For other human remains, no biological traces have been observed, and their completeness and state of preservation state are excellent, indicating rapid burial conditions. Overall, we observed a high degree of homogeneity in bone surface modifications between faunal and human remains. Regarding human vertebrae and the adult mandible ThI-GH-10717, it is worth noting that they have been found in close proximity and that there are very few other examples of associated bones in the fauna, suggesting a particular post-mortem taphonomic history for these hominin remains.

Around 5% of the total bone assemblage present porcupine tooth marks. No cut marks have been found, questioning the human role in this bone accumulation. So, the main part of bone accumulations and modifications at ThI-GH are due to carnivores, through predation upon small- and medium-sized bovids and scavenging of larger taxa. Regarding the role of hominins, however, it must be noted that the studied assemblage comes from inside the cave, whereas any human occupation may have been concentrated closer to the entrance, an area which has not yet been excavated.

Systematic account

MAMMALIA

Rodentia – It should be noted that the rodent sample from ThI studied by Jaeger[29,30] does not come from ThI-GH, but from a more recent filling.

Muridae: dental dimensions of the mouse *Mus hamidae* are somewhat larger than in the type-locality OHQ1-GDR; it differs from the modern *Mus musculus* and *M. spretus* in the shape of its first lower molar[31]. According to[29], the evolution of *Praomys (Berberomys*) in North-Western Africa is anagenetic; however, *Praomys darelbeidae* from OHQ1-GDR is smaller than at Tighennif, so that it is likely that its evolution is more complex than previously suggested. The population of the extinct *Paraethomys tighennifae* is very similar to those of OHQ1-GDR and Tighennif. Two species of *Gerbillus* s.l. can be separated on the basis of m1 length. Subtle size differences can be documented between the various populations of *Gerbillus grandis* and of *Dipodillus* cf. *campestris*, a smaller species formerly included in *Gerbillus*[12,20,32-34]. Two species of jirds include the extinct *Meriones maximus* and the modern *Meriones shawi* (including *M. grandis* and the fossil form formerly called *M. maghrebianus*).

Arvicolidae: The mole vole *Ellobius* immigrated from Western Asia in the Early Pleistocene[30]; a tooth from ThI-GH is reminiscent of those from Tighennif.

Gliridae: The garden dormouse *Eliomys* from ThI-GH is smaller than *E. darelbeidae* from GDR.

Hystricidae: The porcupine *Hystrix* cf. *cristata* is rare in all sites, but a few gnawing marks have been documented.

Lagomorpha – *Trischizolagus*, present at both OHQ1-GDR, ThI-GH, and Tighennif[35] is a mostly Pliocene genus, and the Moroccan sites document its last known occurrence; it is unknown after the Early Pleistocene. A new species of *Lepus* is also present at OHQ1-GDR only, but the lagomorph sample is small in all sites.

Insectivora – Shrews are extremely numerous at OHQ1-GDR, where two species of *Crocidura* could be identified[36]; it is likely that the shrews from ThI-GH are of the same species.

Primates – Cercopithecidae: A few teeth from ThI-GH are similar to *Theropithecus oswaldi* from OHQ1-GDR and Tighennif[37]. The species is known in East Africa until Kapthurin, Kenya, at c. 0.5 Ma[38].

Carnivora – Carnivores are common in both OHQ1-GDR and ThI-GH caves, which they probably used as dens or shelters. They include 12 species at ThI-GH: a mongoose, two species of hyenas, a leopard, a wild cat, a bear, a honey-badger, an otter, a fox, a wild dog, an endemic jackal, and a seal. The carnivore fauna lacks the sabertooth genus *Homotherium* that is present (but quite rare) at Tighennif; besides this, the whole carnivore fauna is virtually identical with that of the Algerian site[39].

Hyaenidae: Two species of hyenas are present in the Thomas sites, and contributed to the accumulation of the fossil assemblage. As at Tighennif, *Hyaena hyaena* is less common than a species of *Crocuta* that probably belongs to *C. ultra*, defined in South Africa[40].

Felidae: A lower carnassial is intermediate in size between *P. pardus* and *P. leo* and is thus similar to the European *P. gombaszogensis* that could be a jaguar, but such identification would be too speculative. A few specimens represent a wildcat, *Felis* cf. *silvestris*, a group that is already present in the Pliocene.

There are three or four species of Canidae. As at Tighennif, the endemic canid *Lupulella mohibi* is the most common carnivore in all sites[39,41]. It has very omnivorous dental adaptations, but otherwise resembles the African jackals. A species similar to the modern Saharan fox, *Vulpes rueppelli*, is rare,

while the presence of the red fox *V. vulpes* is doubtful. ThI-GH and OHQ1-GDR sites also document two of the few known occurrences of the hunting dog *Lycaon magnus* in Africa.
Mustelidae: The honey-badger *Mellivora*, which already had a pan-African distribution in the Pliocene, is not rare in the main sites but the size of a braincase suggests species distinction from the modern *M. capensis*. An otter lower molar may indicate an extinct species of *Lutra*.
Ursidae: Bears are probably descended from the Tighennif species, but they are more common than in this site. They differ from contemporaneous European forms in their narrow teeth and long m2s and there is no compelling evidence that they are closely related to them; thus, they are best assigned to the endemic species *Ursus bibersoni*.

Perissodactyla – Rhinocerotidae: *Ceratotherium mauritanicum* is larger and more cursorial than the modern *C. simum*, that appears in East Africa in the late Pliocene or early Pleistocene, but its teeth are less adapted for grazing[42]. The survival of this primitive form points to an early age.
Equidae: The zebra is probably the same as at OHQ1-GDR, where it displays definite similarities with the Tighennif zebra, *Equus mauritanicus*, in contrast to many localities where the species has been cited on mere geographic grounds.

Artiodactyla – Suidae: There is no evidence of the *Metridiochoerus andrewsi/compactus* that was present at Tighennif; it is replaced here by the smaller, but probably ecologically similar warthog, *Phacochoerus* cf. *africanus*. An incomplete tooth from ThI-GH looks close to the Eastern African *Kolpochoerus majus*.
Bovidae: A large buffalo is present, probably the same as the OHQ1-GDR *Syncerus antiquus*. Alcelaphins are the most abundant bovids; the identity of the small form from ThI-GH is uncertain, but it is likely close to the most common species at GDR, which resembles the '?*Damaliscus* sp. nov.' from the Okote Member of Koobi Fora, Kenya[43]. Another alcelaphin from ThI-GH, *Connochaetes taurinus prognu*, is a primitive subspecies of the blue wildebeest known in East Africa as *C. taurinus olduvaiensis*, best known from Olduvai beds II to IV. It may be that two species of gazelles are present, but horn-cores best match the North African species *Gazella atlantica*.

Biochronological conclusions
The fauna from Tighennif still includes taxa already known in the earliest Pleistocene, such as *Metridiochoerus andrewsi/compactus*, *Homotherium*, or *Hippotragus gigas*, which are no longer present in the ThI-GH assemblage. Some of the other differences between these sites (concerning the suids and some bovids, or the abundance of the murid *Arvicanthis* at Tighennif) can probably be explained by ecological factors linked to the geographic location of the sites. By contrast, ThI-GH and OHQ1-GDR display a number of similarities with the Algerian site that fit an Early Pleistocene age. The main ones concern the rodents, *Trischizolagus*, *Theropithecus*, rhino, zebra, and most of the carnivores.

Among bovids, *Connochaetes taurinus prognu* is more primitive than the living blue wildebeest. None of the bovid taxa from ThI-GH and OHQ1-GDR is definitely more advanced than the Tighennif ones; indeed, the GDR alcelaphin that is reminiscent of the ?*Damaliscus* sp. nov.[43] from the Okote Member of Koobi Fora, even suggests an earlier age for the Moroccan sites. The rare *Kolpochoerus majus* is best known from the late Early and early Middle Pleistocene[44]. Rhinos definitely belong to a relative of the 'white' rhino, *Ceratotherium mauritanicum*, which went extinct in East Africa in the Late Pliocene or Early Pleistocene[42], even if this species survived later in North-Western Africa, its presence in the Moroccan sites suggests an early age. The zebra *Equus* cf. *mauritanicus* shows definite similarities with the early Pleistocene Tighennif species. *Theropithecus oswaldi* is poorly known after the Early Pleistocene. In addition, *Trischizolagus* and *Camelus thomasi* from OHQ1-GDR are further indicators of an early age, as they are unknown in the Middle Pleistocene.

	ThI-GH (SU4-SU5)	*GDR*
Mus hamidae	**cf.**	+
Praomys darelbeidae	+	+
Paraethomys tighennifae	+	+
Gerbillus grandis	+	+
Dipodillus cf. *campestris*	+	+
Meriones shawii/grandis	+	+
Meriones maximus	+	+
Ellobius atlanticus	**cf.**	+
Eliomys darelbeidae	**cf.**	+
Hystrix aff. *cristata*	+	+
Trischizolagus raynali	+	+
Lepus berbericus		+
Crocidura 'darelbeidae'	**cf.**	+
Crocidura cf. *tarfayaensis*		+
Theropithecus oswaldi	+	+
Herpestes cf. *ichneumon*	+	
Hyaena hyaena	+	+
Crocuta ultra	+	+
Panthera* cf. *pardus	+	+
Felis cf. *silvestris*	cf.	+
Lynx sp.	+	
Vulpes cf. *rueppelli*	+	
Vulpes vulpes	?	
Lupulella mohibi	+	+
Lycaon magnus	+	+
Mellivora* aff. *capensis	+	**?**
Lutra sp.	+	
Ursus bibersoni	+	+
Monachus monachus	+	
Ceratotherium mauritanicum	+	+
Equus* cf. *mauritanicus	+	+
Camelus thomasi		+
Hippopotamus* cf. *sirensis		+
Kolpochoerus* cf. *majus	+	
Phacochoerus cf. *africanus*	+	+
**cf. *Syncerus antiquus***	**?**	+
Redunca sp.	+	+
Oryx sp.	cf.	+
***?Damaliscus* sp.**	**?**	+
Connochaetes taurinus prognu	+	+
Gazella* cf. *atlantica	+	+

Table 1. Fauna list from ThI-GH, compared with that of the nearby OHQ1-GDR (extinct taxa in bold).

The most abundant carnivore at ThI-GH is the canid *Lupulella mohibi*, a species endemic to NW Africa. Its dental size is intermediate between Tighennif and the Anfa Formation Member 4 (D0 unit at Sidi Abderrahmane[6]), dated at 367 ± 34 ka[15]. The large *Lycaon*, *L. magnus* is also present at Tighennif and Elandsfontein. A honey-badger is probably worth taxonomic distinction from the living *Mellivora capensis*, and an otter (*Lutra* sp.) is perhaps also of an extinct species. The bear *U. bibersoni* resembles the European *Ursus deningeri* but differs in its narrow lower teeth.

In summary, although all faunal indicators are not fully convergent, we may conclude that Tighennif is certainly the earliest of these sites, but also that ThI-GH and OHQ1-GDR are not very much younger, as their faunas are close to that of this early Pleistocene site, suggesting a late Early Pleistocene age. Comparison with African sites of similar ages (See *infra*) shows that the ratio of extinct vs. living species of large mammals is compatible with a **late Early to early Middle Pleistocene age**, thus in good agreement with paleomagnetic results. Even though a number of factors (the first of which being probably identification biases) lessen the correlation between this ratio and age, the estimated ages of ThI-GH and OHQ1-GDR fit pretty well within the general trend (**Fig. S6**). Even though sample size is small, ages much younger than those provided by the paleomagnetism are very unlikely.

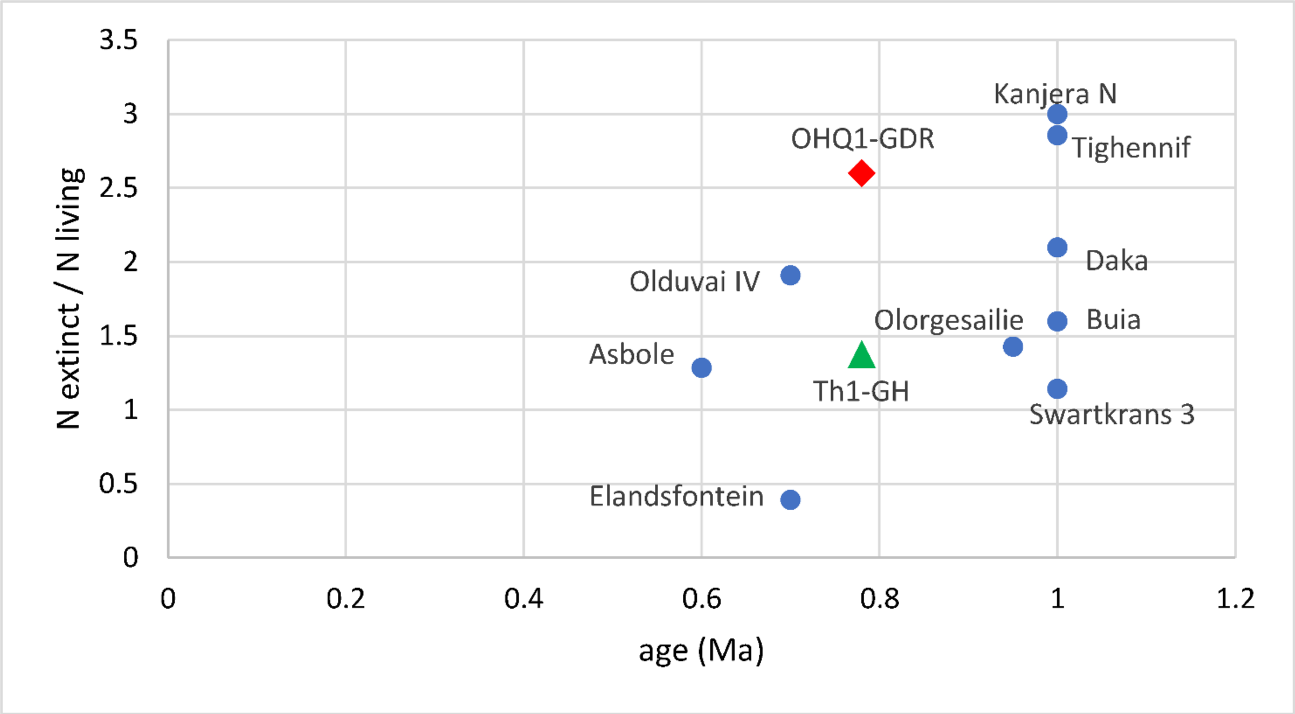

Figure 6. Biplot of approximate ages vs. ratio of extinct / living species in some African sites of late Early to early Middle Pleistocene age (full data below).

Faunal lists used to prepare Fig. S6.

They are mostly based upon published lists but, the extinct vs. living status may have been modified according to the material upon which the identifications are based (e.g., postcranials seldom provide reliable identifications at species level). We suspect that the number of living taxa is over-estimated at Elandsfontein). Species whose extinct vs. living status is unknown are not considered.

THOMAS QUARRY I-GH, SU4 AND SU5

0.78 Ma extinct species = 11 / living species = 8

Phacochoerus africanus, ***Kolpochoerus*** **cf.** ***majus,*** Bovini indet., *Kobus* sp., *Oryx* sp., *Connochaetes taurinus,* Alcelaphini indet., ***Gazella*** **cf.** ***atlantica, Ceratotherium mauritanicum, Equus*** **cf.** ***mauritanicus, Lupulella mohibi, Lycaon magnus,*** *Vulpes* cf. *rueppelli, Vulpes vulpes?,* ***Mellivora*** **aff.** ***capensis,*** *Lutra* sp., ***Ursus bibersoni,*** *Herpestes* cf. *ichneumon,* ***Crocuta ultra****, Hyaena hyaena,* ***Panthera*** **cf.** ***gombaszogensis,*** *Lynx* sp., *Felis* cf. *silvestris, Monachus monachus,* ***Theropithecus oswaldi***

OULAD HAMIDA QUARRY 1-GROTTE DES RHINOCEROS
0.7 Ma extinct species = 13 / living species = 5
Camelus* cf. *thomasi*,** *Phacochoerus africanus*, ***Hippopotamus* cf. *sirensis*,** Bovini indet., *Oryx* sp., *Connochaetes taurinus*, **?*Damaliscus* sp., *Gazella* cf. *atlantica*, *Ceratotherium mauritanicum*, *Equus* cf. *mauritanicus*, *Lupulella mohibi*, *Lycaon magnus*,** *Vulpes* cf. *rueppelli*, *Mellivora* sp., ***Ursus bibersoni*, *Crocuta ultra*,** *Hyaena hyaena*, ***Panthera* cf. *gombaszogensis*, *Lynx thomasi*,** *Felis* cf. *silvestris*, ***Theropithecus oswaldi

TIGHENNIF, ALGERIA[26, 39, 40]
1 Ma extinct species = 20 / living species = 7
Loxodonta atlantica, ***Camelus thomasi***, ***Metridiochoerus andrewsi/compactus***, *Hippopotamus sirensis*, **cf. *Mitilanotherium/Macedonitherium* sp.**, ***Tragelaphus algericus***, ***'Bos' bubaloides***, *Kobus* sp., *Oryx* cf. *gazella*, ***Hippotragus* cf. *gigas***, *Connochaetes taurinus*, ***Parmularius ambiguus***, ***Gazella dracula***, ***Gazella* cf. *atlantica***, *Gazella* sp. B, Caprini indet., ***Ceratotherium mauritanicum***, ***Equus mauritanicus***, ***Lupulella mohibi***, ***Lycaon magnus***, *Vulpes* cf. *rueppelli*, *Mellivora capensis*, *Poecilictis* cf. *libyca*, ***Enhydrictis hoffstetteri***, ***Ursus* aff. *bibersoni***, ***Crocuta ultra***, *Hyaena hyaena*, ***Panthera* aff. *leo***, *Felis silvestris*, *Lynx* sp., Felidae gen. et sp. indet., ***Homotherium* sp.**, ***Theropithecus oswaldi***

BUIA, ERYTHREA[45]
1 Ma extinct species = 8 / living species = 5
Tragelaphini, *Kobus* cf. *ellipsiprymnus*, ***Hippotragus gigas***, ***Bos buiaensis***, *Tragelaphus* cf. *spekei*, *Giraffa jumae/camelopardalis*, ***Metridiochoerus modestus***, ***Kolpochoerus majus***, ***Kolpochoerus olduvaiensis***, **aff. *Hippopotamus karumensis***, *Hippopotamus gorgops/amphibius*, *Equus* cf. *grevyi*, *Ceratotherium simum*, ***Elephas recki***, cf. *Crocuta sp.*, ***Theropithecus* cf. *oswaldi***

ASBOLE, ETHIOPIA[46,47]
0.6 Ma extinct species = 9 / living species = 7
Elephas recki, ***Metridiochoerus modestus***, ***Kolpochoerus majus***, *Hippopotamus* cf. *amphibius*, *Giraffa* cf. *camelopardalis*, *Tragelaphus scriptus*, *Taurotragus* sp., ***Bos* sp.**, *Syncerus* sp.?, cf. *Connochaetes* sp., ***Damaliscus niro***, *Oryx* sp., *Kobus kob*, *Redunca* sp., *Gazella* cf. *thomsoni*, *Gazella* aff. *granti*, *Diceros* cf. *bicornis*, *Equus* sp., *Canis* sp., *Mellivora capensis*, *Herpestes* cf. *ichneumon*, ***Ichneumia* aff. *albicauda***, *Hyaena hyaena*, *Crocuta sp.*, *Felis* cf. *libyca*, *Panthera* cf. *leo*, ***Chlorocebus* aff. *aethiops***, *Chlorocebus* cf. *patas*, ***Colobus* sp.**, *Papio hamadryas*, ***Theropithecus oswaldi***

DAKA, ETHIOPIA[20, 48(with corrections)]
1 Ma extinct species = 21 / living species = 10
Elephas recki, ***Kolpochoerus majus*** , ***Kolpochoerus olduvaiensis***, ***Metridiochoerus compactus***, ***Metridiochoerus* cf. *hopwoodi***, *Phacochoerus* sp., ***Hippopotamus* cf. *gorgops***, *Giraffa sp.*, ***Sivatherium* sp.**, ***Pelorovis antiquus***, ***Pelorovis turkanensis***, ***Syncerus acoelotus***, *Tragelaphus strepsiceros*, *Tragelaphus* cf. *imberbis*, *Tragelaphus* cf. *scriptus*, ***Hippotragus* cf. *gigas***, *Oryx gazella*, *Connochaetes taurinus*, ***Megalotragus kattwinkeli***, *Damaliscus* sp., ***Numidocapra crassicornis***, ***Parmularius angusticornis***, *Kobus ellipsiprymnus*, *Kobus kob*, ***Kobus* aff. *ancystrocera***, *Aepyceros* cf. *melampus*, ***Bouria anngettyae***, ***Nitidarcus asfawi***, *Gazella* sp., *Antidorcas* sp., *Equus* sp., ***Eurygnathohippus* cf. *cornelianus***, *Ceratotherium simum*, *Diceros* sp., ***Crocuta ultra***, *Panthera* cf. *leo*, *Panthera* cf. *pardus*, ***Theropithecus oswaldi***, ***Cercopithecoides alemayehui***

KANJERA NORTH, KENYA[49]

1 Ma extinct species = 12 / living species = 4

Cercopithecus sp., ***Theropithecus oswaldi***, Felidae indet., Hyaenidae indet., ***Metridiochoerus compactus*?, *M. hopwoodi*?, *Kolpochoerus heseloni***, *Phacochoerus* sp.?, **Hippopotamidae small**, ***Hippopotamus* cf. *gorgops***, Tragelaphini, ***Pelorovis* cf. *oldowayensis***, ***Syncerus acoelotus***, Reduncini, *Kobus* sp., Cephalophini, ***Parmularius angusticornis***, ***Megalotragus*** sp., Antilopini, *Gazella* sp., Hippotragini, *Equus* sp., ***Eurygnathohippus* sp.**, *Diceros bicornis*, *Ceratotherium simum*, *Loxodonta africana*, ***Palaeoloxodon recki***, *Phataginus giganteus*

OLORGESAILIE MB 1 & 7, KENYA[40, 50]

0.95 Ma extinct species = 10 / living species = 7

Elephas recki, *Phacochoerus* sp., ***Metridiochoerus hopwoodi***, ***Metridiochoerus compactus***, ***Hippopotamus* cf. *gorgops***, *Giraffa* sp., *Tragelaphus scriptus*, *Taurotragus* sp., ***Pelorovis* cf. *antiquus***, *Redunca* sp., *Connochaetes* sp., ***Megalotragus* sp.**, *Alcelaphus sp.*, *Aepyceros* sp., *Gazella* sp., *Ceratotherium simum*, ***Eurygnathohippus* sp.**, *Equus* cf. *grevyi*, ***Equus oldowayensis***, *Equus* cf. *quagga*, *Canis* cf. *mesomelas*, *Panthera leo*, *Genetta* sp., ***Crocuta ultra***, ***Theropithecus oswaldi***

OLDUVAI BED IV, TANZANIA[51-55]

0.7 Ma extinct species = 21 / living species = 11

Elephas recki, ***Phacochoerus modestus***, ***Kolpochoerus limnetes***, ***Kolpochoerus majus***, ***Metridiochoerus hopwoodi***, ***Metridiochoerus compactus***, ***Hippopotamus gorgops***, ***Sivatherium maurusium***, *Giraffa* sp., *Tragelaphus strepsiceros*, *Tragelaphus spekei/scriptus*, ***Taurotragus arkelli***, ***Pelorovis antiquus***, ***Syncerus acoelotus***, ***Thaleroceros radiciformis***, ***Megalotragus kattwinkeli***, *Connochaetes taurinus*, ***Parmularius rugosus***, ***Damaliscus niro***, ***Rabaticeras arambourgi***, ***Antidorcas recki***, *Aepyceros melampus*, *Ceratotherium simum*, ***'Hipparion' ethiopicum***, ***Equus oldowayensis***, *Equus burchelli*, ***Canis africanus***, *Canis mesomelas*, *Hyaena* sp., *Felis libyca*, *Panthera leo*, *Panthera pardus*, ***Theropithecus oswaldi***, *Papio* sp.

ELANDSFONTEIN MAIN, SOUTH AFRICA[56]

0.7 Ma extinct species = 8 / living species = 23

Loxodonta atlantica, *Hippopotamus amphibius*, *Ceratotherium simum*, *Diceros bicornis*, ***Equus capensis***, *Equus quagga*, *Taurotragus oryx*, ***Pelorovis antiquus***, *Redunca arundinum*, ***Hippotragus gigas***, *Connochaetes gnou*, ***Rabaticeras arambourgi***, ***Damaliscus* aff. *lunatus***, ***Damaliscus* sp.nov.**, *Antidorcas marsupialis*, *Raphicerus melanotis*, *Canis mesomelas*, *Vulpes chama*, *Ictonyx striatus*, *Mellivora capensis*, *Herpestes pulverulentus*, *Atilax paludinosus*, *Suricata suricatta*, *Civettictis civetta*, *Crocuta crocuta*, *Parahyaena brunnea*, *Felis libyca*, *Felis caracal/serval*, *Panthera leo*, *Panthera pardus*, ***Megantereon whitei***

SWARTKRANS MB 3, SOUTH AFRICA[57]

1 Ma extinct species = 15 / living species = 15

Papio hamadryas, ***Theropithecus oswaldi,*** *Panthera leo, Panthera pardus, Acinonyx jubatus, Felis lybica,* ***Megantereon whitei, Crocuta ultra,*** *Parahyaena brunnea,* ***Chasmaporthetes nitidula,*** *Proteles* sp., *Canis mesomelas,* ***Canis* sp.**, *Vulpes* sp., *Aonyx capensis, Atilax* sp., *Cynictis penicillata, Suricata suricatta, Genetta tigrina, Orycteropus afer, Manis* sp., **cf. *Elephas* sp., *Procavia transvaalensis, Procavia antiqua, Eurygnathohippus libycum, E. capensis,*** *E. quagga,* ***Phacochoerus antiquus, Metridiochoerus andrewsi,*** *Hippopotamus* sp., Giraffidae indet., ***Megalotragus* sp.**, *Connochaetes* sp., *Damaliscus* sp., ***Makapania* sp., *Antidorcas recki*,** *Antidorcas* sp., *Gazella* sp., *Oreotragus oreotragus, Raphicerus campestris, Syncerus* sp., *Taurotragus oryx, Tragelaphus strepsiceros, Hippotragus* sp., *Pelea* sp., *Kobus leche*

Supplementary Note 3 - Lithic assemblage

R. Gallotti, A. Mohib, and J.-P. Raynal

The lithic assemblage recovered by recent excavations at ThI-GH in SU4 and SU5 is composed of 313 artifacts and 1483 unmodified cobbles and pebbles, which show a high degree of homogeneity in surface physical state. Artifacts were manufactured mainly on various quartzites available close to the site as cobbles of small to medium size and rare blocks as well as a few silicite nodules derived from the phosphatic plateau in the hinterland of the Meseta and collected from beach deposits in secondary position[9].

The production of flakes was the major objective reflected by both the quartzite and silicite assemblages and is documented by a large number of cores, while the number of flakes is extremely limited. Only two handaxes on cobble were discovered (**Fig. S7**), most probably manufactured outside the cave and then imported.

Quartzite small flaking documents the adoption of various flaking methods well adapted to the cobble morphology.

Plano-convex cobbles were exploited mainly through a unifacial unidirectional flaking from a flat cortical striking platform (**Fig. 8**: 3-5), while in the case of bi-convex cobbles one or two detachments rectify a cobble surface to create a striking platform before unidirectional flaking (**Fig. 8**: 6). When these cores are overexploited, they are similar to *galets tronqués* (**Fig. 8**: 8).

The unidirectional exploitation involves the peripheral convexity of flat and thick cobbles using a flat surface as striking platform (**Fig. 8**: 12). Bi-convex cobbles were usually exploited on two flaking adjacent surfaces, alternating their use as flaking surface and as striking platform (**Fig. 8**: 1, 2). Bi-convex cobbles were also exploited on one surface through a centripetal/cordal exploitation from a natural or partially rectified striking platform (**Fig. 8**: 13, 15, 16). In few cases the centripetal exploitation involves two adjacent surfaces linking these cores to the discoid concept (**Fig. S8**: 14)[58-61]. Other few cores (**Fig. 8**: 20) display two to four adjacent flaking surfaces exploited through unidirectional removals and a continuous rotation of the core (SSDA, Système par *Surface de Débitage Alternée*[62,63]) or show a multifacial multidirectional exploitation where core surfaces were alternatively flaked through multidirectional removals without a clear organisation of the reduction process (**Fig. 8**: 17,18).

Flake production is also the only objective of silicite core knapping. Core blanks are small pebbles flaked preferentially on a single surface using unidirectional (**Fig. 8**: 10) or bipolar removals (**Fig. 8**: 7). The bifacial partial exploitation is also documented (**Fig. 8**: 11) and only one core was exploited on several surfaces using multidirectional removals (**Fig. 8**: 19).

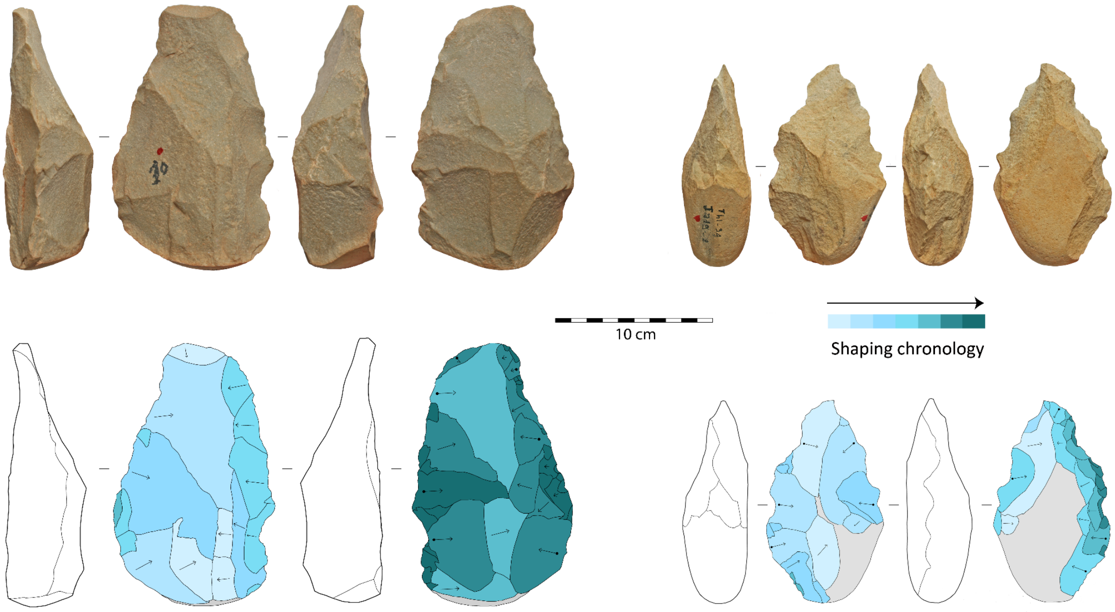

Figure S7. Large Cutting Tools from ThI-GH (R. Gallotti).

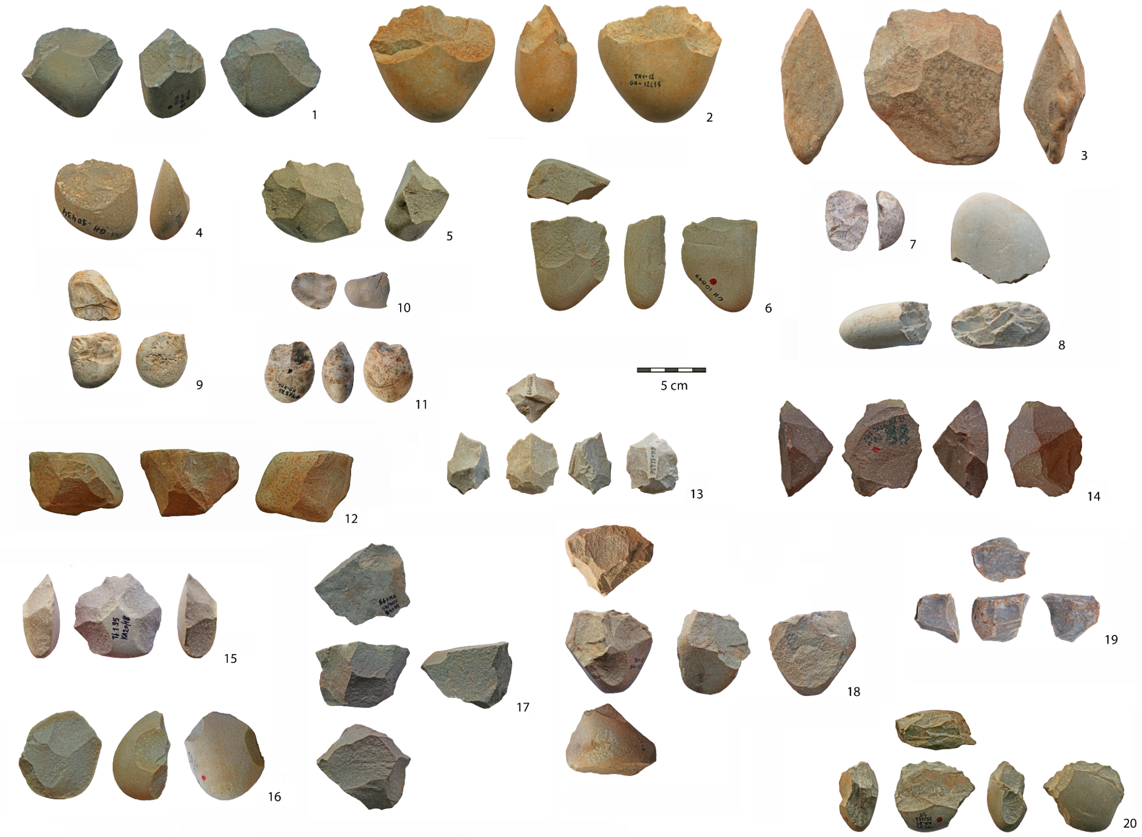

Figure 8. Cores from ThI-GH (photos **R. Gallotti).** 1, 2: bifacial partial cores, quartzite; 3-6: unifacial unidirectional cores, quartzite; 7: bipolar core, silicite; 8: *galet tronqué,* quartzite; 9, 10: unifacial unidirectional cores, silicite; 11: bifacial partial core, silicite; 12: peripheral unidirectional core, quartzite; 13-16: centripetal cores, quartzite; 17-18: multifacial multidirectional cores, quartzite; 19: multifacial multidirectional core, silicite ; 20: SSDA core, quartzite.

Supplementary Note 4 - ESR Dating

C. Falguères, Q. Shao, and J.-J. Bahain

Previous OSL dating of quartz[15] provided an age range between 360 and 470 ka. Recently, combined Electron Spin Resonance and U-series dating approach was directly applied to a hominid tooth (first right upper premolar) unearthed in OA23-24 square, in SU 4, close to the analyzed herbivorous teeth in this work, and yielded an age estimate of 501^{+94}_{-76} ka[16,19] **(ED Fig. 3A and 3B).** OSL analyses were also performed on two sediments collected immediately above and below the hominid tooth and yielded ages of 420 ± 34 ka and 391 ± 32 ka, respectively[16,17] **(ED Fig. 3A and 3B).** All these data place ThI-GH SU4, within the Middle Pleistocene but the disagreement between the age estimates on the hominid tooth and on the sediments on one hand and because the faunal assemblage, showing similarities with that of Tighennif site, Algeria, on the other hand, suggests an older age. Therefore, it was decided to make additional Electron Spin Resonance and U-series analyses on mammal teeth allowing a better comparison for checking the contemporaneity between fossils and sediments.
Combined ESR/U-series dating was applied on four well preserved herbivorous teeth. This method has been applied since more than 30 years successfully. TH0801 and TH0804 (Alcelaphini), TH0802 (Bovini) were discovered in squares RA26, QA25, RA24, at the depth of -2.4m, -2.1m and -2.3m respectively. The obtained ESR-US model ages are comprised between 538 ± 65 and 591 ± 103ka. The TH0803 (Bovini), discovered in square IA22, at the depth of -1.7m, yields a younger age of 380 ka probably in relation with a small equivalent dose (D_e), about 50% lower than the mean D_e determined for the other teeth and despite an annual dose (D_a) 33% smaller than the mean D_a of the other samples **(Tab. 2)**. It should be noticed that the ThI-GH samples have high uranium content in the dental tissues and particularly in enamel (between 13 and 29 ppm). In the case of high uranium in enamel, the internal dose rate probably is too strong for creating efficiently ESR signals in hydroxyapatite, which in turn results in an underestimation of D_e values to different extent, as observed in OHQ1-GDR[64]. Hence, ESR-US results obtained at ThI-GH are considered as minimum ages. The ThI-GH SU4 would be coeval with the upper part of OHQ1-GDR sequence for which a weighted mean age of 611 ± 64 ka was calculated.

Samples	Square	Depth (cm)	De (Gy)	(β=γ) sed +cosmic dose (µGy/a)	(α+β) internal dose (µGy/a)	Total dose rate (µGy/a)	p or n value		ESR/U-series ages (ka)
							enamel	dentine	
TH0801	RA26	-236	4040 ± 111	533 ± 40	6303 ± 1249	6836 ± 1255	-0,12 ± 0,19	-0,27 ± 0,17	591 ± 103
TH0802	RA24	-227	3412 ± 104	378 ± 34	5964 ± 850	6342 ± 857	-0,61± 0,06	*0,0020 ± 0,003*	538 ± 65
TH0803	IA22	-168	1916 ± 46	908 ± 152	4188 ± 693	5096 ± 707	-0,49 ± 0,09	-0,59 ± 0,08	376 ± 49
TH0804	QA25	-206	3437 ± 72	428 ± 52	5855 ± 628	6283 ± 632	-0,70 ± 0,03	*0,0018 ± 0,0002*	547 ± 52

Table 2: internal and external dose rate, p and n-values and ages of teeth analyzed in ThI-GH site

Method

Application of ESR-US dating to herbivore teeth[65-67] considers over time the evolution of the dose rate as a result of postmortem uranium incorporation, through the calculation of a specific U-uptake parameter (p-parameter) for each dental tissue. When samples show evidence of uranium loss, it is not possible to calculate an age though a recent model called the Accelerating uptake model, AU-ESR, allows the consideration of a slight uranium leaching[68]. The interest of such a combined approach is the production of a single age for each tooth, considering all of the analytical data. Age estimates depend then on the evaluation of the uranium uptake rate that occurred over the burial history of the sample, whereby the main difficulty arises from the post-mortem incorporation of uranium causing a change of the dose rate emitted by the different tissues constituting the tooth. At the difference of former models proposed to describe U-incorporation kinetics, such as early uptake and linear

uptake[69,70], the ESR-US approach takes into account both ESR and US data (radioelement contents, isotopic ratios, palaeodoses, external gamma dose rate) allowing a better description of uranium uptake into the different dental tissues, as well as the determination of a single age for each sample, through the calculation of an uptake parameter p for each tissue[67,71]. This combined approach is now reliable for dating materials from archeological and paleontological contexts during the entire Middle Pleistocene[72-74].

Samples

Four herbivore teeth were collected between 2005 and 2008 during excavation campaigns. They were extracted from SU4 and their coordinates are mentioned in the **Tab. 2**. The tissues (enamel and dentine) of each tooth were separated and cleaned mechanically using a dental drill. No cement layer in sufficient quantity was observed. So, for calculation, a dentine-enamel-sediment model was considered. Enamel was then ground and sieved to 100-200 µm grain-size fraction. Nine enamel aliquots (each ≈100 mg) were irradiated between 800 and 32 000 Gy. ESR signal intensities of irradiated and natural aliquots were measured at room temperature by an EMX Bruker X-band spectrometer, with the following measurement conditions: 1mW microwave power, 0.1 mT amplitude modulation, 10 mT scan range, 80 ms time constant, and 100 kHz modulation frequency, for three repeated measurements. The equivalent doses D_e were determined from the asymmetric enamel signal at g = 2.0018 by the additive method, using a linear plus exponential fitting, EXPLIN with 1/I2 weighting, which provides the best fitting for the Thomas data set.

Uranium-Series analyses were carried on ICP-QMS at LSCE, Gif-sur-Yvette, France, for each dental tissue following the chemical protocol described in[75] and adapted in[76]. For each sample, 2 g were used for the analyses and about 0.1g of ^{233}U-^{236}U-^{229}Th mixed spike was added. Samples were dissolved in HNO_3 acid. Chemical extractions and purifications were performed according to the protocol presented in Pons-Branchu et al.[77].

ESR/US ages were calculated with the ESR-DATA program[67], using an alpha efficiency of 0.13 ± 0.02[78] and Monte Carlo beta attenuation factors based on the thickness of the tooth enamel with outer layers removed[79]. In addition, water content was estimated to be 0 wt% in the enamel and 7 wt% in the dentine. The external gamma dose rate was measured *in situ* with a gamma ray spectrometer using a NaI detector by the threshold method[80], and completed by measuring sediment close to the teeth by high resolution low background gamma ray spectrometer equipped with a germanium detector in the Paris lab **(Tab. 3).** D_e are comprised between 3000 and 4000 Gy for the deepest samples. The sample TH0803 taken at -1.70 m yields a smaller D_e (ca 1916 Gy).

Sediment	238U ppm	232Th ppm	K %
SedTH01	2,86 ± 0,09	0,99 ± 0,08	0,25 ± 0,01
SedTH02	2,22 ± 0,11	1,11 ± 0,11	0,18 ± 0,01
SedTH03	5,60 ± 0,11	2,10 ± 0,11	0,49 ± 0,01
SedTH04	3,37 ± 0,11	1,37 ± 0,11	0,33 ± 0,01

Table 3: U, Th, and K measured for calculating the external dose in sediments of ThI-GH

US and ESR age data obtained on *SU4* teeth are shown in **Table 2** and additional analytical data in **Table 4**. U contents of dentins and enamels range between 62 and 85 ppm and 13 and 29 ppm,

respectively. In each tooth, consistent $^{234}U/^{238}U$ activity ratios are observed in dental tissues ranging between 1.119 and 1,541 in dentin and between 1,295 and 1,581 in enamel. $^{230}Th/^{234}U$ ratios are smaller than the equilibrium value in the error range. All p-parameters are comprised between 0 and -1 showing evidence of post-mortem uranium incorporation. Reliable ESR/U-series ages can be calculated excepting for two dentin tissues in which uranium was slightly leached but which can be calculated either using p or n values and yielding the same age in the error range.

Sample	square	z (cm)	U ppm		234U/238U		230Th/234U		222Rn/230Th	
			enamel	dentine	enamel	dentine	enamel	dentine	enamel	dentine
TH0801	RA26	-237	28,98 ± 0,08	84,83 ± 0,24	1,295 ± 0,004	1,119 ± 0,006	0,850 ± 0,007	0,863 ± 0,017	0,65 ± 0,2	0,30 ± 0,2
TH0802	RA24	-227	15,58 ± 0,04	85,15 ± 0,26	1,465 ± 0,006	1,380 ± 0,005	0,946 ± 0,016	1,005 ± 0,014	0,67 ± 0,2	0,31 ± 0,2
TH0803	IA22	-168	14,92 ± 0,04	72,95 ± 0,25	1,341 ± 0,004	1,253 ± 0,004	0,844 ± 0,009	0,868 ± 0,011	0,64 ± 0,2	0,32 ± 0,2
TH0804	QA25	-206	12,90 ± 0,03	61,61 ± 0,25	1,581 ± 0,006	1,541 ± 0,006	0,988 ± 0,011	1,008 ± 0,023	0,71 ± 0,2	0,30 ± 0,2

Table 4: U content in ppm, isotopic ratios of teeth analyzed in ThI-GH site

Supplementary Note 5 - Magnetostratigraphy

S. Perini and G. Muttoni

Our new analyses, obtained from 118 samples retrieved from 5 correlative sub-sections covering the stratigraphic levels between OH4-SU5 to GH-CCC-SU3 and OH3-A, were integrated with those of Gallotti et al.[10] revealing a more complex scenario for the Matuyama-Brunhes transition (MBT) occurring throughout SU6-3 and strengthening the presence of the Jaramillo subchron within Member OH3 [details of the litho-magnetostratigraphy for Members OH3, OH4, GH-CCC and OH5 of this study and Gallotti et al.[10] are reported in **Fig. 9** and also in **Fig. 1B** of main text]. Reported below are the details of the experimental results used to erect the magnetic polarity stratigraphy.

Thermal demagnetization and paleomagnetic component analysis

Vector end-point thermal demagnetization diagrams of 11 new samples from Member OH3 in section OH3-A (**Fig. 10**) revealed the presence in 7 samples of characteristic component (ChRM) directions isolated between ~300-350°C and ~550-625°C and oriented downward (positive inclinations) with northerly declinations (**Fig. 10; Sup. Tab. 1**). These ChRM directions were integrated with data from Gallotti et al.[10], including ChRM directions oriented southerly-and-up from section OH3-B of Member OH3 that were not considered in Gallotti et al.[10] and that we now place above section OH3-A. Altogether, these ChRM component directions from sections OH3-A and OH3-B, transformed into virtual geomagnetic pole (VGP) latitudes, indicate that the Jaramillo subchron is entirely recorded within Member OH3 (**Fig. 9;** see also **Fig. 1B** of main text).

Vector end-point demagnetization diagrams of 107 samples from OH4-SU6, OH4-SU5, GH-CCCSU4, and GH-CCC-SU3 show the presence of initial A component directions removed between room temperature and minimum-median-maximum values of 250-350-500°C and oriented downward (positive inclinations) with northerly declinations. ChRM component directions, oriented broadly to the south with negative (up-pointing) inclinations or to the north with positive (down-pointing) inclinations, were isolated in 62 samples up to median-maximum values of 670-690°C (data in **Sup. Tab.1)**. In detail, three types of behaviors were observed upon thermal demagnetization:

(i) Northerly-and-down A component directions continuing their trajectories at high temperatures towards the origin of the demagnetization axes into northerly-and-down ChRM component directions (**Fig. 11**).

(ii) Northerly-and-down A component directions turning at high temperatures to southerlyand-up ChRM end-point component directions which then tend to trend (with large noiseto-signal ratio) to the origin of the demagnetization axes (**Fig. 12**).

(iii) Northerly-and-down A component directions moving along great circles towards the southern-and-up quadrant without however reaching stable end-points (**Fig. 13**). A statistical analysis of the intersection density of the 48 great circles clearly indicates a starting region in the northern quadrant (northerly-and-down A component) and great circle trends toward the southern quadrant (unresolved southerly-and-up ChRM component directions; **Fig. 14A**).

We interpret the ubiquitous A component directions as a pervasive chemical overprint of secondary origin acquired during the Brunhes Chron and carried essentially by magnetite (see also rock magnetic properties below), and the southerly-and-up or northerly-and-down ChRM directions of cases (i) and (iii) samples as primary magnetizations acquired in reverse and normal magnetic polarity, respectively, and carried essentially by hematite (see also rock magnetic properties below)**.** Furthermore, we interpret the tendency of case (ii) samples to turn to the southern quadrant after removal of the A component overprint as due to the presence of unresolved, primary component directions of reverse

magnetic polarity, as indicated by great circle analysis. The end-point ChRM directions of cases (i) and (iii) were integrated with ChRM directions from Gallotti et al.[10] and transformed into VGP latitudes for magnetostratigraphy. We also retained the unresolved ChRM reverse polarity tendencies of case (iii) samples (labelled 'x' in **Fig. 2C** of main text).

The total of 97 ChRM directions obtained with least-square analysis from Members OH3, OH4, GH-CCC and OH5 of this study and[10] (S.I. Tab. 2) have maximum angular deviation (MAD) values of 12° ± 7° and appear scattered when analyzed using standard Fisher statistics on an equal-area projection, albeit they display clusters in the northern and southern quadrants, indicative of normal and reverse magnetic polarity, respectively (**Fig. 14B**). A plausible hypothesis for the observed high scatter of the ChRM directions is that hematite grains, while partially aligning with the Earth's magnetic field during deposition and thus recording polarity, were also influenced by interactions with the surfaces of much larger surrounding grains. In coarse-grained sediments such as those investigated in this study, these interactions could have hindered a uniform alignment, unlike in fine-grained sediments characterized by more consistent directional signals.

<u>Rock magnetic properties</u>

Rock magnetic analyses were conducted on samples from the calcarenites of OH4-SU5 and the continental sediments of GH-CCC-SU4. Normalized isothermal remanent magnetization (IRM) backfield acquisition curves up to 1500 m T, analyzed with the Gaussian method of[81], reveal that samples contain large proportions (80-90%) of a low coercivity phase with B1/2 (field required to acquire 50% of the IRM) of 40-60mT, coexisting with smaller proportions (10-20%) of a high coercivity phase with B1/2 of 1000mT (**Fig. 15A**). When coupled with AF decay of the IRM, it appears that ~50% of the IRM is acquired at ~50mT and ~60% of the IRM is removed at ~50mT (**Fig. 15A**). Thermomagnetic experiments performed in Ar atmosphere indicate the presence of sharp decays of magnetization intensity starting above ~500°C (**Fig. 17B**). The first derivatives of these curves (**Fig. 15B inset**) indicate the presence of minima at 550-580°C (f1) marking flexes in magnetization decay, which are taken as evidence of complete unblocking of magnetite (and onset of unblocking of hematite). Sample 4OH493 displays instead two minima (main minimum f2 shown in inset) at temperatures at and above 600°C, indicative of hematite. Stepwise thermal demagnetization of a three-component IRM shows a dominant signal carried by the 0.12T curves with a tendency to drop in intensity in the 540-580°C interval, interpreted as mainly due to magnetite, coexisting with a higher (1.5T) and intermediate (0.4T) coercivity component with maximum unblocking temperatures higher than 600°C, interpreted as hematite (**Fig. 15C**). Hysteresis loops are moderately wasp-waisted whereby they tend to retard closure at high fields between 100 and 500 mT (**Fig. 16A**). This is interpreted as due to the co-occurrence of ferromagnetic (s.l.) minerals with contrasting coercivities such as magnetite and hematite, in agreement with the results outlined above. Low-resolution FORC diagrams show low coercivity asymmetric peaks centered at B_u = 0 and B_c <40 mT, compatible with pseudo-single domain (PSD) magnetite grains (e.g.[82]), coexisting with higher coercivity tails extending to B_c >100, compatible with hematite (**Fig. 16B**). In summary, samples contain a mixture of low coercivity magnetite and high coercivity calcarenites of OH4-SU5 and the continental sediments of GH-CCC-SU4.

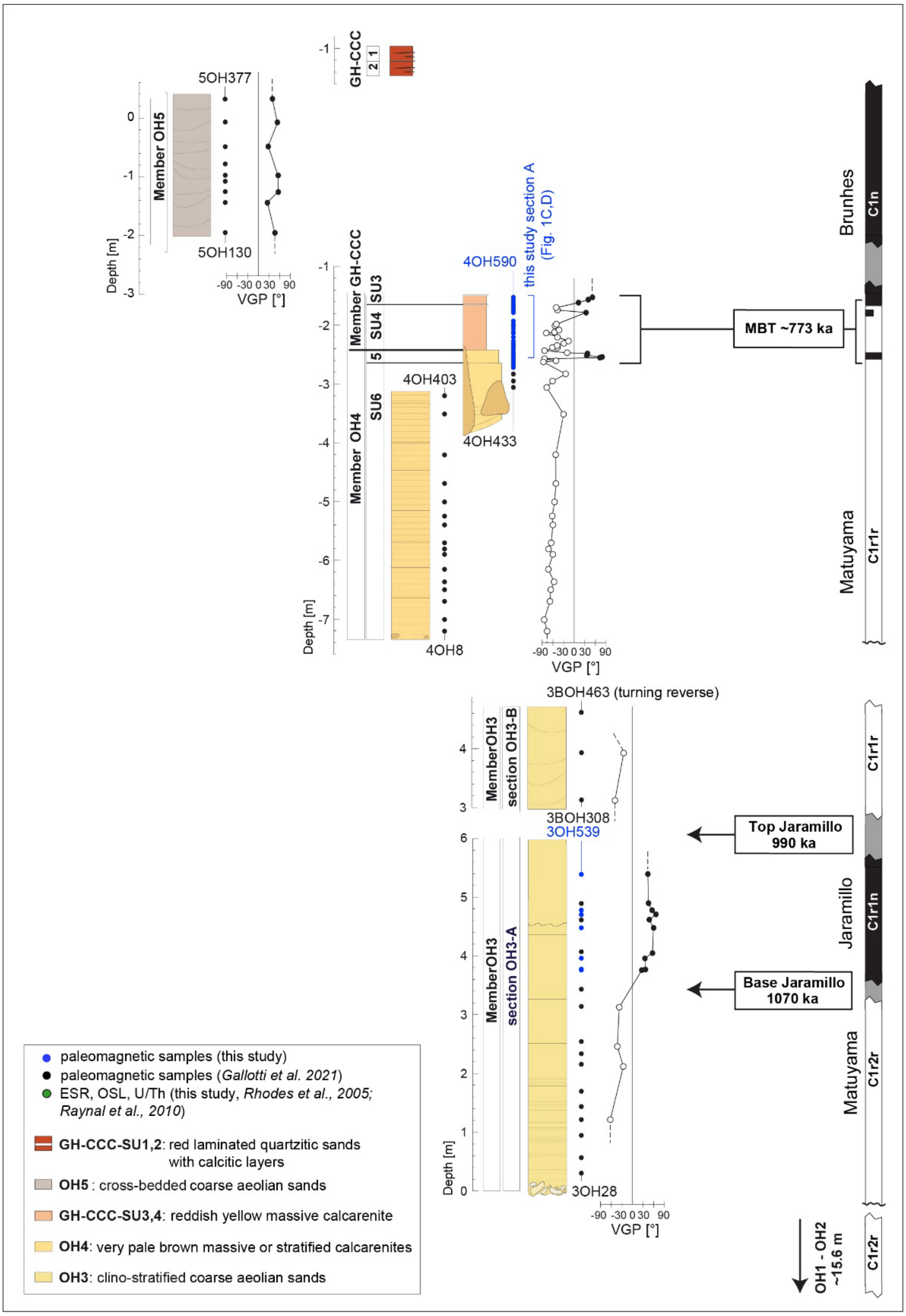

Fig. 9. Litho-Magnetostratigraphy of the Thomas Quarry I sequence with lithostratigraphic members arranged in temporal order according to magnetic polarity stratigraphy. Virtual geomagnetic pole (VGP) data (positive values representing normal polarity, negative values reverse polarity) are from[10] and this study. Only section A of Member OH4 and GH-CCC are reported here (see **Fig. 1D** of main text for details). Note that in this study, we re-evaluated the chronostratigraphic position of section OH3-B of Member OH3, which was not considered in[10] and that we now place above section OH3-A thus obtaining a more continuous record across the Jaramillo subchron. MBT is Matuyama-Brunhes Transition.

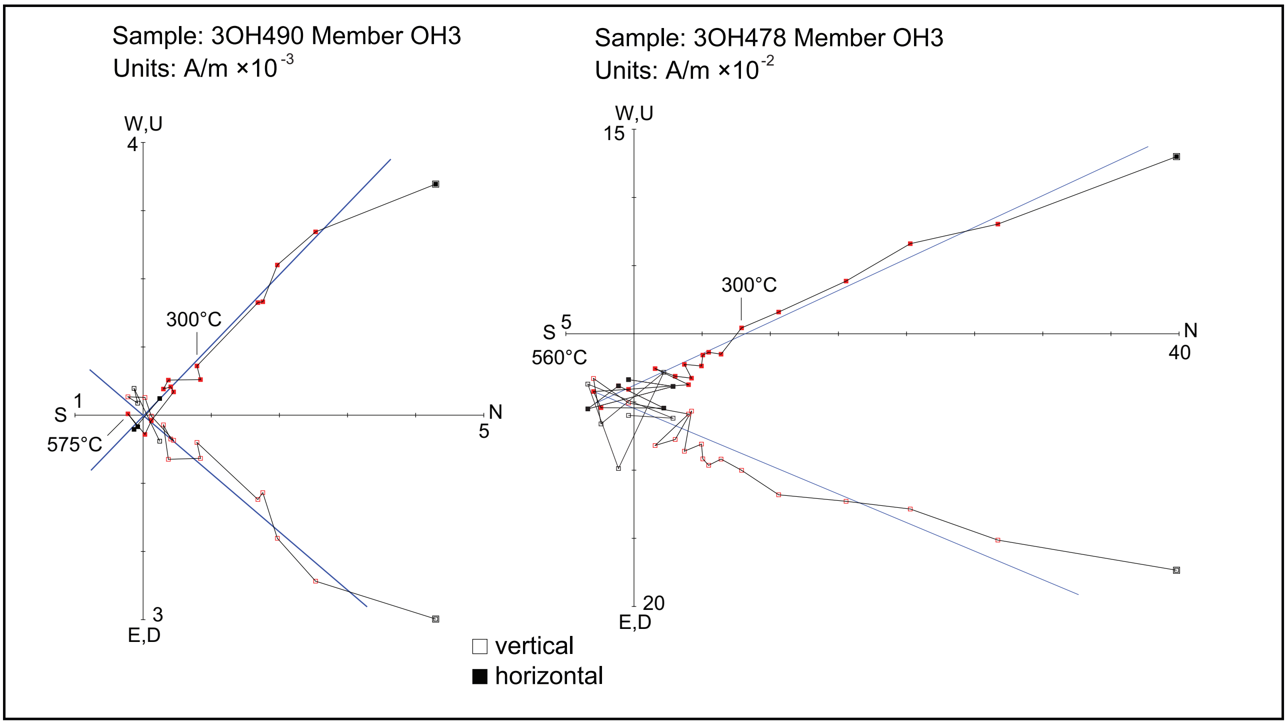

Fig. 10. Vector end-point demagnetization diagrams of representative samples of Member OH3 in section OH3-A. Demagnetization steps highlighted in red are used to extract with the least-square method the characteristic remanent magnetization (ChRM) component directions (blue lines) used to establish magnetic polarity stratigraphy. Filled/open symbols are projections on horizontal/vertical plane. Temperatures are in °C. Made with PuffinPlot[83].

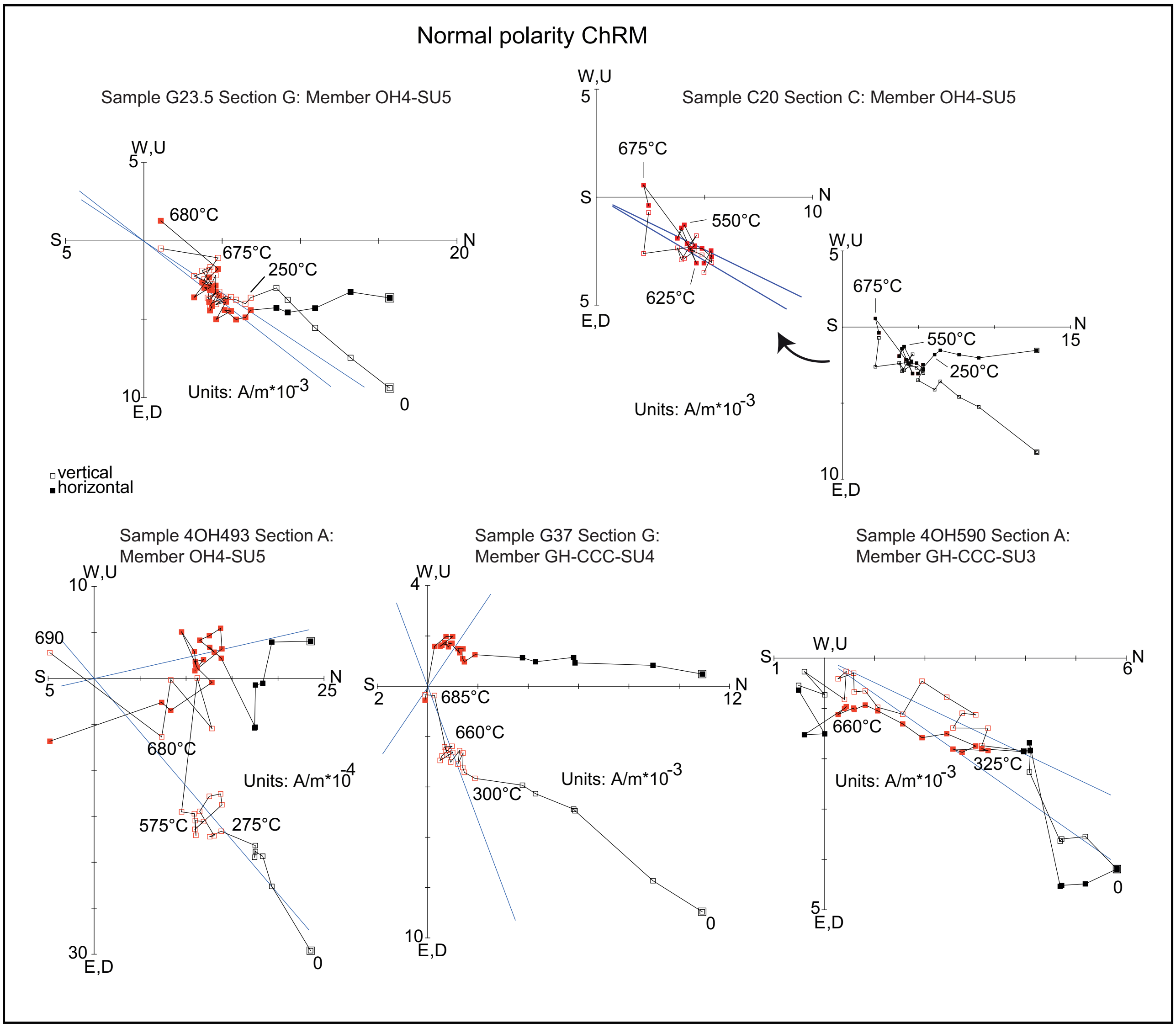

Fig. 11. Vector end-point demagnetization diagrams of representative samples of OH4-SU5, GH-CCC-SU4, and GH-CCC-SU3 interpreted to contain ChRM component directions of normal polarity (case (i) samples). Notes and symbols as in **Fig. 9.** Temperatures are in °C. Made with PuffinPlot[83].

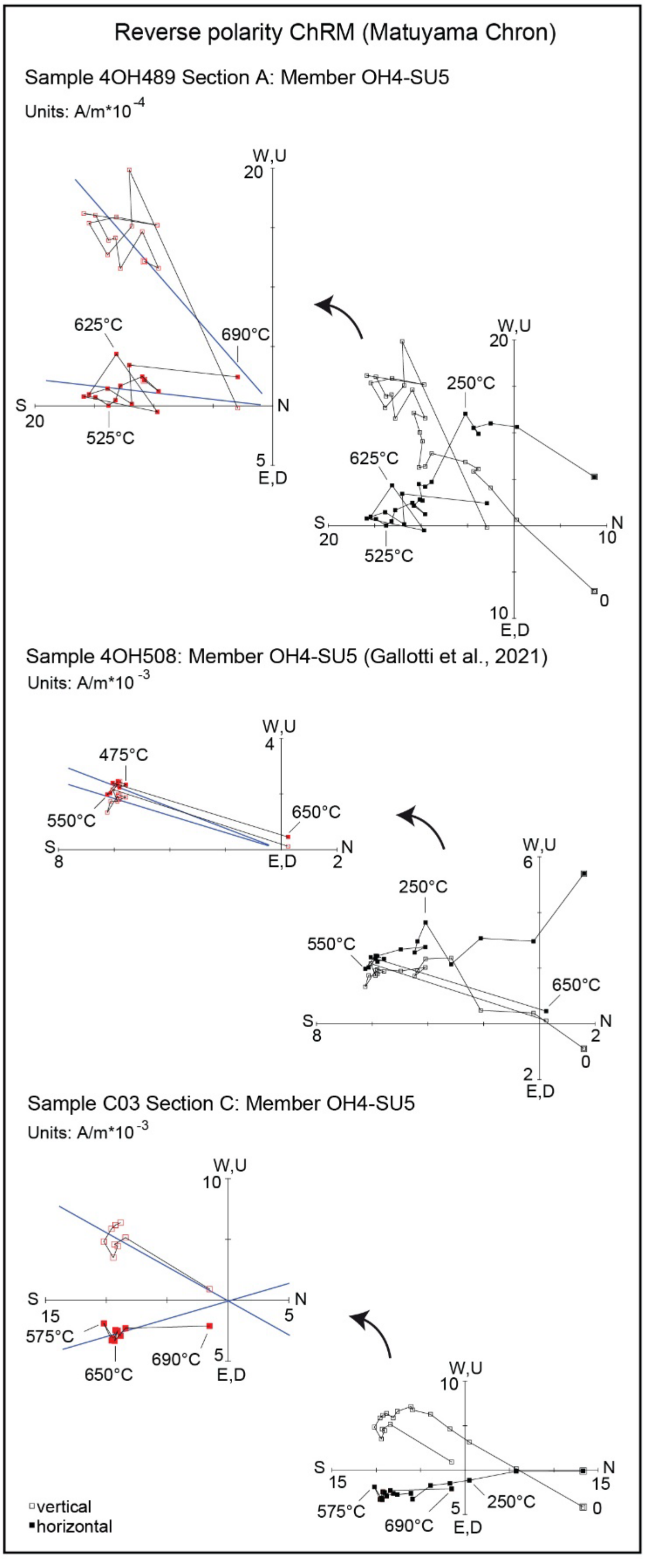

Fig. 12. Vector end-point demagnetization diagrams of representative samples of OH4-SU5 and GH-CCC-SU4 interpreted to contain ChRM component directions of reverse polarity (case (ii) samples). Notes and symbols as in **Fig. 9.** Temperatures are in °C. Made with PuffinPlot[83].

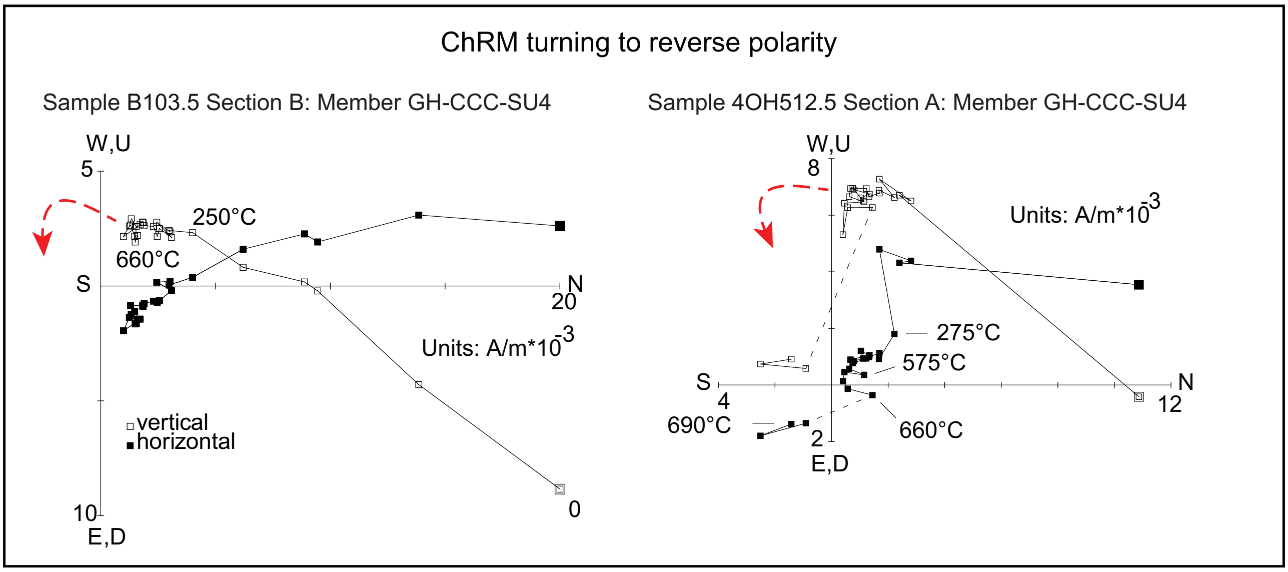

Fig. 13. Vector end-point demagnetization diagrams of representative samples of OH4-SU5 and GH-CCC-SU4 interpreted to contain ChRM component directions showing a tendency to turn to reverse polarity (dashed red arrow), which was assessed qualitatively with great circle analysis (**Fig. 13A**). Notes and symbols as in **Fig. 9.** Temperatures are in °C. Made with PuffinPlot[83].

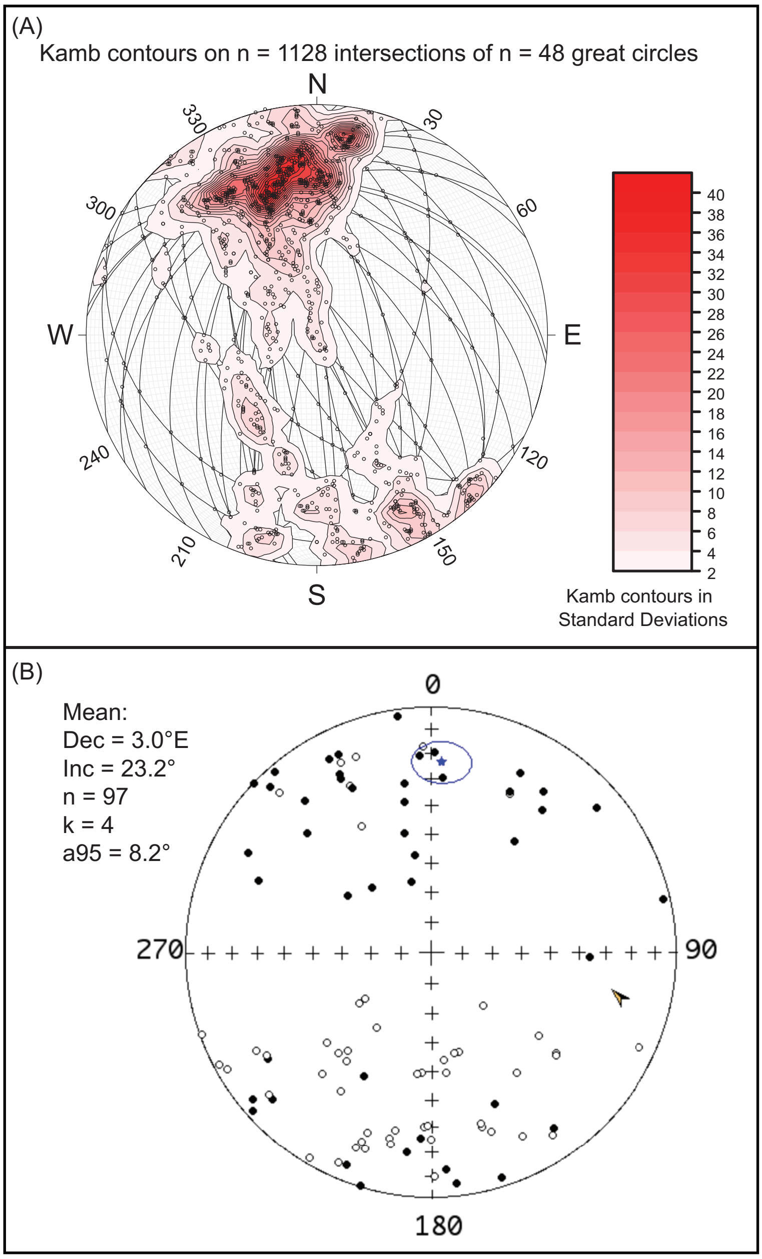

Fig. 14. (A) Great circles of case (iii) samples plotted on an equal-area stereonet with Kamb density contouring of the derived 1128 intersections. Demagnetization trajectories of case (iii) samples tend to move on great circles from the northern quadrant towards the southern quadrant (unresolved ChRM component of reverse polarity). (B) Equal-area projection of the characteristic remanent magnetization (ChRM) component directions for samples of this study and[10] for Members OH3, OH4, GH-CCC, and OH5. Closed symbols are projection onto the lower hemisphere while open symbols onto the upper hemisphere. The star represents the Fisher mean direction and associated cones of 95% confidence.

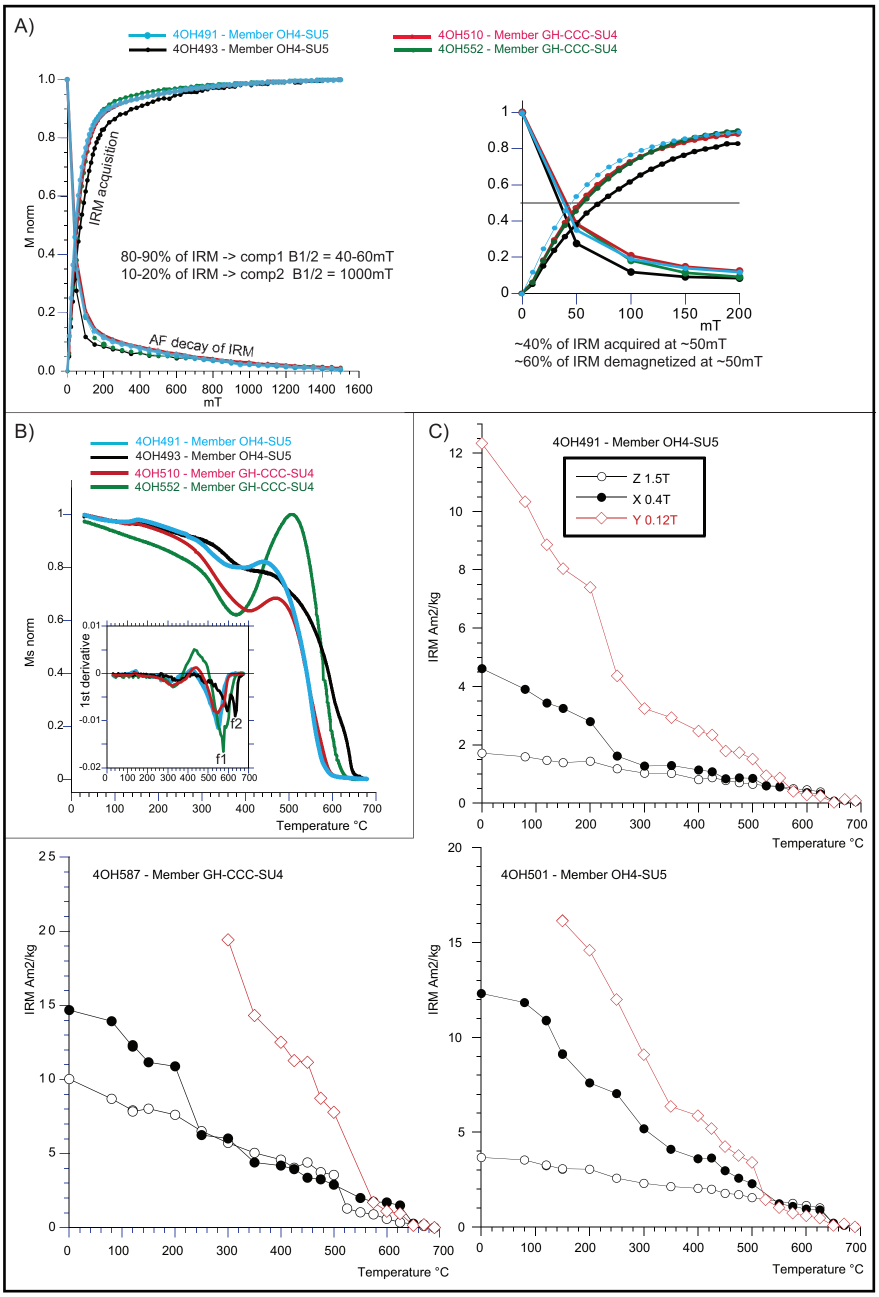

Fig. 15. (A) normalized stepwise acquisition and alternating field (AF) decay of an isothermal remanent magnetization (IRM), (B) thermomagnetic decay of a magnetization induced at 1T in Ar atmosphere, (C) stepwise thermal decay of a three component isothermal remanent magnetization (IRM) acquired in fields of 1.5T, 0.4T and 0.12T.

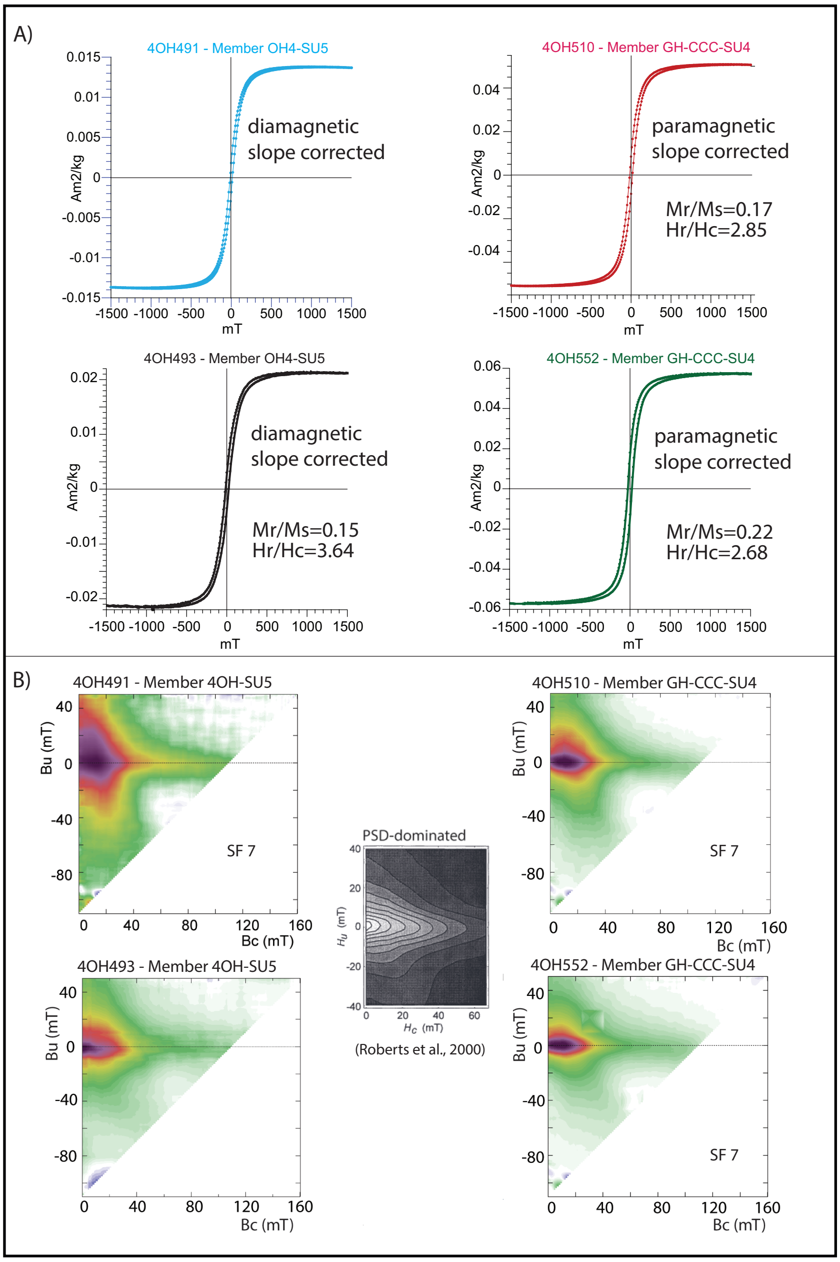

Fig. 16. (A) Hysteresis loops and (B) low-resolution (n=76) FORC diagrams of samples from the calcarenites of OH4-SU5 and the continental sediments of unit GH-CCC-SU4. At the center of panel (B) is an example taken from[83] of a FORC attributed to PSD grains.

Supplementary Note 6 – Adult mandibular morphology, Metrical Comparisons and Geometric Morphometric Analyses

Sarah Freidline and Inga Bergmann

ThI-GH-10717 Mandible: Morphological Description

ThI-GH-10717 is a complete mandible that belonged to an adult individual. The mandible has minimal damage: the tip of the left coronoid process is fractured, the left condyle is broken preserving the medial third, and the anterior alveolar portion of the mandible is broken labially and lingually from the left canine to the right first incisor. The overall size and shape of the mandible is small and gracile, lacking robust superstructures. The labial and lingual surfaces are generally smooth in topography.

Symphysis: Labially, ThI-GH-10717 has a receding symphysis and a small *mentum osseum* conforming to category 2[84]. Lingually, the internal morphology of the symphysis is relatively smooth in its topography. The alveolar planum is nearly vertically oriented and it has slight alveolar prominence. It lacks both a superior and inferior transverse torus and expresses a subtle genioglossal fossa. The base of the symphysis has a pronounced *submental incisura*, a small anterior marginal tubercle at the level of P_4, a slight depression marking a downward facing digastric fossa, and an interdigastric spine is visible from the anterior perspective.

Corpus: The mental foramen is at the level of P_4. Labially the topography is smooth, with a subtle superior and inferior marginal torus, and a shallow intertoral sulcus. The lateral prominence is weak with the maximum expression at the level of M_2 the extramolar sulcus is narrow, and it lacks a retromolar space. The M_3 is partially covered by the ramus in lateral view. Lingually, and the mylohyoid line is faint, runs parallel to the alveolar margin, and is low, at the level of M3. It has a moderate hallowing of the subalveolar fossa.

Ramus: Labially, ThI-GH-10717 has a shallow masseteric fossa. Lingually, it has a shallow pterygoid fossa, an oblique (posteriorly angled) internal coronoid pillar, the endocoronoid crest is obliquely orientated, and the planum triangulare is deep and well-defined. The opening of the mandibular foramen is circular. It has a small medial pterygoid tubercle on the internal surface of the left ramus. The gonial profile is rounded and slightly everted laterally. The mandibular notch is symmetrical.

ThI-GH-1 Mandible: Morphological Description

ThI-GH-1 is a left hemimandible that is broken just lateral to the symphysis at the level of the canine. It is missing the superior portion of the ramus that includes both the coronoid process and the mandibular condyle. It is larger and slightly more robust than ThI-GH-10717, and like ThI-GH-10717, its corpus is low but it has a more pronounced pre-angular notch.

Corpus: There is no evidence of a mental foramen or anterior marginal tubercle on the preserved corpus. If present, it would have been anterior to P_4. Labially, it has a subtle superior and inferior marginal torus, and a shallow intertoral sulcus. It has a more pronounced and posterior lateral prominence at the level of M_2-M_3, the extramolar sulcus is narrow, and it lacks a retromolar space. The M_3 is uncovered in relation to the ramus and visible in lateral view. Lingually, the mylohyoid line is faint and the trajectory is oblique in relation to the alveolar margin. It has a moderate hollowing of the subalveolar fossa.

Ramus: Labially, ThI-GH-1 has a deeper relief of the masseteric fossa. Lingually, it has a shallow pterygoid fossa, the endocoronoid crest is obliquely orientated, and the opening of the mandibular foramen is oval. It has a small medial pterygoid tubercle on the internal surface as well as tuberculum

pterygoideum inferius. The gonial profile is less rounded and more truncated, slightly everted laterally, and more rugous internally.

ThI-GH-10717 and ThI-GH-1 Mandibles: Morphological Comparisons

Morphological comparisons of the ThI-GH mandibles to other fossil hominin groups are provided in Table S6. ThI-GH-1 differs from ThI-GH-10717 in having a stronger and slightly more posterior lateral prominence that is at the level of M_2-M_3, similar to Tighennif 3. A more posterior prominence, at the level of M_3, is the derived condition found in European Middle Pleistocene hominins (MPh) and Neanderthals. In ThI-GH-10717 and the *H. antecessor* mandibles, the M_3 is partially covered by the ramus (as seen in *norma lateralis*). A M_3 that is covered by the ramus is the ancestral state; where as an uncovered M_3 occurs in Neanderthals. In ThI-GH-1, it is completely uncovered. The ThI-GH mandibles lack a retromolar space and the inclination posterior to the M_3 is oblique, like *H. antecessor*, Tighennif, early *Homo* and some European MP hominins. The mylohyoid line on ThI-GH-10717 is archaic, like most *Homo* species except Neanderthals, running low (at the level of M_3) and parallel to the alveolar margin. Whereas, on ThI-GH-1 the mylohyoid line is more similar to Neanderthals, running oblique. The masseteric fossa in ThI-GH-10717 is shallower than ThI-GH-1, and lingually both mandibles have a shallow relief of the pterygoid fossa. Both mandibles have a similarly thick gonial profile that is slightly everted outward. The profile of ThI-GH-1 is less rounded than ThI-GH-10717 and approaching the truncated Neanderthal condition. The intersection between the mandibular notch and condyle is lateral rather than medial in ThI-GH-10717, unlike in Neanderthals.

ThI-GH-10717 shares all features in Table S6 with *H. antecessor* (ATD6-96, ATD6-5, ATD6-113), except the position of the anterior marginal tubercle. This feature is not present in ATD6-96, the only TD6 mandible preserving the region of the anterior marginal tubercle. In ThI-GH-10717, the anterior marginal tubercle is located more posteriorly at the level P_4. A more posterior location occurs in European MPh (*H. heidelbergensis*) and Neanderthals, although an anterior location has also been identified on specimens attributed to these groups. Many of the features that ThI-GH-10717 and *H. antecessor* share are ancestral including: an anterior position of the mental foramen (at the level of P_3-P_4), lateral prominence at M_2, a low mylohyoid line (relative to M_3) that runs parallel to the alveolar margin, and the mandibular notch intersects the condyle laterally. The Th1-GH and TD6 mandibles also have a shallow pterygoid fossa, an ancestral featured shared with early *Homo* and the Tighennif mandibles. Several features that ThI-GH-10717 and *H. antecessor* share are derived relative to *H. habilis* and are variously present in *H. ergaster*, *H. erectus*, the Tighennif mandibles, and the European MPhs: including a partially covered M_3, an obliquely inclined retromolar area, a shallow relief of the masseteric fossa, a regular gonial profile, a moderately hollowed subalveolar fossa, and a lack of alveolar prominence. ThI-GH-1, on the other hand, shares more features with Neanderthals than ThI-GH-10717 including an uncovered M_3 in relation to the ramus, a higher position of the mylohyoid line in relation to the alveolar margin that is nearly obliquely oriented, and a more truncated shape of the gonial profile.

Mandibular Features	State A	State B	State C
1. Position of mental foramen	P3-P4	P4-M1	M1
2. Position of the anterior marginal tubercle	P3-P4	P4-M1	M1
3. Position of the lateral prominence	M2	M2-M3	M3
4. M3 in relation to ramus (retromolar space)	Covert	Partially covert	Uncover
5. Inclination of the retromolar area	Vertical	Oblique	Horizontal
6. Position of the mylohyoid line in relation to alveolar margin at M3 level	Low	Medium	High
7. Trajectory of mylohyoid line in relation to alveolar margin	Parallel	Intermediate	Diagonal
8. Relief of masseteric fossa	Deep	Shallow	Flat
9. Relief of pterygoid fossa	Shallow	Deep	--
10. Gonion profile	Expanded	Regular	Truncated
11. Intersection between mandibular notch and condyle	Lateral	Medial	--
12. Posterior subalveolar fossa	Shallow	Moderately hollowed	Deep
13. Alveolar prominence	Present	Absent	--

Table 5. State of mandibular features analyzed in Table 6 following[85-87].

	Habilis[a]	Ergaster[b]	Erectus (Java)[c]	Erectus (China)[d]	Tighennif (1-3)	ThI-GH-10717	ThI-GH-1	TD6	EMPh[e]	Neanderthal[f]
1	A	A, B	A	A, B	A	A	--	A	B,C	C
2	A, O	A, O	A	A, O	A	B	O	O	A, B, C	A, B, C
3	A	C	A	B	A, B	A	B	A	C	C
4	A	A, B	A, B	B, C	B	B	C	B	B, C	C
5	A	A, B	A, B	A, B	B	B	B	B	B, C	C
6	A, B	A, B	A, B	A	A	A	B	A	A	A, B, C
7	A	A, B	A	A	A, B	A	B	A	A, B, C	B, C
8	A	A, B, C	A	A	A, B	B	A	B	A, B, C	B, C
9	--	A	A	A	A	A	A	A	A, B	B
10	--	A, B	--	A, B	A, B	B	B	B	B, C	C
11	--	A	--	A	A	A	--	A	A, B	B
12	A	A	A	C	B	B	B	B	B, C	C
13	A	A	A	A, B	A	B	--	B	A, B	B

[a]*Homo habilis* sample: KNM-ER 1501 and 1805, OH 7, 13, 37
[b]*Homo ergaster* sample: KNM-ER 730, 731, 992; KNM-BK 67, 8518; OH 22, 23, 51
[c]*Homo erectus* (Java) sample: Sangiran 1B, 5, 8, 9
[d]*Homo erectus* (China) sample: Zhoukoudian, Lantian, Hexian, and PA86
[e]European Middle Pleistocene hominin sample: Arago II, XIII; Mauer; Montmaurin; Atapuerca-SH
[f]Neanderthal sample: Krapina J, G, H, E, D; Vindija 20, 22, 23; La Chaise BD; Ehringsdorf F; Amud 1; Tabun C1 and C2; Spy 1; Hortus 4; La Quina H9; Regourdou 1; Circeo II; St. Cesaire; Kebara; La Ferrassie; Shanidar

Table 6. State of the mandibular features listed in Table S5 modified from[85]. All data from[85] except Th-GH-10717 and Th-GH-1. O = absent

ThI-GH-10717 and ThI-GH-1 Mandibles: Metrical Comparisons

Metrical comparisons of the ThI-GH mandibles to other fossil hominin groups are provided in Table S7 and S8. ThI-GH-10717 corpus height at M_1 (22.2 mm) is shorter than ThI-GH-1 (26.8 mm), the Tighennif mandibles (33.4-37.5 mm), and the *H. antecessor* mandibles (26.7-31.0 mm). This measurement on ThI-GH-1 is similar to ATD6-5 (26.7 mm). The average corpus height at M_1 for the ThI-GH mandibles (24.5 mm) is smaller than all comparative samples, less than *H. antecessor* (28.7 mm) and *H. habilis* (29.3 mm). Whereas, the tallest corpora belong to *H. erectus* from Java, Sidi Abderramane, and the Tighennif mandibles. Similarly, ThI-GH-10717 corpus breadth at M_1 (13.9 mm) is smaller than ThI-GH-1 (14.5 mm), the Tighennif mandibles (16.6-19.8 mm), and the *H. antecessor* mandibles (16.3-19.0 mm). The average corpus breadth at M_1 for the ThI-GH Quarry mandibles (14.2 mm) is similar in size to Upper Paleolithic *H. sapiens* (14.3 mm) and smaller than all other comparative samples; the next smallest being Neanderthals at 15.3 mm.

ThI-GH-10717 has a robust mandible at M_1 (62.5 mm), considerably more robust than ThI-GH-1 (53.2 mm) and slightly larger than the most robust mandible from Gran Dolina, ATD6-113 (61.3 mm). In this value, ThI-GH-1 is more similar to the mandibles from Tighennif (49.7-54.7 mm). The average robusticity value for the ThI-GH mandibles (57.9 mm) is slightly less than the *H. antecessor* mandibles (60.2) and similar to *H. erectus* from China (58.0 mm).

ThI-GH-10717 ramus height (56.3 mm) and breadth (37.3 mm) is slightly smaller than ATD6-96 (60.0 and 40.6 mm, respectively) and considerably smaller than the Tighennif mandibles.

Measurement	ThI-GH-10717	ThI-GH-1	Tig1	Tig2	Tig3	ATD6-96	ATD6-5	ATD6-113
**Symphyseal Height**	[24]	--	36.0	33.3	37.5	--	--	--
**Corpus Height at M_1**	22.2	26.8	36.2	33.4	37.0	28.5	26.7	31.0
**Corpus Breadth at M_1**	13.9	14.5	19.8	16.6	19.5	16.6	16.3	19.0
**Corpus Robustness at M_1**	62.5	53.2	54.7	49.7	52.7	58.2	61.0	61.3
**Ramus breadth**	37.3	35.1	--	45.8	47.3	40.6	--	--
**Ramus height**	56.26	--	--	78.5	85.6	60.0	--	--

Table 7. Mandibular measurements (mm) of the ThI-GH. mandibles compared to the Early Pleistocene mandibles from Tighennif (Tig), Algeria, and Gran Dolina-TD6 (ATD6), Spain, mandibles. Measurement data on the Tighennif and TD6 mandibles is from[85]. Numbers in brackets were estimated. Corpus robustness is measured as Breadth x Height * 100.

**Species**	**Breadth (n)**	**Height (n)**	**Robustness (n)**
Homo habilis	19.7 +/- 2.3 (5)	29.3 +/- 2.2 (4)	64.5 +/- 5.3 (4)
H. ergaster	19.8 ± 1.4 (8)	31.2 ± 2.8 (8)	63.8 ± 5.0 (8)
H. erectus (Java)	21.3 ± 3.9 (5)	38.8 ± 5.3 (5)	54.9 ± 5.8 (5)
H. erectus (China)	16.5 ± 1.8 (7)	28.6 ± 3.3 (7)	58.0 ± 5.6 (7)
Tighennif	18.6 (3)	35.5 (3)	52.4 (3)
Sidi Abderrahmane	17.0	35.8	47.5
**ThI-GH**	**14.2**	**24.5**	**57.9**
H. antecessor	17.3 (3)	28.7 (3)	60.2 (3)
H. heidelbergensis	16.4 ± 1.7 (16)	30.8 ± 3.4 (16)	53.4 ± 5.1 (16)
H. neanderthalensis	15.3 ± 1.7 (21)	32.1 ± 3.3 (21)	47.9 ± 5.1 (21)
Early *H. sapiens**	17.11 ± 2.57 (11)	32.81 ± 5.64 (10)	52.1
Upper Paleolithic *H. sapiens***	14.25 ± 1.57 (26)	29.51 ± 2.19 (29)	48.3

* The early *H. sapiens* specimens include Dar es-Soltan II-H5, El Harhoura 1, Dire Dawa, Klasies River (KRM 13400, KRM 14695, KRM 16424, KRM 21776, KRM 41815), Qafzeh (9, 25), Skhul (IV, V), Tabun C2 and Contrebandiers 1.

** The Upper Palaeolithic and Epipalaeolithic sample includes individuals from Abri Pataud 1, Arene Candide (2, 18), Asselar, Barma del Caviglione, Chancelade, Cro Magnon (1, 3), Dar es-Soltan (II-H2, II-H3), Dolni Věstonice (3, 13, 14, 15, 16), El Mirón, Grotte des Enfants 4, Hayonim (8, 17, 19, 20, 25, 27, 29 and 29a), Isturitz (106 and 115), Le Roc (1, 2), Minat 1, Moh Khiew, Muierii 1, Nahal Oren (6, 8, 14, 18), Nazlet Khater 2, Oase 1, Oberkassel (1, 2), Ohalo II (1, 2), Pavlov 1, Předmostí (3, 21), Sunghir (1, 6), Villabruna 1 and Zhoukoudian Upper Cave (101, 104, 108).

Table 8. Mandibular measurements (mm) of the mandibular body at M_1 in the ThI-GH mandibles compared to *Homo* species and specimens. The comparative data are from[85,88]. See Supplementary Table 2 for sample composition of all comparative data except early *H. sapiens* and Upper Paleolithic *H. sapiens*.

ThI-GH-10717 and ThI-GH-1 Mandibles: Geometric Morphometric Analyses

The overall size and shape of ThI-GH-10717 is small and gracile, lacking robust superstructures. Its corpus shape is archaic, plotting with *H. erectus* and African Early Pleistocene hominins (Fig. S17); whereas its ramus shape is more derived, plotting in between African Early Pleistocene hominin and European Middle Pleistocene hominins (Figures S18 and S19). ThI-GH-10717 consistently shows shape similarities to the European Middle Pleistocene mandible from Montmaurin (Supplementary Tables 3 and 4), as well as fossil African *H. sapiens* from Border Cave (Border Cave 5), Mumbwa (Mumbwa 3), and Contrebandiers Cave (Contrebandiers 1).

ThI-GH-1 is a larger mandible and its mandibular shape is more similar to European Middle Pleistocene hominins and Neanderthals (ED Fig. 5). Its centroid size plots at the upper end of *H. erectus* variation, exceeding the size of all other fossils in the sample, except the Late Pleistocene fossil Olduvai 1 (Fig. S20). Its nearest neighbors according to Procrustes distance are early Holocene (Lothagam KNM-ER5306) and Late Pleistocene *H. sapiens* (Taforalt XXVII XXV) from Africa (Suppl. Tab. 4).

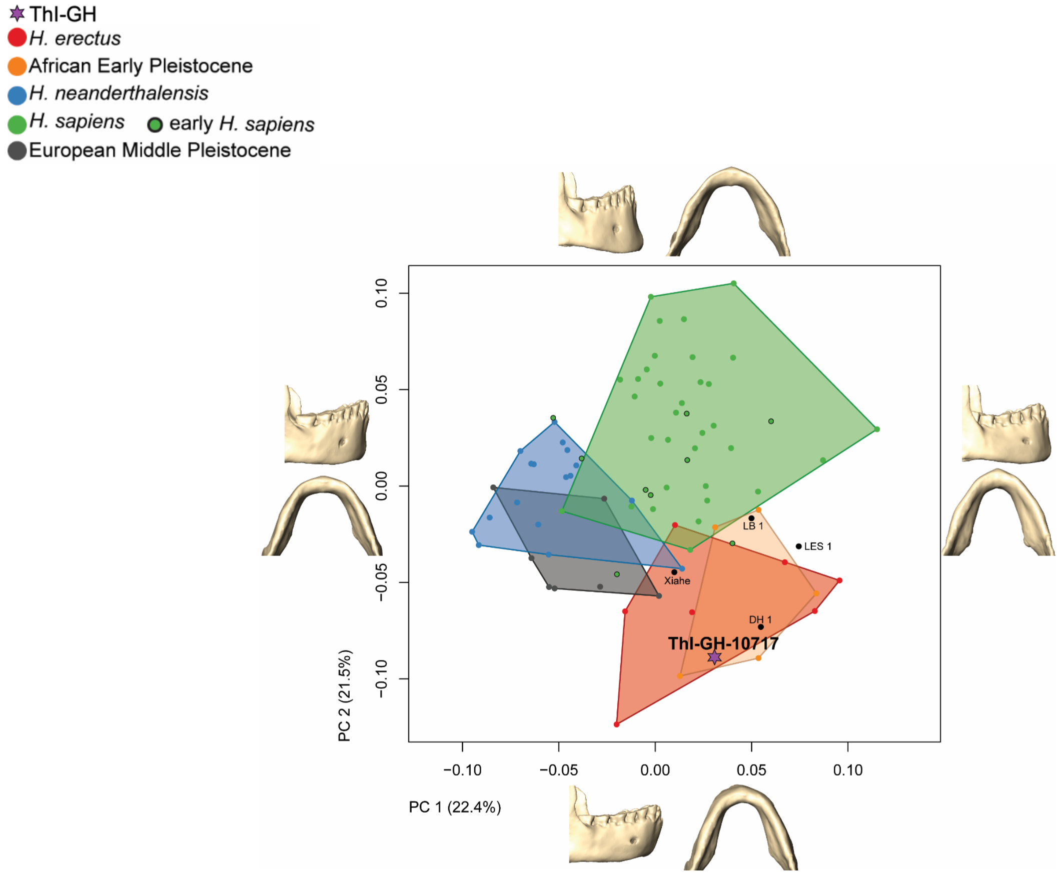

Figure 17. PCA in shape space using the corpus dataset: PC 1 and PC 2. ThI-GH-10717 plots within both the *H. erectus* and the African Early Pleistocene (AEP) hulls; Liang Bua 1 (LB 1), Lesedi 1 (LES 1), and Dinaledi Hominin 1 (DH 1). Shape changes associated with PC 1 (22.4 % shape variance) mainly include symphysis height and corpus breadth and robusticity. Neanderthals and European Middle Pleistocene hominins have a taller symphysis, and a thinner and broader corpus compared to ThI-GH-10717. Shape changes associated with PC 2 (21.5 % shape variance) include corpus height and symphyseal shape. AEP hominins, *H. erectus* and ThI-GH-10717 have a low corpus and a receding symphysis that is distinct from the other hominin groups, especially *H. sapiens*.

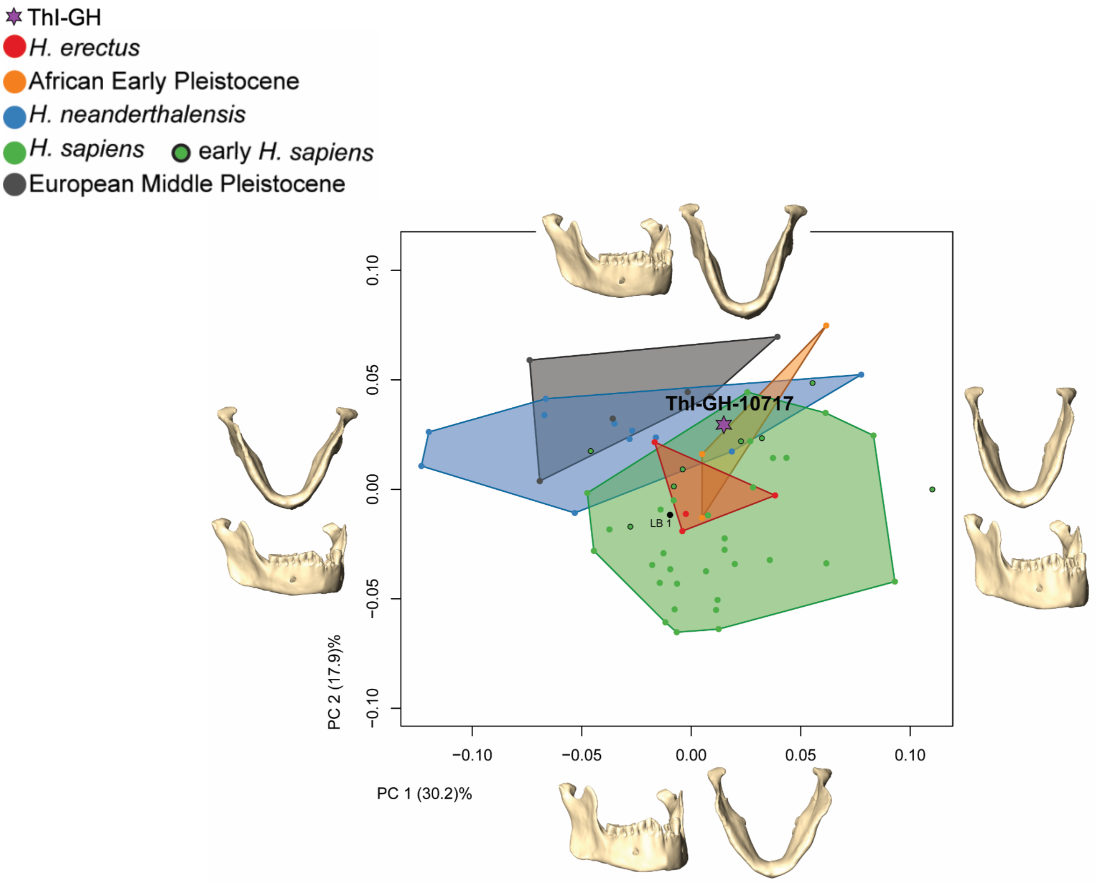

Figure 18. **PCA in shape space using the mandibular dataset: PC 1 and PC 2**. ThI-GH-10717 plots within both Neanderthal range of variation, at the edge of *H. sapiens* variation, and just outside of the African Early Pleistocene hull; Liang Bua 1 (LB 1). Shape changes associated with PC 1 (30.2 % shape variance) mainly include corpus and ramus height and mandibular breadth. Shape changes associated with PC 2 (17.9 % shape variance) include gonial shape, angle of symphysis, and width of the ramus. ThI-GH-10717 resembles more the Neanderthal condition in having a less everted gonial shape, vertical symphysis, and wide ramus.

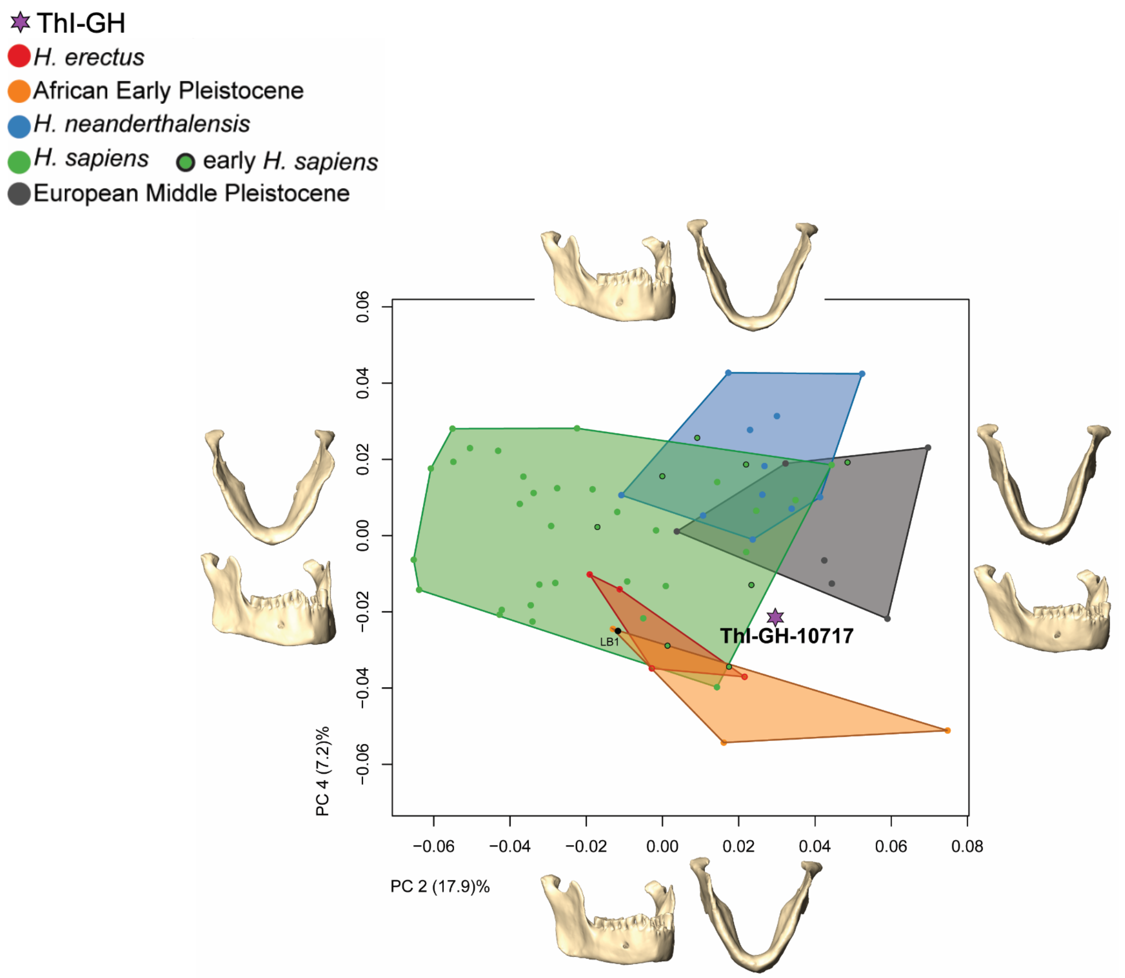

Figure 19. **PCA in shape space using the mandibular dataset: PC 2 and PC 4.** ThI-GH-10717 plots in between African Early Pleistocene (AEP) and European Middle Pleistocene shape variation and just outside of the *H. sapiens* hull; Liang Bua 1 (LB1). Shape changes associated with PC 2 (17.9 % shape variance) mainly include gonial shape, angle of symphysis, and width of the ramus. ThI-GH-10717 is distinct from *H. sapiens* by having a less everted gonial shape, vertical symphysis, and wide ramus. Shape changes associated with PC 4 (7.2 % shape variance) include corpus height and breadth and ramus size. AEP hominins and Neanderthals are distinct in these features, with AEP expressing a shorter and thicker corpus, combined with a wider ramus. ThI-GH-10717 shows an intermediate expression in these features.

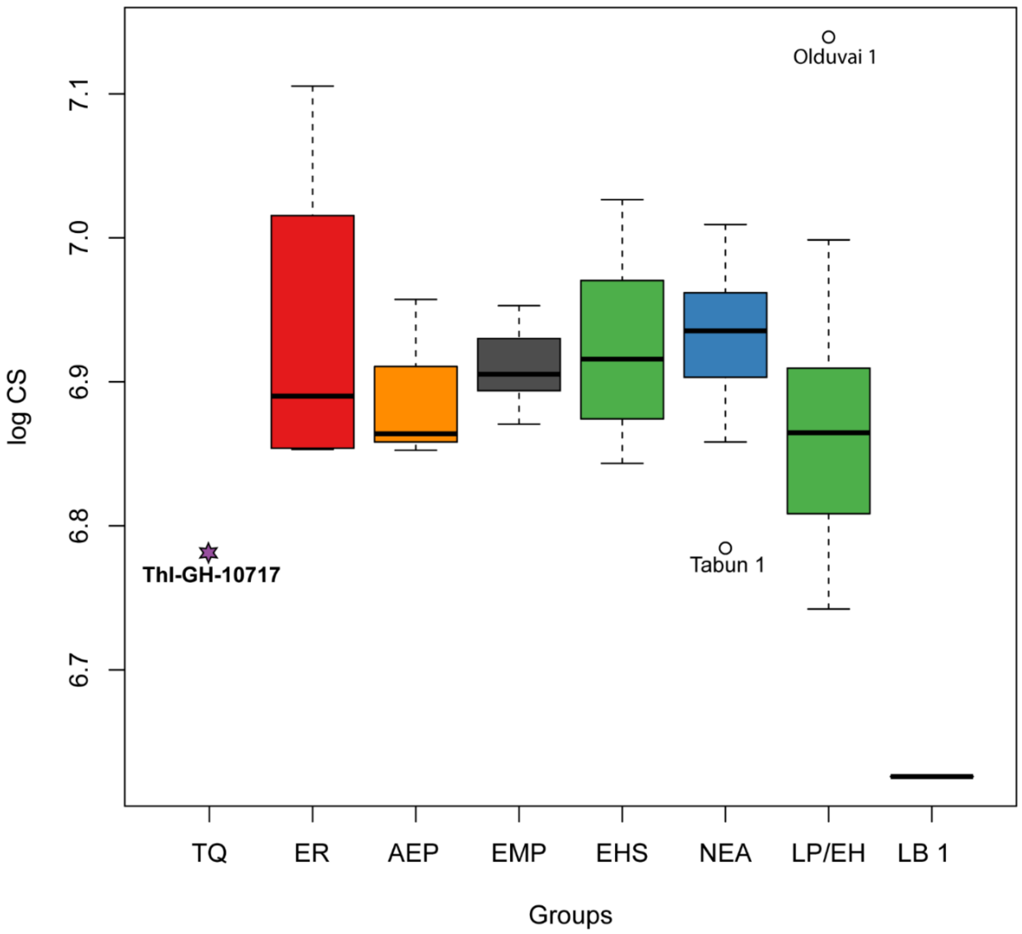

Figure 20. ThI-GH-10717 centroid size using the mandibular dataset. Box plot depicting log centroid size for each group (*H. erectus* [ER], African Early Pleistocene [AEP], European Middle Pleistocene [EMP], early *H. sapiens* [EHS], Neanderthals [NEA], Late Pleistocene and Early Holocene *H. sapiens* [LP/EH], *H. floresiensis* Liang Bua 1 [LB1]). Horizontal lines represent the median of each group. Boxes show the interquartile range (IQR, 25th to 75th percentile). Whiskers extend to 1.5 times IQR. Outliers are represented by circles. ThI-GH-10717 and Tabun C1 are smaller than all fossils in the sample except Liang Bua 1 and several LP/EH individuals.

Supplementary Note 7 - Developmental stage of ThI-GH-10978

Adeline Le Cabec

ThI-GH-10978 is a fragmentary right hemi-mandible (**Fig. 21**) preserving a partial mixed dentition involving the first and the second deciduous molars, and the germs of two unerupted permanent teeth, among which the third premolar and the first molar (**Figs 22-23**). The specimen has undergone extensive taphonomic damage, altering the bone inner structure, as well as the preservation of the dentine (broken) of most of the teeth (**Fig. 24**).

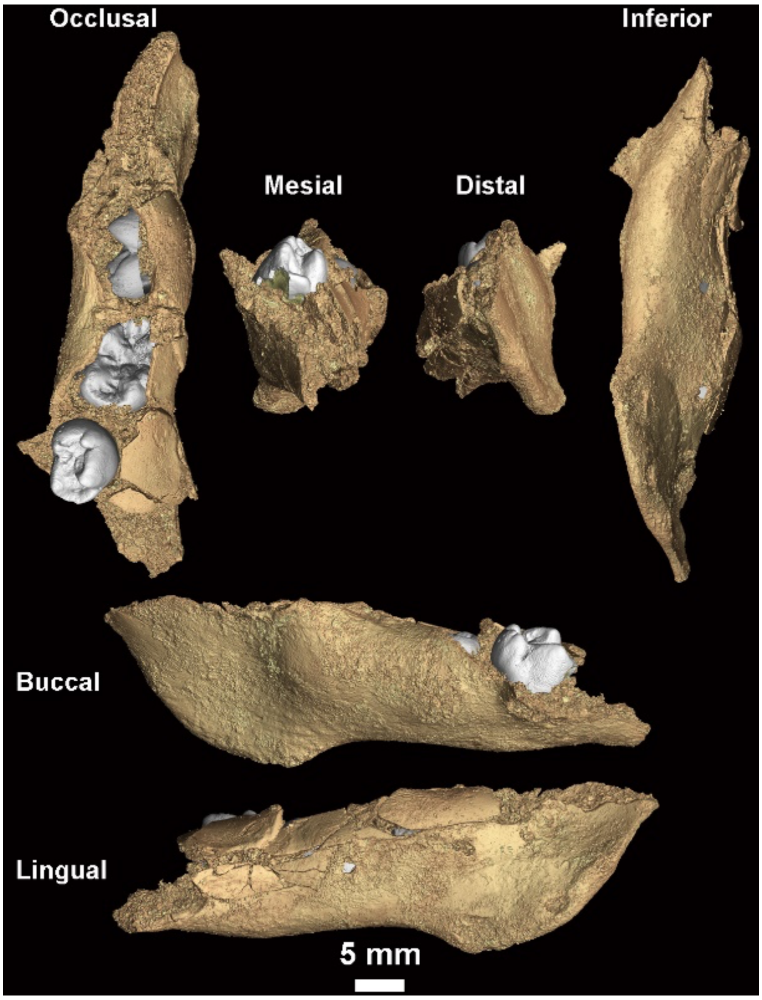

Fig. 21 – 3D models of the segmented bone and tooth tissues of the ThI-GH-10978 specimen.

Virtual 2D sections (**Fig. 22**) were registered in the developmental plane for each tooth (the LRM1 is shown for information), to score their stage of calcification (e.g., [89]). Virtual 2D sections and 3D models were performed in VG Studio MAX 3.5 (Volume Graphics, Heidelberg, Germany).

LRdm1 Although taphonomy may have modified the anatomical position of the mandibular right deciduous first molar, it must have reached occlusal emergence although it appears still unworn. The LRdm1 crown is complete, and its roots were certainly already well-developed although they are broken under the cervix (**Figs. 22, 24**). The protoconid shows a pitted aspect, as well as marked enamel defects visible on the hypoconid (**Fig. 23**).

LRdm2 The mandibular right deciduous second molar has just completed its crown, and some fine spicules of root dentine must have developed and yet must have been broken away (**Fig. 22**). The metaconid shows a deep enamel defect reaching the enamel-dentine junction. Similarly to the LRdm1, its protoconid shows some strong enamel pitting (**Fig. 23**).

LRP3 Only one of the cusps of the mandibular right permanent first premolar could be found in the scan, and it has barely formed its cuspal enamel (**Figs. 22-23**). Debris of teeth involving enamel and dentine still connected may be found in the mandibular body but it remains impossible to know to which tooth they belonged.

LRM1 The mandibular right permanent first molar has completed its occlusal outline and all five cusps are coalesced (**Figs 22-23**). About half of the crown is formed. No obvious developmental defect could be identified in its cuspal enamel, which is however highly cracked due to its stage of mineralization (**Fig. 22**).

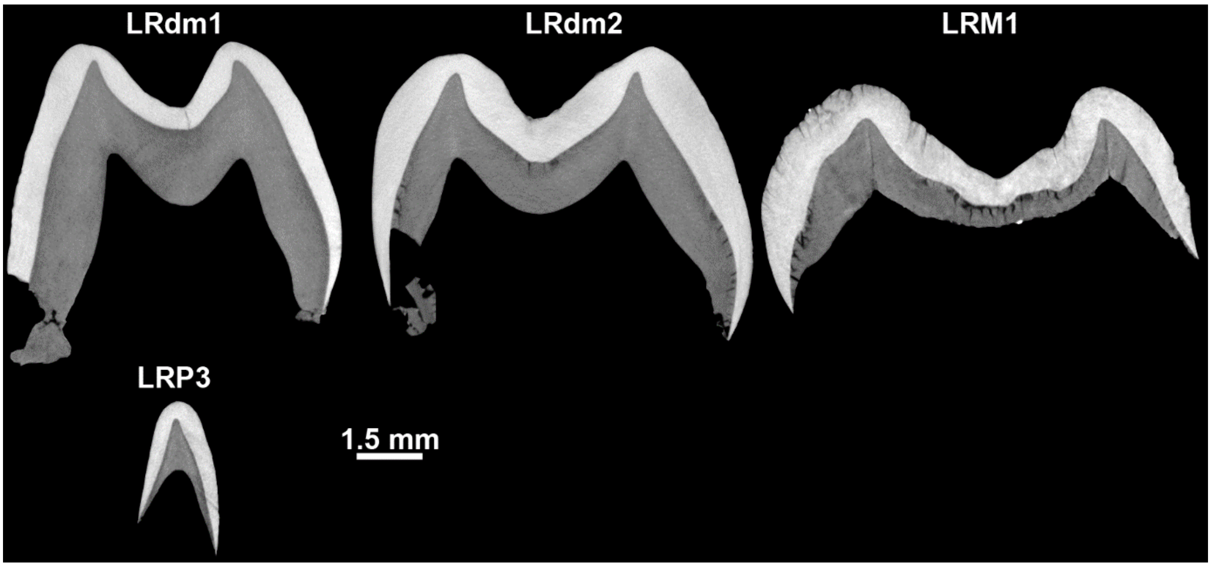

Fig. 22 – Virtual developmental 2D sections through the ThI-GH-10978 deciduous and permanent teeth.

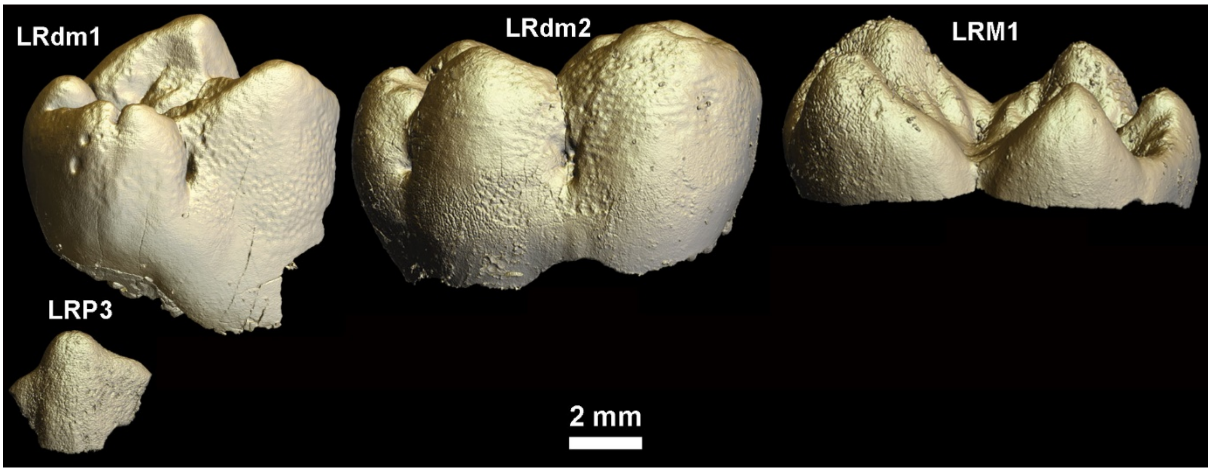

Fig. 23 – 3D models of the enamel caps of the ThI-GH-10978 teeth.

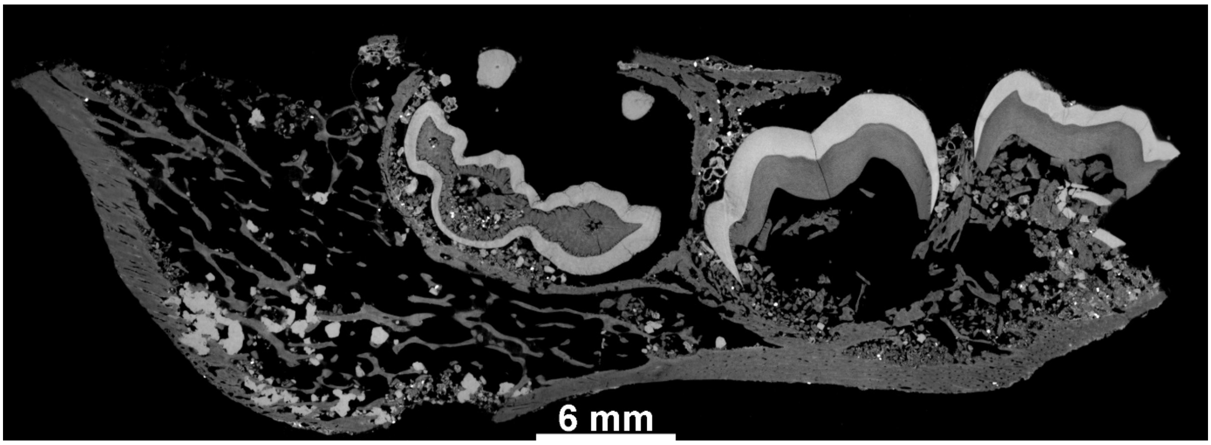

Fig. 24 – 2D section through the ThI-GH-10978 mandibular corpus showing the extensive taphonomic damages affecting the bone and teeth (from left to right: LRM1, LRdm2 and LRdm1).

Although the ThI-GH-10978 dentition is highly fragmentary, an attempt was made to compare its stage of development with recent *H. sapiens* standards and Neanderthal juveniles. A further challenge is that there are very few infants found in the fossil hominin record. The specimens discussed here for comparison are often at a different developmental stage and much younger geologically, yet this enables to highlight the mosaic pattern of development of the dentition.

Based on the atlas built by AlQahtani et al.[90] for characterizing recent *H. sapiens* dental development, and on the stage of calcification of the permanent and deciduous teeth of ThI-GH-10978, the closest recent *H. sapiens* equivalent would be at ~1 to 2.5 years at most. Using histological tooth sections, Mahoney[91] calculated the average crown formation time of modern human second deciduous molars to be 1.29 years. In a sample of modern-day Southern African lower molars, Reid and Dean[92] estimated that the mandibular M1 reaches half crown formation around 1.4 to 1.7 years of age. Overall, in modern human standards, one can estimate that ThI-GH-10978 was ~1.5 years at death.

Several juvenile early hominins show a stage of dental development close to or similar to that of ThI-GH-10978, some of which have a published histological age at death. The stage of development of the dm2 and the M1 crowns are of special interest.

The crown of Dederiyeh 1's maxillary M1 is half formed, which is quite similar to ThI-GH-10978. An histological section of Dederiyeh 1's UM1 yielded an age at death ranging from 1.4 to 1.6 years[93]. Dederiyeh 2 is slightly more advanced in dental development than Dederiyeh 1: three quarters of its M1 crown is formed, half of the roots of the mandibular dm2 is formed, and the lower dm1 has reached root formed at three quarters to root completion. Based on a comparison with modern standards its age at death estimated to be less than 2 years[94].

Similarly, Pech de L'Azé 1 is more advanced than both ThI-GH-10978 and the Dederiyeh infants, with the roots of the deciduous molars being fully formed and the crown of the M1 completed at three quarters, leading to an estimated age at death slightly less than 2 to 3 years[95].

The Neanderthal infant Kebara 1 was estimated to have died at ~1 year[96]. Although the occlusal views of the deciduous teeth and of the permanent tooth germs provided in this paper are dark, it seems that Kebara 1's dm1 had some roots formed, and the dm2 could be considered close to crown ¾ to complete, while the M1 crown may have been close to half formation. ThI-GH-10978 is at a similar or slightly more advanced stage of dental development.

The dm1 of ThI-GH-10978 seems more advanced than that of Amud 7 (see section in Fig. 11 in [97]), which was estimated to have died at ~10 months based on modern human standards[98,99].

Although they do not preserve any deciduous molars, Spy IV (and especially Spy 194a) and Palomas 49 could have been developmentally close to ThI-GH-10978, with a respective age at death estimated at 1.5 years[100] and 1.5 - 2.5 years[101]. Based on the morphology of the alveolar sockets of their deciduous molars and on modern human standards, the Spy IV's dm1 roots length was greater than crown height although they were not yet fully formed, and the dm2 started root formation, which remained less than crown height. In Spy IV and Palomas 49, the mental foramen is placed right below the dm1 (Crevecoeur et al., 2010; Walker et al., 2009). ThI-GH-10978's mental foramen must have been placed under or anteriorly to the dm1, yet taphonomic damages of the mandibular corpus preclude any reliable observation.

Conclusions

Although age death could not be accurately estimated based on histological data, one may speculate that ThI-GH-10978 was ~1.5 years of age, based on the comparison of its calcification stages with present-day humans and other fossil hominins.

Supplementary Note 8 – Dental Morphology

Shara E. Bailey

Abbreviations: EDJ – enamel dentine junction; OES – outer enamel surface; MMR – mesial marginal ridge; DMR – distal marginal ridge; AF – anterior fovea; MTC – middle trigonid crest; MeTC – mesial trigonid crest; DTC – distal trigonid crest; IP – interproximal (wear) facet.

THI-GH10717 Mandible

The lower right canine (RC,) is severely worn (category 6) with secondary dentine exposure. The distal half is substantially more worn than the mesial half resulting in a strong wear gradient. In between there is a marked wear step in labial view. The IP facets have been affected by the severe occlusal wear. There is a medium-sized chip missing from the distal-occlusal edge. The severe wear has obliterated all occlusal morphology at the OES. There is a vertical furrow on the distal aspect of the labial surface. At the EDJ remnants of MMR and DMR are preserved, suggesting some expression of shoveling (> grade 1) was originally present. The labial vertical furrow observed on the OES is not present at the EDJ, suggesting it could be an enamel defect, rather than an anatomical feature.

The lower left third premolar (LP_3) crown is severely worn (category 5/6), with little more than the enamel rim preserved. The wear has obliterated the occlusal crown morphology at the OES. However, the preserved enamel suggests a strong transverse crest joined the protoconid and metaconid. There is a faint vertical mesial furrow and ridge on the buccal aspect. In occlusal view, the asymmetrical crown is wider distally than mesially. The wide distal IP facet has been partially removed by occlusal wear. The smaller mesial IP facet has also been partially removed by wear. An occlusal chip obscures on the superior edge of the mesial IP facet. There is additional occlusal chipping observed on the distolingual edge. The EDJ provides only slightly more information. The vertical furrow and ridge on the mesiobuccal aspect of the crown is also observed at the EDJ. In addition, there is a triangle-shaped occlusal fovea that continues onto the lingual surface via a mesiolingual groove. A weak mesial accessory ridge can be observed running from the protoconid cusp tip to the MMR. The root is complete. There is a mesial groove running the length of the root, which forms a shallow v-shaped depression (Grade 1 Tomes' root[95]). The root ends in two apices. Internal imaging of the pulp chamber confirms a root bifurcation about two-thirds the distance from the cervix.

The lower right third premolar (RP_3) is worn similarly to its antimere (category 5/6) preserving primarily an enamel ring. The smaller mesial facet is circular, while the much larger distal IP facet is rectangular. As with its antimere most of the crown morphology on the OES has been obliterated by wear. However, the mesiolingual groove is preserved, and is more strongly expressed than it is on the left. There is also a shallow distolingual groove present. The two grooves border the metaconid. At the EDJ the mesial and distal lingual grooves are more visible. There is also a low mesiolingual vertical ridge/furrow. The single root has two distinct apices. On the mesial aspect there is a deep, v-shaped groove separating the roots. The groove extends slightly more than one-third the total root length (Grade 3 Tomes' root[95]). On the distal aspect the roots are joined by a dentine sheath. Imaging of the pulp chamber shows a trifurcation of the root: there are two main mesial and distal branches and the distal branch splits about one-third the distance from the apex.

The lower left fourth premolar (LP_4) is markedly worn (category 5). Only a portion of the area around the occlusal basin and the enamel ring are preserved. The mesial and distal IP facets are wide. Two large pieces of the crown have been broken from the buccal aspect and distobuccal corner. The crown shape is a symmetrical squared oval. The EDJ does not provide additional morphological information. The single root is robust. On the lingual aspect the root bifurcates into separate apices. There are wide and shallow mesial and distal grooves and a shorter, deeper distobuccal groove. Imaging of the pulp

chamber confirms two primary branches, mesial and distal, with the distal branch bifurcating about one-quarter the distance from the apex.

The lower right fourth premolar (RP_4) has a badly damaged crown, but the root is intact. Only the buccal half of the crown is preserved and only a partial enamel ring can be observed. No morphological information can be observed from either the OES or EDJ. The single root is robust and internal imaging shows a single large pulp chamber.

The lower left first molar (LM_1) is severely worn (category 6) with only the enamel rim preserved. This wear has obliterated any occlusal morphology. There is also a chip missing from the mesiobuccal corner. The mesial and distal IP facets are large, and approximately half the mesial IP facet has been removed by occlusal wear. What remains of the mesial IP facet preserves sub-vertical grooves, the etiology of which has been debated[103-105]. The occlusal wear pattern is atypical. Under normal occlusion, mandibular crowns are more heavily worn buccally than lingually. In this molar (and its antimere) the distolingual quarter substantially more worn than the rest of the crown. Moreover, the mesiolingual quarter of the crown is unusually high compared to the remaining crown. On the buccal aspect no enamel extension is observed. The mesial and distal roots each have two radicals and two separate apices. The radicals are separated by shallow, wide grooves. Internal imaging shows that the pulp chamber of mesial root bifurcates one-third the distance from the cervix. The distal branch of the pulp chamber bifurcates approximately one-third the distance from the apex.

The lower right fist molar (RM_1) is severely worn with exposed secondary dentine (category 6). It retains only an enamel rim. The large and wide IP facets on the mesial and distal aspects have been eroded and/or chipped. The depth of the mesial IP facet has made the mesial aspect of the crown concave. The tooth exhibits the same unusual wear pattern as its antimere. The distolingual quarter is considerably more worn than the buccal aspect and the mesiolingual quarter of the crown is unusually high. Damage to the crown has removed most of the lingual portion, part of the distobuccal portion and much of the mesial portion of the enamel rim. The occlusal surface also exhibits chipping. On the buccal aspect a deep intercuspal groove is preserved and no enamel extension is present. Each of the two roots (mesial and distal) has two radicals separated by a shallow, wide groove. Each root ends with two distinct apices. Internal imaging of the pulp chamber shows branching of the mesial portion about one-third the distance from the cervix, whereas the distal root is primarily a wide, single branch, which bifurcates about one-third the distance from the apex.

The lower left second molar (LM_2) is heavily worn, with a large dentin facet connecting the lingual cusps that continues onto the distal surface and effects the hypoconulid (category 5). Like the first molars, the wear pattern is atypical, being stronger lingual than buccal and more distal than mesial. There is a large dentine patch on the protoconid and a smaller dentine patch on the hypoconid. Both mesial and distal IP facets are large and wide and contain multiple sub-vertical grooves. These grooves are deepest on the distal facet. The extensive wear has obliterated the occlusal morphology at the OES. On the buccal aspect no enamel extension is observed. At the EDJ the essential crests of the protoconid, metaconid and hypoconid are well developed and those of the protoconid and metaconid join to form a continuous MTC that dips at the sagittal sulcus (grade 2). The MeTC and DTC are absent. The MMR is high and there is an elevation between the protoconid and metaconid corresponding to a mesial accessory tubercle. The pattern of the preserved fissures and crown shape suggest that the hypoconulid was large. There are two roots preserved. The mesial root has two distinct radicals that end in separate apices. The radicals are separated by shallow mesial and distal grooves. The lingual root has a single radical and single root apex, although a mesial groove is also present. Internal imaging of the pulp chamber shows branching of the mesial root near the cervix, while the distal root maintains a single, wide pulp chamber.

The lower right second molar (RM_2) is heavily worn. All cusps are worn and there is a large, continuous, and U-shaped dentin facet joining them (category 5). Unlike its antimere, the wear pattern is typical (more worn buccally than lingually). The protoconid is the most heavily worn cusp and only hints of the original fissure pattern are preserved at the OES. Both mesial and distal IP facets are large

and wide. The mesial IP facet contains sub-vertical grooves. No enamel extension is observed. At the EDJ the essential crests of the protoconid and metaconid are well developed but a continuous MTC is absent (grade 1). The MeTC and DTC are also absent. The MMR is high. The pattern of the preserved fissures and crown shape suggest that the hypoconulid was large. The tooth possesses two roots. The mesial root comprises two distinct radicals with separate apices. The radicals are separated by shallow mesial and distal grooves. The lingual root has a single radical and single root apex, although a mesial groove is also present.

The lower left third molar (LM_3) is considerably less worn than the other molars. Although all cusps are flattened, there is only a single small dentine patch on the protoconid cusp tip (category 2). Consistent with the wear pattern observed in the more mesial molars the crown exhibits unusual distolingual wear relative to the other cusps. There is a wide mesial IP facet that contains sub-vertical grooves. The crown is a distally-tapered oval. The distal cusps are relatively small compared to the mesial cusps. No trigonid crests are preserved at the OES and cusps are arranged in a Y-fissure pattern. The OES is too worn to accurately assess cusp number, although multiple cusps are suggested by the preserved occlusal fissures. At the EDJ we observe a moderately-sized hypoconulid (grade 4) and a similarly-sized cusp 6 (grade 3). The cusp 7 is expressed on the distal shoulder of the metaconid (grade d). On the mesiobuccal aspect of the protoconid there is a vertical ridge bordered mesially and distally by shallow grooves. The ridge merges inferiorly with a horizontal shelf on which there is a weak dentine elevation. This morphology may be part of the protostylid complex but its expression is not captured by[102]. The essential ridges of the protoconid and metaconid are well developed but they do not form a continuous MTC (grade 1). The metaconid ridge is straight but exhibits a midline constriction, or weak deflecting wrinkle (grade 1). No enamel extension is observed. The tooth possesses well-separated mesial and distal roots. The mesial root has two radicals separated by shallow grooves. The distal root has a single radical. Internal imaging of the pulp cavity shows bifurcation in the mesial branch near the cervix and a single distal branch.

The lower right third molar (RM_3) is slightly more worn than its antimere. The dentine patch on the protoconid is moderately sized and there is an additional small dentine patch on the hypoconid (category 3). There is a wide mesial IP facet that contains multiple sub-vertical grooves. Consistent with the wear pattern observed on its antimere, the crown exhibits unusual distolingual wear relative to the other cusps. Also like its antimere, the ovoid crown tapers distally. Although much of the occlusal morphology has been removed by wear, the trigonid portion of the sagittal sulcus and a small portion of the transverse fissure are visible. The shape of the crown suggests that the hypoconulid, cusp 6 and possibly cusp 7 were present. At the EDJ the protoconid and metaconid present well-developed essential ridges but continuous trigonid crests are absent. The deflecting wrinkle observed on the left antimere is absent on the right. There are dentine horns corresponding to a moderate hypoconulid (grade 3) and equally-sized cusp 6 (grade 3). In between there is a small accessory cuspule with a weak, but independent, dentine horn. The cusp 7 is expressed as a dentine horn-like feature is not closely associated with the metaconid (grade d). There is a weak shelf on the buccal aspect at the EDJ that extends from the MMR and wraps around the protoconid. It traverses horizontally before fading distal to the buccal groove. This shelf makes the mesiobuccal portion of the crown project disproportionately and, like its antimere, is likely related to the protostylid complex. The tooth possesses well-separated mesial and distal roots. The mesial root has two radicals separated by a shallow and wide mesial groove and a deeper distal groove. The distal root has a single radical. Internal imaging of the pulp cavity confirms two branches to the mesial root and a single branch in the distal root.

ThI-GH-1 Hemimandible

The lower right fourth premolar (RP_4) crown is square in occlusal profile having been flattened mesially and distally by IP wear. The protoconid is worn flat with a large dentine facet and the

metaconid has been worn to a similar height but possesses a smaller facet (category 4). The thick DMR has also been flattened by wear, but no dentine is exposed. The OES preserves a deep transverse fissure and a deep, pit-like AF. On the buccal aspect there are faint mesial and distal vertical furrows. The large and wide mesial and distal IP facets. The EDJ provides additional morphological information. The essential ridge of the metaconid is bifurcated. The mesial branch forms a continuous crest with the that of the trifurcated protoconid essential ridge. This ridge, together with the high and sharp MMR, circumscribe a large AF. Short mesial and distal accessory ridges emanate from the buccal margin. The high, sharp DMR is delineated by a deep mesial groove. The DMR extends onto the lingual surface. There is a triangular-shaped fovea on the distobuccal aspect of the crown corresponding to the vertical ridge on the enamel surface. On the mesiobuccal aspect there is a vertical ridge/furrow. The single root is deeply invaginated on the lingual aspect, bifurcating about half the distance from the cervix, while a dentine sheath connects the roots on the distal aspect (grade 3 Tomes' root). Imaging of the pulp chamber shows a bifurcation where the root splits with mesial and distal branches.

The lower right first molar (RM_1) crown is quite worn with a large dentine patch that connects the protoconid, hypoconid, hypoconulid and part of the entoconid and a smaller dentine patch on the metaconid (category 5). There are wide mesial and distal IP facets lacking sub-vertical grooves. The worn OES preserves deep buccal and lingual grooves separating the protoconid/hypoconid and metaconid/entoconid. At the EDJ the metaconid with the protoconid mesial accessory ridge form a continuous MeTC. There is a distal accessory ridge on the metaconid and no indication of a deflecting wrinkle. Middle and DTCs are absent. The mesial and distal roots are well separated. Each has two distinct radicals with separate apices. Internal imaging of the pulp shows a bifurcation of the mesial branch just below the cervix. The distal branch bifurcates about one-third the distance from the cervix and the lingual branch bifurcates one-third the distance from the first bifurcation.

The lower right second molar (RM_2) is moderately worn (category 4), with medium-sized dentine patches on the protoconid, hypoconid and entoconid and smaller dentine patches on the metaconid, hypoconulid and cusp 7. The mesial and distal IP facets are wide. The mesial IP facet possesses sub-vertical grooves. There are some small chips removed from on the occlusal border of both the mesial and distal IP facets. Most of the crown morphology has been obliterated by wear. However, some of the occlusal fissures are preserved suggesting a + or X fissure pattern and a moderate-sized AF (grade 3), which is bordered distally by mesial accessory ridges of the metaconid and protoconid. The buccal grooves between the protoconid/hypoconid and between the hypoconid/hypoconoulid are deep. There is no enamel extension present. The EDJ provides additional morphological information. At the EDJ the MMR is high and thin. A discontinuous MeTC and a continuous MTC (grade 3) are present. There are also distal accessory ridges on the protoconid and metaconid that meet at the sagittal sulcus but do not form a DTC. There are additional accessory ridges emanating from all major cusps. The deflecting wrinkle is absent. What appears to be a worn cusp 7 on the OES was more likely an accentuated distal accessory ridge on the metaconid. It is indicated only by a slight elevation on the occlusal rim, and there is no lingual grove associated with a 'true' cusp 7. There are weak vertical furrows on the mesial aspect of the protoconid, mesial and distal to the buccal groove and also associated with the hypoconulid. The vertical furrow on the hypoconulid is associated with an oblique shelf-like ridge, which is particularly noticeable in distal view. These buccal features may be related to the protostylid complex but are not accounted for by Turner et al.[102]. The mesial and distal roots run parallel to each other. The mesial root has two radicals separated by a groove and there are two apices. The distal root also has two radicals but they are only divided on the mesial surface of the root. Imaging of the pulp shows that the bifurcation of the mesial pulp occurs near the cervix, whereas the bifurcation of the distal branch occurs approximately half the length of the root.

The lower right third molar (RM_3) is lightly worn with a single, small dentine facet on the protoconid (category 3). There is a large mesial IP facet, which is offset lingually such that most of the wear affects the protoconid. There is some minor chipping on the buccal surface of the entoconid and the distal surface of the cusp 6. The six cusps are configured in a Y-pattern. There is a moderate-sized AF (grade

3) bordered distally by essential ridges of the protoconid and metaconid. The sagittal sulcus is continuous and divides these ridges at the OES. There appears to be a shelf-like protostylid that extends the protoconid and hypoconid. There is a shallow buccal pit just below the shelf. At the EDJ the MMR is well defined and it forms the mesial border of the wide AF. The essential ridges of the protoconid and metaconid are well developed but do not form a continuous MTC (grade 1). The protoconid has a distal accessory ridge. The deflecting wrinkle is absent. The essential ridges of the distal cusps are slightly less well-developed than the mesial cusps but they are clearly present. The entoconid also has a distal accessory ridge. There is a low accessory dentine horn between the hypoconulid and cusp 6 along the DMR. At the EDJ it appears that the protostylid shelf is continuous with the MMR. The mesial and distal roots run parallel to each other. The mesial root is wider than the distal root and comprises two radicals separated by a wide developmental groove mesially and a deeper groove distally. The radicals end in separate root apices. The distal root has a developmental groove on its mesial surface but not on its distal surface. Internal imaging of the pulp shows a complex pattern with the mesial branch bifurcated but then rejoining briefly and splitting again about one-third the distance to the apex. The distal branch starts rather narrow and widens towards the apex, but it does not bifurcate.

ThI-GH-10978

The right deciduous lower first molar (RdM_1) crown is unworn. The metaconid and protoconid are well developed and high. The hypoconid possesses two cusp apices which appear be the result of a split dentine horn rather than two distinct cusps (see EDJ description below). The entoconid is smaller and somewhat lower than the hypoconid. There is a deep buccal groove mesial to the hypoconid, the groove deflects distally, spills onto the occlusal rim and is associated with a v-shaped fovea. The MMR is thick. It has a short and deep mesiolingual groove separating it from the metaconid. There is a weak groove separating the MMR from the protoconid. At the EDJ there is a weak elevation on the distal aspect of the protoconid. Examination of the distobuccal region shows that rather than there being a true hypoconulid, the hypoconid dentine horn is split into two. The buccal aspect shows a large, deep v-shaped fovea between the mesial and distal buccal cusps. There is also a vertical ridge associated with the essential lobe of the hypoconid. It is sharp and restricted to the middle part of the cusp. Additional faint ridges are present distally at the EDJ. The MMR is high and sharp and the AF is deep and continuous with the mesial lingual groove. The essential crests of the mesial cusps are joined by a sharp and low trigonid crest. The cusp apices are sharp and high.
The lower right deciduous second molar (RdM_2) is unworn.The five main cusps form a Y fissure pattern. They are separated by deep grooves. The buccal grooves separating protoconid/hypoconid and hypoconid/hypoconulid each terminate in a deep pit at about half the crown height. The deeper lingual groove extends three-quarters the height of the crown. There is a large metaconulid-type cusp 7 (grade c). The MMR is thick. The protoconid and metaconid possess mesial accessory ridges that are separated by the sagittal sulcus. Their essential ridges meet to form a low MTC (grade 2). The essential crests of all five main cusps are well developed. The EDJ shows a complex occlusal surface. In addition to well-developed essential ridges, they possess weak accessory ridges. A low but sharp MTC joins the protoconid and metaconid. A mesial accessory ridge of the protoconid joins a short crest emanating from the MMR (similar, but not identical to Type 2 of de Pinillos et al 2014). Another short crest emanates from the marginal ridge of the metaconid. No DTC or deflecting wrinkle is present. The deep pits associated with the buccal grooves are associated with wide foveae at the EDJ. The mesial buccal groove is also associated with a weak elevation bordered mesially and distally by vertical furrows. There is a vertical associated with the mesial aspect of the cusp 7.
The lower right permanent first molar (RM_1) is represented by an incompletely mineralized tooth cap; slightly less than half the crown is formed. There are five main cusps arranged in a Y pattern. Cusp 6 and 7 are absent. All five cusps have well developed essential ridges. The MMR is thick and protrudes mesially. It has weak fissures suggestive of accessory cusps. The essential ridges of the entoconid and

hypoconulid form the mesial border of a weak distal fovea. Similar to the dM_2 the main cusps are separated by deep buccal and lingual grooves. At the EDJ the essential ridges of the protoconid and metaconid do not form a continuous MTC (grade 1) and the deflecting wrinkle is absent. There are two small dentine elevations and three weak ridges emanating from the MMR. There is a vertical elevation and associated furrow on the mesiobuccal aspect of the protoconid and there is an oblique ridge emanating from the distal aspect hypoconulid cusp tip and coursing towards the hypoconid.

ThI-GH Isolated teeth

ThI-GH-SA26-88 is an upper left central incisor (LI^1). It is moderately worn with about one-quarter of the crown height removed (category 3). The wear is obliquely oriented such that the lingual aspect is considerably more worn than the labial aspect. The lingually oriented wear is unusual (in Neanderthals, for example, incisors get labially beveled with increased wear) and could reflect malocclusion. There is a moderately sized mesial IP facet and a smaller, rounder distal IP facet. Chipping can be observed along the labial incisal edge. The mid-crown is moderately convex (grade 3), although the incisal edge is straighter. We infer that the crown was not strongly shovel shaped (< grade 3), as there is no indication of marginal ridges on the preserved lower half of the crown and the lingual fossa is shallow. There is, however, a weak vertical elevation offset distally from the midline of the tooth. There is no lingual tubercle. The root and crown are moderately convex along the length in sagittal view. The tooth possesses two damaged roots, which may have been joined by a dentine sheath distally. Internal imaging shows that each root has a single pulp chamber.

ThI-GH-SA26-90 is an upper right third premolar (RP^3). It is quite worn (category 5). The cusps are flat and possess large dentine facets, which have removed most of the occlusal morphology. There are large mesial and distal IP facets that are obliquely oriented. Although worn, the crown preserves deep anterior and posterior grooves delineating the MMR and DMR. There is a vertical furrow on the mesiobuccal aspect of the crown, which defines the essential lobe. At the EDJ, a continuous transverse crest joins the protocone and paracone. The essential ridges of the paracone appears to be trifurcated. No accessory marginal cusps are noted. The vertical furrow on the mesiobuccal aspect is expressed as a ridge at the EDJ. The tooth possesses two roots, which begin to split one-third (mesial) to one-half (distal) the distance from the cervix. The developmental groove separating them is deeper and wider mesially than it is distally.

ThI-GH-OA23-24 is an upper left fourth premolar (LP^4). It is moderately worn: the cusp tips are removed and the crown exhibits two small dentine facets (category 3). There are large mesial and distal IP facets present. The distal facet exhibits multiple sub-vertical grooves. The OES preserves a thick DMR and thinner, but still well-defined, MMR. Both are delineated by deep transverse grooves, which run perpendicular to the sagittal sulcus. The essential ridge of the paracone appears to be trifurcated and a sagittal sulcus is continuous at the OES. There is a vertical furrow/elevation on the mesiobuccal aspect of the paracone. At the EDJ the essential lobe of the paracone has one strong and three weak ridges running from the cusp tip towards the sagittal sulcus. The most prominent ridge is sharp. It joins a similar ridge emanating from the protocone and together these form a continuous transverse crest. Short mesial and distal accessory ridges (MxPAR grade 2) are visible on the paracone and are associated with weak elevations on the occlusal rim. The protocone also has accessory ridges emanating from the cusp tip. No accessory marginal ridge cusps are noted. The vertical furrow/elevation on the mesiobuccal aspect takes the form of a furrow/sharp ridge at the EDJ. The tooth has a single root with two radicals separated by shallow grooves and two distinct apices. Internal imaging shows a bifurcation of the pulp chamber approximately half the distance from the cervix.

ThI-GH-PA24-107 is an upper left third premolar crown (LP^3). It is quite worn with a moderately-sized dentine facet on the paracone and a larger facet on the protocone (category 4). Both facets exhibit basin-like wear. There are large mesial and distal IP facets. There is a small chip above the distal IP facet. Deep fissures border the MMR and DMR. On the buccal aspect there are mesial and distal vertical furrows associated with weak vertical ridges. At the EDJ there is a moderate elevation of the

buccal essential lobe, associated with a mesial vertical furrow. The MMR and DMR are high, sharp and delineated by deep furrows. The essential ridge of the paracone is bifurcated, the mesial arm of which joins the protocone essential ridges to form a low, sharp continuous transverse crest that crosses the sagittal sulcus. The tooth most likely had two roots but the lingual one has been broken post-mortem. It is not possible to say to what degree the roots were joined (or not) by a dentine sheath.

Supplementary Note 9 – ThI-GH-10978 Crown outline morphometry

Stefano Benazzi and Rita Sorrentino

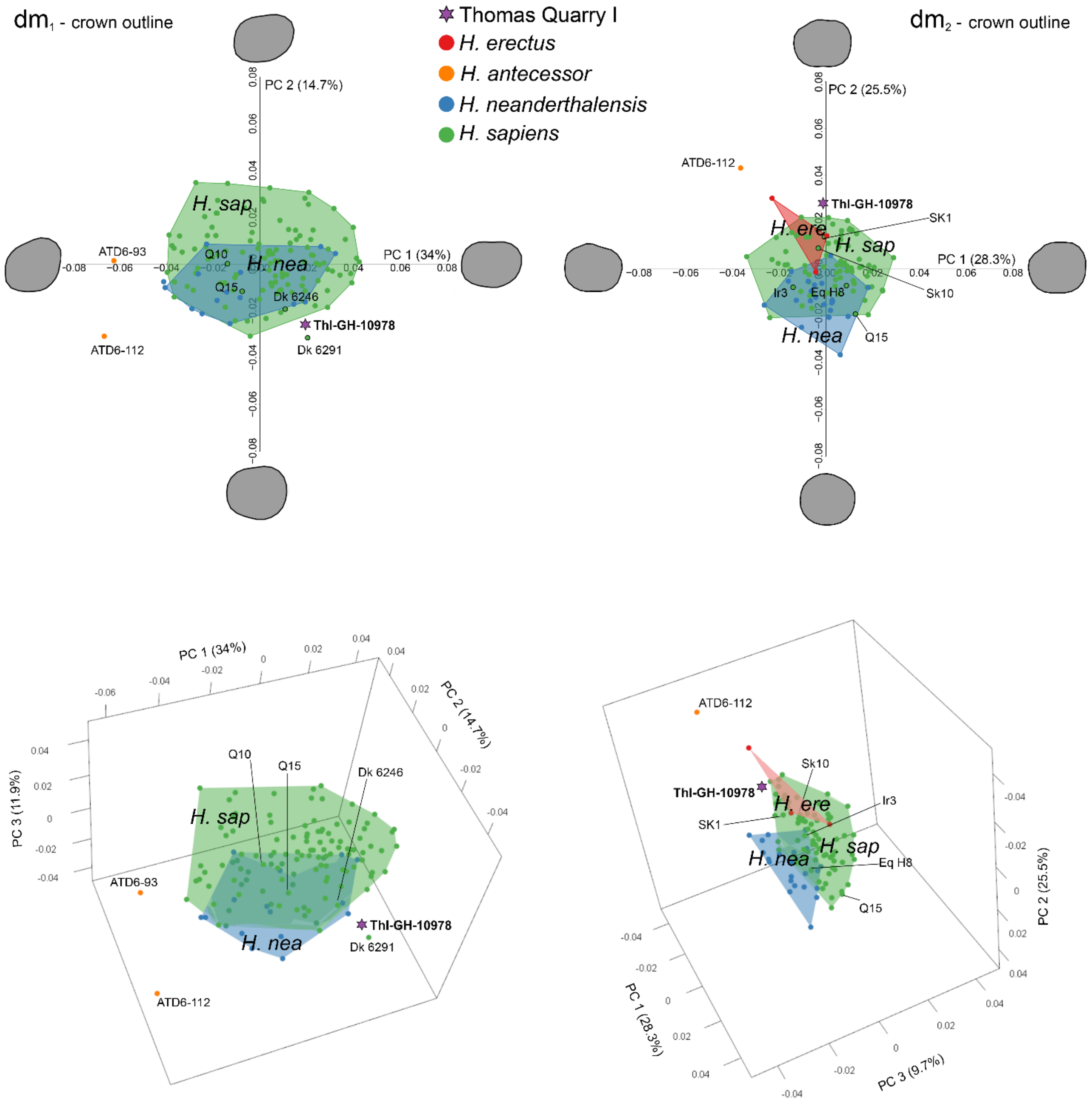

Figure 25. 2D and 3D shape–space PCA plots of left dm_1 (right) and dm_2 (left) crown outlines. The shape-space is computed on *H. sapiens* and Neanderthal specimens, whereas others are projected including the ThI-GH specimen. Extreme shape changes along the PC axes are represented for PC1 and PC2. Specimens are listed in Supplementary Table 7

dm1

The first three PCs account for 60.6% of the total variance (PC1=34 %, PC2=14.7%, and PC3=11.9%; Fig. 24). A Permutation test, computed on the first 3 PCs, reveals significant differences between Neanderthals and the RHS samples (p<0.01; Table S9), although the two groups overlap in the shape space PCA plot (Fig. S24). The ThI-GH-10978 plots outside the *H. sapiens* and Neanderthal variability

along the negative PC2 axis, quite close to an early *H. sapiens* specimen (Die Kelders 6291) and at the opposite side of ATD6-93 and ATD6-112. The ThI-GH-10978 dm_1 reflects a more rectangular shape (positive PC 1) and relatively large mesial cusps (negative PC2), resembling early *H. sapiens* specimens. However, it is worth noting that these characteristics are equally seen in both recent *H. sapiens* and Neanderthals. ATD6-93 and ATD6-112 are characterized by a more trapezoidal shape with a mesiobuccal projection related to the mesiobuccal cusp. The QDA performed on 5 PCs (accounting for 79.7% of the variance) distinguishes Neanderthals and *H. sapiens* (early and recent combined) with 72.3% accuracy (Table S10). ThI-GH-10978 is classified as *H. sapiens* with a posterior probability of 100%.

	Early *H. sapiens* (n=4)	Neanderthal
Neanderthal (*n* = 15)	1.000	
Recent and Upper Paleolithic *H. sapiens* (*n* = 112)	0.815	**0.004**

Table 9. Permutation tests (Bonferroni corrected p-value) of significant differences in crown shape of the dm_1 among Neanderthals, early and recent *H. sapiens*.

	H. neanderthalensis	*H. sapiens*	% correct
H. neanderthalensis (*n* = 15)	11	4	73.3
H. sapiens (*n* =116)	32	83	72.2

Table 10. Results of quadratic discriminant functions assignments (early and recent *H. sapiens* combined) based on the crown shape of the dm_1 by using 5 PCs (79.7%).

dm_2

The first three PCs account for 63.5% of the total variance (PC1=28.3 %, PC2=25.5%, and PC3=9.7%; Fig.1). Permutation test, computed on the first 3 PCs, reveals significant differences between Neanderthals and the RHS samples ($p<0.01$; Table S11). ThI-GH-10978 plots outside, but close to, the *H. sapiens* and *H. erectus* ranges of variation. It is positioned along the positive scores of PC2, which accounts for a rectangular shape, a reduction of the buccodistal crown outline shape and a straight lingual outline shape, as seen in *H. sapiens* (both early and recent). These features are also common in *H. erectus*, but not in Neanderthals, who are instead characterized by a more oval shape, buccodistal expansion and convex lingual outline shape. Despite sharing a straight lingual outline, the shape of ThI-GH-10978 dm2 differs from that of ATD6-112, as the latter still exhibits an enlarged buccodistal outline, albeit less pronounced than in Neanderthals. The QDA performed on 6 PCs (accounting for 82.6% of the variance) distinguishes between *H. neanderthalensis* and *H. sapiens* (both early and recent combined) with 93.1% accuracy (Table S12). ThI-GH-10978 is classified as *H. sapiens* with a posterior probability of 99.9%.

	Early *H. sapiens* (n=5)	Neanderthal
Neanderthal (*n* = 23)	1.000	
Recent and Upper Paleolithic *H. sapiens* (*n* = 73)	0.968	**0.000**

Table 11. Permutation tests (Bonferroni corrected p-value) of significant differences in crown shape of the dm_2 among Neanderthals, early and recent *H. sapiens*.

	H. neanderthalensis	*H. sapiens*	% correct
H. neanderthalensis (*n* = 23)	20	3	86.9
H. sapiens (*n* = 78)	4	74	94.9

Table 12 Results of quadratic discriminant functions assignments (early and recent *H. sapiens* combined) based on the crown shape of the dm_2 by using 6 PCs (82.6%).

Supplementary Note 10 - Anterior dentition morphometrics

Adeline Le Cabec

ThI-GH has yielded few permanent anterior teeth, among which an isolated tooth and teeth *in-situ* in a mandible. The anterior tooth root morphology has proven quite discriminant between Neanderthals and *H. sapiens*[106] and could therefore contribute to better describe the taxonomical status of these teeth.

The isolated tooth, ThI-GH-SA26-88, is well-preserved and was published by[16] as a maxillary central incisor. However, its anatomical attribution remains uncertain (see discussion hereafter). In the mandible ThI-GH-10717, the right permanent canine is well-preserved *in-situ*. In contrast, the left canine and incisors, and the right central incisor are heavily damaged: their crown and the cervical portion of their root are broken away and were not recovered. The right lateral incisor is slightly better preserved, the enamel and cervical line being present on the distal aspect of the tooth and on half of the labial and lingual aspects of the crown.

From the micro-CT scans, the tooth tissues of these teeth were segmented (i.e., enamel, dentine, pulp cavity) and several variables were measured after the protocol described in[106]. Comparative data (Supplementary Table S 7) involve Neanderthals, early *H. sapiens* (EHS), upper paleolithic and Epipaleolithic humans (UPEPIH), and recent *H. sapiens* (RHS), all from[106]. Three teeth from Tighennif (Algeria; ~ 1 Ma, see [107] for a study of teeth from Tighennif) were segmented and measured as well. These unpublished results are reported in **Tables 13-14**. The Tighennif and ThI-GH teeth were compared to the other samples using adjusted z-scores (later abbreviated as “Azs”; See[108,109] for details on the statistics). An Azs close to 0 indicates that the specimen of interest is very close to the mean of the comparative sample. The specimen falls within the variation of the comparative sample when $-1 \leq Azs \leq 1$. When $Azs > 1$ or $Azs > -1$, the specimen falls outside of the Student test SD variation of the comparative sample.

ThI-GH-SA26-88

The root of the maxillary incisor ThI-GH-SA26-88 is more robust than that of recent *H. sapiens* and fall closer to the Neanderthals and EHS variations (**Table 15**). Its labio-lingual crown diameter is relatively small, which would tend to classify ThI-GH-SA26-88 as a lateral incisor (**Figs. 26-27**). To note that the bucco-lingual and mesio-distal crown diameters seem to have been swapped in Raynal et al. [16]. For both root length and labio-lingual crown diameter, ThI-GH-SA26-88 is much smaller than the KNM-WT 1500A (*H. erectus*) incisor. When compared to root lengths collected from the literature (**Fig. 28**), the root length of ThI-GH-SA26-88 falls in the lower end of the Sima de Los Huesos variation, and is lesser than that of Dmanisi.

ThI-GH-10717

Since the crown of the lateral incisor of ThI-GH-10717 is heavily damaged, only some measurements were taken, and when they may be affected by the missing enamel and dentine, they are indicated in parentheses in **Table 13**. In spite of this, the values of this LRI2 are overall relatively close to ThI-GH-SA26-88, or even greater (root surface and volume). In spite of the damaged crown, ThI-GH-10717 LRI2 labio-lingual crown diameter is clearly small and falls within the recent *H. sapiens* variation. The mandibular incisors Tighennif-1954-7-04 and Tighennif-1954-7-08 have gracile roots and small crowns (which is quite similar to the ThI-GH-10717 LRI2), within the range of variation of recent *H. sapiens* (**Table 13**, **Fig. 29**). Root length (which is reliably measured) of the ThI-GH-10717 LRI2 falls in the area of overlap between Neanderthals, early and recent *H. sapiens.* More specifically, it falls close to Mauer and Sangiran S7-50 (*H. erectus*), but is greater than Dar-es-Soltane II H4 and Temara (Grotte des Contrebandiers), and to a lesser extent to Tighennif 1954-7-08. The ThI-GH-10717 LRI2 root is slightly shorter than that of Irhoud 11. When compared to root length collected from the literature for other hominins (Fig. 31), the ThI-GH-10717

LRI2 root falls close to Dmanisi and Sima de Los Huesos.

The dimensions of the mandibular canine of ThI-GH-10717 fall in the middle of the *H. sapiens* range of variation and show a relatively short root for its crown size (**Fig. 30**). Its root is shorter than that of Temara, Dar-es-Soltane II H4, Irhoud 11, Mauer and to a greater extent, than that of Tighennif 1954-7-05. When compared to data from the literature (**Fig. 31**), the ThI-GH-10717 LRI2 root is much shorter than in ATD6-H1 (Gran Dolina), Sima de Los Huesos, and Dmanisi.

Conclusions

These results tend to show that ThI-GH-SA26-88 is more likely a maxillary lateral incisor. In terms of root shape, the ratio between the labial and the lingual portions of the total root surface area is overall greater than 1, attesting for a slight labial convexity, similarly to Neanderthals, and to a lesser extent to early *H. sapiens*, including Aterians. In addition, neither the Tighennif nor ThI-GH permanent anterior teeth show any marked lingual relief (shovel-shaped morphology for the incisors), as for the younger early *H. sapiens* Dar-El-Soltane II H4 and Temara (Grotte des Contrebandiers).

The crown and root morphometrics show the mosaic pattern between a robust morphology evocative of the retention of ancestral characters, and gracile features more similar to recent *H. sapiens*.

Specimen	Tooth type	Wear[a]	CrLL	RL	R(MD)	R(LL)	RSA
ThI-GH-SA26-88	ULI2	4	6.3	16.0	5.7	6.1	249.1
ThI-GH-10717	LRI2	0	-	16.7	-	(5.7)	(305.4)
Tighennif 1954-7-04†	LRI1	3	6.5	13.5	6.5	4.3	216.3
Tighennif 1954-7-08	LRI2	4	6.2	15.4	4.3	6.4	242.4

[a] wear scored after[110]; LI: mandibular incisor, UI: maxillary incisor. CrLL, R(MD), R(LL) in mm; RSA in mm^2; *: value that required estimation of the missing portion of a broken root following[106]. †: the distal end of the crown is broken. Values in parentheses for ThI-GH-10717 LRI2 are considered under-estimated. Measurements that were too affected by the tooth damage are not reported.

Specimen	Tooth type	RSA -Ling	RSA_Lab	RSA_Lab/ RSA_Ling	CA	RV	RPV	CrPV
ThI-GH-SA26-88	ULI2	105.0	144.5	1.4	28.7	307.6	16.7	4.2
ThI-GH-10717	LRI2	-	-	-	-	(313.3)	-	-
Tighennif 1954-7-04	LRI1	94.4	122.2	1.3	21.4	232.0	8.3	5.2
Tighennif 1954-7-08	LRI2	105.1	137.6	1.3	22.1	252.8	20.4	7.1

RSA_Ling, RSA_Lab, CA in mm^2; RV, RPV, CrPV in mm^3; [#]values affected by broken root by not corrected; *: value that required estimation of the missing portion of a broken root following[106]. Values in parentheses for ThI-GH-10717 LRI2 are considered under-estimated. Measurements that were too affected by the tooth damage are not reported.

Table 13. Crown and root metrics of the ThI-GH and Tighennif incisors.

Specimen	Tooth type	Wear[a]	CrLL	RL	R(MD)	R(LL)	RSA
ThI-GH-10717	LRC	5	8.0	15.0	5.1	7.8	429.4
Tighennif 1954-7-05	LLC	4	9.9	22.7*	7.1	9.9	432.7*

[a] wear scored after[110]; L: mandibular canine; UC: maxillary canine. CrLL, R(MD), R(LL) in mm; RSA in mm²; *: value that required estimation of the missing portion of a broken root following[106].

Specimen	Tooth type	RSA-Ling	RSA_Lab	RSA_Lab/RSA_Ling	CA	RV	RPV	CrPV
ThI-GH-10717	LRC	121.1	143.1	1.2	32.4	313.9	10.5	6.1
Tighennif 1954-7-05	LLC	167.1#	179.4#	1.1#	56.0	825.7*	33.7#	10.9

RSA_Ling, RSA_Lab, CA in mm²; RV, RPV, CrPV in mm³; #values affected by broken root by not corrected; *: value that required estimation of the missing portion of a broken root following[106].

Table 14. Crown and root metrics of the ThI-GH and Tighennif canines.

	UI1_Neand	UI2_Neand	UI1_EHS	UI2_EHS	UI1_UPEPIH	UI2_UPEPIH	UI1_RHS	UI2_RHS
CrLL	-1.7	-1.9	-0.5	-0.2	-0.8	0.4	-0.1	0.6
R(MD)	-0.6	-0.3	-0.3	0.1	-1.0	1.1	-0.1	1.2
R(LL)	-1.4	-1.5	-0.3	-0.4	-0.8	0.0	-0.3	0.3
RL	-0.2	-0.4	0.0	0.1	0.5	0.5	1.1	1.0
RSA	-0.5	-1.0	0.0	0.1	0.3	0.7	0.9	1.3
RSA_Ling	-0.5	-0.8	0.0	0.0	-0.2	0.3	0.0	0.8
RSA_Lab	-0.4	-0.9	0.0	0.2	0.8	1.1	1.6	1.4
CA	-0.8	-0.8	-0.2	0.0	-0.7	0.7	-0.2	1.0
RV	-0.6	-0.9	0.0	0.1	0.2	0.9	0.9	1.6
RPV	-0.5	-0.4	0.0	-0.1	0.7	1.1	0.7	0.9

Table 15. Assessment of the position of the ThI-GH-SA26-88 maxillary incisor in relation to the mean of the comparative samples (adjusted z-scores).

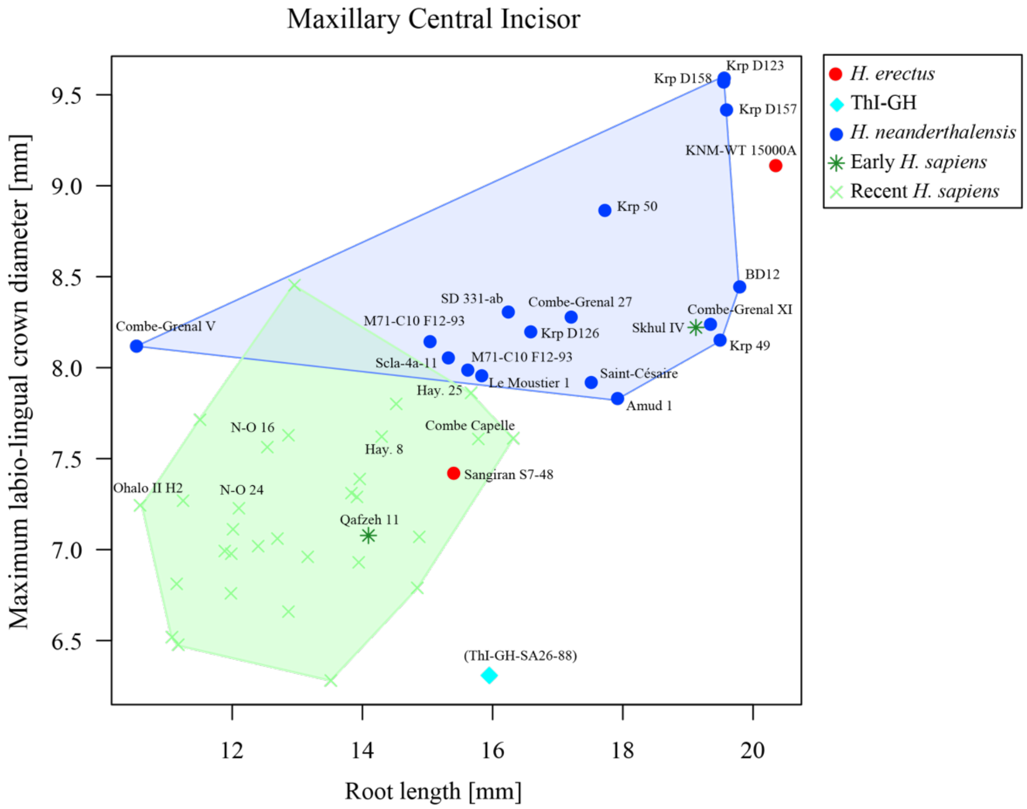

Figure 26. Bivariate plot of root length against labio-lingual crown diameter (in millimeter) for the permanent maxillary central incisors. The position of ThI-GH-SA26-88 as a central incisor is shown for information, supporting the hypothesis that this is likely not a central incisor.

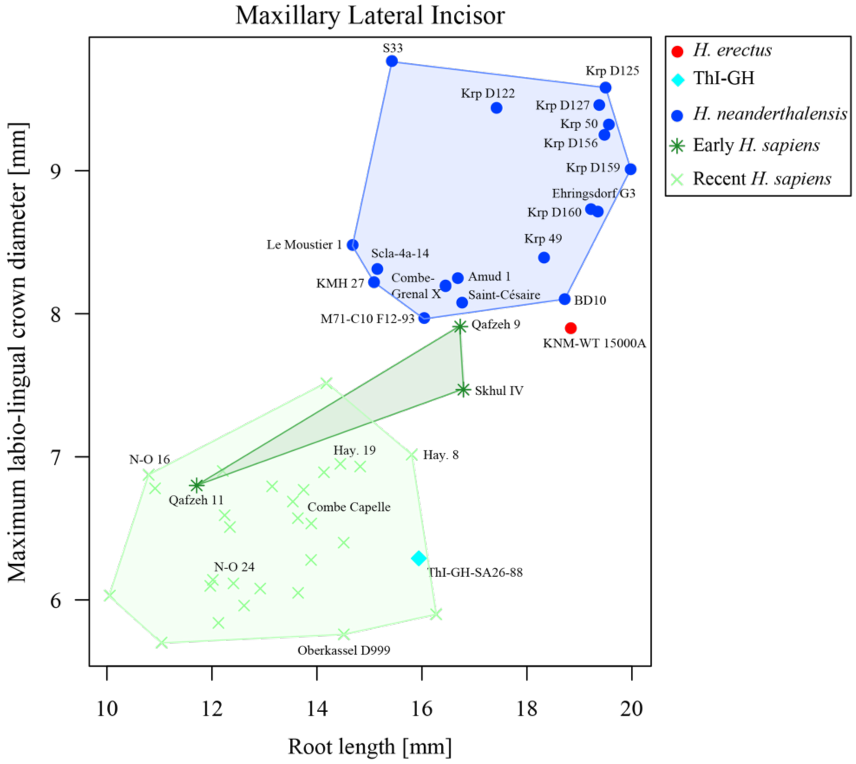

Figure 27. Bivariate plot of root length against labio-lingual crown diameter (in millimeter) for the permanent maxillary lateral incisors. The position of ThI-GH-SA26-88 is shown.

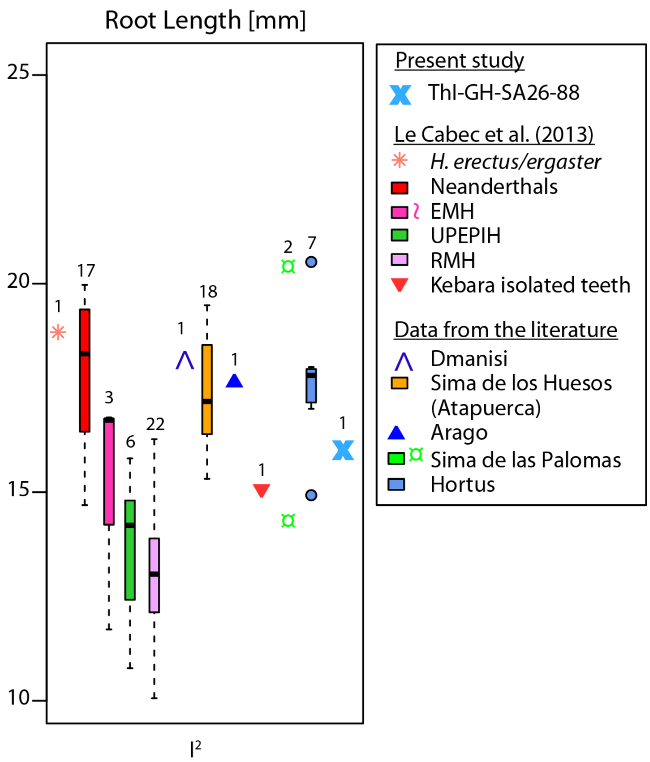

Figure 28. Comparison of the ThI-GH-SA26-88 maxillary lateral incisor with root length collected (or measured on pictures) from the literature, for Early and Middle Pleistocene hominins. The figures above the boxplots are sample sizes. (Modified from [106])

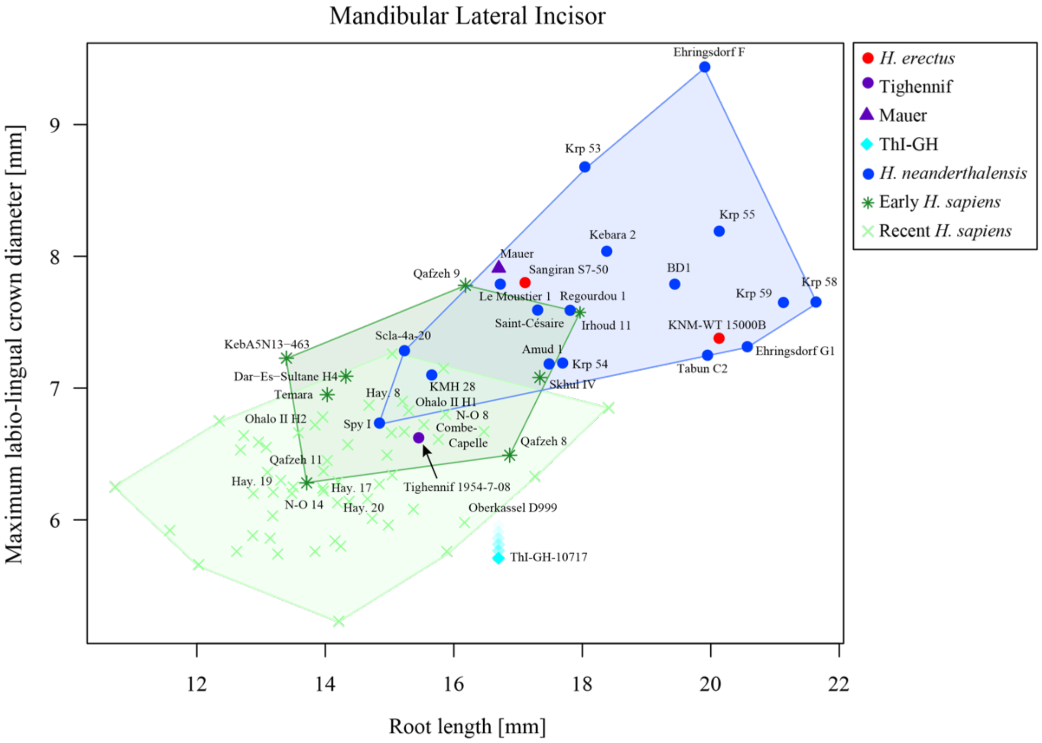

Figure 29. Bivariate plot of root length against labio-lingual crown diameter (in millimeter) for the permanent mandibular lateral incisors. The position of ThI-GH-10717 is shown. Since the crown of the ThI-GH-10717 LRI2 is heavily damaged, the uncertainty in the measurement of the labio-lingual crown diameter is estimated and shown with a gradient of transparency over turquoise diamonds spreading on the estimated range.

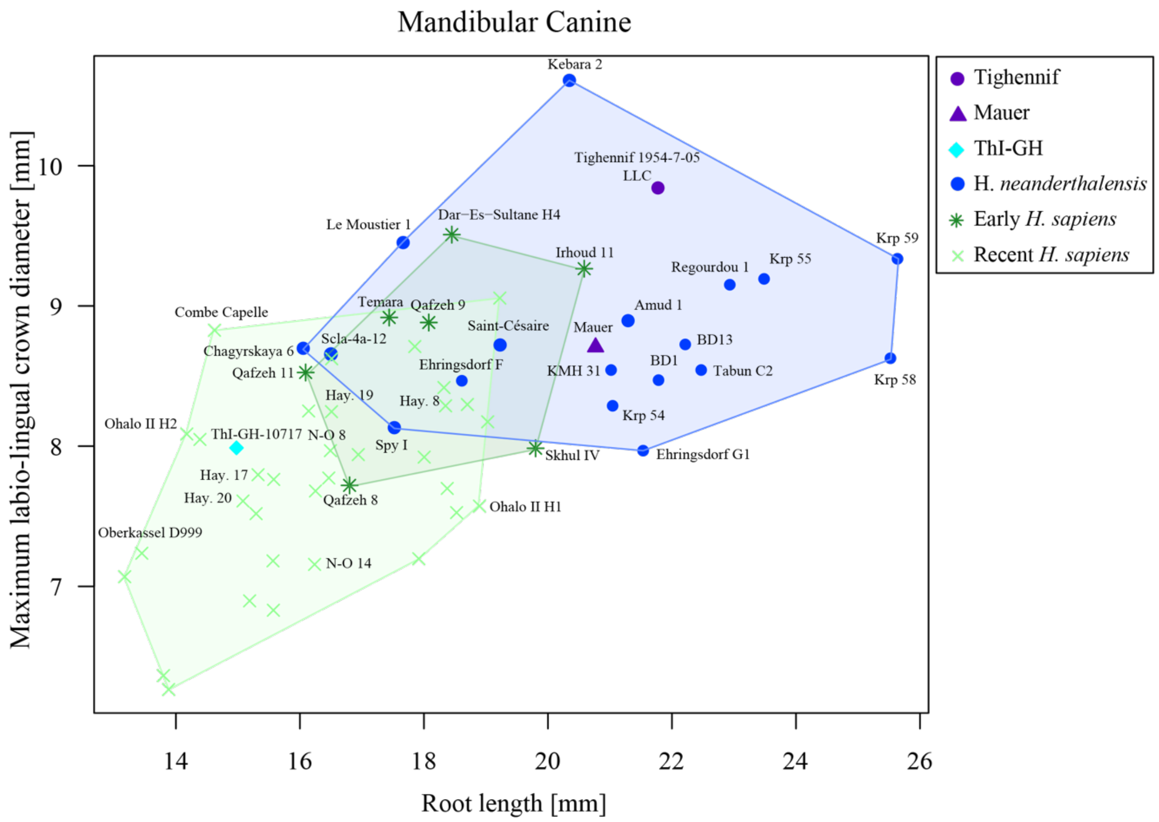

Figure 30. Bivariate plot of root length against labio-lingual crown diameter (in millimeter) for the permanent mandibular canines. The position of ThI-GH-10717 and Tighennif 1954-7-05 is shown.

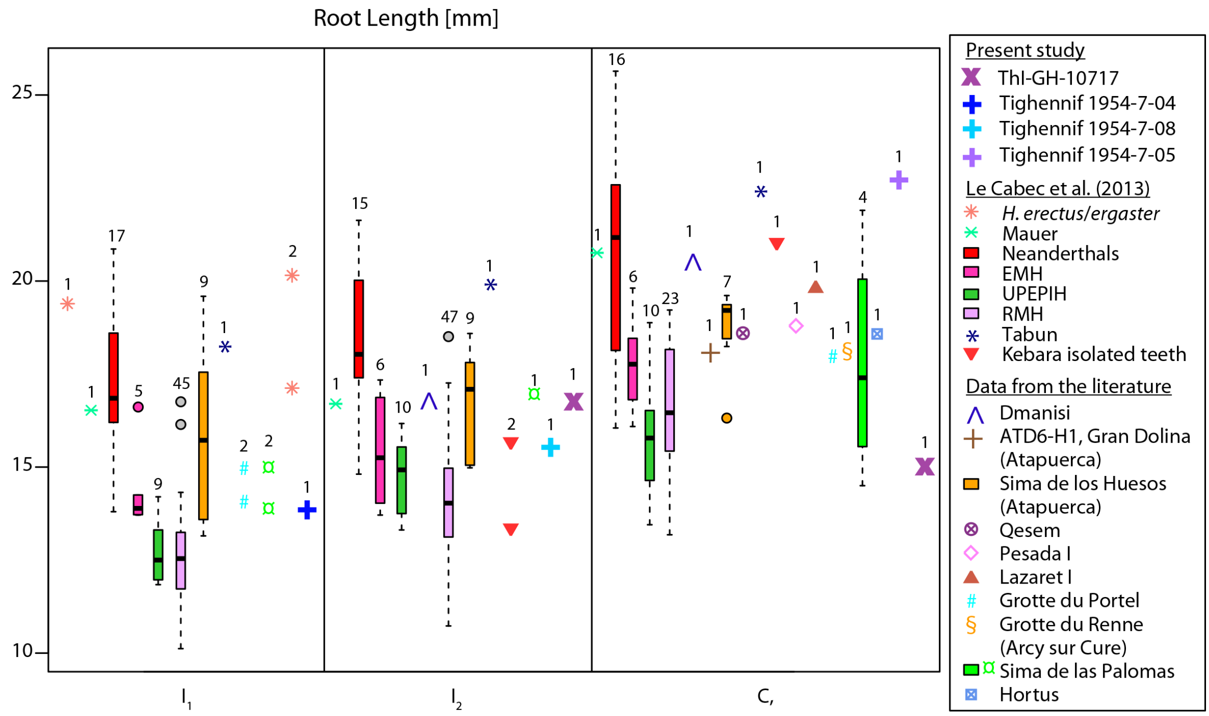

Figure 31. Comparison of the ThI-GH and Tighennif mandibular teeth with root length collected (or measured on pictures) from the literature, for Early and Middle Pleistocene hominins. The figures above the boxplots are sample sizes. (Modified from[106])

Supplementary Method 1 : Landmarks and curves semilandmarks used in the mandibular geometric morphometric analyses

Sarah Freidline and Inga Bergmann

Midline landmarks (i.e., non-bilateral) italicized. One end of curve defined with curve semilandmark and set as a true landmark when sliding.

**Landmark/Semilandmark**	**Definition**
**Mandible Landmarks (n = 301)**	
Gnathion	The most basal point on the symphysis
Infradentale	The midpoint between both central incisors sockets at the external side of the alveolar margin
Linguale	The most superior point of symphysis at the lingual side
Mentale	The most inferior point on the margin of the mental foramen
Mandibular Foramen	The most inferior point on the margin of the mandibular foramen
Condylion laterale	The most lateral point on the mandibular condyle
Condylion mediale	The most medial point on the mandibular condyle
Condylion superior	The most superior point on the mandibular condyle
Coronion	The most superior point on the coronoid process
I_1, I_2, C_1, P_3, P_4, M_1,M_2, M_3	The midpoint between the tooth and its distal neighbor at the external side of the alveolar margin and the distobuccal corner of the molar
**Mandible Curve Semilandmarks**	
Anterior symphysis (n = 10)	Anterior symphysis at midsagittal plane from infradentale to gnathion
Posterior symphysis (n = 10)	Posterior symphysis at midsagittal plane from lingaule to gnathion
Anterior ramus (n = 20)	Along anterior ramus from coronoid to distobuccal corner of M_2
Lingual alveolar margin (n = 30)	Superior alveolar margin on lingual side from linguale to coronoid
Mandibular notch (n = 15)	Along notch from coronoid to most superior point of condyle
Lateral condyle (n = 5)	Superior plane of the condyle from the most lateral to the most superior point.
Medial condyle (n=5)	Superior plane of the condyle from the most superior point to the most medial point
Posterior ramus (n = 50)	Along posterior ramus and base of mandible from gnathion to most superior point of condyle
**Corpus Landmarks (n = 153)**	
Gnathion	See above
Infradentale	See above
Linguale	See above
Mentale	See above
Mandibular Foramen	See above
I_1, I_2, C_1, P_3, P_4, M_1,M_2	See above

**Corpus Curve Semilandmarks**	
Anterior symphysis (n = 10)	See above
Posterior symphysis (n = 10)	See above
Anterior ramus[1] (n = 11)	Along anterior ramus from distobuccal corner of M_2 to 12th curve semilandmark (originally to coronoid)
Lingual alveolar margin (n = 22)	Superior alveolar margin on lingual side from linguale to 23rd curve semilandmark (originally to coronoid)
Posterior ramus (n = 21)	Along posterior ramus and base of mandible from gnathion to 22nd curve semilandmark (orginally superior condyle)
**Left Mandible Landmarks (n = 87)**	
C_1, P_3, P_4, M_1,M_2, M_3	See above
Lingual canine margin	Base of canine crown at lingual alveolar margin
Mandibular Foramen	See above
**Left Mandible Curve Semilandmarks**	
Anterior ramus (n = 16)	Along anterior ramus from distobuccal corner of M_2 to 17th curve semilandmark (originally to coronoid)
Lingual alveolar margin (n = 21)	Superior alveolar margin on lingual side from lingual canine margin to 22nd curve semilandmark (originally coronoid)
Posterior ramus (n = 38)	Along posterior ramus and base of mandible from 5th curve semilandmark to 40th curve semilandmark (originally from gnathion to most superior point of condyle)

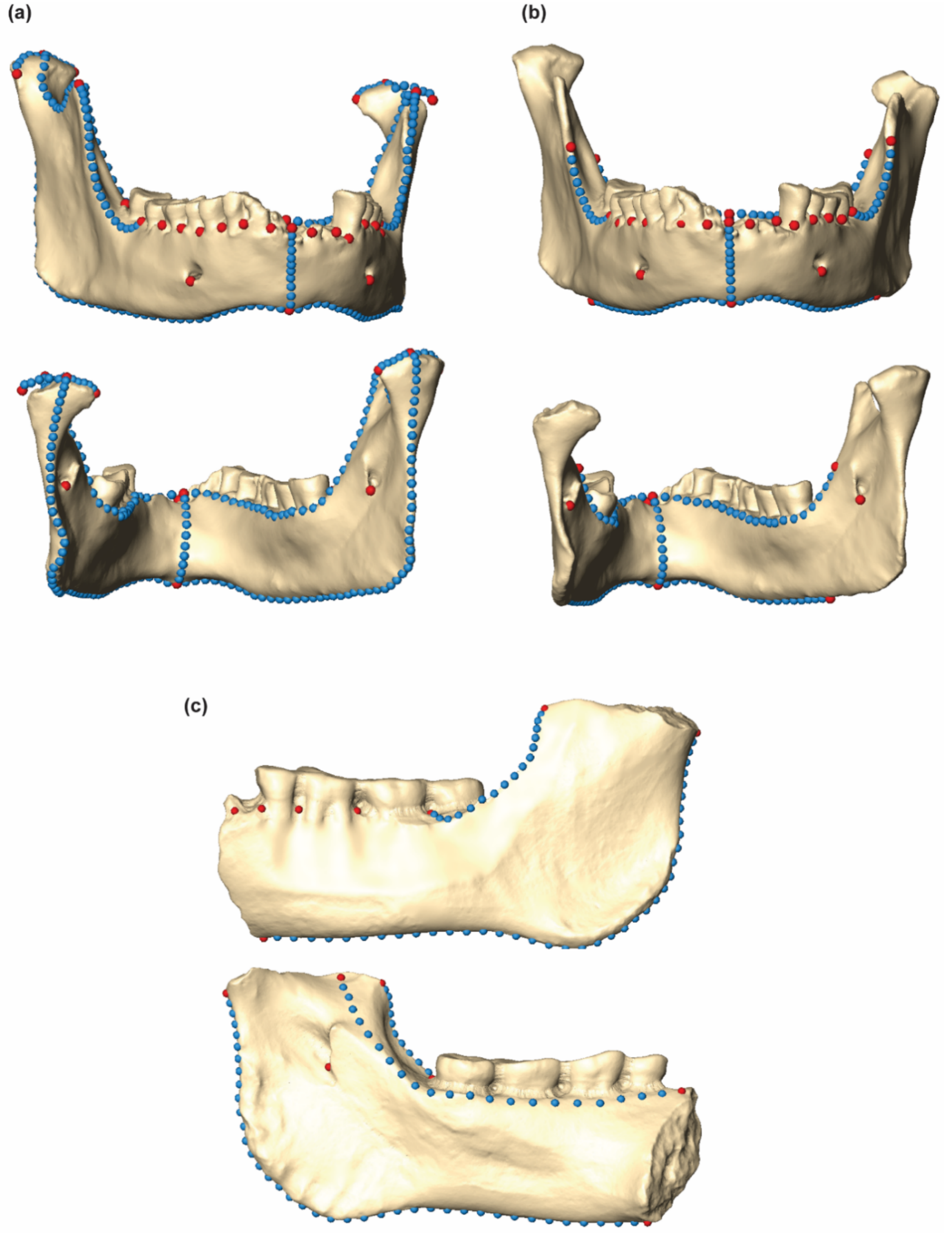

Figure 32. Landmarks (red) and semilandmarks (blue) used in the geometric morphometric analyses. (a) mandible on ThI-GH-10717; (b) corpus on ThI-GH-10717; (c) left mandible on ThI-GH-1. See Supplementary Table 2 for landmark and curve semilandmark definitions.

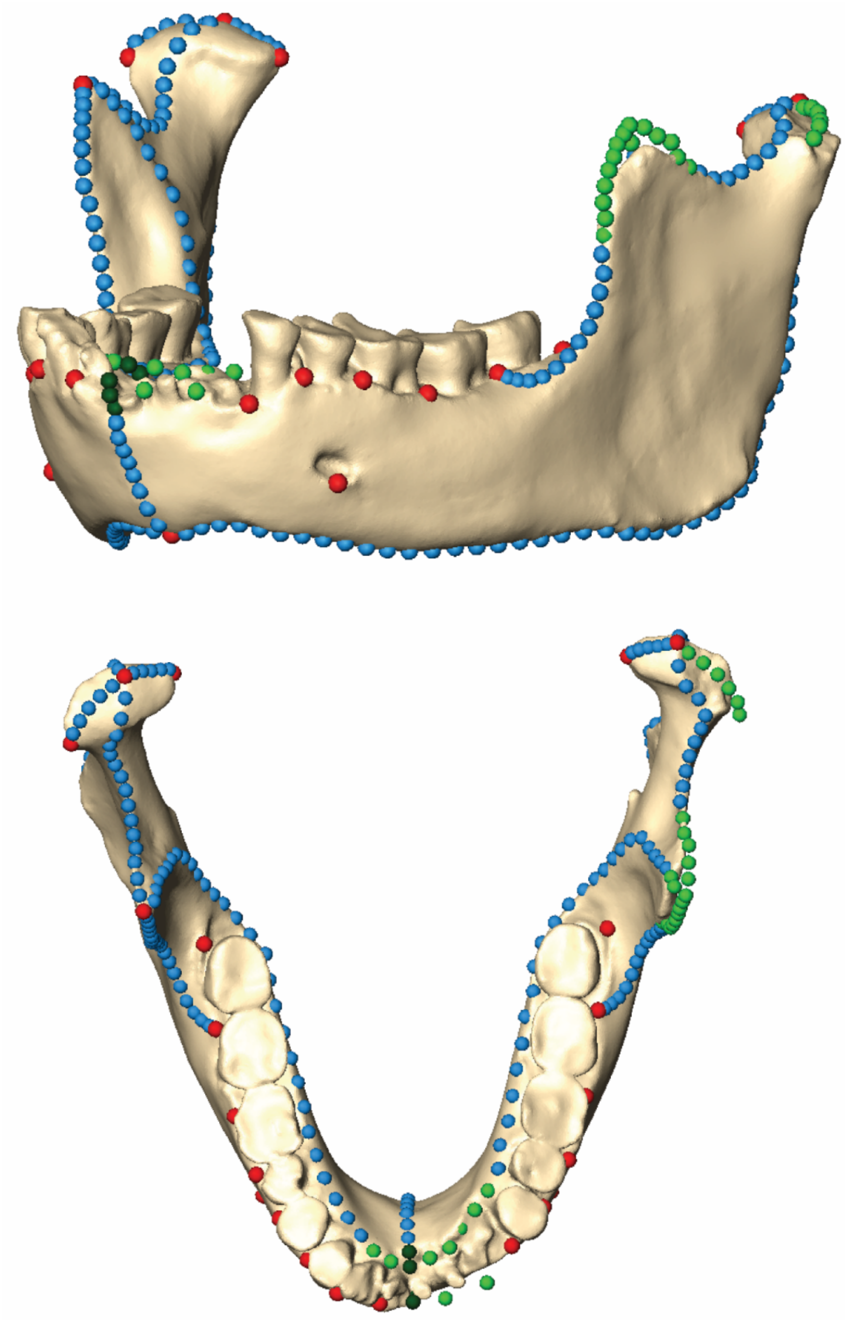

Figure 33. Estimated missing landmarks on ThI-GH-10717. Missing bilateral landmarks estimated by mirroring preserved side in light green and missing midline landmarks estimated by thin-plate spline deformation between it and the sample mean in dark green.

Supplementary Table 16: Magnetic component data of the studied samples

S. Perini and G. Muttoni

Sample: sample ID; **Reference**: magnetostratigraphic data from[10] and this study; **Depth**: [m]; **Comment**: [turning S quadrant=t.S.; not interpreted=n.i.; hand-sample=h.s]; **LT**: minimum temperature of the ChRM component [°C]; HT: maximum temperature of the ChRM component [°C]; **ChRM Dec**: declination of the ChRM component [°E]; **ChRM Inc**: inclination of the ChRM component [°]; **MAD**: maximum angular deviation of the ChRM component [°]; **VGP**: latitude of the virtual geomagnetic pole [°]; **GC pole Dec**: great cyrcle pole declination [°E]; **GC pole Inc**: great cyrcle pole inclination [°]

OH3A Member (Fig.1 B-C)

Sample	Reference	Depth [m]	Comment	LT [°C]	HT [°C]	ChRM Dec [°E]	ChRM Inc [°]	MAD [°]	VGP [°]	GC pole Dec (°E)	GC pole Inc (°)
3OH28	10	-1.734	n.i.								
3OH51	10	-1.523	n.i.								
3OH94	10	-1.111	n.i.								
3OH121	10	-0.879		250	625	161.5	-23.9	16.6	-63.0		
3OH144	10	-1.59	n.i.								
3OH172	10	-1.413	n.i.								
3OH212	10	-1.19		200	575	240.1	-5.1	7.5	-26.1		
3OH229	10	-1.011	n.i.								
3OH246	10	-0.742		200	625	183.2	25.3	19.6	-43.1		
3OH308	this study	-0.122	n.i.								
3OH313	10	-0.39		100	300	145.2	14.0	8.0	-37.6		
3OH339	10	-0.075	n.i.								
3OH353	this study	0.065	n.i.								
3OH376	this study	0.194		100	690	331.3	-41.1	14.5	26.6		
3OH377	this study	0.204		150	690	334.0	-25.7	14.5	36.8		
3OH379	10	0.21	n.i.								
3OH385	this study	0.27	n.i.								
3OH396	this study	0.359		600	690	313.6	0.5	20.0	35.3		
3OH401	this study	0.274	n.i.								
3OH405	10	0.246		100	675	37.0	42.9	6.2	56.8		
3OH412	this study	0.321	n.i.								
30H448	this study	-0.083		150	525	334.5	27.1	18.8	59.9		
3OH462	10	0.044		150	650	357.6	-17.1	10.4	47.6		
3OH471	this study	0.119		150	525	1.1	19.8	8.0	66.6		
3OH478	this study	0.008		150	600	332.8	19.7	10.7	55.7		
3OH490	10	0.108		100	575	313.9	30.7	5.7	45.4		
3OH493	this study	0.112									
3OH512	this study	0.108									
3OH539	this study	0.594		150	680	339.0	-16.0	21.8	43.8		

OH3B Member (Fig.1 B-C)

Sample	Reference	Depth [m]	Comment	LT [°C]	HT [°C]	ChRM Dec [°E]	ChRM Inc [°]	MAD [°]	VGP [°]	GC pole Dec (°E)	GC pole Inc (°)
3BOH30	10	3.131	n.i.								
3BOH57	10	3.36	n.i.								
3BOH94	10	3.51	n.i.								
3BOH136	10	3.664	n.i.								
3BOH178	10	3.855	n.i.								
3BOH221	10	3.56	n.i.								
3BOH281	10	3.44	n.i.								
3BOH308	10	3.562		150	425	210.5	-3.4	24.0	-47.2		
3BOH388	10	4.362		200	675	208.6	42.1	10.5	-26.0		
3BOH428	10	4.762	n.i.								
3BOH463	10	5.112	n.i.								
3BOH486	10	5.342	n.i.								
3BOH510	10	8.582	n.i.								

OH4 Member (Fig.1 B-C)

Sample	Reference	Depth [m]	Comment	LT [°C]	HT [°C]	ChRM Dec [°E]	ChRM Inc [°]	MAD [°]	VGP [°]	GC pole Dec (°E)	GC pole Inc (°)
4OH8	10	-7.166		250	675	178.6	-33.9	8.2	-74.9		
4OH29	10	-6.949		350	625	184.5	-49.2	3.1	-84.8		
4OH59	10	-6.656		350	675	191.4	-26.9	3.6	-68.1		
oh4_7	10		h.s.	325	675	182.1	-29.6	6.9	-72.2		
4OH96	10	-6.329		300	650	192.5	-23.5	4.3	-65.8		
4OH116	10	-6.131		300	675	217.8	-43.5	8.8	-56.3		
4OH145	10	-5.843		300	675	198.6	-16.8	8.7	-59.7		
oh4_8	10		h.s.	300	675	181.1	-30.2	5.5	-72.6		
4OH166	10	-5.671		350	675	192.0	-21.1	5.9	-64.8		
4OH198	10	-5.35		300	625	200.1	-18.9	6.8	-59.9		
oh4_9	10		h.s.	325	675	179.2	-9.4	9.0	-61.2		
4OH237	10	-4.977		300	675	220.2	-46.7	7.2	-55.3		
4OH263	10	-4.642		350	675	162.7	4.2	7.3	-50.8		
4OH303	10	-4.173		400	650	222.8	-43.7	15.0	-52.2		
4OH363	10	-3.997	n.i.								
4OH403	10	-3.552		300	625	240.6	-19.4	6.2	-29.9		
4OH433	10	-3.171		400	675	164.2	-55.6	7.1	-76.8		
4OH443	10	-3.086		200	475	216.1	-59.1	14.5	-60.6		
4OH454	10	-2.933		425	650	242.2	-2.8	9.1	-23.7		
4OH483	10	-2.622	t.S.								
4OH484	10	-2.632		350	650	173.0	-53.8	10.1	-84.1		

GH-CCC/OH4 SU 6-3: section A (Fig. 1 C-D; ED Fig. 2B)

Sample	Reference	Depth [m]	Comment	LT [°C]	HT [°C]	ChRM Dec [°E]	ChRM Inc [°]	MAD [°]	VGP [°]	GC pole Dec (°E)	GC pole Inc (°)
4OH485	this study	-2.619		325	670	221.9	-34.0	23.8	-49.8		
4OH489	this study	-2.582		400	690	186.5	-48.8	6.8	-83.3		
4OH491	this study	-2.562		325	680	3.9	29.5	10.9	71.9		
4OH493	this study	-2.551		350	680	347.7	48.24	17.94	78.71		
4OH495	this study	-2.523		325	650	26.5	-28.8	12.9	34.9		
4OH497	this study	-2.492		325	690	48.8	11.6	8.4	37.0		
4OH498	this study	-2.478		275	690	236.7	22.0	7.7	-19.8		
4OH503	this study	-2.441		325	670	173.1	-49.3	16.4	-83.2		
4OH505	this study	-2.415	n.i.								
4OH508	this study	-2.388		325	690	234.6	-60.8	7.7	-46.8		
4OH508	10	-2.388		450	650	201.0	-16.0	3.1	-58.1		
4OH510	this study	-2.354		275	680	201.7	7.7	9.6	-47.3		
4OH512.5	this study	-2.332	t.S.							226.4	-1.7
4OH514	this study	-2.319		400	680	230.6	6.7	10.3	-29.7		
4OH516.5	this study	-2.290	t.S.							62.0	-64.4
4OH518	this study	-2.279		350	650	250.2	-1.0	6.9	-16.7		
4OH521.5	this study	-2.240	t.S.							228.6	10.4
4OH525	this study	-2.208		375	690	187.0	19.0	6.4	-46.2		
4OH528	10	-2.177	n.i.								
4OH532	this study	-2.140		375	670	167.3	-55.4	15.7	-79.3		
4OH532.5	this study	-2.123		300	680	153.5	-17.8	19.5	-55.5		
4OH536	this study	-2.090		325	670	129.0	-35.1	8.7	-42.7		
4OH539	this study	-2.059	t.S.							86.4	16.4
4OH543.5	this study	2.018		325	660	135.7	-65.3	10.9	-54.2		
4OH545	this study	-1.990		350	680	175.9	12.3	12.0	-50.1		
4OH548	10	-1.971	n.i.								
4OH552	this study	-1.937	t.S.							236.7	-13.4
4OH557	this study	-1.880	t.S.							7.1	-54.7
4OH561	this study	-1.842	t.S.							36.4	76.3
4OH564	this study	-1.810	t.S.							117.2	46.3
4OH566	this study	-1.789		275	660	316.5	-11.7	15.5	33.0		
4OH570	this study	-1.749		325	680	229.0	-43.1	11.0	-46.9		
4OH574	this study	-1.710		450	690	173.6	6.1	9.4	-52.9		
4OH575	this study	-1.700	n.i.							38.3	29.9
4OH578	10	-1.669	n.i.							206.0	-62.0
4OH583	this study	-1.620		325	660	77.2	3.7	6.4	11.7		
4OH588	this study	-1.570		275	660	315.8	6.8	11.7	39.0		
4OH590	this study	-1.530		325	660	35.0	21.3	10.3	50.8		

GH-CCC/OH4 SU 6-3: section B (Fig. 1 C-D, E.D Fig. 2B)

Sample	Reference	Depth [m]	Comment	LT [°C]	HT [°C]	ChRM Dec [°E]	ChRM Inc [°]	MAD [°]	VGP [°]	GC pole Dec (°E)	GC pole Inc (°)
B03	this study	-2.608		450	690	199.3	-23.4	12.6	-62.3		
B06	this study	-2.589		400	690	344.2	65.5	17.4	71.6		
B08	this study	-2.560	n.i.								
B12	this study	-2.523	n.i.								
B13	this study	-2.515		400	690	334.7	-15.6	18.2	42.0		
B15	this study	-2.499		350	690	227.2	13.2	18.0	-29.9		
B17.5	this study	-2.480		400	690	114.6	-7.6	12.6	-22.5		
B18	this study	-2.469		350	690	144.4	-39.7	7.0	-56.9		
B21	this study	-2.438	t.S.							77.5	28.8
B24	this study	-2.418	t.S.							228.9	28.2
B26.5	this study	-2.391		575	675	228.3	3.4	49.0	-32.5		
B27.5	this study	-2.378	t.S.							247.1	24.3
B29	this study	-2.362	t.S.							211.2	34.9
B33	this study	-2.322	t.S.							240.2	-1.5
B36	this study	-2.295	t.S.							137.6	61.6
B42	this study	-2.254	t.S.							201.0	45.8
B45	this study	-2.225	t.S.							284.4	-37.1
B49	this study	-2.188	t.S.								
B53.5	this study	-2.140	n.i.								
B57.5	this study	-2.098		500	690	163.7	-27.0	9.5	-65.7		
B59.5	this study	-2.079	t.S.							266.3	-17.4
B62.5	this study	-2.050	t.S.							254.6	24.4
B65	this study	-2.023		300	690	332.5	21.7	8.2	56.4		
B68	this study	-1.994	t.S.							124.6	58.6
B70	this study	-1.971	t.S.							231.2	-37.7
B82	this study	-1.851	n.i.								
B86.5	this study	-1.806	t.S.							260.2	-13.2
B92.5	this study	-1.746		325	690	235.3	-63.1	11.8	-46.6		
B97	this study	-1.701	t.S.							265.0	-1.3
B100	this study	-1.671	t.S.							240.0	-36.3
B102	this study	-1.651		530	680	157.4	33.7	20.6	-33.7		
B103.5	this study	-1.636	t.S.							75.0	28.0
B105	this study	-1.621	t.S.							108.8	38.5
B113	this study	-1.541		250	680	350.0	38.0	14.2	74.9		

GH-CCC/OH4 SU 5-3: section C and D (Fig. 1 C-D; ED Fig. 2B)

Sample	Reference	Depth [m]	Comment	LT [°C]	HT [°C]	ChRM Dec [°E]	ChRM Inc [°]	MAD [°]	VGP [°]	GC pole Dec (°E)	GC pole Inc (°)
C03	this study	-2.569		500	690	163.7	-28.3	6.0	-66.4		
C06	this study	-2.543		400	690	180.1	-24.6	12.4	-69.3		
C10	this study	-2.511	t.S.							105.6	20.2
C14	this study	-2.479		300	690	350.2	57.1	13.0	81.0		
C17	this study	-2.449		350	650	356.8	20.8	29.4	67.0		
C20	this study	-2.419		300	690	26.0	28.0	8.7	60.0		
C24.5	this study	-2.377		550	690	127.9	-44.1	10.8	-44.5		
C28.5	this study	-2.344	t.S.							262.5	40.5
C32	this study	-2.311	t.S.							97.1	26.6
C36	this study	-2.268	t.S.							4.7	-56.2
C41	this study	-2.216		450	690	129.5	-34.6	27.6	-43.0		
C46	this study	-2.173	t.S.							104.1	14.5
C50	this study	-2.190		400	690	146.4	-11.6	14.8	-48.3		
C57	this study	-2.068		250	550	351.0	31.3	7.3	71.5		
D5.5	this study	-2.590		350	690	352.0	3.5	32.3	57.3		
D11	this study	-2.513		250	650	332.3	12.2	7.4	52.5		
D17.5	this study	-2.449	t.S.							226.9	42.6
D21	this study	-2.415	t.S.							230.4	48.4
D24	this study	-2.390	t.S.							146.3	52.1
D27	this study	-2.360	t.S.							257.4	0.3

GH-CCC/OH4 SU 6-4: section E (Fig. 1 C-D ; E.D. Fig. 2B)

Sample	Reference	Depth [m]	Comment	LT [°C]	HT [°C]	ChRM Dec [°E]	ChRM Inc [°]	MAD [°]	VGP [°]	GC pole Dec (°E)	GC pole Inc (°)
4OH458	this study	-2.790	t.S.							241.9	8.7
4OH462	this study	-2.750	n.i.								
4OH466.5	this study	-2.684	t.S.							245.9	3.4
4OH468	10	-2.710	n.i.								
4OH469.5	this study	-2.665	n.i.								
4OH470	this study	-2.686	t.S.							244.8	0.5
4OH472	this study	-2.628		500	680	196.8	1.0	24.0	-52.5		
4OH475	this study	-2.597	t.S.							291.3	33.2
G6	this study	-2.635	t.S.							100.7	3.3
G7	this study	-2.618		300	690	203.7	-7.2	9.2	-52.8		
G10	this study	-2.620	t.S.							260.2	18.5
G11	this study	-2.591		350	690	238.0	-22.4	10.5	-33.0		
G14	this study	-2.559	t.S.							264.5	2.3
G16	this study	-2.540		325	690	91.5	35.6	12.0	9.5		
G23.5	this study	-2.464		250	680	37.9	27.6	7.6	50.9		
G26	this study	-2.437		550	620	228.7	-13.2	3.7	-37.6		
G28	this study	-2.409	t.S.							102.1	17.1
G29	this study	-2.402	t.S.							335.0	-61.2
G33	this study	-2.363		510	690	213.0	-33.4	6.9	-56.8		
G37	this study	-2.314		250	685	18.75	44.28	34.25	72.09		

OH5 (Fig.1 B-C)

Sample	Reference	Depth [m]	Comment	LT [°C]	HT [°C]	ChRM Dec [°E]	ChRM Inc [°]	MAD [°]	VGP [°]	GC pole Dec (°E)	GC pole Inc (°)
5OH181	10	-1.346		350	600	292.5	25.2	16.5	25.9		
5OH215	10	-0.997		400	625	26.3	19.9	18.6	56.5		
5OH244	10	-0.897	n.i.								
5OH266	10	-0.677		300	625	318.1	60.8	20.1	56.2		
5OH291	10	-0.42	n.i.								
5OH326	10	-0.064		350	600	298.4	16.4	27.6	28.2		
5OH351	10	0.365		300	550	334.9	12.1	9.4	54.0		
5OH377	10	0.759		350	525	319.1	3.1	13.1	40.2		

Supplementary Table 17. Comparative Sample used in the Mandibular Study, including origin, groups affiliation and landmark dataset; underlined specimens were casts. Abbreviations: PS = photogrammetry scan; CT = computed tomography.

Sarah Freidline and Inga Bergmann

Specimen	Origin	Group Affiliation	Data	Scan
Dmanisi 211	Georgia	*Homo erectus*	Corpus	CT
Dmanisi 2735	Georgia	*Homo erectus*	Left Mandible, Corpus	CT
KNM-WT 15000**	Kenya	*Homo erectus*	Left Mandible, Mandible, Corpus	CT
Lantian	China	*Homo erectus*	Corpus	CT
Zhoukoudian LC G1.6	China	*Homo erectus*	Corpus	CT
Zhoukoudian LC G1/G2*	China	*Homo erectus*	Left Mandible, Mandible, Corpus	CT
Zhoukoudian LC H/1 (H1.12)	China	*Homo erectus*	Left Mandible, Mandible, Corpus	CT
Liang Bua 1	Indonesia	*Homo floresiensis*	Left Mandible, Mandible, Corpus	CT
Lesedi 1	South Africa	*Homo naledi*	Left Mandible, Corpus	CT
Dinaledi Hominin 1	South Africa	*Homo naledi*	Corpus	CT
Baringo Kapthurin 67 (KNM-BK)	Kenya	African Early Pleistocene	Left Mandible, Mandible, Corpus	CT
Baringo Kapthurin 8518 (KNM-BK)	Kenya	African Early Pleistocene	Left Mandible, Corpus	CT
Thomas Quarry 1	Morocco	African Early Pleistocene	Left Mandible	CT
Thomas Quarry 1 Gh10717	Morocco	African Early Pleistocene	Left Mandible, Mandible, Corpus	CT
Tighennif 1	Algeria	African Early Pleistocene	Corpus	CT
Tighennif 2	Algeria	African Early Pleistocene	Left Mandible, Mandible, Corpus	PS
Tighennif 3	Algeria	African Early Pleistocene	Left Mandible, Mandible, Corpus	PS
Arago II	France	European Middle Pleistocene	Left Mandible, Mandible, Corpus	PS
Arago XIII	France	European Middle Pleistocene	Left Mandible	PS
AT-250/AT-793 (individual IV)	Spain	European Middle Pleistocene	Corpus	PS
AT-888/721/776 (ind. XXI/cran. V)	Spain	European Middle Pleistocene	Left Mandible, Mandible, Corpus	PS
AT-950 (individual XXVIII)	Spain	European Middle Pleistocene	Left Mandible, Mandible, Corpus	PS

AT-952/AT-505/ AT-604 (ind. XIX)	Spain	European Middle Pleistocene	Left Mandible, Mandible, Corpus	PS
Mauer	Germany	European Middle Pleistocene	Left Mandible, Mandible, Corpus	CT
Montmaurin	France	European Middle Pleistocene	Left Mandible, Mandible, Corpus	PS
Xiahe	China	Denisovan	Corpus	CT
Amud 1	Israel	Neanderthal	Left Mandible, Mandible, Corpus	CT
Guattari 2	Italy	Neanderthal	Corpus	CT
Guattari 3	Italy	Neanderthal	Left Mandible, Corpus	CT
Kebara 2	Israel	Neanderthal	Left Mandible, Corpus	CT
Krapina 58	Croatia	Neanderthal	Corpus	CT
Krapina 59	Croatia	Neanderthal	Left Mandible, Mandible	CT
La Chaise: Bourgeois-Delaunay 1	France	Neanderthal	Left Mandible, Mandible, Corpus	CT
La Ferrassie 1	France	Neanderthal	Left Mandible, Mandible, Corpus	CT
La Quina H5	France	Neanderthal	Left Mandible, Corpus	CT
La Quina H9	France	Neanderthal	Left Mandible, Corpus	PS
Regourdou 1	France	Neanderthal	Left Mandible, Mandible, Corpus	CT
Spy 3	Belgium	Neanderthal	Left Mandible, Mandible, Corpus	CT
Tabun C1	Israel	Neanderthal	Left Mandible, Mandible, Corpus	PS
Vindija 11.4	Croatia	Neanderthal	Left Mandible, Mandible, Corpus	CT
Zafarraya 2	Spain	Neanderthal	Left Mandible, Mandible, Corpus	CT
El Sidrón 1	Spain	Neanderthal	Mandible, Corpus	CT
El Sidrón 2	Spain	Neanderthal	Mandible, Corpus	CT
Border Cave 5	South Africa	early *H. sapiens*	Left Mandible, Mandible, Corpus	PS
Contrebandiers 1	Morocco	early *H. sapiens*	Left Mandible, Mandible, Anterior Corpus	CT
Dar es-Soltane H5	Morocco	early *H. sapiens*	Left Mandible, Mandible, Corpus	CT
Jebel Irhoud 11	Morocco	early *H. sapiens*	Left Mandible, Mandible, Corpus	CT
Klasies River Mouth 41815	South Africa	early *H. sapiens*	Left Mandible, Corpus	PS
Qafzeh 9 + 25	Israel	early *H. sapiens*	Left Mandible, Mandible, Corpus	CT
Skhul 4	Israel	early *H. sapiens*	Left Mandible, Mandible, Corpus	CT
Skhul 5	Israel	early *H. sapiens*	Left Mandible, Mandible, Corpus	CT

Tabun C2	Israel	early *H. sapiens*	Left Mandible, Mandible, Corpus	CT
Afalou (n = 8)	Algeria	Late Pleistocene *H. sapiens*	Left Mandible, Mandible, Corpus	PS
El Harhoura 1	Morocco	Late Pleistocene *H. sapiens*	Corpus	CT
Hayonim (*n* = 5)	Israel	Late Pleistocene *H. sapiens*	Left Mandible, Mandible, Corpus	CT/PS
Mumbwa 3	Zambia	Late Pleistocene *H. sapiens*	Left Mandible, Corpus	PS
Nahal Oren (*n* = 4)	Israel	Late Pleistocene *H. sapiens*	Left Mandible, Mandible, Corpus	CT/PS
Nazlet Khater 2	Egypt	Late Pleistocene *H. sapiens*	Left Mandible, Mandible, Corpus	CT
Olduvai 1	Tanzania	Late Pleistocene *H. sapiens*	Left Mandible, Mandible, Corpus	PS
Taforalt (*n* = 9)	Morocco	Late Pleistocene *H. sapiens*	Left Mandible, Mandible, Corpus	PS
Asselar	Mali	Early Holocene *H. sapiens*	Left Mandible, Mandible, Corpus	PS
Lothagam KNM-ER5306	Kenya	Early Holocene *H. sapiens*	Left Mandible, Mandible, Corpus	CT
Lothagam KNM-LT13702	Kenya	Early Holocene *H. sapiens*	Left Mandible, Mandible, Corpus	CT

*Reconstruction by[101] based on Zhoukoudian LC G1.6
**Non-adult

Supplementary Table 18: Procrustes Distances of ThI-GH-10717 Mandibular Shape.

Sarah Freidline and Inga Bergmann

ThI-GH-10717	PD
Border Cave 5	0.0765
Montmaurin	0.0767
Mauer	0.0790
SH XXVIII	0.0818
Tighennif 2	0.0854
Afalou 28	0.0860
KNM-WT 15000	0.0883
Zhoukoudian LC H/1 (H1.12)	0.0886
Nazlet Khater 2	0.0888
Nahal Oren 6	0.0926
Zafarraya 2	0.0933
Liang Bua 1	0.0934
Hayonim 19A	0.0942
El Sidrón 1	0.0949
Nahal_Oren 8	0.0955
Skhul 5	0.0957
Olduvai 1	0.0958
Taforalt VIII	0.0960
Krapina 59	0.0972
Lothagam KNM-ER5306	0.0976
Dmanisi 2735	0.0976
Taforalt XIX	0.0985
Taforalt XVIII	0.0990
Hayonim 20	0.0998
Taforalt XXV	0.1002
Skhul 4	0.1007
Spy 3	0.1007
Jebel Irhoud 11	0.1021
Taforalt I	0.1026
Nahal Oren 14	0.1030
Contrebandiers 1	0.1067
Taforalt XX	0.1072
Zhoukoudian LC G1/G2	0.1079
Hayonim 29	0.1082
Asselar	0.1086
Tabun C1	0.1095
Tabun C2	0.1108
Taforalt XII	0.1118
Lothagam KNM-LT13702	0.1119
SH XIX	0.1153
Afalou 48	0.1171

Afalou 2	0.1172
Hayonim 27	0.1178
Baringo Kapthurin 67 (KNM-BK)	0.1187
Tighenif 3	0.1189
Nahal Oren 18	0.1196
La Ferrassie 1	0.1202
SH XXI	0.1215
Arago II	0.1220
Amud 1	0.1227
Hayonim 17	0.1253
Afalou 5	0.1265
Afalou 3	0.1269
Qafzeh 9 + 25	0.1274
El Sidrón 2	0.1300
Taforalt XXVII	0.1301
Dar es-Soltane H5	0.1304
Afalou B4	0.1321
Afalou 10	0.1323
Regourdou 1	0.1352
Taforalt XXV	0.1376
Vindija 11.4	0.1544
Afalou 46	0.1616
La Chaise: Bourgeois-Delaunay 1	0.1665
La Quina H5	0.1697

Supplementary Table 19: Procrustes distances of ThI-GH-10717 and ThI-GH-1 left mandible shape.

Sarah Freidline and Inga Bergmann

ThI-GH-10717	PD	ThI-GH-1	PD
Mumbwa 3	0.0974	Lothagam KNM-ER5306	0.1173
Montmaurin	0.1014	Taforalt XXVII	0.1174
Zhoukoudian LC H/1 (H1.12)	0.1086	Taforalt XXV	0.1228
Tabun C1	0.1106	KNM-WT 15000	0.1253
SH XXVIII	0.1113	Nahal Oren 14	0.1320
ThI-GH-1	0.1174	Lothagam KNM-LT13702	0.1343
La Quina H9	0.1177	El Sidrón 1	0.1379
Taforalt XX	0.1185	La Ferrassie 1	0.1388
Nahal_Oren 8	0.1190	Skhul 4	0.1422
Mauer	0.1190	Arago XIII	0.1423
Contrebandiers 1	0.1218	SH XIX	0.1441
Arago II	0.1253	Taforalt XIX	0.1500
Hayonim 20	0.1263	Contrebandiers 1	0.1526
Dmanisi 2735	0.1290	Nazlet Khater 2	0.1528
Krapina 59	0.1296	Regourdou 1	0.1530
Lothagam KNM-ER5306	0.1297	Guattari 3	0.1541
Nahal Oren 18	0.1308	Afalou 48	0.1548
Hayonim 19A	0.1318	Zhoukoudian LC H/1 (H1.12)	0.1558
Zafarraya 2	0.1331	Skhul 5	0.1563
Nahal Oren 14	0.1334	Zafarraya 2	0.1592
SH XIX	0.1340	SH XXVIII	0.1594
El Sidrón 1	0.1347	Zhoukoudian LC G1/G2	0.1597
Tabun C2	0.1350	Krapina 59	0.1598
Taforalt I	0.1358	Nahal_Oren 6	0.1607
Afalou 28	0.1361	Tighennif 3	0.1612
Taforalt VIII	0.1364	Afalou 28	0.1631
Hayonim 17	0.1367	Amud 1	0.1633
La Quina H5	0.1370	Spy 3	0.1634
Afalou 48	0.1382	Olduvai 1	0.1646
Baringo Kapthurin 67 (KNM-BK)	0.1396	La Chaise: Bourgeois-Delaunay 1	0.1657
Dar es-Soltane H5	0.1400	Afalou 2	0.1661
Klasies River Mouth 41815	0.1415	El Sidrón 2	0.1665
Nahal_Oren 6	0.1415	Dmanisi 2735	0.1666
Regourdou 1	0.1422	Nahal_Oren 8	0.1684
Spy 3	0.1423	Asselar	0.1688
La Ferrassie 1	0.1425	Mumbwa 3	0.1691
Vindija 11.4	0.1428	Hayonim 19A	0.1693
Hayonim 29	0.1430	Taforalt XII	0.1704
Afalou 2	0.1442	Kebara 2	0.1725
Taforalt XVIII	0.1442	Border Cave 5	0.1726

Olduvai 1	0.1443	Lesedi 1	0.1766
Skhul 5	0.1448	Tabun C1	0.1772
Hayonim 27	0.1450	Liang Bua 1	0.1785
Guattari 3	0.1456	Qafzeh 9 + 25	0.1785
Tighennif 2	0.1466	Afalou 5	0.1794
Qafzeh 9 + 25	0.1481	Arago II	0.1797
Jebel Irhoud 11	0.1486	Taforalt VIII	0.1799
Baringo Kapthurin 8518 (KNM-BK)	0.1488	Mauer	0.1814
Lothagam KNM-LT13702	0.1508	Afalou 10	0.1826
Nazlet Khater 2	0.1528	Tighennif 2	0.1830
Asselar	0.1529	Tabun C2	0.1833
Taforalt XIX	0.1531	ThI-GH-1	0.1846
Border Cave 5	0.1531	SH XXI	0.1861
Zhoukoudian LC G1/G2	0.1535	Hayonim 20	0.1864
Arago XIII	0.1555	Hayonim 17	0.1879
Skhul 4	0.1581	Afalou 3	0.1880
Taforalt XXV	0.1605	Taforalt XVIII	0.1882
Lesedi 1	0.1613	Klasies River Mouth 41815	0.1883
El Sidrón 2	0.1621	Montmaurin	0.1886
Afalou 5	0.1637	Afalou B4	0.1891
La Chaise: Bourgeois-Delaunay 1	0.1645	La Quina H9	0.1912
Kebara 2	0.1684	Hayonim 29	0.1913
Amud 1	0.1691	Taforalt XXV	0.1938
Taforalt XXV	0.1696	Hayonim 27	0.1947
Liang Bua 1	0.1708	La Quina H5	0.1953
SH XXI	0.1709	Vindija 11.4	0.1969
Taforalt XII	0.1710	Taforalt I	0.1988
Tighenif 3	0.1711	Baringo Kapthurin 67 (KNM-BK)	0.1991
KNM-WT 15000	0.1739	Baringo Kapthurin 8518 (KNM-BK)	0.1999
Afalou 10	0.1742	Dar es-Soltane H5	0.2023
Afalou B4	0.1869	Jebel Irhoud 11	0.2083
Afalou 3	0.1914	Nahal Oren 18	0.2179
Taforalt XXVII	0.1941	Taforalt XX	0.2187
Afalou 46	0.2259	Afalou 46	0.2319

Supplementary Table 20: Non-metric Dental Traits

Shara E. Bailey

Frequencies of trait presence (grade = trait presence given in first column). P4: fourth premolar, M1: first molar, M2: second molar, M3: third molar. Numbers of individuals between brackets.

	ThI-GH	Tighennif	Sidi Abderrahmane	Middle Pleistocene Africa	Jebel Irhoud	*Homo erectus s.l.*	*H. antecessor*	Middle Pleistocene Europe	Neanderthals	Early *H. sapiens*	Recent (Upper Paleolithic + extant)
P^4 Maxillary premolar accessory ridges (MxPAR)[112] presence ≥ 2	100 (1)	–	–	100 (1)	–	66.7 (3)	–	50.0 (2)	63.2 (19)	0.0 (4)	29.4 (170)
P_4 Transverse crest[113] presence ≥ 2	100 (1)	100 (2)	100 (1)	100 (4)	0.0 (2)	36.4 (11)	–	25.0 (4)	74.2 (31)	11.1 (9)	6.1 (197)
P_4 Asymmetry[113] presence ≥ 1	0.0 (1)	0.0 (1)	–	50.0 (2)	100 (2)	46.2 (13)	–	20.0 (5)	93.9 (33)	20.0 (10)	12.5 (136)
M_1 Cusp 6[102] presence ≥ 1	0.0 (1)	0.0 (1)	0.0 (1)	100 (1)	100 (2)	28.6 (7)	0.0 (2)	33.3 (6)	38.0 (21)	0.0 (14)	18.8 (229)
M_1 Cusp 7[102] presence ≥ 2	0.0 (2)	100 (1)	0.0 (1)	33.3 (3)	100 (2)	54.5 (11)	33.3 (3)	0.0 (6)	23.5 (34)	42.9 (21)	12.7 (283)
M_1 Protostylid[102] presence ≥ 2	100 (1)	0.0 (1)	0.0 (1)	33.3 (3)	50.0 (2)	50.0 (8)	0.0 (3)	0.0 (9)	0.0 (38)	12.5 (16)	2.4 (252)
M_2 Middle Trigonid Crest[114-116] presence ≥ 2	100 (1)	0.0 (2)	0.0 (1)	0.0 (4)	0.0 (2)	16.7 (18)	0.0 (3)	33.3 (6)	96.2 (26)	0.0 (16)	1.3 (226)
M_2 Deflecting Wrinkle[102] presence ≥ 2	0.0 (1)	–	–	0.0 (1)	0.0 (1)	30.0 (10)	0.0 (3)	25.0 (4)	0.0 (20)	0.0 (3)	1.0 (203)
M_2 groove pattern[102] presence = Y	0.0 (1)	100 (2)	0.0 (1)	75.0 (4)	50.0 (2)	91.7 (12)	100 (3)	57.1 (7)	78.8 (33)	69.8 (16)	29.0 (272)

M_2 Cusp Number[102] presence = 4	0.0 (2)	0.0 (2)	0.0 (1)	0.0 (4)	0.0 (2)	0.0 (13)	0.0 (3)	0.0 (7)	0.0 (37)	15.0 (20)	47.0 (234)
M_2 cusp 6[102] presence ≥ 1	–	50.0 (2)	100 (1)	66.7 (3)	100 (2)	50.0 (12)	0.0 (2)	0.0 (6)	57.9 (19)	9.1 (11)	12.7 (212)
M_2 Cusp 7[102] presence ≥ 2	0.0 (1)	0.0 (2)	100 (1)	25.0 (4)	0.0 (1)	29.4 (17)	50.0 (2)	0.0 (8)	19.2 (26)	5.6 (18)	4.4 (248)
M_2 Protostylid[102] presence ≥ 2	100 (1)	50.0 (2)	0.0 (1)	50.0 (2)	0.0 (2)	13.3 (15)	0.0 (1)	0.0 (8)	3.0 (33)	14.3 (14)	4.1 (195)
M_3 groove pattern[102] presence = Y	100 (2)	0.0 (2)	0.0 (1)	50.0 (4)	50.0 (2)	55.6 (9)	50.0 (2)	33.3 (3)	37.5 (16)	44.4 (9)	23.7 (177)
M_3 Cusp Number[102] presence = 4	0.0 (2)	0.0 (2)	0.0 (1)	0.0 (4)	0.0 (2)	22.2 (9)	0.0 (2)	0.0 (2)	0.0 (25)	16.7 (12)	35.8 (162)
M_3 Middle Trigonid Crest[114-116] presence ≥ 2	0.0 (2)	100 (1)	–	0.0 (2)	0.0 (1)	12.5 (8)	50.0 (2)	33.3 (3)	93.8 (16)	0.0 (10)	0.0 (162)
M_3 Deflecting Wrinkle[102] presence ≥ 2	50.0 (2)	–	–	0.0 (1)	0.0 (1)	50.0 (6)	0.0 (2)	0.0 (3)	6.7 (15)	11.1 (9)	6.4 (140)
M_3 Cusp 6[102] presence ≥ 1	100 (2)	50.0 (2)	100 (1)	100 (2)	0.0 (1)	57.0 (7)	0.0 (1)	50.0 (2)	50.0 (10)	36.4 (11)	30.2 (162)
M_3 Cusp 7[102] presence ≥ 2	50.0 (2)	50.0 (2)	0.0 (1)	50.0 (2)	0.0 (1)	14.3 (7)	100 (1)	0.0 (2)	35.3 (17)	8.3 (12)	7.5 (160)
M_3 Protostylid[102] presence ≥ 2	100 (2)	50.0 (2)	0.0 (1)	0.0 (2)	0.0 (1)	50.0 (8)	0.0 (1)	33.3 (3)	17.4 (23)	25.0 (12)	17.4 (132)

Supplementary Table 21: Dental Metrics

Shara E. Bailey

Measurements in mm. Parentheses indicate sample size. Brackets indicate range.

Site	UI1	P3	P4
Maxilla			
THI-GH			
BL	6.2	11.7	11.8
MD	7.5	8.4	8.4
Tighennif			
BL	–	–	–
MD	–	–	–
Sidi Abderrahmane			
BL	–	–	–
MD	–	–	–
African Middle Pleistocene			
BL	–	–	11.3 (1)
MD	–	8.7 (1)	7.9 (1)
Jebel Irhoud			
BL	–	11.6 (1)	11.3 (1)
MD	–	8.4 (1)	8.3 (1)
H. erectus			
BL	8.1 (10) [7.4-9.4]	11.9 (14) [10.4-13.4]	11.3 (22) [9.9-12.7]
MD	10.6 (10) [8.4-12.0]	8.4 (16) [7.4-9.3]	8.1 (21) [7.2-9.2]
H. antecessor			
BL	–	11.7 (2) [11.6,11.8]	11.6 (2) [11.6,11.7]
MD	–	8.6 (2) [8.4,8.8]	8.1 (1)
European Middle Pleistocene			
BL	7.8 (13) [7.5-8.2]	10.9 (7) [10.5-12.1]	11.1 (3) [9.9-12.2]
MD	9.6 (13) [9.2-9.9]	8.8 (4) [8.0-10.7]	8.0 (3) [7.3-8.4]
H. neanderthalensis			
BL	8.5 (18) [7.3-9.5)	10.7 (30) [9.1-11.9]	10.5 (25) [8.2-11.7]
MD	9.4 (18) [8.0-11.0]	8.0 (29) [6.2-9.3]	7.6 (25) [5.7-8.8]
Early *H. sapiens*			
BL	7.9 (10) [6.6-8.9]	10.4 (10) [10.0-11.6]	10.7 (11) [9.4-11.5]
MD	9.9 (13) [8.5-11.3]	7.9 (10) [7.0-8.7]	7.7 (12) [6.5-9.3]
Upper Paleolithic and recent *H. sapiens*			
BL	7.2 (124) [5.9-8.6]	9.4 (213) [7.9-11.2]	9.5 (208) [7.6-12.3]
MD	8.7 (117) [6.7-10.6	7.1 (199) [5.6-8.6]	6.9 (190) [5.4-11.3]

Mandible								
	dm1	dm2	C	P3	P4	M1	M2	M3
ThI-GH								
BL	7.6 (1)	9.2 (1)	9.5 (1)	7.75 (1) [7.6,7.9]	9.8 (2) [10.7,8.9]	11.4 (2) [10.4,12.5]	11.8 (2) [10.6, 13.0]	10.8 (2) [9.9, 11.6]
MD	9.0 (1)	11.4 (1)	7.0 (1)	9.0 (1) [8.9,9.1]	8.35 (2) [8.9,7.8]	12.9 (2) [12.3, 13.5]	13.7 (2) [12.3, 15.0]	12.5 (2) [11.7, 13.2]
Tighennif								
BL	–	–	–	10.6 (2) [10.1,11.1]	10.8 (2) [10.2,11.3]	12.5 (2) [12.2,12.7]	13.3 (2) [13.2,13.4]	12.2 (2) [12.1,12.3]
MD	–	–	–	8.6 (2) [8.4,8.7]	8.6 (2) [8.1,9.1]	13.6 (2) [13.0-14.1]	13.4 (2) [12.8,14.0]	12.5 (2) [11.9,13.0]
Sidi Abderrahmane								
BL	–	–	–	–	10.6 (1)	12.5 (1)	12.9 (1)	11.6 (1)
MD	–	–	–	–	9.0 (1)	13.8 (1)		12.9 (1)
Middle Pleistocene Africa								
BL	–	–	8.8 (2) [7.7,10.0]	10.1 (4) [9.4-11.1]	10.2 (7) [8.7-11.3]	11.8 (9) [10.5-12.7]	11.8 (9) [10.3-13.4]	11.6 (7) [10.6-12.3]
MD	–	–	7.8 (2) [7.2,8.2]	8.9 (4) [8.4-10.0]	8.9 (8) [7.5-10.3]	12.9 (9) [11.9-14.1]	12.8 (9) [11.0-14.0]	13.0 (6) [11.9-15.2]
Jebel Irhoud								
BL	–	10.1 (1)	9.25 (2) [9.1, 9.4]	9.6 (1)	10.5 (2) [10.5,10.5]	12.2 (2) [12.2,12.2]	12.2 (1)	11.0 (1)
MD	–	11.5 (1)	8.85 (2) [8.6,9.1]	9.5 (2) [9.2-9.7]	9.2 (2) [8.9,9.5]	14.6 (2)	13.8 (2)	12.9 (1)
H. erectus s.l. [117,118]								
BL	6.8 (3) [6.6-7.0]	9.5 (9) [8.4-10.5]	8.8 (9) [8.3-9.6]	10.3 (9) [8.9-11.5]	10.6 (10) [9.7-11.7]	12.2 (15) [10.7-13.5]	13.1 (14) [11.7-14.3]	12.4 (8)
MD	8.5 (3) [7.0-9.8]	11.3 (9) [10.4-12.5]	8.4 (4) [8.0-8.9]	8.9 (9) [7.9-9.9]	8.7 (7) [7.2-9.9]	13.3 (13) [12.1-14.9]	13.3 (12) [12.5-14.4]	13.4 (8)
H. antecessor[119,120]								
BL	8.55 (2)	9.2 (1)	10.0 (1)	10.2 (2) [9.7,10.6]	10.0 (3) [9.4-10.5)	11.5 (2) [11.2,11.8]	11.7 (3) [11.6-12.0]	9.6 (2) [8.8,10.4]
MD	8.95 (2)	11.4 (1)	8.1 (1)	8.4 (2) [8.0,8.8]	8.0 (3) [7.6-83)	12.1 (2) [12.0,12.2]	13.4 (3) [13.0-13.8]	10.7 (2) [9.2,12.1]
Middle Pleistocene Europe[121,122]								

BL	–	–	8.4 (11) [5.9-9.8]	8.9 (9) [7.6-10.0]	9.9 (5) [8.7-11.1)	10.6 (15) [9.7-11.5]	10.4 (17) [8.6-12.4]	10.0 (10) [8.7-11.3]
MD	–	–	7.6 (11) [6.7-83.7]	7.8 (8) [7.3-8.4]	8.9 (6) [7.5-10.3]	11.2 (16) [10.6-12.0]	11.5 (17) [9.7-14.8]	11.2 (10) [9.4-12.7]
Neanderthals								
BL	7.5 (17) [6.2-8.7]	9.5 (26) [8.4-10.5]	9.2 (24) [7.8-10.3]	9.1 (31) [7.2-10.3]	9.3 (28) [7.6-11.1]	11.1 (36) [9.7-12.9]	11.1 (32) [9.6-12.4]	10.8 (29) [7.8-13.1]
MD	9.0 (17) [8.0-9.9]	10.6 (27) [9.2-11.5]	8.0 (22) [6.9-9.0]	8.0 (31) [6.3-9.9]	7.9 (23) [5.7-11.8]	11.8 (33) [10.1-13.6]	12.0 (29) [10.5-14.0]	11.8 (26) [9.4-13.9]
Early *H. sapiens*								
BL	7.4 (10) [6.6-8.4]	9.8 (7) [8.7-10.8]	8.7 (14) [7.0-10.2]	9.3 (12) [8.0-12.2]	9.5 (18) [7.8-10.9]	11.8 (21) [10.5-14.3]	11.1 (24) [9.2-12.7]	11.0 (15) [9.2-12.8]
MD	9.0 (7) [7.9-10.0]	11.0 (4) [10.1-11.6]	8.2 (12) [6.4-10.0]	8.4 (11) [7.2-11.0]	8.0 (14) [7.0-9.6]	12.7 (22) [10.8-14.7]	11.9 (17) [10.2-14.5]	11.9 (15) [10.1-13.8]
Recent (Upper Paleolithic + extant)								
BL	7.1 (16) [6.2-8.0]	9.1 (20) [7.8-10.0]	7.7 (166) [6.0-9.7]	8.0 (203) [6.4-10.2]	8.4 (190) [6.8-10.8]	10.7 (296) [6.7-12.6]	10.5 (243) [8.6-12.5]	10.4 (137)) [8.6-14.2]
MD	8.3 (16) [6.7-9.4]	10.3 (19) [8.5-11.1]	6.9 (162) [5.4-8.1	7.1 (189) [5.8-7.1]	7.2 (176) [5.5-10.4]	11.4 (273) [9.2-13.5]	10.9 (216) [8.9-14.3]	10.8 (123) [8.2-12.6]

Supplementary Table 22: Tooth Comparative Samples.

Shara E. Bailey, Stefano Benazzi, Adeline Le Cabec, Thomas W. Davies, Jean-Jacques Hublin, Mykolas D. Imbrasas, Kornelius Kupczik, Alejandra Ortiz, Matthew M. Skinner, Rita Sorrentino,

Name	Tooth	Species/Group	Site/Collection	Size	EDJ all	EDJ ridge	Roots	Crown outline
KNM-ER 1507	LdM2	*Homo erectus*	Koobi Fora, Kenya					*
KNM-ER 1808G	LM2	*Homo erectus*	Koobi Fora, Kenya	*				
KNM-ER 1812C	LM3	*Homo erectus*	Koobi Fora, Kenya	*	*			
KNM-ER 3733	UP4	*Homo erectus*	Koobi Fora, Kenya		*			
KNM-ER 730	LM1	*Homo erectus*	Koobi Fora, Kenya				*	
KNM-ER 730	LM2	*Homo erectus*	Koobi Fora, Kenya				*	
KNM-ER 730	LM3	*Homo erectus*	Koobi Fora, Kenya				*	
KNM-ER 806	LM1	*Homo erectus*	Koobi Fora, Kenya	*		*		
KNM-ER 806	LM2	*Homo erectus*	Koobi Fora, Kenya	*				
KNM-ER 806	LM3	*Homo erectus*	Koobi Fora, Kenya	*	*			
KNM-ER 806	LP3	*Homo erectus*	Koobi Fora, Kenya	*				
KNM-ER 820	LM2	*Homo erectus*	Koobi Fora, Kenya	*				
KNM-ER 820	LdM2	*Homo erectus*	Koobi Fora, Kenya					*
KNM-ER 820	LM1	*Homo erectus*	Koobi Fora, Kenya	*				
KNM-ER 820	LP3	*Homo erectus*	Koobi Fora, Kenya	*				
KNM-ER 820	LP4	*Homo erectus*	Koobi Fora, Kenya	*				
KNM-ER 992	LM1	*Homo erectus*	Koobi Fora, Kenya	*		*	*	
KNM-ER 992	LM2	*Homo erectus*	Koobi Fora, Kenya	*			*	
KNM-ER 992	LM3	*Homo erectus*	Koobi Fora, Kenya	*	*			
KNM-ER 992	LP3	*Homo erectus*	Koobi Fora, Kenya	*				
KNM-ER 992	LP4	*Homo erectus*	Koobi Fora, Kenya	*	*			
KNM-WT 15000	LM1	*Homo erectus*	Nariokotome, Kenya	*			*	
KNM-WT 15000	LM2	*Homo erectus*	Nariokotome, Kenya	*				
KNM-WT 15000	LP3	*Homo erectus*	Nariokotome, Kenya	*				
KNM-WT 15000	LP4	*Homo erectus*	Nariokotome, Kenya	*				
KNM-WT 15000	UI1	*Homo erectus*	Nariokotome, Kenya				*	
KNM-WT 15000	UI2	*Homo erectus*	Nariokotome, Kenya				*	
KNM-WT 15000	LI2	*Homo erectus*	Nariokotome, Kenya				*	
OH 23	LM1	*Homo erectus*	Olduvai Gorge, Tanzania	*				
OH 23	LM2	*Homo erectus*	Olduvai Gorge, Tanzania	*				
OH 23	LP4	*Homo erectus*	Olduvai Gorge, Tanzania	*				
S7-48	UI1	*Homo erectus*	Sangiran, Indonesia				*	
S7-50	LI2	*Homo erectus*	Sangiran, Indonesia				*	
Sangiran 1b	LM1	*Homo erectus*	Sangiran, Indonesia	*		*		
Sangiran 1b	LM2	*Homo erectus*	Sangiran, Indonesia	*				
Sangiran 1b	LM3	*Homo erectus*	Sangiran, Indonesia	*	*			
Sangiran 1b	LP4	*Homo erectus*	Sangiran, Indonesia	*	*			
Sangiran PCG2	LdM2	*Homo erectus*	Sangiran, Indonesia					*
Sangiran S4	UP4	*Homo erectus*	Sangiran, Indonesia		*			
SKX 21204	LP4	*Homo erectus*	Swarktrans, South Africa		*			
Tighennif 1	LM1	Early Pleist. *Homo*	Tighennif, Algeria	*				
Tighennif 1	LM2	Early Pleist. *Homo*	Tighennif, Algeria	*				
Tighennif 1	LM3	Early Pleist. *Homo*	Tighennif, Algeria	*	*			
Tighennif 1	LP3	Early Pleist. *Homo*	Tighennif, Algeria	*				
Tighennif 1	LP4	Early Pleist. *Homo*	Tighennif, Algeria	*				
Tighennif 1954-7-04	LI1	Early Pleist. *Homo*	Tighennif, Algeria				*	
Tighennif 1954-7-05	LC	Early Pleist. *Homo*	Tighennif, Algeria				*	
Tighennif 1954-7-08	LI2	Early Pleist. *Homo*	Tighennif, Algeria				*	
Tighennif 2	LM1	Early Pleist. *Homo*	Tighennif, Algeria	*		*		

Tighennif 2	LM2	Early Pleist. *Homo*	Tighennif, Algeria	*				
Tighennif 2	LM3	Early Pleist. *Homo*	Tighennif, Algeria	*				
Tighennif 2	LP3	Early Pleist. *Homo*	Tighennif, Algeria	*				
Tighennif 2	LP4	Early Pleist. *Homo*	Tighennif, Algeria	*	*			
ATD6-112	LdM1	*H. antecessor*	Gran Dolina, Spain					*
ATD6-112	LdM2	*H. antecessor*	Gran Dolina, Spain	*				*
ATD6-112	LM1	*H. antecessor*	Gran Dolina, Spain	*		*		
ATD6-113	LM2	*H. antecessor*	Gran Dolina, Spain	*				
ATD6-113	LM3	*H. antecessor*	Gran Dolina, Spain	*	*			
ATD6-3	LP3	*H. antecessor*	Gran Dolina, Spain	*				
ATD6-4	LP4	*H. antecessor*	Gran Dolina, Spain	*	*			
ATD6-5	LM2	*H. antecessor*	Gran Dolina, Spain	*				
ATD6-5b	LM3	*H. antecessor*	Gran Dolina, Spain	*	*			
ATD6-9	UP4	*H. antecessor*	Gran Dolina, Spain		*			
ATD6-93	LM1	*H. antecessor*	Gran Dolina, Spain					*
ATD6-94	LM1	*H. antecessor*	Gran Dolina, Spain	*		*		
BH-1	LM1	Middle Pleist. *Homo*	Balanica, Serbia	*		*	*	
BH-1	LM2	Middle Pleist. *Homo*	Balanica, Serbia	*			*	
BH-1	LM3	Middle Pleist. *Homo*	Balanica, Serbia	*	*			
Mauer	LI2	Middle Pleist. *Homo*	Mauer, Germany				*	
Mauer	LC	Middle Pleist. *Homo*	Mauer, Germany				*	
Mauer	LM1	Middle Pleist. *Homo*	Mauer, Germany	*		*	*	
Mauer	LM2	Middle Pleist. *Homo*	Mauer, Germany	*			*	
Mauer	LM3	Middle Pleist. *Homo*	Mauer, Germany	*	*		*	
Mauer	LP3	Middle Pleist. *Homo*	Mauer, Germany	*				
Mauer	LP4	Middle Pleist. *Homo*	Mauer, Germany	*	*			
Steinheim	UP4	Middle Pleist. *Homo*	Steinheim, Germany		*			
OH 22	LM1	Middle Pleist. *Homo*	Olduvai Gorge, Tanzania	*		*		
OH 22	LM2	Middle Pleist. *Homo*	Olduvai Gorge, Tanzania	*				
OH 22	LP3	Middle Pleist. *Homo*	Olduvai Gorge, Tanzania	*				
OH 22	LP4	Middle Pleist. *Homo*	Olduvai Gorge, Tanzania	*	*			
U.W. 101-1400	LdM2	Middle Pleist. *Homo*	Rising Star, South Africa					*
U.W. 101-1686	LdM2	Middle Pleist. *Homo*	Rising Star, South Africa					*
Sale	UP4	Middle Pleist. *Homo*	Sale, Morocco		*			
Sidi Abderrahmane	LM1	Middle Pleist. *Homo*	Sidi Abderrahmane, Morocco	*		*		
Sidi Abderrahmane	LM2	Middle Pleist. *Homo*	Sidi Abderrahmane, Morocco	*				
Sidi Abderrahmane	LM3	Middle Pleist. *Homo*	Sidi Abderrahmane, Morocco	*	*			
Amud 1	UI1	*H. neanderthalensis*	Amud, Israel				*	
Amud 1	UI2	*H. neanderthalensis*	Amud, Israel				*	
Amud 1	LI2	*H. neanderthalensis*	Amud, Israel				*	
Amud 1	LC	*H. neanderthalensis*	Amud, Israel				*	
Amud 1	LM2	*H. neanderthalensis*	Amud, Israel				*	
Amud 1	LM1	*H. neanderthalensis*	Amud, Israel				*	
Amud 1	LM3	*H. neanderthalensis*	Amud, Israel				*	
Archi 1	LdM1	*H. neanderthalensis*	Archi, Italy					*
Archi 1	LdM2	*H. neanderthalensis*	Archi, Italy					*
Arcy 25	LdM1	*H. neanderthalensis*	Arcy-sur-Cure, France					*
Arcy 29	LdM2	*H. neanderthalensis*	Arcy-sur-Cure, France					*
Barakai	LdM1	*H. neanderthalensis*	Barakai, Russia					*
Barakai	LdM2	*H. neanderthalensis*	Barakai, Russia					*
Bruniquel II	LdM1	*H. neanderthalensis*	Bruniquel, France					*
Cavallo A	LdM2	*H. neanderthalensis*	Cavallo Cave, Italy					*
Chagyrskaya 6	LC	*H. neanderthalensis*	Chagysrkaya, Russia				*	
Combe-Grenal 27	UI1	*H. neanderthalensis*	Combe-Grenal, France				*	
Combe-Grenal 29	LP4	*H. neanderthalensis*	Combe-Grenal, France	*	*			
Combe-Grenal I	LdM1	*H. neanderthalensis*	Combe-Grenal, France					*
Combe-Grenal I	LdM2	*H. neanderthalensis*	Combe-Grenal, France					*
Combe-Grenal I	LM1	*H. neanderthalensis*	Combe-Grenal, France	*		*		
Combe-Grenal I	LP3	*H. neanderthalensis*	Combe-Grenal, France	*				
Combe-Grenal I	LP4	*H. neanderthalensis*	Combe-Grenal, France	*				
Combe-Grenal IV	LM1	*H. neanderthalensis*	Combe-Grenal, France	*		*	*	
Combe-Grenal IV	LP4	*H. neanderthalensis*	Combe-Grenal, France	*	*			
Combe-Grenal V	UI1	*H. neanderthalensis*	Combe-Grenal, France				*	
Combe-Grenal VIII	LP4	*H. neanderthalensis*	Combe-Grenal, France	*	*			

Combe-Grenal X	UI2	*H. neanderthalensis*	Combe-Grenal, France				*	
Combe-Grenal XI	UI1	*H. neanderthalensis*	Combe-Grenal, France				*	
Combe-Grenal XII	LM3	*H. neanderthalensis*	Combe-Grenal, France	*	*		*	
Combe-Grenal XV	LP3	*H. neanderthalensis*	Combe-Grenal, France	*				
Ehringsdorf 1	LM1	*H. neanderthalensis*	Ehringsdorf, Germany				*	
Ehringsdorf F	LI2	*H. neanderthalensis*	Ehringsdorf, Germany				*	
Ehringsdorf F	LC	*H. neanderthalensis*	Ehringsdorf, Germany				*	
Ehringsdorf G 1049	LP4	*H. neanderthalensis*	Ehringsdorf, Germany		*			
Ehringsdorf G1	LI2	*H. neanderthalensis*	Ehringsdorf, Germany				*	
Ehringsdorf G1	LC	*H. neanderthalensis*	Ehringsdorf, Germany				*	
Ehringsdorf G1010_69	LM2	*H. neanderthalensis*	Ehringsdorf, Germany				*	
Ehringsdorf G1048_69	LM1	*H. neanderthalensis*	Ehringsdorf, Germany	*		*		
Ehringsdorf G3	UI2	*H. neanderthalensis*	Ehringsdorf, Germany				*	
SD 1106	UP4	*H. neanderthalensis*	El Sidrón, Spain		*			
SD 1135	LM3	*H. neanderthalensis*	El Sidrón, Spain	*	*			
SD 331ab	UI1	*H. neanderthalensis*	El Sidrón, Spain				*	
SD 411	UP4	*H. neanderthalensis*	El Sidrón, Spain		*			
SD 50	UP4	*H. neanderthalensis*	El Sidrón, Spain		*			
SD 540	LM2	*H. neanderthalensis*	El Sidrón, Spain	*				
SD 657	LM2	*H. neanderthalensis*	El Sidrón, Spain				*	
SD 755	LM2	*H. neanderthalensis*	El Sidrón, Spain	*				
SD 756	LM1	*H. neanderthalensis*	El Sidrón, Spain	*		*		
SD 780	LM1	*H. neanderthalensis*	El Sidrón, Spain	*		*		
Engis 2	LdM1	*H. neanderthalensis*	Engis, Belgium					*
Engis 2	LdM2	*H. neanderthalensis*	Engis, Belgium	*				*
Kebara 1	LdM1	*H. neanderthalensis*	Kebara, Israel	*				*
Kebara 1	LdM2	*H. neanderthalensis*	Kebara, Israel	*				*
Kebara 2	LI2	*H. neanderthalensis*	Kebara, Israel				*	
Kebara 2	LC	*H. neanderthalensis*	Kebara, Israel				*	
Kebara 2	LM1	*H. neanderthalensis*	Kebara, Israel	*			*	
Kebara 2	LM2	*H. neanderthalensis*	Kebara, Israel	*			*	
Kebara 2	LM3	*H. neanderthalensis*	Kebara, Israel	*				
Kebara 2	LP3	*H. neanderthalensis*	Kebara, Israel	*				
Kebara 2	LP4	*H. neanderthalensis*	Kebara, Israel	*				
KMH 27	UI2	*H. neanderthalensis*	Kebara, Israel				*	
KMH 28	LI2	*H. neanderthalensis*	Kebara, Israel				*	
KMH 31	LC	*H. neanderthalensis*	Kebara, Israel				*	
KMH 4	LdM1	*H. neanderthalensis*	Kebara, Israel	*				
KMH 4	LdM2	*H. neanderthalensis*	Kebara, Israel	*				
KDP D126	UI1	*H. neanderthalensis*	Krapina, Croatia				*	
KRP 46 (KDP 2)	UP4	*H. neanderthalensis*	Krapina, Croatia		*			
KRP 47 (KDP 3)	UP4	*H. neanderthalensis*	Krapina, Croatia		*			
KRP 48 (KDP 4)	UP4	*H. neanderthalensis*	Krapina, Croatia		*			
KRP 49 (KDP 5)	UP4	*H. neanderthalensis*	Krapina, Croatia		*			
KRP 49 (KDP 5)	UI1	*H. neanderthalensis*	Krapina, Croatia				*	
KRP 49 (KDP 5)	UI2	*H. neanderthalensis*	Krapina, Croatia				*	
KRP 50	UI1	*H. neanderthalensis*	Krapina, Croatia				*	
KRP 50	UI2	*H. neanderthalensis*	Krapina, Croatia				*	
KRP 52 (KDP 8)	LM1	*H. neanderthalensis*	Krapina, Croatia	*		*		
KRP 52 (KDP 8)	LP3	*H. neanderthalensis*	Krapina, Croatia	*				
KRP 52 (KDP 8)	LP4	*H. neanderthalensis*	Krapina, Croatia	*	*			
KRP 53 (KDP 9)	LdM2	*H. neanderthalensis*	Krapina, Croatia					*
KRP 53 (KDP 9)	LI2	*H. neanderthalensis*	Krapina, Croatia				*	
KRP 53 (KDP 9)	LM1	*H. neanderthalensis*	Krapina, Croatia	*		*		
KRP 53 (KDP 9)	LM2	*H. neanderthalensis*	Krapina, Croatia	*				
KRP 53 (KDP 9)	LM3	*H. neanderthalensis*	Krapina, Croatia	*				
KRP 53 (KDP 9)	LP4	*H. neanderthalensis*	Krapina, Croatia	*				
KRP 54 (KDP 4)	LM1	*H. neanderthalensis*	Krapina, Croatia	*		*	*	
KRP 54 (KDP 4)	LM2	*H. neanderthalensis*	Krapina, Croatia	*			*	
KRP 54 (KDP 4)	LP3	*H. neanderthalensis*	Krapina, Croatia	*				
KRP 54 (KDP 4)	LP4	*H. neanderthalensis*	Krapina, Croatia	*	*			
KRP 54 (KDP 4)	LI2	*H. neanderthalensis*	Krapina, Croatia				*	
KRP 54 (KDP 4)	LC	*H. neanderthalensis*	Krapina, Croatia				*	
KRP 55 (KDP 10)	LI2	*H. neanderthalensis*	Krapina, Croatia				*	

KRP 55 (KDP 10)	LC	*H. neanderthalensis*	Krapina, Croatia				*	
KRP 55 (KDP 10)	LM1	*H. neanderthalensis*	Krapina, Croatia	*		*	*	
KRP 55 (KDP 10)	LM2	*H. neanderthalensis*	Krapina, Croatia	*			*	
KRP 55 (KDP 10)	LP3	*H. neanderthalensis*	Krapina, Croatia	*				
KRP 55 (KDP 10)	LP4	*H. neanderthalensis*	Krapina, Croatia	*				
KRP 57 (KDP 12)	LM1	*H. neanderthalensis*	Krapina, Croatia	*			*	
KRP 57 (KDP 12)	LM2	*H. neanderthalensis*	Krapina, Croatia	*			*	
KRP 57 (KDP 12)	LM3	*H. neanderthalensis*	Krapina, Croatia	*	*		*	
KRP 58 (KDP 6)	LI2	*H. neanderthalensis*	Krapina, Croatia				*	
KRP 58 (KDP 6)	LC	*H. neanderthalensis*	Krapina, Croatia				*	
KRP 58 (KDP 6)	LM1	*H. neanderthalensis*	Krapina, Croatia	*			*	
KRP 58 (KDP 6)	LM2	*H. neanderthalensis*	Krapina, Croatia	*				
KRP 58 (KDP 6)	LM3	*H. neanderthalensis*	Krapina, Croatia	*				
KRP 58 (KDP 6)	LP3	*H. neanderthalensis*	Krapina, Croatia	*				
KRP 58 (KDP 6)	LP4	*H. neanderthalensis*	Krapina, Croatia	*	*			
KRP 59 (KDP 13)	LI2	*H. neanderthalensis*	Krapina, Croatia				*	
KRP 59 (KDP 13)	LC	*H. neanderthalensis*	Krapina, Croatia				*	
KRP 59 (KDP 13)	LM1	*H. neanderthalensis*	Krapina, Croatia	*			*	
KRP 59 (KDP 13)	LM2	*H. neanderthalensis*	Krapina, Croatia	*				
KRP 59 (KDP 13)	LM3	*H. neanderthalensis*	Krapina, Croatia	*				
KRP 59 (KDP 13)	LP4	*H. neanderthalensis*	Krapina, Croatia	*				
KRP D1 (KDP 23)	LM2	*H. neanderthalensis*	Krapina, Croatia	*				
KRP D104	LM3	*H. neanderthalensis*	Krapina, Croatia	*	*			
KRP D105	LM2	*H. neanderthalensis*	Krapina, Croatia	*				
KRP D106 (KDP 10)	LM3	*H. neanderthalensis*	Krapina, Croatia	*	*			
KRP D107 (KDP 3)	LM2	*H. neanderthalensis*	Krapina, Croatia	*				
KRP D111 (KDP 9)	LP3	*H. neanderthalensis*	Krapina, Croatia	*				
KRP D114 (KDP 31)	LP3	*H. neanderthalensis*	Krapina, Croatia	*				
KRP D115 (KDP 31)	UP4	*H. neanderthalensis*	Krapina, Croatia		*			
KRP D122	UI2	*H. neanderthalensis*	Krapina, Croatia				*	
KRP D123	UI1	*H. neanderthalensis*	Krapina, Croatia				*	
KRP D125	UI2	*H. neanderthalensis*	Krapina, Croatia				*	
KRP D127	UI2	*H. neanderthalensis*	Krapina, Croatia				*	
KRP D156	UI2	*H. neanderthalensis*	Krapina, Croatia				*	
KRP D157	UI1	*H. neanderthalensis*	Krapina, Croatia				*	
KRP D158	UI1	*H. neanderthalensis*	Krapina, Croatia				*	
KRP D159	UI2	*H. neanderthalensis*	Krapina, Croatia				*	
KRP D160	UI2	*H. neanderthalensis*	Krapina, Croatia				*	
KRP D27 (KDP 23)	LP3	*H. neanderthalensis*	Krapina, Croatia	*				
KRP D28 (KDP 18)	LP3	*H. neanderthalensis*	Krapina, Croatia	*				
KRP D29 (KDP 34)	LP3	*H. neanderthalensis*	Krapina, Croatia	*				
KRP D33 (KDP 27)	LP3	*H. neanderthalensis*	Krapina, Croatia	*				
KRP D34 (KDP 11)	LP3	*H. neanderthalensis*	Krapina, Croatia	*				
KRP D35 (KDP 34)	LP4	*H. neanderthalensis*	Krapina, Croatia	*	*			
KRP D41 (KDP 23)	UP4	*H. neanderthalensis*	Krapina, Croatia		*			
KRP D42	UP4	*H. neanderthalensis*	Krapina, Croatia		*			
KRP D44	UP4	*H. neanderthalensis*	Krapina, Croatia		*			
KRP D50 (KDP 27)	LP4	*H. neanderthalensis*	Krapina, Croatia	*	*			
KRP D51	LP3	*H. neanderthalensis*	Krapina, Croatia	*				
KRP D6 (KDP 27)	LM2	*H. neanderthalensis*	Krapina, Croatia	*				
KRP D62 (KDP 8)	LdM2	*H. neanderthalensis*	Krapina, Croatia	*				*
KRP D63 (KDP 28)	LdM2	*H. neanderthalensis*	Krapina, Croatia	*				*
KRP D64	LdM2	*H. neanderthalensis*	Krapina, Croatia					*
KRP D65	LdM2	*H. neanderthalensis*	Krapina, Croatia	*				*
KRP D66	LdM2	*H. neanderthalensis*	Krapina, Croatia					*
KRP D68 (KDP 22)	LdM2	*H. neanderthalensis*	Krapina, Croatia	*				*
KRP D79 (KDP 23)	LM1	*H. neanderthalensis*	Krapina, Croatia			*		
KRP D80	LM2	*H. neanderthalensis*	Krapina, Croatia	*			*	
KRP D81 (KDP 27)	LM1	*H. neanderthalensis*	Krapina, Croatia	*		*		
KRP D82 (KDP 20)	LM1	*H. neanderthalensis*	Krapina, Croatia				*	
KRP D85 (KDP 23)	LM3	*H. neanderthalensis*	Krapina, Croatia				*	
KRP D86	LM2	*H. neanderthalensis*	Krapina, Croatia	*				
KRP D9	LM2	*H. neanderthalensis*	Krapina, Croatia	*				
KRP_MdB_O_C	LdM2	*H. neanderthalensis*	Krapina, Croatia					*

BD 1	LI2	*H. neanderthalensis*	La Chaise, France				*	
BD 1	LC	*H. neanderthalensis*	La Chaise, France				*	
BD 1	LM1	*H. neanderthalensis*	La Chaise, France				*	
BD 1	LM2	*H. neanderthalensis*	La Chaise, France				*	
BD 1	LM3	*H. neanderthalensis*	La Chaise, France				*	
BD 10	UI2	*H. neanderthalensis*	La Chaise, France				*	
BD 12	UI1	*H. neanderthalensis*	La Chaise, France				*	
BD 13	LC	*H. neanderthalensis*	La Chaise, France				*	
BD J4 C9	LM1	*H. neanderthalensis*	La Chaise, France				*	
Abri Suard 14-7	LM1	*H. neanderthalensis*	La Chaise, France	*		*		
Abri Suard 49	LM1	*H. neanderthalensis*	La Chaise, France	*		*		
Abri Suard 5	LM1	*H. neanderthalensis*	La Chaise, France	*		*		
Abri Suard S14-1a	LdM1	*H. neanderthalensis*	La Chaise, France					*
Abri Suard S18	LdM1	*H. neanderthalensis*	La Chaise, France					*
Abri Suard S18	LdM2	*H. neanderthalensis*	La Chaise, France					*
Abri Suard S33	UI2	*H. neanderthalensis*	La Chaise, France				*	
Abri Suard S36	LM2	*H. neanderthalensis*	La Chaise, France	*			*	
Abri Suard S36	LM3	*H. neanderthalensis*	La Chaise, France	*	*		*	
Abri Suard S37	LdM1	*H. neanderthalensis*	La Chaise, France	*				
Abri Suard S37	LdM2	*H. neanderthalensis*	La Chaise, France	*				
Abri Suard S43	LM3	*H. neanderthalensis*	La Chaise, France	*	*			
La Fate 2	LdM2	*H. neanderthalensis*	La Fate, France					*
La Ferrassie 8	LdM1	*H. neanderthalensis*	La Ferrassie, France					*
La Quina H9	LM1	*H. neanderthalensis*	La Quina, France	*				
La Quina H9	LM2	*H. neanderthalensis*	La Quina, France	*				
La Quina H9	LM3	*H. neanderthalensis*	La Quina, France	*	*			
LKH 1	LM3	*H. neanderthalensis*	Lakonis, Greece	*	*			
Le Moustier 1	UI1	*H. neanderthalensis*	Le Moustier, France				*	
Le Moustier 1	UI2	*H. neanderthalensis*	Le Moustier, France				*	
Le Moustier 1	LI2	*H. neanderthalensis*	Le Moustier, France				*	
Le Moustier 1	LC	*H. neanderthalensis*	Le Moustier, France				*	
Le Moustier 1	LM1	*H. neanderthalensis*	Le Moustier, France	*		*	*	
Le Moustier 1	LM2	*H. neanderthalensis*	Le Moustier, France	*			*	
Le Moustier 1	LM3	*H. neanderthalensis*	Le Moustier, France	*	*			
Le Moustier 1	LP3	*H. neanderthalensis*	Le Moustier, France	*				
Le Moustier 1	LP4	*H. neanderthalensis*	Le Moustier, France	*				
Le Moustier 1	UP4	*H. neanderthalensis*	Le Moustier, France		*			
M71-C10 F12-93 (M13A	UI1	*H. neanderthalensis*	Les Pradelles, France				*	
M71-C10 F12-93 (M13B)	UI1	*H. neanderthalensis*	Les Pradelles, France				*	
M71-C10 F12-93 (M13C)	UI2	*H. neanderthalensis*	Les Pradelles, France				*	
Mezmaskaiya	LdM1	*H. neanderthalensis*	Mezmaskaiya, Russia					*
Molare	LdM1	*H. neanderthalensis*	Molare, Italy					*
Molare	LdM2	*H. neanderthalensis*	Molare, Italy					*
Pech de l'Aze	LdM1	*H. neanderthalensis*	Pech de l'Aze, France	*				*
Pech de l'Aze	LdM2	*H. neanderthalensis*	Pech de l'Aze, France	*				*
Regourdou 1	LI2	*H. neanderthalensis*	Regourdou, France				*	
Regourdou 1	LC	*H. neanderthalensis*	Regourdou, France				*	
Regourdou 1	LM1	*H. neanderthalensis*	Regourdou, France	*			*	
Regourdou 1	LM2	*H. neanderthalensis*	Regourdou, France	*			*	
Regourdou 1	LM3	*H. neanderthalensis*	Regourdou, France	*	*		*	
Regourdou 1	LP3	*H. neanderthalensis*	Regourdou, France	*				
Regourdou 1	LP4	*H. neanderthalensis*	Regourdou, France	*	*			
Roc de Marsal	LM1	*H. neanderthalensis*	Roc de Marsal, France	*		*		
Roc de Marsal	LdM1	*H. neanderthalensis*	Roc de Marsal, France	*				*
Roc de Marsal	LdM2	*H. neanderthalensis*	Roc de Marsal, France	*				*
Saint-Césaire	UI1	*H. neanderthalensis*	Saint-Césaire, France				*	
Saint-Césaire	UI2	*H. neanderthalensis*	Saint-Césaire, France				*	
Saint-Césaire	LI2	*H. neanderthalensis*	Saint-Césaire, France				*	
Saint-Césaire	LC	*H. neanderthalensis*	Saint-Césaire, France				*	
Saint-Césaire	LM1	*H. neanderthalensis*	Saint-Césaire, France	*			*	
Saint-Césaire	LM2	*H. neanderthalensis*	Saint-Césaire, France	*				
Saint-Césaire	LM3	*H. neanderthalensis*	Saint-Césaire, France	*	*		*	
Saint-Césaire	LP3	*H. neanderthalensis*	Saint-Césaire, France	*				
Saint-Césaire	LP4	*H. neanderthalensis*	Saint-Césaire, France	*				

Scla-4A-1	LM1	*H. neanderthalensis*	Scladina, Belgium	*		*	*	
Scla-4A-1	LM2	*H. neanderthalensis*	Scladina, Belgium	*				
Scla-4A-1	LM3	*H. neanderthalensis*	Scladina, Belgium	*	*			
Scla-4A-1	LP4	*H. neanderthalensis*	Scladina, Belgium	*				
Scla-4A-11	UI1	*H. neanderthalensis*	Scladina, Belgium				*	
Scla-4A-12	LC	*H. neanderthalensis*	Scladina, Belgium				*	
Scla-4A-13	LdM2	*H. neanderthalensis*	Scladina, Belgium					*
Scla-4A-14	UI2	*H. neanderthalensis*	Scladina, Belgium				*	
Scla-4A-2	UP4	*H. neanderthalensis*	Scladina, Belgium		*			
Scla-4A-20	LI2	*H. neanderthalensis*	Scladina, Belgium				*	
Scla-4A-6	LP3	*H. neanderthalensis*	Scladina, Belgium	*				
Spy 110c	LdM2	*H. neanderthalensis*	Spy, Belgium					*
Spy 37b	LdM2	*H. neanderthalensis*	Spy, Belgium					*
Spy 549K	LdM1	*H. neanderthalensis*	Spy, Belgium					*
Spy I	LI2	*H. neanderthalensis*	Spy, Belgium				*	
Spy I	LC	*H. neanderthalensis*	Spy, Belgium				*	
Vi 11.39	LM1	*H. neanderthalensis*	Vindija, Croatia	*				
Vi 11.39	LM2	*H. neanderthalensis*	Vindija, Croatia	*				
Vi 11.39	LM3	*H. neanderthalensis*	Vindija, Croatia	*	*			
Vi 11.45	LM1	*H. neanderthalensis*	Vindija, Croatia	*				
Vi 11.45	LM2	*H. neanderthalensis*	Vindija, Croatia	*				
Vi 11.45	LM3	*H. neanderthalensis*	Vindija, Croatia	*				
Cavallo H8	LdM2	early *H. sapiens*	Cavallo Cave, Italy					*
Cave of Hearths	LM1	early *H. sapiens*	Cave of Hearths, South Africa				*	
Dar es Soltane H4	LI2	early *H. sapiens*	Dar es Soltane, Morocco				*	
Dar es Soltane H4	LC	early *H. sapiens*	Dar es Soltane, Morocco				*	
Dar es Soltane H4	LM1	early *H. sapiens*	Dar es Soltane, Morocco	*			*	
Dar es Soltane H4	LM2	early *H. sapiens*	Dar es Soltane, Morocco	*			*	
Dar es Soltane H4	LM3	early *H. sapiens*	Dar es Soltane, Morocco	*	*			
Dar es Soltane H4	LP3	early *H. sapiens*	Dar es Soltane, Morocco	*				
Dar es Soltane H5	LM1	early *H. sapiens*	Dar es Soltane, Morocco	*			*	
Dar es Soltane H5	LM2	early *H. sapiens*	Dar es Soltane, Morocco	*			*	
Dar es Soltane H5	LM3	early *H. sapiens*	Dar es Soltane, Morocco	*			*	
Dar es Soltane H5	LP4	early *H. sapiens*	Dar es Soltane, Morocco	*				
DK 6246	LdM1	early *H. sapiens*	Die Kelders, South Africa					*
DK 6291	LdM1	early *H. sapiens*	Die Kelders, South Africa					*
SAM AP 6242	LM1	early *H. sapiens*	Die Kelders, South Africa				*	
El Harhoura	LM1	early *H. sapiens*	El Harhoura, Morocco	*			*	
El Harhoura	LM2	early *H. sapiens*	El Harhoura, Morocco	*			*	
El Harhoura	LM3	early *H. sapiens*	El Harhoura, Morocco	*	*		*	
El Harhoura	LP4	early *H. sapiens*	El Harhoura, Morocco	*	*			
Equus Cave H71-33	LM2	early *H. sapiens*	Equus Cave, South Africa				*	
Equus Cave H71-33	LM3	early *H. sapiens*	Equus Cave, South Africa				*	
Irhoud 11	LI2	early *H. sapiens*	Jebel Irhoud, Morocco				*	
Irhoud 11	LC	early *H. sapiens*	Jebel Irhoud, Morocco				*	
Irhoud 11	LM1	early *H. sapiens*	Jebel Irhoud, Morocco	*			*	
Irhoud 11	LM2	early *H. sapiens*	Jebel Irhoud, Morocco	*			*	
Irhoud 11	LM3	early *H. sapiens*	Jebel Irhoud, Morocco	*	*		*	
Irhoud 11	LP3	early *H. sapiens*	Jebel Irhoud, Morocco	*				
Irhoud 11	LP4	early *H. sapiens*	Jebel Irhoud, Morocco	*	*			
Irhoud 3	LdM2	early *H. sapiens*	Jebel Irhoud, Morocco					*
Irhoud 3	LM1	early *H. sapiens*	Jebel Irhoud, Morocco	*		*		
Irhoud 3	LM2	early *H. sapiens*	Jebel Irhoud, Morocco	*				
Irhoud 3	LP3	early *H. sapiens*	Jebel Irhoud, Morocco	*				
Irhoud 3	LP4	early *H. sapiens*	Jebel Irhoud, Morocco	*	*			
KebA5N13-463	LI2	early *H. sapiens*	Kebara, Israel				*	
Qafzeh 10	LdM1	early *H. sapiens*	Qafzeh, Israel					*
Qafzeh 10	LM1	early *H. sapiens*	Qafzeh, Israel	*		*		
Qafzeh 10	LM2	early *H. sapiens*	Qafzeh, Israel	*				
Qafzeh 10	LP3	early *H. sapiens*	Qafzeh, Israel	*				
Qafzeh 10	UP4	early *H. sapiens*	Qafzeh, Israel		*			
Qafzeh 11	UI1	early *H. sapiens*	Qafzeh, Israel				*	
Qafzeh 11	UI2	early *H. sapiens*	Qafzeh, Israel				*	
Qafzeh 11	LI2	early *H. sapiens*	Qafzeh, Israel				*	

Qafzeh 11	LC	early *H. sapiens*	Qafzeh, Israel				*	
Qafzeh 11	LM1	early *H. sapiens*	Qafzeh, Israel	*			*	
Qafzeh 11	LM2	early *H. sapiens*	Qafzeh, Israel	*				
Qafzeh 11	LP3	early *H. sapiens*	Qafzeh, Israel	*				
Qafzeh 11	LP4	early *H. sapiens*	Qafzeh, Israel	*	*			
Qafzeh 11	UP4	early *H. sapiens*	Qafzeh, Israel		*			
Qafzeh 12	LM3	early *H. sapiens*	Qafzeh, Israel				*	
Qafzeh 15	LdM1	early *H. sapiens*	Qafzeh, Israel					*
Qafzeh 15	LdM2	early *H. sapiens*	Qafzeh, Israel	*				*
Qafzeh 15	LM1	early *H. sapiens*	Qafzeh, Israel	*		*		
Qafzeh 15	LM2	early *H. sapiens*	Qafzeh, Israel	*				
Qafzeh 15	UP4	early *H. sapiens*	Qafzeh, Israel		*			
Qafzeh 2	LM1	early *H. sapiens*	Qafzeh, Israel				*	
Qafzeh 2	LM2	early *H. sapiens*	Qafzeh, Israel				*	
Qafzeh 2	LM3	early *H. sapiens*	Qafzeh, Israel				*	
Qafzeh 26	LM1	early *H. sapiens*	Qafzeh, Israel				*	
Qafzeh 8	LI2	early *H. sapiens*	Qafzeh, Israel				*	
Qafzeh 8	LC	early *H. sapiens*	Qafzeh, Israel				*	
Qafzeh 9	UI2	early *H. sapiens*	Qafzeh, Israel				*	
Qafzeh 9	LI2	early *H. sapiens*	Qafzeh, Israel				*	
Qafzeh 9	LC	early *H. sapiens*	Qafzeh, Israel				*	
Qafzeh 9	LM1	early *H. sapiens*	Qafzeh, Israel	*			*	
Qafzeh 9	LM2	early *H. sapiens*	Qafzeh, Israel	*			*	
Qafzeh 9	LM3	early *H. sapiens*	Qafzeh, Israel	*	*		*	
Qafzeh 9	LP3	early *H. sapiens*	Qafzeh, Israel	*				
Qafzeh 9	LP4	early *H. sapiens*	Qafzeh, Israel	*	*			
Qafzeh 9	UP4	early *H. sapiens*	Qafzeh, Israel		*			
Skhul 1	LdM2	early *H. sapiens*	Skhul, Israel					*
Skhul 10	LdM2	early *H. sapiens*	Skhul, Israel	*				*
Skhul IV	UI1	early *H. sapiens*	Skhul, Israel				*	
Skhul IV	UI2	early *H. sapiens*	Skhul, Israel				*	
Skhul IV	LI2	early *H. sapiens*	Skhul, Israel				*	
Skhul IV	LC	early *H. sapiens*	Skhul, Israel				*	
Skhul IV	LM1	early *H. sapiens*	Skhul, Israel	*				
Skhul IV	LM2	early *H. sapiens*	Skhul, Israel	*				
Skhul IV	LM3	early *H. sapiens*	Skhul, Israel	*				
Skhul IV	LP3	early *H. sapiens*	Skhul, Israel	*				
Skhul IV	LP4	early *H. sapiens*	Skhul, Israel	*				
Tabun C2	LM1	early *H. sapiens*	Tabun, Israel	*			*	
Tabun C2	LM2	early *H. sapiens*	Tabun, Israel	*			*	
Tabun C2	LM3	early *H. sapiens*	Tabun, Israel	*			*	
Tabun C2	LP3	early *H. sapiens*	Tabun, Israel	*				
Tabun C2	LP4	early *H. sapiens*	Tabun, Israel	*				
Tabun C2	LI2	early *H. sapiens*	Tabun, Israel				*	
Tabun C2	LC	early *H. sapiens*	Tabun, Israel				*	
Temara	LI2	early *H. sapiens*	G des Contrebandiers, Morocco				*	
Temara	LC	early *H. sapiens*	G des Contrebandiers, Morocco				*	
Temara	LM1	early *H. sapiens*	G des Contrebandiers, Morocco	*			*	
Temara	LM2	early *H. sapiens*	G des Contrebandiers, Morocco	*			*	
Temara	LM3	early *H. sapiens*	G des Contrebandiers, Morocco	*	*		*	
Temara	LP3	early *H. sapiens*	G des Contrebandiers, Morocco	*				
Temara	LP4	early *H. sapiens*	G des Contrebandiers, Morocco	*	*			
SA 12330	LdM1	recent *H. sapiens*	Am Mus Natural History, USA					*
SA 18331	LdM1	recent *H. sapiens*	Am Mus Natural History, USA					*
SA 18977	LdM1	recent *H. sapiens*	Am Mus Natural History, USA					*
SA 5896	LdM1	recent *H. sapiens*	Am Mus Natural History, USA					*
SA 5897	LdM1	recent *H. sapiens*	Am Mus Natural History, USA					*
SA 5918	LdM1	recent *H. sapiens*	Am Mus Natural History, USA					*
SA 99-1-108	LdM1	recent *H. sapiens*	Am Mus Natural History, USA					*
SA 99-1-167	LdM1	recent *H. sapiens*	Am Mus Natural History, USA					*
SA 99-1-221	LdM1	recent *H. sapiens*	Am Mus Natural History, USA					*
SA 99-1-224	LdM1	recent *H. sapiens*	Am Mus Natural History, USA					*
SA 99-1-280	LdM1	recent *H. sapiens*	Am Mus Natural History, USA					*
SA 99-1-876	LdM1	recent *H. sapiens*	Am Mus Natural History, USA					*

SAM 4957	LdM2	recent *H. sapiens*	Am Mus Natural History, USA					*
SAM 5889	LdM2	recent *H. sapiens*	Am Mus Natural History, USA					*
SAM 5895	LdM2	recent *H. sapiens*	Am Mus Natural History, USA					*
SAM 5896	LdM2	recent *H. sapiens*	Am Mus Natural History, USA					*
SAM 9769	LdM2	recent *H. sapiens*	Am Mus Natural History, USA					*
SAM 99-1-108	LdM2	recent *H. sapiens*	Am Mus Natural History, USA					*
SAM 99-1-876	LdM2	recent *H. sapiens*	Am Mus Natural History, USA					*
R1101_1498	LM1	recent *H. sapiens*	Anth Institute, Romania	*		*	*	
R1101_1498	LM2	recent *H. sapiens*	Anth Institute, Romania	*			*	
R1101_1498	LM3	recent *H. sapiens*	Anth Institute, Romania	*	*			
R1119-2349	LI2	recent *H. sapiens*	Anth Institute, Romania				*	
R1119-2349	UI1	recent *H. sapiens*	Anth Institute, Romania				*	
R1119-2349	UI2	recent *H. sapiens*	Anth Institute, Romania				*	
R1140_899	LM1	recent *H. sapiens*	Anth Institute, Romania				*	
R1140_899	LM3	recent *H. sapiens*	Anth Institute, Romania				*	
R1160_440	LM1	recent *H. sapiens*	Anth Institute, Romania	*		*	*	
R123	UI1	recent *H. sapiens*	Anth Institute, Romania				*	
R123	UI2	recent *H. sapiens*	Anth Institute, Romania				*	
R123	LI2	recent *H. sapiens*	Anth Institute, Romania				*	
R123	LM1	recent *H. sapiens*	Anth Institute, Romania	*		*		
R123	LM2	recent *H. sapiens*	Anth Institute, Romania	*				
R1586-2425	LM3	recent *H. sapiens*	Anth Institute, Romania	*	*			
R1620-2480	LM3	recent *H. sapiens*	Anth Institute, Romania	*	*		*	
R1639-1186	UI2	recent *H. sapiens*	Anth Institute, Romania				*	
R1639-1186	LM2	recent *H. sapiens*	Anth Institute, Romania	*				
R165	UI1	recent *H. sapiens*	Anth Institute, Romania				*	
R165	UI2	recent *H. sapiens*	Anth Institute, Romania				*	
R165	LI2	recent *H. sapiens*	Anth Institute, Romania				*	
R167-I75	LM1	recent *H. sapiens*	Anth Institute, Romania	*		*	*	
R1719-1237	LM2	recent *H. sapiens*	Anth Institute, Romania	*			*	
R1719-1237	LM3	recent *H. sapiens*	Anth Institute, Romania	*	*		*	
R186	LM3	recent *H. sapiens*	Anth Institute, Romania	*	*		*	
R1989-1382	LM1	recent *H. sapiens*	Anth Institute, Romania	*		*	*	
R1989-1382	LM2	recent *H. sapiens*	Anth Institute, Romania	*				
R2064_1420	UI1	recent *H. sapiens*	Anth Institute, Romania				*	
R2070-1423	LM2	recent *H. sapiens*	Anth Institute, Romania	*				
R2207-1493	LM2	recent *H. sapiens*	Anth Institute, Romania	*			*	
R2386-1561	UI2	recent *H. sapiens*	Anth Institute, Romania				*	
R239	UI1	recent *H. sapiens*	Anth Institute, Romania				*	
R2433_1156	UI1	recent *H. sapiens*	Anth Institute, Romania				*	
R2525-1641	LM2	recent *H. sapiens*	Anth Institute, Romania	*				
R258-144	LM1	recent *H. sapiens*	Anth Institute, Romania	*		*	*	
R258-144	LM2	recent *H. sapiens*	Anth Institute, Romania	*				
R2602-1673	LM1	recent *H. sapiens*	Anth Institute, Romania	*		*	*	
R2643_1941	UI1	recent *H. sapiens*	Anth Institute, Romania				*	
R433	LM1	recent *H. sapiens*	Anth Institute, Romania				*	
R433	LM2	recent *H. sapiens*	Anth Institute, Romania	*				
R463	LM3	recent *H. sapiens*	Anth Institute, Romania				*	
R488-274	LM1	recent *H. sapiens*	Anth Institute, Romania	*		*	*	
R605	LM2	recent *H. sapiens*	Anth Institute, Romania				*	
R605	LI2	recent *H. sapiens*	Anth Institute, Romania				*	
R605-1185	LI2	recent *H. sapiens*	Anth Institute, Romania				*	
R605-1185	UI1	recent *H. sapiens*	Anth Institute, Romania				*	
R605-1185	UI2	recent *H. sapiens*	Anth Institute, Romania				*	
R605-1185	LM1	recent *H. sapiens*	Anth Institute, Romania	*		*		
R605-1185	LM3	recent *H. sapiens*	Anth Institute, Romania	*	*			
R690-1372	LM2	recent *H. sapiens*	Anth Institute, Romania				*	
R752-692	LM2	recent *H. sapiens*	Anth Institute, Romania	*			*	
R913-759	LM2	recent *H. sapiens*	Anth Institute, Romania				*	
Arene Candide 2	LM1	recent *H. sapiens*	Arene Candide, Italy	*				
Arene Candide 2	LM2	recent *H. sapiens*	Arene Candide, Italy	*				
Arene Candide 2	LM3	recent *H. sapiens*	Arene Candide, Italy	*				
Arene Candide 2	LP3	recent *H. sapiens*	Arene Candide, Italy	*				
Arene Candide 2	LP4	recent *H. sapiens*	Arene Candide, Italy	*	*			

Balla Barlang	LdM1	recent *H. sapiens*	Balla Barlang, Hungary					*
Barma Grande 2	LM1	recent *H. sapiens*	Barma Grande, Italy	*				
Barma Grande 2	LM2	recent *H. sapiens*	Barma Grande, Italy	*				
Barma Grande 2	LM3	recent *H. sapiens*	Barma Grande, Italy	*				
Barma Grande 2	LP4	recent *H. sapiens*	Barma Grande, Italy	*				
Barma Grande 4	LM1	recent *H. sapiens*	Barma Grande, Italy	*				
Barma Grande 4	LM2	recent *H. sapiens*	Barma Grande, Italy	*				
Barma Grande 4	LM3	recent *H. sapiens*	Barma Grande, Italy	*				
Barma Grande 4	LP3	recent *H. sapiens*	Barma Grande, Italy	*				
Barma Grande 4	LP4	recent *H. sapiens*	Barma Grande, Italy	*				
Barma Grande 5	LM1	recent *H. sapiens*	Barma Grande, Italy	*				
Barma Grande 5	LM2	recent *H. sapiens*	Barma Grande, Italy	*				
Barma Grande 5	LM3	recent *H. sapiens*	Barma Grande, Italy	*				
Barma Grande 5	LP3	recent *H. sapiens*	Barma Grande, Italy	*				
Barma Grande 5	LP4	recent *H. sapiens*	Barma Grande, Italy	*	*			
Bruniquel II	LdM2	recent *H. sapiens*	Bruniquel, France					*
CH1 B3	LdM1	recent *H. sapiens*	Chauveau, France					*
Combe Capelle	UP4	recent *H. sapiens*	Combe Capelle, France		*			
Combe Capelle	UI1	recent *H. sapiens*	Combe Capelle, France				*	
Combe Capelle	UI2	recent *H. sapiens*	Combe Capelle, France				*	
Combe Capelle	LI2	recent *H. sapiens*	Combe Capelle, France				*	
Combe Capelle	LC	recent *H. sapiens*	Combe Capelle, France				*	
DV 13-1	UP4	recent *H. sapiens*	Dolni Vestonice, Czech Rep		*			
DV 13-2	LM1	recent *H. sapiens*	Dolni Vestonice, Czech Rep	*				
DV 13-2	LM2	recent *H. sapiens*	Dolni Vestonice, Czech Rep	*				
DV 13-2	LM3	recent *H. sapiens*	Dolni Vestonice, Czech Rep	*				
DV 13-2	LP3	recent *H. sapiens*	Dolni Vestonice, Czech Rep	*				
DV 13-2	LP4	recent *H. sapiens*	Dolni Vestonice, Czech Rep	*				
DV 14-1	UP4	recent *H. sapiens*	Dolni Vestonice, Czech Rep		*			
DV 14-7	LM1	recent *H. sapiens*	Dolni Vestonice, Czech Rep	*				
DV 14-7	LM2	recent *H. sapiens*	Dolni Vestonice, Czech Rep	*				
DV 14-7	LM3	recent *H. sapiens*	Dolni Vestonice, Czech Rep	*				
DV 14-7	LP3	recent *H. sapiens*	Dolni Vestonice, Czech Rep	*				
DV 14-7	LP4	recent *H. sapiens*	Dolni Vestonice, Czech Rep	*	*			
DV 15-4	LM1	recent *H. sapiens*	Dolni Vestonice, Czech Rep	*				
DV 15-4	LM2	recent *H. sapiens*	Dolni Vestonice, Czech Rep	*				
DV 15-4	LM3	recent *H. sapiens*	Dolni Vestonice, Czech Rep	*				
DV 15-4	LP3	recent *H. sapiens*	Dolni Vestonice, Czech Rep	*				
DV 15-4	LP4	recent *H. sapiens*	Dolni Vestonice, Czech Rep	*				
Estelas	LdM1	recent *H. sapiens*	Estelas, France					*
Estelas	LdM2	recent *H. sapiens*	Estelas, France					*
Fontechevade	LdM2	recent *H. sapiens*	Fontechevade, France					*
GUID_T_39	LdM1	recent *H. sapiens*	Guidizzolo, Italy	*				
GUID_T_41	LdM2	recent *H. sapiens*	Guidizzolo, Italy	*				
GUID_T_49	LdM2	recent *H. sapiens*	Guidizzolo, Italy	*				
GUID_T_52	LdM2	recent *H. sapiens*	Guidizzolo, Italy	*				
GUID_T_54	LdM2	recent *H. sapiens*	Guidizzolo, Italy	*				
GUID_T_65	LdM1	recent *H. sapiens*	Guidizzolo, Italy	*				
GUID_T_77	LdM1	recent *H. sapiens*	Guidizzolo, Italy	*				
GUID_T_80	LdM2	recent *H. sapiens*	Guidizzolo, Italy	*				
GUID_TB_49_B	LdM1	recent *H. sapiens*	Guidizzolo, Italy	*				
Hayonim 17	LI2	recent *H. sapiens*	Hayonim, Israel				*	
Hayonim 17	LC	recent *H. sapiens*	Hayonim, Israel				*	
Hayonim 19	UI2	recent *H. sapiens*	Hayonim, Israel				*	
Hayonim 19	LI2	recent *H. sapiens*	Hayonim, Israel				*	
Hayonim 19	LC	recent *H. sapiens*	Hayonim, Israel				*	
Hayonim 20	LI2	recent *H. sapiens*	Hayonim, Israel				*	
Hayonim 20	LC	recent *H. sapiens*	Hayonim, Israel				*	
Hayonim 25	UI1	recent *H. sapiens*	Hayonim, Israel				*	
Hayonim 8	UI1	recent *H. sapiens*	Hayonim, Israel				*	
Hayonim 8	UI2	recent *H. sapiens*	Hayonim, Israel				*	
Hayonim 8	LI2	recent *H. sapiens*	Hayonim, Israel				*	
Hayonim 8	LC	recent *H. sapiens*	Hayonim, Israel				*	
WAF 2903	LdM1	recent *H. sapiens*	Inst Pal Humain, France					*

WAF 2904	LdM1	recent *H. sapiens*	Inst Pal Humain, France					*
Isturitz III 1950-6	LdM2	recent *H. sapiens*	Isturitz, France					*
Isturitz III 1950-5-1	LdM2	recent *H. sapiens*	Isturitz, France					*
Isturitz III 1950-7	LdM1	recent *H. sapiens*	Isturitz, France					*
Isturitz III 1950-7	LdM2	recent *H. sapiens*	Isturitz, France					*
Kostenki 12	LdM2	recent *H. sapiens*	Kostenki, Russia					*
Kostenki 15	LdM2	recent *H. sapiens*	Kostenki, Russia					*
La Madeleine	LdM1	recent *H. sapiens*	La Madeleine, France					*
La Madeleine	LdM2	recent *H. sapiens*	La Madeleine, France					*
Lagar Velho	LdM1	recent *H. sapiens*	Lagar Velho, Portugal					*
Lagar Velho	LdM2	recent *H. sapiens*	Lagar Velho, Portugal					*
Figuier	LdM1	recent *H. sapiens*	Le Figuier, France					*
Figuier	LdM2	recent *H. sapiens*	Le Figuier, France					*
Maurenne Cave A38	LdM1	recent *H. sapiens*	Maurenne Cave, Belgium					*
Miesslingtal	LdM2	recent *H. sapiens*	Miesslingtal, Austria					*
MPI_07_078_28	UP4	recent *H. sapiens*	MPI Clinical, Germany		*			
MPI_07_114_28	UP4	recent *H. sapiens*	MPI Clinical, Germany		*			
MPI_07_124	UI1	recent *H. sapiens*	MPI Clinical, Germany				*	
MPI_07_155	UI1	recent *H. sapiens*	MPI Clinical, Germany				*	
MPI_07_208	LI2	recent *H. sapiens*	MPI Clinical, Germany				*	
MPI_07_243	UI1	recent *H. sapiens*	MPI Clinical, Germany				*	
MPI_07_295	UI2	recent *H. sapiens*	MPI Clinical, Germany				*	
MPI_07_355	UI1	recent *H. sapiens*	MPI Clinical, Germany				*	
MPI_07_373	LI2	recent *H. sapiens*	MPI Clinical, Germany				*	
MPI_07_386	UI1	recent *H. sapiens*	MPI Clinical, Germany				*	
MPI_07_387	UI2	recent *H. sapiens*	MPI Clinical, Germany				*	
MPI_07_397	UI2	recent *H. sapiens*	MPI Clinical, Germany				*	
MPI_07_493	UI1	recent *H. sapiens*	MPI Clinical, Germany				*	
MPI_07_518	UI2	recent *H. sapiens*	MPI Clinical, Germany				*	
MPI_07_565_28	UP4	recent *H. sapiens*	MPI Clinical, Germany		*			
MPI_07_602_28	UP4	recent *H. sapiens*	MPI Clinical, Germany		*			
MPI_07_617_28	UP4	recent *H. sapiens*	MPI Clinical, Germany		*			
MPI_07_724_28	UP4	recent *H. sapiens*	MPI Clinical, Germany		*			
MPI_08_367	UI2	recent *H. sapiens*	MPI Clinical, Germany				*	
MPI_08_368	UI1	recent *H. sapiens*	MPI Clinical, Germany				*	
MPI_09_001	UI2	recent *H. sapiens*	MPI Clinical, Germany				*	
MPI_09_002	UI1	recent *H. sapiens*	MPI Clinical, Germany				*	
MPI_09_003	UI2	recent *H. sapiens*	MPI Clinical, Germany				*	
MPI_09_012	UI1	recent *H. sapiens*	MPI Clinical, Germany				*	
MPI_09_069	LI2	recent *H. sapiens*	MPI Clinical, Germany				*	
MPI_09_078	LI2	recent *H. sapiens*	MPI Clinical, Germany				*	
MPI_09_083	LI2	recent *H. sapiens*	MPI Clinical, Germany				*	
MPI_09_116	LI2	recent *H. sapiens*	MPI Clinical, Germany				*	
MPI_09_146	UI2	recent *H. sapiens*	MPI Clinical, Germany				*	
MPI_09_149	UI2	recent *H. sapiens*	MPI Clinical, Germany				*	
MPI_09_150	UI1	recent *H. sapiens*	MPI Clinical, Germany				*	
MPI_09_162	UI2	recent *H. sapiens*	MPI Clinical, Germany				*	
MPI_09_164	UI1	recent *H. sapiens*	MPI Clinical, Germany				*	
MPI_09_168	LI2	recent *H. sapiens*	MPI Clinical, Germany				*	
MPI_09_214	LI2	recent *H. sapiens*	MPI Clinical, Germany				*	
MPI_09_216	UI2	recent *H. sapiens*	MPI Clinical, Germany				*	
MPI_09_218	LI2	recent *H. sapiens*	MPI Clinical, Germany				*	
MPI_09_219	LI2	recent *H. sapiens*	MPI Clinical, Germany				*	
MPI_09_221	UI1	recent *H. sapiens*	MPI Clinical, Germany				*	
MPI_09_222	UI1	recent *H. sapiens*	MPI Clinical, Germany				*	
MPI_09_224	UI2	recent *H. sapiens*	MPI Clinical, Germany				*	
MPI_09_252	LI2	recent *H. sapiens*	MPI Clinical, Germany				*	
MPI_09_254	UI1	recent *H. sapiens*	MPI Clinical, Germany				*	
MPI_09_256	LI2	recent *H. sapiens*	MPI Clinical, Germany				*	
MPI_M12_28	UP4	recent *H. sapiens*	MPI Clinical, Germany		*			
MPI_T08_046_28	UP4	recent *H. sapiens*	MPI Clinical, Germany		*			
MPI_T08_062_28	UP4	recent *H. sapiens*	MPI Clinical, Germany		*			
MPI_T08_076_28	UP4	recent *H. sapiens*	MPI Clinical, Germany		*			
MPI_T08_081_28	UP4	recent *H. sapiens*	MPI Clinical, Germany		*			

MPI_T08_094_28	UP4	recent *H. sapiens*	MPI Clinical, Germany		*			
MPI_T09_125_28	UP4	recent *H. sapiens*	MPI Clinical, Germany		*			
MPI_T09_127_28	UP4	recent *H. sapiens*	MPI Clinical, Germany		*			
MPI_T09_311_28	UP4	recent *H. sapiens*	MPI Clinical, Germany		*			
M10	LI2	recent *H. sapiens*	MPI Clinical, Germany				*	
M10	UI2	recent *H. sapiens*	MPI Clinical, Germany				*	
M123	LM2	recent *H. sapiens*	MPI Clinical, Germany	*				
M131	LM3	recent *H. sapiens*	MPI Clinical, Germany	*	*			
M132	LM3	recent *H. sapiens*	MPI Clinical, Germany	*	*			
M133	LM3	recent *H. sapiens*	MPI Clinical, Germany	*	*			
M135	LM3	recent *H. sapiens*	MPI Clinical, Germany	*	*			
M139	LP3	recent *H. sapiens*	MPI Clinical, Germany	*				
M14	LP4	recent *H. sapiens*	MPI Clinical, Germany	*	*			
M145	LM2	recent *H. sapiens*	MPI Clinical, Germany	*				
M146	LM2	recent *H. sapiens*	MPI Clinical, Germany	*				
M15	UI1	recent *H. sapiens*	MPI Clinical, Germany				*	
M162	LM2	recent *H. sapiens*	MPI Clinical, Germany	*				
M181	LM2	recent *H. sapiens*	MPI Clinical, Germany	*				
M185	LM2	recent *H. sapiens*	MPI Clinical, Germany	*				
M19	LM2	recent *H. sapiens*	MPI Clinical, Germany	*			*	
M21	UI1	recent *H. sapiens*	MPI Clinical, Germany				*	
M213	LM1	recent *H. sapiens*	MPI Clinical, Germany				*	
M213	LM3	recent *H. sapiens*	MPI Clinical, Germany	*	*			
M232	LM2	recent *H. sapiens*	MPI Clinical, Germany	*			*	
M3	LM1	recent *H. sapiens*	MPI Clinical, Germany	*		*		
M34	LP3	recent *H. sapiens*	MPI Clinical, Germany	*				
M35	LP3	recent *H. sapiens*	MPI Clinical, Germany	*				
M5	LM1	recent *H. sapiens*	MPI Clinical, Germany	*		*	*	
M53	LM2	recent *H. sapiens*	MPI Clinical, Germany	*				
M6	LM1	recent *H. sapiens*	MPI Clinical, Germany				*	
M6	LM2	recent *H. sapiens*	MPI Clinical, Germany	*				
M71	LM3	recent *H. sapiens*	MPI Clinical, Germany	*	*			
EAF 17590	LdM2	recent *H. sapiens*	Musee de l'Homme, France					*
EAF 17590	LdM1	recent *H. sapiens*	Musee de l'Homme, France					*
EAF 19226	LdM1	recent *H. sapiens*	Musee de l'Homme, France					*
EAF 25068	LdM2	recent *H. sapiens*	Musee de l'Homme, France					*
EAF 25068	LdM1	recent *H. sapiens*	Musee de l'Homme, France					*
EAF 25088	LdM2	recent *H. sapiens*	Musee de l'Homme, France					*
EAF 25088	LdM1	recent *H. sapiens*	Musee de l'Homme, France					*
EAF 25119	LdM2	recent *H. sapiens*	Musee de l'Homme, France					*
EAF 25119	LdM1	recent *H. sapiens*	Musee de l'Homme, France					*
EAF 25123	LdM1	recent *H. sapiens*	Musee de l'Homme, France					*
EAF 2899	LdM1	recent *H. sapiens*	Musee de l'Homme, France					*
EAF 4641	LdM1	recent *H. sapiens*	Musee de l'Homme, France					*
EAF 7340	LdM2	recent *H. sapiens*	Musee de l'Homme, France					*
EAF 7344	LdM2	recent *H. sapiens*	Musee de l'Homme, France					*
EAF 7344	LdM1	recent *H. sapiens*	Musee de l'Homme, France					*
EAF 7361	LdM2	recent *H. sapiens*	Musee de l'Homme, France					*
EAF 7361	LdM1	recent *H. sapiens*	Musee de l'Homme, France					*
EGY 18447	LdM2	recent *H. sapiens*	Musee de l'Homme, France					*
EGY 18447	LdM1	recent *H. sapiens*	Musee de l'Homme, France					*
EGY 7330	LdM1	recent *H. sapiens*	Musee de l'Homme, France					*
EGY 7341	LdM1	recent *H. sapiens*	Musee de l'Homme, France					*
EGY 7345	LdM2	recent *H. sapiens*	Musee de l'Homme, France					*
EGY 7345	LdM1	recent *H. sapiens*	Musee de l'Homme, France					*
EGY 7359	LdM1	recent *H. sapiens*	Musee de l'Homme, France					*
Nahal-Oren 14	LI2	recent *H. sapiens*	Nahal-Oren, Israel				*	
Nahal-Oren 14	LC	recent *H. sapiens*	Nahal-Oren, Israel				*	
Nahal-Oren 16	UI1	recent *H. sapiens*	Nahal-Oren, Israel				*	
Nahal-Oren 16	UI2	recent *H. sapiens*	Nahal-Oren, Israel				*	
Nahal-Oren 24	UI1	recent *H. sapiens*	Nahal-Oren, Israel				*	
Nahal-Oren 24	UI2	recent *H. sapiens*	Nahal-Oren, Israel				*	
Nahal-Oren 8	LI2	recent *H. sapiens*	Nahal-Oren, Israel				*	
Nahal-Oren 8	LC	recent *H. sapiens*	Nahal-Oren, Israel				*	

AUS 1971-187	LdM1	recent *H. sapiens*	Nat History Mus, U Kingdom					*
AUS 30-444	LdM1	recent *H. sapiens*	Nat History Mus, U Kingdom					*
IN 1910-9-5-1	LdM2	recent *H. sapiens*	Nat History Mus, U Kingdom					*
IN 1949-12-7-19	LdM1	recent *H. sapiens*	Nat History Mus, U Kingdom					*
IN 58-6-24-154	LdM1	recent *H. sapiens*	Nat History Mus, U Kingdom					*
IN 6-8501	LdM1	recent *H. sapiens*	Nat History Mus, U Kingdom					*
IN 6-8639	LdM1	recent *H. sapiens*	Nat History Mus, U Kingdom					*
IN 6-8640	LdM1	recent *H. sapiens*	Nat History Mus, U Kingdom					*
IN 6-8641	LdM1	recent *H. sapiens*	Nat History Mus, U Kingdom					*
IN 6-8642	LdM1	recent *H. sapiens*	Nat History Mus, U Kingdom					*
IN 6-9093	LdM1	recent *H. sapiens*	Nat History Mus, U Kingdom					*
IN 6-9773	LdM1	recent *H. sapiens*	Nat History Mus, U Kingdom					*
IND 1949-12-7-19	LdM2	recent *H. sapiens*	Nat History Mus, U Kingdom					*
IND 58-6-24-154	LdM2	recent *H. sapiens*	Nat History Mus, U Kingdom					*
IND 6-9773	LdM2	recent *H. sapiens*	Nat History Mus, U Kingdom					*
PB 1009	LdM2	recent *H. sapiens*	Nat History Mus, U Kingdom					*
PB 1009	LdM1	recent *H. sapiens*	Nat History Mus, U Kingdom					*
PB 1046	LdM2	recent *H. sapiens*	Nat History Mus, U Kingdom					*
PB 1046	LdM1	recent *H. sapiens*	Nat History Mus, U Kingdom					*
PB 1132	LdM1	recent *H. sapiens*	Nat History Mus, U Kingdom					*
PB 124	LdM2	recent *H. sapiens*	Nat History Mus, U Kingdom					*
PB 124	LdM1	recent *H. sapiens*	Nat History Mus, U Kingdom					*
PB 1255	LdM2	recent *H. sapiens*	Nat History Mus, U Kingdom					*
PB 1255	LdM1	recent *H. sapiens*	Nat History Mus, U Kingdom					*
PB 149	LdM1	recent *H. sapiens*	Nat History Mus, U Kingdom					*
PB 227	LdM2	recent *H. sapiens*	Nat History Mus, U Kingdom					*
PB 227	LdM1	recent *H. sapiens*	Nat History Mus, U Kingdom					*
PB 292	LdM2	recent *H. sapiens*	Nat History Mus, U Kingdom					*
PB 311	LdM1	recent *H. sapiens*	Nat History Mus, U Kingdom					*
PB 321	LdM1	recent *H. sapiens*	Nat History Mus, U Kingdom					*
PB 335B	LdM1	recent *H. sapiens*	Nat History Mus, U Kingdom					*
PB 379	LdM2	recent *H. sapiens*	Nat History Mus, U Kingdom					*
PB 379	LdM1	recent *H. sapiens*	Nat History Mus, U Kingdom					*
PB 414	LdM2	recent *H. sapiens*	Nat History Mus, U Kingdom					*
PB 414	LdM1	recent *H. sapiens*	Nat History Mus, U Kingdom					*
PB 419	LdM2	recent *H. sapiens*	Nat History Mus, U Kingdom					*
PB 419	LdM1	recent *H. sapiens*	Nat History Mus, U Kingdom					*
PB 422	LdM1	recent *H. sapiens*	Nat History Mus, U Kingdom					*
PB 424	LdM1	recent *H. sapiens*	Nat History Mus, U Kingdom					*
PB 452	LdM1	recent *H. sapiens*	Nat History Mus, U Kingdom					*
PB 473	LdM1	recent *H. sapiens*	Nat History Mus, U Kingdom					*
PB 492	LdM2	recent *H. sapiens*	Nat History Mus, U Kingdom					*
PB 492	LdM1	recent *H. sapiens*	Nat History Mus, U Kingdom					*
PB 51	LdM2	recent *H. sapiens*	Nat History Mus, U Kingdom					*
PB 544	LdM1	recent *H. sapiens*	Nat History Mus, U Kingdom					*
PB 601	LdM2	recent *H. sapiens*	Nat History Mus, U Kingdom					*
PB 623	LdM1	recent *H. sapiens*	Nat History Mus, U Kingdom					*
PB 637	LdM2	recent *H. sapiens*	Nat History Mus, U Kingdom					*
PB 649	LdM1	recent *H. sapiens*	Nat History Mus, U Kingdom					*
PB 674	LdM2	recent *H. sapiens*	Nat History Mus, U Kingdom					*
PB 833	LdM2	recent *H. sapiens*	Nat History Mus, U Kingdom					*
PB 833	LdM1	recent *H. sapiens*	Nat History Mus, U Kingdom					*
PB 868	LdM2	recent *H. sapiens*	Nat History Mus, U Kingdom					*
PB 891	LdM2	recent *H. sapiens*	Nat History Mus, U Kingdom					*
PB 891	LdM1	recent *H. sapiens*	Nat History Mus, U Kingdom					*
PB 919	LdM1	recent *H. sapiens*	Nat History Mus, U Kingdom					*
PB1132	LdM2	recent *H. sapiens*	Nat History Mus, U Kingdom					*
PB149	LdM2	recent *H. sapiens*	Nat History Mus, U Kingdom					*
PB321	LdM2	recent *H. sapiens*	Nat History Mus, U Kingdom					*
PB452	LdM2	recent *H. sapiens*	Nat History Mus, U Kingdom					*
PB544	LdM2	recent *H. sapiens*	Nat History Mus, U Kingdom					*
PB636	LdM2	recent *H. sapiens*	Nat History Mus, U Kingdom					*
PB919	LdM2	recent *H. sapiens*	Nat History Mus, U Kingdom					*
PB936	LdM2	recent *H. sapiens*	Nat History Mus, U Kingdom					*

PB940	LdM2	recent *H. sapiens*	Nat History Mus, U Kingdom					*
SI 12	LM1	recent *H. sapiens*	NMNH, Smithsonian, USA	*		*		
SI 13	LM1	recent *H. sapiens*	NMNH, Smithsonian, USA	*		*		
SI 14	LM3	recent *H. sapiens*	NMNH, Smithsonian, USA	*	*			
SI 15	LM1	recent *H. sapiens*	NMNH, Smithsonian, USA	*		*		
SI 34	LM1	recent *H. sapiens*	NMNH, Smithsonian, USA	*		*		
SI 36	LM1	recent *H. sapiens*	NMNH, Smithsonian, USA	*		*		
SI 37	LM1	recent *H. sapiens*	NMNH, Smithsonian, USA	*		*		
SI 38	LM1	recent *H. sapiens*	NMNH, Smithsonian, USA	*		*		
SI 40	LM1	recent *H. sapiens*	NMNH, Smithsonian, USA	*		*		
SI 42	LM1	recent *H. sapiens*	NMNH, Smithsonian, USA	*		*		
SI 44	LM1	recent *H. sapiens*	NMNH, Smithsonian, USA	*		*		
SI 45	LM1	recent *H. sapiens*	NMNH, Smithsonian, USA	*		*		
SI 46	LM1	recent *H. sapiens*	NMNH, Smithsonian, USA	*		*		
SI 47	LM1	recent *H. sapiens*	NMNH, Smithsonian, USA	*		*		
SI 48	LM1	recent *H. sapiens*	NMNH, Smithsonian, USA	*		*		*
Oberkassel D999	UP4	recent *H. sapiens*	Oberkassel, Germany		*			
Oberkassel D999	UI2	recent *H. sapiens*	Oberkassel, Germany				*	
Oberkassel D999	LI2	recent *H. sapiens*	Oberkassel, Germany				*	
Oberkassel D999	LC	recent *H. sapiens*	Oberkassel, Germany				*	
Ohalo II H1	LI2	recent *H. sapiens*	Ohalo, Israel				*	
Ohalo II H1	LC	recent *H. sapiens*	Ohalo, Israel				*	
Ohalo II H2	UI1	recent *H. sapiens*	Ohalo, Israel				*	
Ohalo II H2	LI2	recent *H. sapiens*	Ohalo, Israel				*	
Ohalo II H2	LC	recent *H. sapiens*	Ohalo, Israel				*	
190-23-011_BR_45	LI2	recent *H. sapiens*	Paul Brown Clinical, USA				*	
219-07-004_BR_50	UI2	recent *H. sapiens*	Paul Brown Clinical, USA				*	
220-23-014_BR_50	LI2	recent *H. sapiens*	Paul Brown Clinical, USA				*	
221-09-010_BR_50	UI1	recent *H. sapiens*	Paul Brown Clinical, USA				*	
26-004_BR_42	LI2	recent *H. sapiens*	Paul Brown Clinical, USA				*	
271-26-011_BR_68	LI2	recent *H. sapiens*	Paul Brown Clinical, USA				*	
278-07-005_BR_64	UI2	recent *H. sapiens*	Paul Brown Clinical, USA				*	
280-23-016_BR_64	LI2	recent *H. sapiens*	Paul Brown Clinical, USA				*	
313-23-019_BR_77	LI2	recent *H. sapiens*	Paul Brown Clinical, USA				*	
362-26-015_BR_100	LI2	recent *H. sapiens*	Paul Brown Clinical, USA				*	
363-07-010_BR_100	UI2	recent *H. sapiens*	Paul Brown Clinical, USA				*	
Pavlov 2-1	UP4	recent *H. sapiens*	Pavlov, Czech Republic		*			
Belgian 129a	LM1	recent *H. sapiens*	R Belgian Inst Nat Sci, Belgium	*		*		
Belgian 13e	LM2	recent *H. sapiens*	R Belgian Inst Nat Sci, Belgium	*				
Belgian 89a	LM1	recent *H. sapiens*	R Belgian Inst Nat Sci, Belgium	*		*		
Belgian 93a	LM1	recent *H. sapiens*	R Belgian Inst Nat Sci, Belgium	*		*		
Belgian A31	LM1	recent *H. sapiens*	R Belgian Inst Nat Sci, Belgium	*		*		
Belgian A32	LM1	recent *H. sapiens*	R Belgian Inst Nat Sci, Belgium	*		*		
Solutre	LdM1	recent *H. sapiens*	Solutre, France					*
Solutre	LdM2	recent *H. sapiens*	Solutre, France					*
St Germain B5	LdM2	recent *H. sapiens*	St.-Germaine-en-Laye, France					*
St Germaine B3	LdM2	recent *H. sapiens*	St.-Germaine-en-Laye, France					*
Sunghir 3	LdM2	recent *H. sapiens*	Sunghir, Russia					*
Mandible 9726	LI2	recent *H. sapiens*	U Leipzig Anat Coll, Germany				*	
Mandible 9726	LC	recent *H. sapiens*	U Leipzig Anat Coll, Germany				*	
ULAC 001	LM1	recent *H. sapiens*	U Leipzig Anat Coll, Germany	*		*		
ULAC 001	LM2	recent *H. sapiens*	U Leipzig Anat Coll, Germany	*				
ULAC 001	LM3	recent *H. sapiens*	U Leipzig Anat Coll, Germany	*				
ULAC 001	LP3	recent *H. sapiens*	U Leipzig Anat Coll, Germany	*				
ULAC 001	LP4	recent *H. sapiens*	U Leipzig Anat Coll, Germany	*	*			
ULAC 001	LI2	recent *H. sapiens*	U Leipzig Anat Coll, Germany				*	
ULAC 001	LC	recent *H. sapiens*	U Leipzig Anat Coll, Germany				*	
ULAC 013	LI2	recent *H. sapiens*	U Leipzig Anat Coll, Germany				*	
ULAC 013	LC	recent *H. sapiens*	U Leipzig Anat Coll, Germany				*	
ULAC 013	LM1	recent *H. sapiens*	U Leipzig Anat Coll, Germany	*		*		
ULAC 013	LM2	recent *H. sapiens*	U Leipzig Anat Coll, Germany	*				
ULAC 013	LM3	recent *H. sapiens*	U Leipzig Anat Coll, Germany	*				
ULAC 013	LP3	recent *H. sapiens*	U Leipzig Anat Coll, Germany	*				
ULAC 013	LP4	recent *H. sapiens*	U Leipzig Anat Coll, Germany	*	*			

ULAC 058	LI2	recent *H. sapiens*	U Leipzig Anat Coll, Germany				*	
ULAC 058	LC	recent *H. sapiens*	U Leipzig Anat Coll, Germany				*	
ULAC 058	LM1	recent *H. sapiens*	U Leipzig Anat Coll, Germany	*		*		
ULAC 058	LM2	recent *H. sapiens*	U Leipzig Anat Coll, Germany	*				
ULAC 058	LM3	recent *H. sapiens*	U Leipzig Anat Coll, Germany	*	*			
ULAC 058	LP3	recent *H. sapiens*	U Leipzig Anat Coll, Germany	*				
ULAC 058	LP4	recent *H. sapiens*	U Leipzig Anat Coll, Germany	*	*			
ULAC 066	LI2	recent *H. sapiens*	U Leipzig Anat Coll, Germany				*	
ULAC 066	LC	recent *H. sapiens*	U Leipzig Anat Coll, Germany				*	
ULAC 074	LI2	recent *H. sapiens*	U Leipzig Anat Coll, Germany				*	
ULAC 074	LC	recent *H. sapiens*	U Leipzig Anat Coll, Germany				*	
ULAC 081	LdM1	recent *H. sapiens*	U Leipzig Anat Coll, Germany	*				
ULAC 081	LdM2	recent *H. sapiens*	U Leipzig Anat Coll, Germany	*				
ULAC 101	LdM2	recent *H. sapiens*	U Leipzig Anat Coll, Germany	*				
ULAC 119	LI2	recent *H. sapiens*	U Leipzig Anat Coll, Germany				*	
ULAC 119	LC	recent *H. sapiens*	U Leipzig Anat Coll, Germany				*	
ULAC 140	LdM2	recent *H. sapiens*	U Leipzig Anat Coll, Germany	*				
ULAC 151	LI2	recent *H. sapiens*	U Leipzig Anat Coll, Germany				*	
ULAC 151	LC	recent *H. sapiens*	U Leipzig Anat Coll, Germany				*	
ULAC 171	LI2	recent *H. sapiens*	U Leipzig Anat Coll, Germany				*	
ULAC 171	LC	recent *H. sapiens*	U Leipzig Anat Coll, Germany				*	
ULAC 171	LP4	recent *H. sapiens*	U Leipzig Anat Coll, Germany		*			
ULAC 179	LI2	recent *H. sapiens*	U Leipzig Anat Coll, Germany				*	
ULAC 179	LC	recent *H. sapiens*	U Leipzig Anat Coll, Germany				*	
ULAC 179	LM1	recent *H. sapiens*	U Leipzig Anat Coll, Germany	*				
ULAC 179	LM2	recent *H. sapiens*	U Leipzig Anat Coll, Germany	*				
ULAC 179	LM3	recent *H. sapiens*	U Leipzig Anat Coll, Germany	*	*			
ULAC 179	LP3	recent *H. sapiens*	U Leipzig Anat Coll, Germany	*				
ULAC 179	LP4	recent *H. sapiens*	U Leipzig Anat Coll, Germany	*	*			
ULAC 259	LI2	recent *H. sapiens*	U Leipzig Anat Coll, Germany				*	
ULAC 259	LC	recent *H. sapiens*	U Leipzig Anat Coll, Germany				*	
ULAC 447	LdM2	recent *H. sapiens*	U Leipzig Anat Coll, Germany	*				
ULAC 477	LdM1	recent *H. sapiens*	U Leipzig Anat Coll, Germany	*				
ULAC 522	LI2	recent *H. sapiens*	U Leipzig Anat Coll, Germany				*	
ULAC 522	LC	recent *H. sapiens*	U Leipzig Anat Coll, Germany				*	
ULAC 536	LI2	recent *H. sapiens*	U Leipzig Anat Coll, Germany				*	
ULAC 536	LC	recent *H. sapiens*	U Leipzig Anat Coll, Germany				*	
ULAC 536	LM1	recent *H. sapiens*	U Leipzig Anat Coll, Germany	*				
ULAC 536	LM2	recent *H. sapiens*	U Leipzig Anat Coll, Germany	*				
ULAC 536	LM3	recent *H. sapiens*	U Leipzig Anat Coll, Germany	*	*			
ULAC 536	LP3	recent *H. sapiens*	U Leipzig Anat Coll, Germany	*				
ULAC 536	LP4	recent *H. sapiens*	U Leipzig Anat Coll, Germany	*	*			
ULAC 566	LI2	recent *H. sapiens*	U Leipzig Anat Coll, Germany				*	
ULAC 566	LC	recent *H. sapiens*	U Leipzig Anat Coll, Germany				*	
ULAC 607	LP4	recent *H. sapiens*	U Leipzig Anat Coll, Germany		*			
ULAC 607	LI2	recent *H. sapiens*	U Leipzig Anat Coll, Germany				*	
ULAC 607	LC	recent *H. sapiens*	U Leipzig Anat Coll, Germany				*	
ULAC 659	LI2	recent *H. sapiens*	U Leipzig Anat Coll, Germany				*	
ULAC 659	LC	recent *H. sapiens*	U Leipzig Anat Coll, Germany				*	
ULAC 742	LdM1	recent *H. sapiens*	U Leipzig Anat Coll, Germany	*				
ULAC 742	LdM2	recent *H. sapiens*	U Leipzig Anat Coll, Germany	*				
ULAC 752	LI2	recent *H. sapiens*	U Leipzig Anat Coll, Germany				*	
ULAC 752	LC	recent *H. sapiens*	U Leipzig Anat Coll, Germany				*	
ULAC 790	LM1	recent *H. sapiens*	U Leipzig Anat Coll, Germany	*				
ULAC 790	LM2	recent *H. sapiens*	U Leipzig Anat Coll, Germany	*				
ULAC 790	LM3	recent *H. sapiens*	U Leipzig Anat Coll, Germany	*	*			
ULAC 790	LP3	recent *H. sapiens*	U Leipzig Anat Coll, Germany	*				
ULAC 790	LP4	recent *H. sapiens*	U Leipzig Anat Coll, Germany	*	*			
ULAC 790	LI2	recent *H. sapiens*	U Leipzig Anat Coll, Germany				*	
ULAC 790	LC	recent *H. sapiens*	U Leipzig Anat Coll, Germany				*	
ULAC 797	LM1	recent *H. sapiens*	U Leipzig Anat Coll, Germany	*		*		
ULAC 797	LM2	recent *H. sapiens*	U Leipzig Anat Coll, Germany	*				
ULAC 797	LM3	recent *H. sapiens*	U Leipzig Anat Coll, Germany	*				
ULAC 797	LI2	recent *H. sapiens*	U Leipzig Anat Coll, Germany				*	

ULAC 797	LC	recent *H. sapiens*	U Leipzig Anat Coll, Germany				*	
ULAC 797	LP3	recent *H. sapiens*	U Leipzig Anat Coll, Germany	*				
ULAC 797	LP4	recent *H. sapiens*	U Leipzig Anat Coll, Germany	*	*			
ULAC 799-27	LI2	recent *H. sapiens*	U Leipzig Anat Coll, Germany				*	
ULAC 799-27	LC	recent *H. sapiens*	U Leipzig Anat Coll, Germany				*	
ULAC 799-28	LI2	recent *H. sapiens*	U Leipzig Anat Coll, Germany				*	
ULAC 799-28	LC	recent *H. sapiens*	U Leipzig Anat Coll, Germany				*	
ULAC 801	LI2	recent *H. sapiens*	U Leipzig Anat Coll, Germany				*	
ULAC 801	LC	recent *H. sapiens*	U Leipzig Anat Coll, Germany				*	
ULAC 806	LI2	recent *H. sapiens*	U Leipzig Anat Coll, Germany				*	
ULAC 806	LC	recent *H. sapiens*	U Leipzig Anat Coll, Germany				*	
ULAC 864	LdM2	recent *H. sapiens*	U Leipzig Anat Coll, Germany	*				
ULAC 966	LdM2	recent *H. sapiens*	U Leipzig Anat Coll, Germany	*				
ULAC 982	LdM1	recent *H. sapiens*	U Leipzig Anat Coll, Germany	*				
ULAC 982	LdM2	recent *H. sapiens*	U Leipzig Anat Coll, Germany	*				
ULAC 997	LdM2	recent *H. sapiens*	U Leipzig Anat Coll, Germany	*				
RN_XX_S_Tb_10	LdM1	recent *H. sapiens*	University of Bologna, Italy	*				
RN_XX_T_03	LdM1	recent *H. sapiens*	University of Bologna, Italy	*				
ROEEAPEZALIO_US_26	LdM1	recent *H. sapiens*	University of Bologna, Italy	*				
SM_BT_TB_6	LdM1	recent *H. sapiens*	University of Bologna, Italy	*				
SM_BT_US_22	LdM1	recent *H. sapiens*	University of Bologna, Italy	*				
SAF 137	LdM1	recent *H. sapiens*	Uni of Capetown, South Africa					*
SAF 150	LdM1	recent *H. sapiens*	Uni of Capetown, South Africa					*
SAF 151	LdM1	recent *H. sapiens*	Uni of Capetown, South Africa					*
SAF 190	LdM1	recent *H. sapiens*	Uni of Capetown, South Africa					*
SAF 193	LdM1	recent *H. sapiens*	Uni of Capetown, South Africa					*
SAF 195	LdM1	recent *H. sapiens*	Uni of Capetown, South Africa					*
SAF 197	LdM1	recent *H. sapiens*	Uni of Capetown, South Africa					*
SAF 208	LdM2	recent *H. sapiens*	Uni of Capetown, South Africa					*
SAF 208	LdM1	recent *H. sapiens*	Uni of Capetown, South Africa					*
SAF 231	LdM2	recent *H. sapiens*	Uni of Capetown, South Africa					*
SAF 231	LdM1	recent *H. sapiens*	Uni of Capetown, South Africa					*
SAF 234	LdM2	recent *H. sapiens*	Uni of Capetown, South Africa					*
SAF 234	LdM1	recent *H. sapiens*	Uni of Capetown, South Africa					*
SAF 239	LdM1	recent *H. sapiens*	Uni of Capetown, South Africa					*
SAF 330	LdM2	recent *H. sapiens*	Uni of Capetown, South Africa					*
SAF 330	LdM1	recent *H. sapiens*	Uni of Capetown, South Africa					*
SAF 346	LdM1	recent *H. sapiens*	Uni of Capetown, South Africa					*
SAF 357	LdM1	recent *H. sapiens*	Uni of Capetown, South Africa					*
SAF 381	LdM2	recent *H. sapiens*	Uni of Capetown, South Africa					*
SAF 381	LdM1	recent *H. sapiens*	Uni of Capetown, South Africa					*
SAF 437	LdM1	recent *H. sapiens*	Uni of Capetown, South Africa					*
SAF 468	LdM2	recent *H. sapiens*	Uni of Capetown, South Africa					*
SAF 468	LdM1	recent *H. sapiens*	Uni of Capetown, South Africa					*
SAF 471	LdM2	recent *H. sapiens*	Uni of Capetown, South Africa					*
SAF 471	LdM1	recent *H. sapiens*	Uni of Capetown, South Africa					*
SAF 51	LdM1	recent *H. sapiens*	Uni of Capetown, South Africa					*
SAF 95	LdM1	recent *H. sapiens*	Uni of Capetown, South Africa					*
SAF UK2	LdM1	recent *H. sapiens*	Uni of Capetown, South Africa					*
IT 1105	LdM1	recent *H. sapiens*	University of Siena, Italy					*
IT Italy 1	LdM1	recent *H. sapiens*	University of Siena, Italy					*
IT Italy 2	LdM1	recent *H. sapiens*	University of Siena, Italy					*
SAF A1235	LdM1	recent *H. sapiens*	Uni of Witwatersrand, S Africa					*
SAF A1244	LdM2	recent *H. sapiens*	Uni of Witwatersrand, S Africa					*
SAF A1244	LdM1	recent *H. sapiens*	Uni of Witwatersrand, S Africa					*
SAF A1247	LdM1	recent *H. sapiens*	Uni of Witwatersrand, S Africa					*
SAF A1320	LdM1	recent *H. sapiens*	Uni of Witwatersrand, S Africa					*
SAF A1327	LdM1	recent *H. sapiens*	Uni of Witwatersrand, S Africa					*
SAF A1439	LdM2	recent *H. sapiens*	Uni of Witwatersrand, S Africa					*
SAF A1439	LdM1	recent *H. sapiens*	Uni of Witwatersrand, S Africa					*
SAF A1469	LdM1	recent *H. sapiens*	Uni of Witwatersrand, S Africa					*
SAF A1655	LdM2	recent *H. sapiens*	Uni of Witwatersrand, S Africa					*
SAF A1655	LdM1	recent *H. sapiens*	Uni of Witwatersrand, S Africa					*
SAF A2819	LdM1	recent *H. sapiens*	Uni of Witwatersrand, S Africa					*

SAF A3018	LdM1	recent *H. sapiens*	Uni of Witwatersrand, S Africa					*
SAF A3019	LdM1	recent *H. sapiens*	Uni of Witwatersrand, S Africa					*
SAF A3025	LdM1	recent *H. sapiens*	Uni of Witwatersrand, S Africa					*
SAF A3148	LdM2	recent *H. sapiens*	Uni of Witwatersrand, S Africa					*
SAF A656	LdM1	recent *H. sapiens*	Uni of Witwatersrand, S Africa					*
SAF A667	LdM1	recent *H. sapiens*	Uni of Witwatersrand, S Africa					*
SAF A854	LdM1	recent *H. sapiens*	Uni of Witwatersrand, S Africa					*
Be Tb 75	LdM1	recent *H. sapiens*	Valdaro, Italy	*				
Tb 57	LdM1	recent *H. sapiens*	Valdaro, Italy	*				
Tb 76	LdM1	recent *H. sapiens*	Valdaro, Italy	*				

Supplementary Table 23: Vertebral Transverse process orientation

Asier Gómez-Olivencia

Transverse process orientation[a] (in º) of the ThI-GH thoracic vertebrae compared to other fossil and to a recent male modern sample[b].

Level and side		ThI-GH	KNM-WT 15000	Neandertals[c]			Recent European males[c]		
				Kebara 2	La Chapelle-aux-Saints 1	Regourdou 1	Right	Left	*n*
							Mean ± SD (min-max)	Mean ± SD (min-max)	
T1	right/left	70.3/70.6	**62.4***/**65.6***	78.3/74.6	-/72.1		78.0 ± 6.5 (67.9-92.5)	77.9 ± 5.6 (66.2-88.1)	28
	Total	140.9	**128.0***	152.9			155.9 ± 11.2 (138.1-174.9)		28
T2	right/left	-/59.3	**53.3***/61.0	**52.9***/(59.1)	(66.0)/(57.9)	58.3/58.9	64.0 ± 4.5 (54.1-72.9)	64.3 ± 5.1 (52.8-75.4)	30
	Total		114.3	112.0	(123.9)	117.2	128.3 ± 8.8 (107.6-147.9)		30

[a]The right/left values indicate the orientation from the mid-sagittal plane in cranial view, while the total value indicates the orientation of the transverse process to one another. Larger values (as in T1) indicate more laterally oriented transverse processes, while smaller values indicate more dorsally oriented transverse processes.
[b]Including the results of the z-score test between each fossil individual and our modern human male sample
For the z-score test, the values indicated by an asterisk are significantly different from the modern male comparative sample (* = $p < 0.05$; ** = $p < 0.01$). Values underlined are outside the range of the modern human comparative sample. Values in parentheses are estimated.
[c]Data from[118].

Supplementary Table 24: Vertebral foramen cross-sectional area

Asier Gómez-Olivencia

Absolute vertebral foramen cross-sectional area (mm²) of the ThI-GH fossils, compared to other fossil hominins and to recent *Homo sapiens* and *Pan*. In[123] recent *H. sapiens* have an age range of 12-50 years. In[117] recent *H. sapiens* are only males

Level	ThI-GH	*A. sediba* (MH1)	Dmanisi	KNM-WT 15000	*Homo antecessor*	Sima de los Huesos	*H. sapiens*	*Pan*
C2	245					315 ± 14.8 n = 3[124]	302 ± 47.5 n = 30[124]	
C3	191		187[123]			234 (VC10)[123]	224 ± 23.2 n = 62[123]	185** ± 25.3 n = 18[123]
C6	174							
C7	172	125*[123]		135*[123]	185 (ATD6-75)[123]		212 ± 26.5 n = 37 [123]	139** ± 20.9 n = 13[123]
T1	137			137[117]			193 ± 33.1 n = 25[123]	114** ± 17.2 n = 10[123]
T2	119	95*[123]		103*[123]			167 ± 26.3 n = 52[123]	92** ± 14.9 n = 19[123]

*Significantly different from recent *H. sapiens* $p < 0.05$.
**Significantly different from recent *H. sapiens* $p < 0.01$.

Supplementary Table 25: Comparative overview of key anatomical features in the ThI-GH hominins. Frequency : Low ≥ 0 up to 33.3%, Moderate = 34%-66.7%, High ≥ 66.7%

	H. erectus	***H. antecessor***	**Neanderthal**	**Early *H. sapiens***	**ThI-GH**
Anterior dentition (Incisors and Canines)	Pronounced shovel shape, labial convexity	Pronounced shovel shape, labial convexity	Pronounced shovel shape, labial convexity, lingual tubercle, marginal ridges	No marked lingual reliefs	No marked lingual reliefs
P_4 Asymmetry	Present (moderate)	Present (high)	Present (high)	Present (low)	Absent
P_4 transverse crest	Uninterrupted (moderate)	Uninterrupte d (high)	Uninterrupted (high)	Uninterrupted (low)	Uninterrupted
Cusp 6 LM_1	Present (low)	Absent	Present (moderate)	Absent	Absent
Protostylid LM_1	Present (moderate)	Absent	Absent	Present (low)	Present
Cusp 6 LM_2	Present (moderate)	Absent	Present (moderate)	Present (low)	Absent
Cusp 7 LM_2	Present (moderate)	Present (moderate)	Present (low)	Present (low)	Absent
Protostylid LM_2	Present (low)	Absent	Present (low)	Present (low)	Absent
Cusp 6 LM_3	Present (moderate)	Absent	Present (moderate)	Present (moderate)	Absent
Protostylid LM_3	Present (moderate)	Absent	Present (low)	Present (low)	Present
P_4 dentine body height EDJ)	Short	Short	Tall	Intermediate	Intermediate
DM_2 mesial marginal ridge EDJ)	-	Intermediate	Intermediate	Variable	Elongated
dM_2 distal marginal ridge (EDJ)	-	Elongated	Intermediate	Variable	Short
dM_2 marginal ridges (EDJ)	-	Narrow and elongated	Rounded	Rounded	Narrow
M_1 marginal ridges (EDJ)	Narrow and elongated	Narrow and elongated	Rounded	Rounded	Narrow and elongated
M_3 marginal ridges (EDJ)	Mesiodistally elongated	Mesiodistally elongated	Mesiodistally short	Mesiodistally short	Mesiodistally elongated
dm_1 shape outline	-	Trapezoidal	Rectangular	Rectangular	Rectangular
dm_1 mesio-buccal cusp	-	Very large	Discrete large	Discrete large	Discrete large
dm_2 shape outline	Rectangular	Rectangular	Oval	Rectangular/ slight oval	Rectangular
dm_2 bucco distal outline	Reduced	Enlarged	Enlarged	Reduced	Reduced
dm_2 lingual outline	Straight	Straight	Convex	Straight	Straight
Anterior tooth (Incisors and Canines) root size	Large length, volume and surface	Moderate length	Large length, volume and surface	Moderate length, surface and volume.	Intermediate to large length volume and surface.

Anterior tooth (Incisors and Canines) root shape	Moderate root labial convexity	?	Marked root labial convexity	Slight root labial convexity	Slight root labial convexity
M_2 root form	Hypo to meso-taurodont	?	Meso- to hyper-taurodont	Hypo- to mesotaurodont	Hypotaurodont (GH1) to mesotaurodont (GH-10717)
M_3 root form	Hypotaurodont	?	Meso- to hypertaurodont	Mesotaurodont	Hypotaurodont (GH1) to mesotaurodont (GH-10717)
M_{1-3} root volume	$M_1 < M_2 > M_3$	?	$M_1 < M_2 >> M_3$	$M_1 < M_2 >> M_3$	$M_1 < M_2 >> M_3$
Position of mental foramen	P3-P4, P4-M1	P3-P4	M1	P3-P4, P4, M1	P3-P4
Anterior marginal tubercle	Absent, Present	Absent	Absent, Present	Absent, Present	Present (GH-10717) Absent (GH1)
Position of the lateral prominence	M2, M2-M3, M3	M2	M3	M2, M2-3, M3	M2 (GH-10717) M2-M3 (GH1)
M3 in relation to ramus	Covered, Partially covered, Uncovered	Partially covered	Uncovered	Partially covered, Uncovered	Partially covered (GH-10717) Uncovered (GH1)
Retromolar area	Vertical, Inclined	Inclined	Horizontal	Horizontal, Inclined	Inclined
Mylohyoid line in relation to alveolar margin at M3	Low, Intermediate	Low	Low, Intermediate, High	Low, Intermediate, High	Low (GH-10717) Intermediate (GH1)
Mylohyoid line in relation to alveolar margin	Parallel, Inclined	Parallel	Inclined, Diagonal	Parallel, Inclined	Parallel (GH-10717) Inclined (GH1)
Masseteric fossa	Deep, Shallow, Flat	Shallow	Shallow, Flat	Flat, Shallow	Shallow (GH10717) Deep (GH1)
Pterygoid fossa	Shallow	Shallow	Deep	Shallow, Deep	Shallow
Gonion profile	Expanded, Regular	Regular	Truncated	Regular	Regular
Orientation of the C7 lower articular facets	Ventro-laterally oriented	Ventrally oriented	Ventrally oriented	Ventrally oriented	Ventro-laterally oriented
C7 relative canal area	Small	Normal	?	Normal	Normal
T1 transverse processes	Slightly more dorsally oriented*	?	Laterally oriented	Laterally oriented	Significantly more dorsally oriented*
T2 relative canal area	Small	?	?	Normal	Small

*Modern *Homo sapiens* used as a baseline here.

Supplementary References

1. Lefèvre, D. & Raynal, J.-P. Les formations plio-pléistocènes de Casablanca et la chronostratigraphie du Quaternaire marin du Maroc revisitées / The Plio-Pleistocene formations of Casablanca and the marine Quaternary chronostratigraphy of Morocco revisited. *Quaternaire* **13**, 9-21 (2002).
2. Lefèvre, D. *et al.* Enregistrements-réponses des variations climatiques du Pléistocène supérieur et de l'Holocène sur le littoral de Casablanca (Maroc). *Quaternaire* **5**, 173-180 (1994).
3. Lefèvre, D. *et al.* Les paléolittoraux plio-pléistocènes de Casablanca, cadre chronostratigraphique et paléogéographique de la Préhistoire ancienne du Maroc atlantique. *Bull. Archéol. Maroc* **21**, 39-70 (2021).
4. Texier, J.-P. *et al.* Contribution pour un nouveau cadre stratigraphique des formations littorales du Quaternaire de la région de Casablanca (Maroc). *C. R. Acad. Sc. Paris.* **318,** séries II, 1247-1253 (1994).
5. Texier, J.-P. *et al.* Lithostratigraphy of the littoral deposits of the last one million years in the Casablanca region (Maroc). *Quaternaire* **13**, 23-41 (2002).
6. Biberson, P. *Le cadre paléogéographique de la Préhistoire du Maroc atlantique*. Publications du Service des Antiquités du Maroc **16**, Rabat (1961).
7. Lefèvre, D., *et al.* in *Préhistoire de Casablanca. I - La grotte des Rhinocéros (fouilles 1991 et 1996)* (eds J.-P. Raynal & A. Mohib) 45-59 (Institut National des Sciences de l'Archéologie et du Patrimoine, VESAM **VI**, 2016).
8. Chabli, A. *et al.* Déformations néotectoniques dans les dépôts plio-quaternaires de la région de Casablanca-Mohammedia (Meseta côtière, Maroc). *Rev. Soc. Geol. Esp.* **18**, 169-178 (2005).
9. Raynal, J.-P. *et al.* in *Handbook of Pleistocene Archaeology of Africa: Hominin behavior, geography, and chronology* (eds A. Beyin, D. K. Wright, J. Wilkins & D. I. Olszewski) 841-861 (Springer International Publishing, 2023).
10. Gallotti, R. *et al.* First high resolution chronostratigraphy for the early North African Acheulean at Casablanca (Morocco). *Sci. Rep.* **11**, 15340 (2021). https://doi.org/10.1038/s41598-021-94695-3
11. Gallotti, R., *et al.* Early North African Acheulean techno-economic systems at Thomas Quarry I - L1 (Casablanca, Morocco). *J. of Anthropol. Sc.* **101**, 1-59 (2023). https://doi 10.4436/JASS.10015.
12. Geraads, D. Biochronologie mammalienne du Quaternaire du Maroc atlantique, dans son cadre régional. *L'Anthropologie* **114**, 324-340 (2010).
13. Geraads, D., Raynal, J.-P. & Sbihi-Alaoui, F.-Z. Mammalian faunas from the Pliocene and Pleistocene of Casablanca (Morocco). *Hist. Biol.* **22**, 275-285 (2010).
14. Geraads, D. *et al.* in *African Paleoecology and Human Evolution* (eds S. Reynolds & R. Bobe) 481-491, Cambridge University Press (2022).
15. Rhodes, E. J. *et al.* New age estimates for the Palaeolithic assemblages and Pleistocene succession of Casablanca, Morocco. *Quat. Sci. Rev.* **25**, 2569-2585 (2006).
16. Raynal, J.-P. *et al.* Hominid Cave at Thomas Quarry I (Casablanca, Morocco): Recent findings and their context. *Quat. Int.* **223-224**, 369-382 (2010).
17. Raynal, J. P. *et al.* Contextes et âge des nouveaux restes dentaires humains du Pléistocène moyen de la carrière Thomas I à Casablanca (Maroc). *Bull. Soc. Préhist. Franç.* **108**, 645-669 (2011).
18. Daujeard, C. *et al.* Pleistocene Hominins as a Resource for Carnivores: A c. 500,000-Year-Old Human Femur Bearing Tooth-Marks in North Africa (Thomas Quarry I,

Morocco). *PLoS ONE* **11**, e0152284
(2016). https://doi.org/10.1371/journal.pone.0152284
19. Grün, R. & Stringer, C. Direct dating of human fossils and the ever-changing story of human evolution. *Quat. Sci. Rev.***322** (2023).
20. Geraads, D. Plio-Pleistocene mammalian biostratigraphy of Atlantic Morocco. *Quaternaire* **13,** 1, 43-53 (2002).
21. Dearing, J. A. *Environmental magnetic susceptibility using the Bartington MS2 system* (Chi Publishing, Kenilworth, 1994).
22. Sitzia, L. *et al.* Dynamics and sources of last glacial aeolian deposition in southwest France derived from dune patterns, grain-size gradients and geochemistry, and reconstruction of efficient wind directions. *Quat. Sc. Rev.* **170**, 250–268 (2017).
23. Sitzia, L. *et al.* A perched, high-elevation wetland complex in the Atacama Desert (northern Chile) and its implications for past human settlement. *Quat. Res.* **92**, 33-52 (2019).
24. Geraads, D. La faune des sites à *Homo erectus* des carrières Thomas (Casablanca, Maroc). *Quaternaria* **22**, 65–94 (1980).
25. Geraads, D. *et al.* in *African Paleoecology and Human Evolution* (eds S. Reynolds & R. Bobe) 475–480 (Cambridge University Press, 2022).
26. Pickford, M. The fossil Suidae (Mammalia, Artiodactyla) from Ternifine (Tighenif) Algeria. *Münch. Geowiss. Abh. A: Geol. Paläontol.* **50**, 1–67 (2020).
27. Daujeard, C., Geraads, D., Gallotti, R., Mohib, A. & Raynal, J.-P. Carcass acquisition and consumption by carnivores and hominins in Middle Pleistocene sites of Casablanca (Morocco). *J. Taphon.* **10**, 349–372 (2012).
28. Abellán, N. *et al.* 2021. Deep learning classification of tooth scores made by different carnivores: achieving high accuracy when comparing African carnivore taxa and testing the hominin shift in the balance of power. *Archaeol. Anthropol. Sci.* **13**(2), 1–14 (2021).
29. Jaeger, J.-J. in *After the Australopithecines* (eds K.W. Butzer & G. Ll. Isaac) 399–410 (Mouton, 1975).
30. Jaeger, J.-J. Origine et évolution du genre *Ellobius* (Mammalia, Rodentia) en Afrique Nord-Occidentale. *Folia Quat* **57**, 3–50 (1988).
31. Geraads, D. Rongeurs et Lagomorphes du Pléistocène moyen de la "Grotte des Rhinocéros", carrière Oulad Hamida 1, à Casablanca, Maroc. *N. Jhrb. Geol. Paläont. Mon.* **191**, 147–172 (1994).
32. Tong, H. The Gerbillinae (Rodentia) from Tighennif (Pleistocene of Algeria) and their significance. *Mod. Geol.* **10**, 197–214 (1986).
33. Tong, H. Origine et évolution des Gerbillidae (Mammalia, Rodentia) en Afrique du Nord. *Mém. Soc. géol. Fr***, NS 155**, 1–118 (1989).
34. Geraads, D. Biogeographic relationships of Pliocene and Pleistocene North-western African mammals. *Quat. Int.* **212**, 159–168 (2010).
35. Sen, S., Geraads, D., Pickford, M. & Vacant, R. 2024. Pliocene and Pleistocene lagomorphs (Mammalia) from Northwest Africa: new discoveries. *Palaeobiodiv. Palaeoenvir*. **104**, 381–417 (2024).
36. Geraads, D. Middle Pleistocene *Crocidura* (Mammalia, Insectivora) from Oulad Hamida I, Morocco, and their phylogenetic relationships. *Proc. K. Ned. Akad.van Wet.* **96**, 281–294 (1993).
37. Delson, E. & Hoffstetter, R. in *Theropithecus: the rise and fall of a primate genus* (ed Jablonski, N. G.) 191–208 (Cambridge University Press, 1993).
38. Deino, A. & McBrearty, S. 40Ar/39Ar dating of the Kapthurin Formation, Baringo, Kenya. *J. Hum. Evol.* **42**, 185–210 (2002).

39. Geraads, D. Pleistocene Carnivora (Mammalia) from Tighennif (Ternifine), Algeria. *Geobios* **49**, 445–458 (2016).
40. Lewis, M. E. & Werdelin, L. A revision of the genus *Crocuta* (Mammalia, Hyaenidae). *Palaeontogr.* A, **322**, 1–115 (2022).
41. Geraads, D. A revision of the fossil Canidae (Mammalia) of North-western Africa. *Palaeontol.* **54**, 429–446 (2011).
42. Geraads, D. Pliocene Rhinocerotidae (Mammalia) from Hadar and Dikika (Lower Awash, Ethiopia), and a revision of the origin of modern African rhinos. *J. Vertebr. Paleontol.* **25**, 451–461 (2005).
43. Harris, J. M. in *Koobi Fora Research Project* (ed J. M. Harris) 139–320 (Clarendon Press, Oxford, 1991).
44. Suwa, G., Souron, A. & Asfaw, B. Fossil Suidae of the Konso Formation. *Univ. Tokyo Mus.* **47**, 73–125 (2014).
45. Medin, T. *et al.* in *African Paleoecology and Human Evolution* (eds S. Reynolds & R. Bobe) 187–196 (Cambridge University Press, 2022).
46. Geraads D., Alemseged Z., Reed D., Wynn J. & Roman, D. C. The Pleistocene fauna (other than Primates) from Asbole, lower Awash Valley, Ethiopia, and its environmental and biochronological implications. *Geobios* **37**, 697–718 (2004).
47. Frost, S. R. & Alemseged, Z. Middle Pleistocene fossil Cercopithecidae from Asbole, Afar Region, Ethiopia. *J. Hum. Evol.* **53**, 227–259 (2007).
48. Gilbert, H., & Asfaw, B. (eds) Homo erectus - *Pleistocene evidence from the Middle Awash*. (Univ. of California Press, 2009).
49. Bishop, L.C. *et al.* in *African Paleoecology and Human Evolution* (eds S. Reynolds & R. Bobe) 360–375 (Cambridge University Press, 2022).
50. Potts, R. & Faith, J. T. in *African Paleoecology and Human Evolution* (eds S. Reynolds & R. Bobe) 376–383 (Cambridge University Press, 2022).
51. Gentry, A. W. & Gentry, A. Fossil Bovidae (Mammalia) of Olduvai Gorge, Tanzania. Part I. *Bull. Br. Mus. (Nat. Hist.) Geol.* **29**, 289–446 (1978).
52. Harris, J. M. & White, T. D. Evolution of the Plio-Pleistocene African Suidae. *Trans. Am. Philos. Soc.* **69**(2), 1–128 (1979).
53. Churcher, C. S. & Richardson M. L. in *Evolution of African Mammals* (eds Maglio, V. J. & Cooke, H. B. S) 379–422 (Harvard University Press, Cambridge, 1978).
54. Leakey, L. S. B. 1965. *Olduvai Gorge 1951–61 - A preliminary report on the geology and fauna.* (Cambridge University Press, 1965).
55. Werdelin, L. & Peigné, S. in *Cenozoic Mammals of Africa* (eds L. Werdelin & W. J. Sanders) 603–658 (University of California Press, 2010).
56. Smith, G. M., Ruebens, K., Gaudzinski-Windheuser, S. & Steele, T. E. Subsistence strategies throughout the African Middle Pleistocene: Faunal evidence for behavioral change and continuity across the Earlier to Middle Stone Age transition. *J. Hum. Evol.* **127**, 1–20 (2019).
57. De Ruiter, D. in *African Paleoecology and Human Evolution* (eds S. Reynolds & R. Bobe) 102–119 (Cambridge University Press, 2022).
58. Boëda, E. Le débitage discoïde et le débitage Levallois récurrent centripète. *Bull. Soc. Préhist. Franç.* **90-96**, 392-404 (1993).
59. Jaubert, J. & Mourre, V. Coudoulous, le Rescundudou, Mauran : diversité des matières premières et variabilité des schémas de production d'éclats in *Reduction Processes (Chaînes Opératoires) in the European Mousterian* (eds A. Bietti & S. Grimaldi), 313-341 (Quaternaria Nova 6, Istituto Italiano di Paleontologia Humana, 1996).

60. Mourre, V. in *Discoid Lithic Technology: Advances and Implications* (ed M. Peresani) 1-18 (BAR International Series 1120, 2003).
61. Terradas, X. in *Discoid Lithic Technology: Advances and Implications* (ed M. Peresani) 19-32 (BAR International Series 1120, 2003).
62. Ashton, N., Cook, J.M., Lewis, S.G. & Rose, J. (eds), *High Lodge: Excavations by G. de G. Sieveking, 1962-8 and J. Cook, 1988* (British Museum Press, 1992).
63. Forestier, H. Le Clactonien : mise en application d'une nouvelle méthode de débitage s'inscrivant dans la variabilité des systèmes de production lithique du paléolithique ancien. *Paléo* **5**, 53-82 (1993).
64. Daujeard, C., Falguères, C., Shao, Q., Geraads, D., Hublin, J.J., Lefèvre, D., El Graoui, M., Rué, M., Gallotti, R., Delvigne, V., Queffelec, A., Ben Arous, E., Tombret, O., Mohib, A. & Raynal, J.-P., 2020. Earliest African evidence of carcass processing and consumption in cave, Casablanca, Morocco. *Sc. Rep.* **10**, 4761 (2020). https://doi.org/10.1038/s41598-020-61580-4
65. Grün, R., Schwarcz, H.P. & Chadam, J.M. ESR dating of tooth enamel: Coupled correction for U-uptake and U-series disequilibrium. *Nuclear Tracks and Radiation Measurements* **14**, 237–241 (1988). https://doi.org/10.1016/1359-0189(88)90071-4
66. Bahain J.J., Yokoyama Y., Falguères C. & Sarcia M.N. ESR dating of tooth enamel: A comparison with K-Ar dating. *Quat. Sc. Rev.* **11**, 245–250 (1992).
67. Grün, R. The DATA program for the calculation of ESR age estimates on tooth enamel. *Quaternary Geochronology* **4**, 231–232 (2009).
68. Shao, Q., Bahain, J.J., Falguères, C., Dolo, J. M., & Garcia, T. A new U-uptake model for combined ESR/U-series dating of tooth enamel. *Quat. Geochr.* **10**, 406–411 (2012).
69. Ikeya, M. A model of linear uranium accumulation for ESR age of Heidelberg (Mauer) and Tautavel bones. *Jap. J. Appl. Phys.* **22**, L763–L765 (1982). https:/doi.org/10.1143/JJAP.21.L690
70. Bischoff, J.L. & Rosenbauer, R.J. Uranium series dating of human skeletal remains from the Del Mar and Sunnyvale sites, California. *Science* **213**, 1003–1005 (1981).
71. Falguères, C., Bahain, J.J. & Saleki, H. U-series and ESR dating of teeth from Acheulian and Mousterian levels at La Micoque (Dordogne, France). *J. Arch. Sc.* **24**, 537–545 (1997).
72. Bahain, J.J., Falguères, C., Laurent, M., Voinchet, P., Dolo, J.M., Antoine, P. & Tuffreau, A. ESR chronology of the Somme River Terrace system and first human settlements in Northern France. *Quat. Geochr.* **2**, 356–362 (2007).
73. Grün, R. Direct dating of human remains. *Ybk. Phys. Anthr.* **49**, 2–48 (2006).
74. Falguères, C., Bahain, J.J., Duval, M., Shao, Q., Han, F., Lebon, M., Mercier, N., Perez-Gonzalez, A., Dolo, J.M. & Garcia, T. A 300-600 ka ESR/U-series chronology of Acheulian sites in Western Europe. *Quat. Intern.* **223–224**, 293–298 (2010).
75. Bischoff, J.L., Rosenbauer, R.J., Tavoso, A. & de Lumley, H. A test of uranium-series dating of fossil tooth enamel: results from Tournal Cave, France. *Appl. Geochem.* **3**, 145-151 (1988). https://doi.org/10.1016/0883-2927(88)90002-9
76. Douville, E., Sallé, E., Frank, N., Eisele, M., Pons-Branchu, E. & Ayrault, S. Rapid and accurate U–Th dating of ancient carbonates using inductively coupled plasma-quadrupole mass spectrometry. *Chem. Geol.* **272**, 1-11 (2010).
77. Pons-Branchu, E., Hillaire-Marcel, C., Deschamps, P., Ghaleb, B. & Sinclair, D. J. Early diagenesis impact on precise U-series dating of deep-sea corals: example of a 100-200-years old Lophelia pertusa sample from the northeast Atlantic. *Geochim. et Cosmochim. Acta* **69**, 4865-4879 (2005).

78. Grün, R. & Katzenberger-Apel, O. An alpha irradiator for ESR dating. *Ancient TL*, **12**, 35–38 (1994).
79. Brennan, B.J., Rink, W.J., McGuirl, E.L., Schwarcz, H.P. & Prestwich, W.V. Beta doses in tooth enamel by ''One Group'' theory and the Rosy ESR dating software. *Rad. Meas.* **27**, 307-314 (1997).
80. Mercier, N. & Falguères, C. Field gamma dose-rate measurement with a NaI(Tl) detector: re-evaluation of the "threshold" technique. *Ancient TL*, **25**, n°1, 1-4 (2007).
81. Maxbauer, D. P., Feinberg, J. M., & Fox, D. L. MAX UnMix: A web application for unmixing magnetic coercivity distributions. *Comput. Geosci.* **95**, 140-145 (2016).
82. Roberts, A. P., Tauxe, L., Heslop, D., Zhao, X., & Jiang, Z. A critical appraisal of the "Day" diagram. *J. Geophys. Res. Solid Earth* **123**, 4, 2618-2644(2018).
83. Lurcock, P. C., & Wilson, G. S. PuffinPlot: A versatile, user-friendly program for paleomagnetic analysis. *Geochem. Geophys. Geosys.* **13**, 6 (2012).
84. Dobson, S.D. & Trinkaus, E. Cross-sectional geometry and morphology of the mandibular symphysis in Middle and Late Pleistocene Homo. *J. Hum. Evol.* **43**(1), 67-87 (2002)
85. Bermúdez de Castro, J.-M., Martinón-Torres, M., Gómez-Robles, A., Prado, L. & Sarmiento, S. Comparative analysis of the Gran Dolina-TD6 (Spain) and Tighennif (Algeria) hominin mandibles. *Bul. Mém. Soc. Anthrop. Paris* **19** (3-4), 149-167 (2007).
86. Carbonell, E., Bermúdez de Castro, J.M., Arsuaga, J. L., Allue, E., Bastir, M., Benito, A., Cáceres, I., Canals, T., Díez, J. C., van der Made, J., Mosquera, M., Ollé, A., Pérez-González, A., Rodríguez, J., Rodríguez, X. P., Rosas, A., Rosell, J., Sala, R., Vallverdú, J. & Vergés, J. M. An Early Pleistocene hominin mandible from Atapuerca-TD6, Spain. *Proc. Natl. Acad. Sci. USA* **102**(16), 5674-8 (2005).
87. Rosas, A., Occurrence of neanderthal features in mandibles from the Atapuerca-SH site. *Am. J. Phys. Anthropol.* **114**(1), 74-91 (2001).
88. Hublin, J.J., Ben-Ncer, A., Bailey, S. E., Freidline, S. E., Neubauer, S., Skinner, M. M., Bergmann, I., Le Cabec, A., Benazzi, S., Harvati, K. & Gunz, P. New fossils from Jebel Irhoud, Morocco and the pan-African origin of *Homo sapiens*. *Nature* **546**, 289-292 (2007).
89. Smith, T. M. *et al.* Dental evidence for ontogenetic differences between modern humans and Neanderthals. *Proc. Natl. Acad. Sci.* **107**, 20923–20928 (2010).
90. AlQahtani, S. J., Hector, M. P. & Liversidge, H. M. Brief communication: the London atlas of human tooth development and eruption. *Am. J. Phys. Anthropol.* **142**, 481–490 (2010).
91. Mahoney, P. Human deciduous mandibular molar incremental enamel development. *Am. J. Phys. Anthropol.* **144**, 204–214 (2011).
92. Reid, D. & Dean, M. C. Variation in modern human enamel formation times. *J. Hum. Evol.* **50**, 329–346 (2006).
93. Sasaki, C., Suzuki, K., Mishima, H. & Kozawa, Y. Age determination of the Dederiyeh 1 Neanderthal child using enamel cross-striations. In *Neanderthal Burials: Excavations of the Dederiyeh Cave, Afrin, Syria* (eds Akazawa, T. & Muhesen, S.) 263–267 (International Research Center for Japanese Studies, Kyoto, 2002).
94. Ishida, H. & Kondo, O. The skull of the Neanderthal child of burial no. 2. In *Neanderthal Burials: Excavations of the Dederiyeh Cave, Afrin, Syria* (eds Akazawa, T. & Muhesen, S.) 271–297 (International Research Center for Japanese Studies, Kyoto, 2002).

95. Dodo, Y., Kondo, O., Muhesen, S. & Akazawa, T. Anatomy of the Neandertal infant skeleton from Dederiyeh Cave, Syria. In *Neandertals and Modern Humans in Western Asia* (eds Akazawa, T., Aoki, K. & Bar-Yosef, O.) 323–338 (Springer, Boston, 2002).
96. Tillier, A.-M., Vandermeersch, B., Arensburg, B. & Chech, M. New human remains from Kebara Cave (Mount Carmel). The place of the Kebara hominids in the Levantine Mousterian fossil record. *Paléorient* **29**, 35–62 (2003).
97. Dean, M. C. *et al.* The distribution and biogenic origins of zinc in the mineralised tooth tissues of modern and fossil hominoids: Implications for life history, diet and taphonomy. *Biology* **12**, 1455 (2023).
98. Rak, Y., Kimbel, W. H. & Hovers, E. A Neandertal infant from Amud Cave, Israel. *J. Hum. Evol.* **26**, 313–324 (1994).
99. Hovers, E., Rak, Y., Lavi, R. & Kimbel, W. H. Hominid remains from Amud Cave in the context of the Levantine Middle Paleolithic. *Paléorient* **21**, 47–61 (1995).
100. Crevecoeur, I. *et al.* The Spy VI child: A newly discovered Neandertal infant. *J. Hum. Evol.* **59**, 641–656 (2010).
101. Walker, M. J., Lombardi, A. V., Zapata, J. & Trinkaus, E. Neandertal mandibles from the Sima de las Palomas del Cabezo Gordo, Murcia, southeastern Spain. *Am. J. Phys. Anthropol.* **142**, 261–272 (2010).
102. Turner C.G. II, Nichol C.R. & Scott G.R. in *Advances in Dental Anthropology* (eds M. Kelley & C. Larsen) 13-31 (Wiley Liss, 1991).
103. Villa, G. & Giacobini, G. Subvertical grooves of interproximal facets in Neandertal posterior teeth. *Am. J. Phys. Anthropol.* **96**, 51-62 (1997).
104. Poisson, P., Maureille, B., Couture, C., Tournepiche, J. F., Miquel, J. L. Contribution à l'étude des sillons sub-verticaux intéressant des facettes interproximales. Applications aux dents néandertaliennes de Rocherlot (Saint-Amant-de-Bonnieure, Charente, France). *Bull. Mem. Soc. Anthropol. Paris.* **14,** 75-87 (2002).
105. Estalrrich, A., Rosas, A., García-Vargas, S., García-Tabernero, A., Santamaría, D. & de la Rasilla, M. Brief communication: Subvertical grooves on interproximal wear facets from the El Sidrón (Asturias, Spain) Neanderthal dental sample. *Am. J. Phys. Anthropol.* **144**, 154-161 (2011).
106. Le Cabec, A., Gunz, P., Kupczik, K., Braga, J. & Hublin, J.-J. Anterior tooth root morphology and size in Neanderthals: Taxonomic and functional implications. *J. Hum. Evol.* **64**, 169–193 (2013).
107. Zanolli, C. & Mazurier, A. Endostructural characterization of the *H. heidelbergensis* dental remains from the early Middle Pleistocene site of Tighenif, Algeria. *C. R. Palevol* **12**, 293–304 (2013).
108. Maureille, B., Rougier, H., Houet, F. & Vandermeersch, B. Les dents inférieures du Néandertalien Regourdou 1 (site de Regourdou, commune de Montignac, Dordogne) : Analyses métriques et comparatives. *PALEO* **13**, 183–200 (2001).
109. Scolan, H., Santos, F., Tillier, A.-M., Maureille, B. & Quintard, A. Des nouveaux vestiges néanderthaliens à Las Pélénos (Monsempron-Libos, Lot-et-Garonne, France). *Bull. Mém. Société Anthropol. Paris* **24**, 69–95 (2012).
110. Smith, B. H. Patterns of molar wear in hunter-gatherers and agriculturalists. *Am. J. Phys. Anthropol.* **63**, 39–56 (1984).
111. Tattersall, I. & Sawyer, G. J. The skull of "Sinanthropus" from Zhoukoudian, China: a new reconstruction. *J. Hum. Evol.* **31**, 311-314 (1996).
112. Burnett S., Hawkey D.E. & Turner C.G. II. Brief communication: Population variation in human maxillary premolar accessory ridges (MxPAR). *Am. J. Phys. Anthropol.* **141**, 319-324 (2010)

113. Bailey S.E. *Neanderthal dental morphology: Implications for modern human origins.* Ph.D. Thesis, Department of Anthropology, Arizona State University, pp. 238 (2002)
114. Bailey S.E., Skinner M.M. & Hublin J.-J. What lies beneath? An evaluation of lower molar trigonid crest patterns based on both dentine and enamel expression. *Am. J. Phys. Anthropol.* **45**, 505-518 (2011)
115. de Pinillos *et al.* Trigonid crests expression in Atapuerca-Sima de los Huesos lower molars: Internal and external morphological expression and evolutionary inferences. *C.R. Palevol* **13**, 205-221 (2014).
116. de Pinillos M.M. *et al.* Comparative analysis of the trigonid crests patterns in *Homo antecessor* at the enamel and dentine surfaces. *Quart Int.* **4233**, 189-198 (2017)
117. Weidenreich F. The dentition of *Sinanthropus pekinensis*: A comparative odontography of the hominids. *Paleontologia Sinica* **D**, 1-180 (1937).
118. Zanolli C. *et al.* Brief communication: Two human fossil deciduous molars from the Sangiran Dome (Java Indonesia): outer and inner morphology. *Am. J. Phys. Anthropol.* **147**, 472-481 (2012).
119. Bermúdez de Castro J.M., *et al.* Early Pleistocene hominin deciduous teeth from the *Homo antecessor* Gran Dolina-TD 6 bearing level (Sierra de Atapuerca, Spain). *Am. J. Phys. Anthropol.* **163**, 1-14 (2017).
120. Bermúdez de Castro J.M. *et al.* Dental remains from Atapuerca – TD 6 (Gran Dolina site, Burgos, Spain). *J. Hum. Evol.* **37**, 523-566. (1999).
121. Bermúdez de Castro J.M. *et al.* The Atapuerca dental remains. New evidence (1987-1991 excavations) and interpretations. **24**, 339-371 (1993).
122. Bermúdez de Castro J. M. Dental remains from Atapuerca (Spain) I. Metrics. *J. Hum. Evol.* **15**, 265-287 (1986).
123. Gómez-Olivencia, A., Carretero, J.M., Arsuaga, J.L., Rodríguez-García, L., García-González, R. & Martínez, I. Metric and morphological study of the upper cervical spine from the Sima de los Huesos site (Sierra de Atapuerca, Burgos, Spain). *J. Hum. Evol.* **53**, 6–25 (2007).
124. Meyer, M.R. & Haeusler, M. Spinal cord evolution in early *Homo*. *J. Hum. Evol.* **88**, 43-53 (2015).
